# Phylogenetic relationships of †*Luisiella feruglioi* (Bordas) and the recognition of a new clade of freshwater teleosts from the Jurassic of Gondwana

**DOI:** 10.1186/s12862-015-0551-6

**Published:** 2015-12-03

**Authors:** Emilia Sferco, Adriana López-Arbarello, Ana María Báez

**Affiliations:** Laboratorio de Paleontología Evolutiva de Vertebrados, Departamento de Geología, Facultad de Ciencias Exactas y Naturales, Universidad de Buenos Aires, Ciudad Universitaria, Pabellón II, 1428 Buenos Aires, Argentina; CICTERRA-CONICET-UNC. Av. Velez Sarsfield 1611, X0516GCA Córdoba, Argentina; SNSB-Bayerische Staatssammlung für Paläontologie und Geologie and GeoBio-Center Ludwig Maximilian University, Richard-Wagner-Strasse 10, D-80333 Munich, Germany

**Keywords:** Teleostei, Jurassic teleosts, Teleost phylogeny, Freshwater teleosts, Gondwana

## Abstract

**Background:**

Teleosts constitute more than 99 % of living actinopterygian fishes and fossil teleosts have been studied for about two centuries. However, a general consensus on the definition of Teleostei and the relationships among the major teleostean clades has not been achieved. Our current ideas on the origin and early diversification of teleosts are mainly based on well-known Mesozoic marine taxa, whereas the taxonomy and phylogenetic relationships of many Jurassic continental teleosts are still poorly understood despite their importance to shed light on the early evolutionary history of this group. Here, we explore the phylogenetic relationships of the Late Jurassic (Oxfordian – Tithonian) freshwater †*Luisiella feruglioi* from Patagonia, in a comprehensive parsimony analysis after a thorough revision of characters from previous phylogenetic studies on Mesozoic teleosts.

**Results:**

We retrieved †*Luisiella feruglioi* as the sister taxon of the Late Jurassic †*Cavenderichthys talbragarensis*, both taxa in turn forming a monophyletic group with the Early Cretaceous †*Leptolepis koonwarri*. This new so far exclusively Gondwanan freshwater teleost clade*,* named †Luisiellidae fam. nov. herein, is placed outside crown Teleostei, as a member of the stem-group immediately above the level of †*Leptolepis coryphaenoides*. In addition, we did not retrieve the Late Jurassic †Varasichthyidae as a member of †Crossognathiformes. The position of †Crossognathiformes within Teleocephala is confirmed whereas †Varasichthyidae is placed on the stem.

**Conclusions:**

The general morphology of luisiellids is that of basal, stem Teleocephala; however, most of their synapomorphies have evolved independently in teleocephalans. Similarly, the resemblance between varasichthyids and crossognathiforms might be due to parallel evolution. In accordance to most teleostean phylogenies, our analysis shows that a major morphological change occurred along the stem line and are currently recorded at the level of †*Leptolepis coryphaenoides*. A stem-based total clade Teleostei has been accepted for this work.

**Electronic supplementary material:**

The online version of this article (doi:10.1186/s12862-015-0551-6) contains supplementary material, which is available to authorized users.

## Background

The Teleostei are the most speciose group of vertebrates, with more than 32,000 living valid species that constitute more than 99 % of Recent actinopterygian species [1]. The group shows enormous taxonomic diversity, together with a noteworthy variety of morphological features that have enabled life in very different aquatic habitats including freshwater, brackish, and marine environments [2, 3]. Although teleosts are important in modern ecosystems, a general consensus on the phylogenetic relationships among major teleostean lineages and on its timing of diversification has not been reached [4, 5]. According to available paleontological evidence teleosts had a modest beginning in the Triassic [6, 7] and underwent an extraordinary radiation throughout the Late Jurassic and Early Cretaceous (see the section Taxonomic framework and the names of higher clades, for a discussion on the definition of Teleostei adopted here). All five major clades of living teleosts (i.e. Elopomorpha, Osteoglossomorpha, Clupeomorpha, Ostariophysi, Euteleostei) are first recorded during that time [8].

Studies on fossil teleosts began in the 19^th^ century and continued in the 20^th^ century with the publication of numerous papers on new genera and species of Jurassic representatives, mainly from marine sediments of Europe [2]. The first hypotheses on teleostean phylogenetic relationships considering both fossil and living taxa and following Hennig’s methodology were proposed by Patterson [9] and Patterson and Rosen [10]. Some years later, Arratia [11] explored for the first time the phylogenetic relationships of Jurassic teleosts using cladistic principles and computer programs in a comparative study mainly focused on the caudal vertebrae and caudal skeleton of selected fossil and extant teleosts. Subsequently, Arratia [6, 7, 12–17] and Arratia and Tischlinger [18], produced a series of detailed anatomical descriptions of numerous Jurassic teleosts from Chile and Germany, together with cladistic analyses that have drawn our current ideas on the origin and early diversification of Teleostei. Other proposals on the relationships of Jurassic teleosts have not been based on computerized cladistic analyses [19, 20].

Although all these post-hennigian phylogenetic studies represent major improvements in our knowledge of teleostean phylogeny, they are strongly biased because of their almost exclusive sampling of marine teleosts of Europe and America and their lack of freshwater taxa. Before Arratia’s contributions, all previous studies included only a few marine teleost taxa from the Jurassic of Europe (†*Dorsetichthys,* †*Ichthyokentema*, †*Leptolepis*, †*Tharsis*, †*Thrissops*). Arratia added several marine teleosts from the Jurassic of Chile (†*Varasichthys*, †*Chongichthys*, †*Protoclupea*) and Cuba (†*Luisichthys*), and several teleost species from the palaeoarchipelago of Solnhofen in Germany (†*Ascalabos*, †*Ascalabothrissops*, †*Anaethalion*, †*Bavarichthys*, †*Eurycormus*, †*Leptolepides*, †*Orthogonikleithrus*, †*Siemensichthys*) to her data-set. Despite their importance, the taxonomic placement and phylogenetic relationships of the many Jurassic continental teleosts are still poorly understood and only †*Cavenderichthys talbragarensis* from Australia and †*Catervariolus hornemani* from the Democratic Republic of Congo have been included in computerized cladistic analyses [7, 11, 14]. Four monospecific genera from the Late Jurassic Talbragar Beds of Australia and †*Oreochima ellioti* from the Early Jurassic of Antarctica have been included in the family Archaeomaenidae, a group of basal teleosts according to Schaeffer [21], but their phylogenetic relationships have never been explored through a cladistic analysis. Likewise, although some of the many teleost taxa from continental strata of the Stanleyville beds of central Africa have been revised [19, 20, 22], most of these fishes have never been included in a cladistic analysis and the situation is not different for †*Hulettia americana* and †*Todiltia schoewei*, the teleosts from the Sundance and Wanakah formations of North America, respectively [23]. Other Jurassic freshwater teleosts from North America [24], South America [25], and Asia [26] are even more poorly known.

From the foregoing, it is evident that our current ideas on the phylogeny of early Mesozoic teleosts are mainly based on the Jurassic marine teleosts of Europe, Chile, and Cuba. Phylogenetic studies of Triassic and Jurassic teleosts from other continents, and especially freshwater taxa, are needed to have a more comprehensive scenario of the early evolutionary history of Teleostei. Compared to other vertebrate groups, early Mesozoic teleosts are underrepresented in cladistic analyses, which is certainly an important flaw. The benefits of incorporating fossils in phylogenetic analyses have been masterfully summarized by Donoghue and coauthors [27]. Adding taxa, fossil or not, to a cladistic analysis might change the pattern of relationships and the ideas of character evolution. In particular, adding fossils that represent basal taxa and are nearer to the nodes, as is the case of early Mesozoic teleosts, might have important implications in elucidating phylogenetic relationships of living groups by breaking artifacts like long-branch attraction [28, 29], especially in cases of character exhaustion [30]. The recent taxonomic revision of the small-sized freshwater teleost †*Luisiella feruglioi* (Bordas, 1942) [31], from the Upper Jurassic of Patagonia provided detailed information on its skeletal anatomy [32]. Based on this new information, the present study is aimed to investigate the phylogenetic relationships of †*Luisiella feruglioi* in a comprehensive parsimony analysis including 29 Jurassic taxa, two freshwater species among them, in the taxonomic sampling.

## Methods

### Taxonomic framework and the names of higher clades

**Fig. 1:**
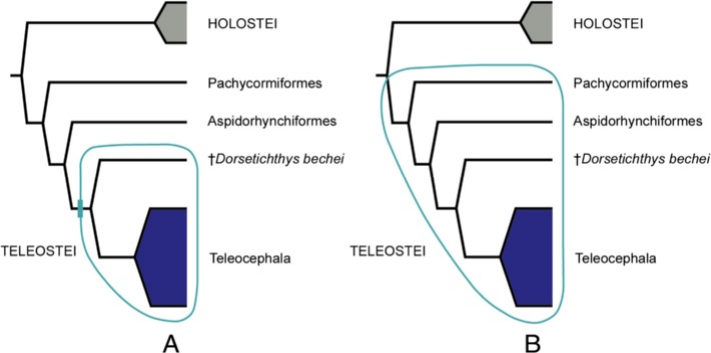
Different definitions of Teleostei represented on a simplified cladogram [4]. **a**. Total clade Teleostei sensu De Pinna [33]; **b**. Apomorphy-based clade Teleostei sensu Arratia [15]. Since the cladogram of Near et al. [4] includes only living taxa, †*Dorsetichthys bechei*, †Pachycormiformes, and †Aspidorhynchiformes have been added manually according to Patterson [9] and Arratia [15]

Although the monophyly of extant teleosts and their close phylogenetic relationships to several fossil taxa is generally well established and widely accepted, the delimitation of Teleostei has been problematic [33, 34]. Due to the long tradition of essentialist thinking in taxonomy [35], after the original definition of Müller [36] and the recognition of the close phylogenetic relationships of some early Mesozoic taxa with living teleosts, many authors attempted to delimit Teleostei on the basis of shared derived traits [9, 10, 15, 37–43]. The disadvantages of apomorphy-based definitions have been extensively discussed (e.g. [35, 44]) and recently De Queiroz [45] stressed the feasibility of using a stem-based (branch-based, maximum clade) definition to define the name of a total clade. In the same line of thought, De Pinna ([33]: 150) had proposed a clear and stable stem-based definition of Teleostei as follows: “Teleostei is here defined … as the largest (i.e. most inclusive) actinopterygian clade not including either the Halecomorphi (*Amia* and close relatives) and/or the Ginglymodi (*Lepisosteus* and close relatives)” (Fig. 1a). This total group definition was also that applied by Patterson [9]. Arratia [15], however, subsequently presented an apomorphy-based definition of Teleostei, which has been adopted by many authors (e.g. [46–48]). Teleostei sensu Arratia (Fig. 1b) is more restricted than the definition proposed by De Pinna [33] because “including all taxa down to †*Pholidophorus bechei*” ([15]: 323) excludes several fossil taxa such as pachycormiforms, aspidorhynchiforms, and pycnodontiforms, although it was already accepted that they are more closely related to Recent teleosts than they are to *Amia* or *Lepisosteus*. This apomorphy-based definition depends on the features of the taxa that are added or excluded from the base of Teleostei (e.g. compare [7] with [15]). Conversely, the total clade definition of Teleostei sensu De Pinna [33] is conceptually sound and stable. The name Teleosteomorpha proposed by Arratia [49] to include her Teleostei and all its stem taxa (e.g. pachycormiforms, aspidorhynchiforms, pycnodontiforms) is redundant because it is equivalent to Teleostei sensu Patterson [9] or De Pinna [33].

Comparison of the definitions of Teleostei by De Pinna [33] and by Arratia [15], shows, as commented on by the former author, that the name Teleostei has always been bound to a concept opposed to either Holostei, Halecomorphi or Ginglymodi and, thus, his stem-based definition of Teleostei more properly reflects the Teleostei as conceived by Müller [36] even when stem-group teleost taxa were not considered in the classification of teleosts at that time. De Pinna [33] further named the clade including all living teleosts and its fossil representatives as Teleocephala (Fig. 1a). As explained by De Queiroz [45] this node-based definition should be stable and independent of our knowledge of a species representing the immediate sister taxon of the crown clade Teleostei. Herein we accept the node-based clade Teleocephala and the stem-based clade Teleostei of De Pinna [33], considering the name Teleosteomorpha redundant.

### Taxonomic sampling and nomenclature

The investigation of the phylogenetic relationships of †*Luisiella feruglioi* was performed through a parsimony analysis of a matrix of 178 morphological characters scored for 61 taxa (46 extinct and 15 living taxa). In addition to the taxa sampled by Arratia and Tischlinger [18], our matrix includes eight other Mesozoic teleostean species: the two well-known Australian freshwater taxa †*Cavenderichthys talbragarensis* from the Late Jurassic Talbragar Beds and †*Leptolepis koonwarri* from the Early Cretaceous Koonwarra Beds; the Cretaceous †*Tharrhias araripis* from the Brazilian Araripe Basin; and †*Pholidophorus latiusculus*, †*Pholidophorus gervasuttii*, †*Siemensichthys siemensi*, †*Siemensichthys macrocephalus* and †*Eurycormus speciosus* from the Upper Triassic and Upper Jurassic of Europe.

According to previous phylogenetic hypotheses of relationships involving Jurassic teleosts [17, 18], the halecomorphs *Amia calva* and †*Amia pattersoni*, the lepisosteiforms *Lepisosteus osseus* and †*Obaichthys decoratus*, and the stem-group teleost taxa †*Mesturus verrucosus* (Pycnodontiformes), †*Aspidorhynchus acutirostris*, †*Belonostomus tenuirostris* and †*Vinctifer comptoni* (†Aspidorhynchiformes), and †*Pachycormus macropterus* and †*Hypsocormus macrodon* (†Pachycormiformes) were chosen as outgroup taxa.

Taxonomic names are used, proposed and/or defined according to the rules and recommendations of the International Code of Zoological Nomenclature [50].

### Character coding and scoring

Numerous characters (118) were taken from Arratia’s phylogenetic analyses [6, 7, 11–18] and other systematic studies including living and fossil neopterygians [3, 9, 51–64]. Most of the remaining characters have been modified from their original definitions, whereas a new set of seven characters (47, 58, 76, 99, 112, 120 and 171) are proposed herein or used for the first time in a cladistic analysis. The complete list and discussions of characters are given in the Additional file 1.

Most of the emended definitions of characters are based on a thorough revision of primary homology hypotheses taking special care to avoid those definitions that imply the use of unspecified “absence” character states [65]. According to Jenner ([65]: 5) “absence/presence coding (a/p coding) is perfectly legitimate when the goal is to express whether a feature is simply absent or present among the taxa of interest”. However, many a/p characters often do not represent the absence/presence of a feature, but of a characteristic of a certain feature. In these cases, the “absence” state might be grouping on the basis of non-homologous absences, as it can be scored for taxa with very dissimilar morphologies. Unspecified “absence” states may result from not recognizing inapplicable character states that are simply scored as “absent”, or it results from not recognizing a multistate variation and, thus, the different conditions that are not expressed by the “presence” state are inappropriately united with unrelated morphologies [65]. The occurrence of character definitions involving an unspecified “absence” state is very common in metazoan literature and frequently found in fossil fish literature (e.g. [17, 18, 52, 54, 56, 57], among others). The main problem of the use of unspecified character states in phylogenetics is the incorrect suggestion of common ancestry and similarity in morphologically dissimilar taxa, and the assumption that the disparate morphologies grouped within this state are a clear alternative to the other character state [65].

Whenever possible character scoring was based on examination of adult specimens housed in different European and Argentinian paleontological and zoological collections (see list of examined material in Additional file 2). Character scoring was based on descriptions in the literature if the material was not available to us (see also list of literature in Additional file 2). The data matrix was prepared with Mesquite Version 2.75 [66].

The following institutions gave permissions to access their collections: BSPG, Bayerische Staatssammlung für Paläontologie und Geologie, Munich, Germany; CPBA-V, Vertebrate Paleontology Collection, Universidad de Buenos Aires, Argentina; JME-ETT, Jura-Museum Eischttät, Germany (Ettling); JME-SOS, Jura-Museum Eischttät, Germany (Eischttät); MACN, Museo Argentino de Ciencias Naturales “Bernardino Rivadavia”, Buenos Aires, Argentina; MB. f. Museum für Naturkunde, Berlín, Germany; MLP, Museo de La Plata, La Plata, Argentina; MPEF-PV, Museo Paleontológico Egidio Feruglio – Colección de Paleovertebrados, Trelew, Argentina; NHMUK, Natural History Museum, London, UK; TRF, Helmut Tischlinger, private collection, Germany; UBA, Universidad de Buenos Aires, Vertebrate Collection, Buenos Aires, Argentina.

### Cladistic methodology

Tree search was performed through the traditional search option of TNT v. 1.1 [67] applying random addition sequence (RAS) and tree bisection reconnection (TBR) through 1000 replicates keeping 10 trees per replicate. Additionally, TBR was applied to all the trees retained in memory. All characters were considered unordered and equally weighted. The most parsimonious trees (MPTs) were rooted at the Holostei (Halecomorphi, Ginglymodi) based on all recent morphological (e.g. [63]) and molecular (e.g. [4, 68, 69]) phylogenetic analyses of actinopterygian relationships. Branch support was evaluated using the Jackknife method and calculating decay indexes for each node (Bremer support). The Jackknife was run with TNT [67] through 1000 replicates and the values were expressed as GC values (Groups present/contradicted) with a probability of change of 0.36, which is the default value assigned by the program. Bremer support was calculated manually through successive iterations using TNT. The taxon-character matrix, most parsimonious trees, and strict consensus tree are available in the Additional file 3. The reduced consensus tree was obtained with the agreement subtrees algorithm of PAUP* 4.0 beta [70].

The distribution of characters was analysed using the ‘trace character history’ option in Mesquite v. 2.75. Only the unambiguous synapomorphies were taken into account, discriminating between unique and non-unique synapomorphies. Unique synapomorphies are those features that derive only once in the tree whereas non-unique synapomorphies are homoplastic [71].

## Results and discussion

### Phylogenetic analysis

**Fig. 2:**
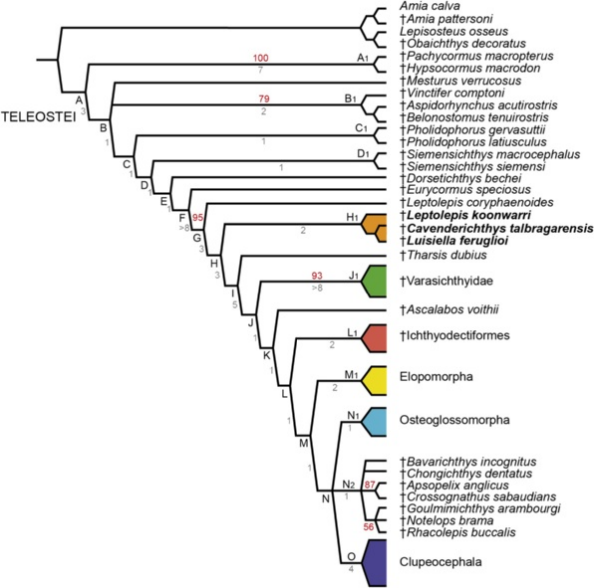
Simplified strict consensus tree of 16 most parsimonious trees. Numbers given on branches: Bremer values in gray; Jackknife frequencies in red

The cladistic analysis resulted in 16 most parsimonious trees (MPTs) of 872 steps, with a consistency index of 0.285 and a retention index of 0.640. The strict consensus of the 16 most parsimonious trees is shown in Fig. 2. The consensus shows a generally well-resolved and monophyletic Teleostei, with only a few unresolved internal nodes (N and N2). Bremer values for each clade and Jackknife values higher than 50 are shown in the same figure. Apart from the taxonomic placement and relationships of †*Luisiella feruglioi* and †*Leptolepis koonwarri*, which are explored for the first time herein, with regard to the interrelationships of basal teleosts our consensus differs from recent cladistic hypotheses (i.e. [7, 17, 18]) in the lack of close relationship between the varasichthyids and the crossognathids and pachyrhyzodontoids of the clade N2. It also differs in the position of a few basal teleost taxa (e.g. †*Siemensichthys siemensi*, †*Siemensichthys macrocephalus*, †*Eurycormus speciosus* and †*Dorsetichthys bechei*). In addition, the position of the Esociformes (represented by *Esox lucius* and *Umbra krameri*) outside Euteleostei (see Additional file 3) is noteworthy. This position, however, might be due to a bias in our data matrix, which was meant to explore relationships among basal, non-teleocephalan teleosts. The inclusion of esociforms in Euteleostei is well supported by numerous phylogenetic studies (e.g. [59, 68, 72–76]).

Within Teleocephala, our hypothesis is in accordance with the studies mentioned above and other morphologic (e.g. [3]) as well as molecular-based phylogenetic hypotheses (e.g. [4, 68]), in which Elopomorpha is sister to the remaining groups of extant teleosts (i.e. Osteoglossomorpha, Clupeomorpha, Ostariophysii, and Euteleostei).

### Systematic position of †*Luisiella feruglioi*

†*Luisiella feruglioi* is recovered as the sister taxon of the Late Jurassic †*Cavenderichthys talbragarensis* (Node H2), both taxa in turn forming a monophyletic group with †*Leptolepis koonwarri* (Node H1). This small clade is placed outside the crown group Teleocephala (Node L), immediately above the level of †*Leptolepis coryphaenoides* but more basal than †*Tharsis dubius*, the Jurassic †Varasichthyidae, †*Ascalabos voithii*, and the †Ichthyodectiformes (Fig. 2).

### Node H1 ((†*Luisiella feruglioi*, †*Cavenderichthys talbragarensis*) †*Leptolepis koonwarri*)

This clade is supported by three unambiguous but not unique synapomorphies representing relatively derived conditions among basal teleosts (Fig. 2).

**Fig. 3:**
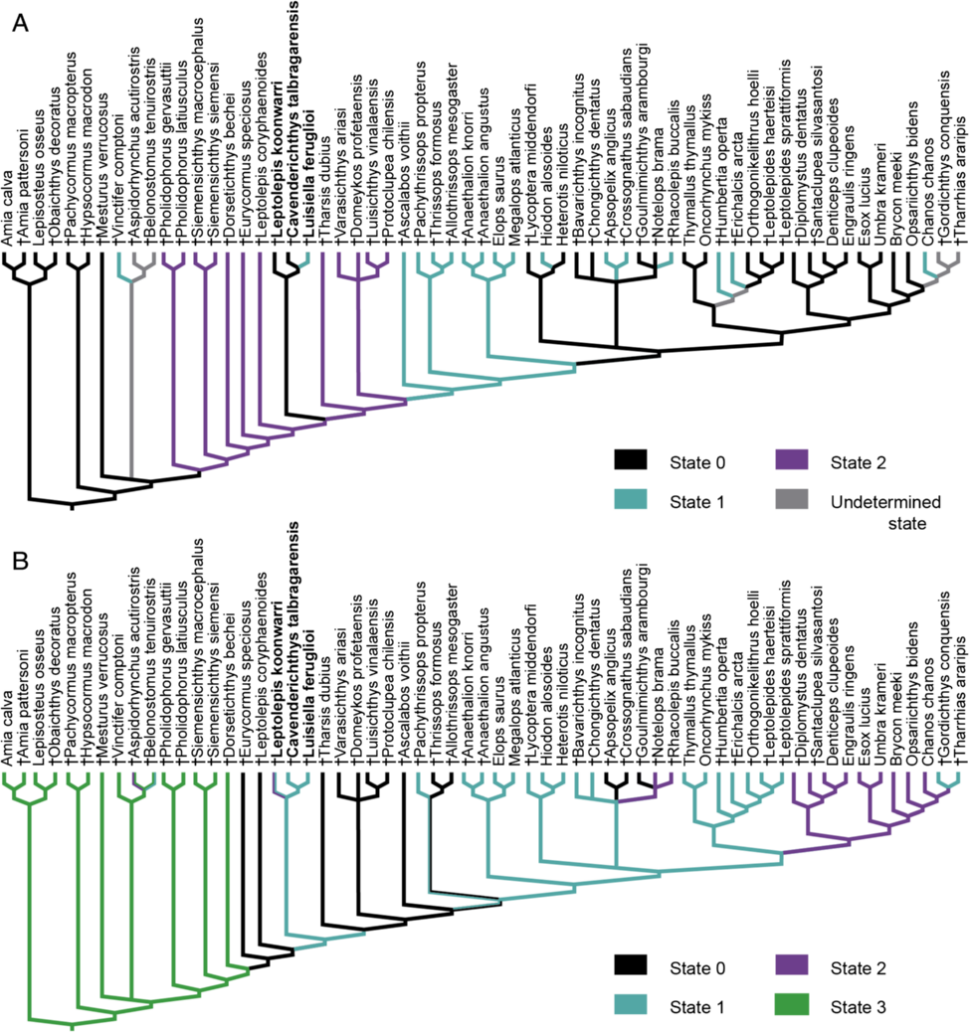
Traced history of characters 83 and 145. **a**. Character 83 – Composition of preopercular sensory canal: four or less, short simple tubules [0], seven or eight (up to 10), short simple or branched tubules [1], 12 or more long, simple or branched tubules [2]; **b**. Character 145 – Anterior extent of first uroneural: preural centra 4 or 3 [0], preural centrum 2 [1], preural centrum 1 [2], centra U1 or U2 [3]

Character 83{2 → 0}: presence of four or less simple tubules in the preopercular sensory canal. The variation of the tubules of this canal was summarized in three different states (four or less, short simple tubules {0}, seven or eight (up to 10), short simple or branched tubules {1}, at least 12 long, simple or branched tubules {2}; see Additional file 1). This character has a parabolic distribution in the consensus tree (Fig. 3a). From the base to the top there is first a trend towards an increase in the number of tubules of the preopercular sensory canal from four or less tubules in the holosteans and †*Mesturus* within the outgroup, to seven or eight tubules in †*Vinctifer*, up to at least 12 tubules in the basal teleosts †*Eurycormus*, †*Siemensichthys*, †*Pholidophorus*, †*Dorsetichthys bechei*, †*Leptolepis coryphaenoides*, †*Tharsis dubius*, and the Jurassic varasichthyids. Above the level of †Varasichthyidae, the trend reverts to a decrease in the number of tubules, with a reversal to four or less tubules in most of the teleocephalans more closely related to osteoglossomorphs and clupeocephalans (Clupeomorpha, Ostariophysi, Euteleostei) than to elopomorphs, passing through the intermediate state of seven or eight tubules in †*Ascalabos*, ichthyodectiforms, and elopomorphs. Interestingly, the presence of only four or less simple tubules in the preopercular sensory canal is independently derived in the clade formed by †*Luisiella feruglioi*, †*Cavenderichthys talbragarensis* and †*Leptolepis koonwarri*. Likewise, the intermediate number of seven or eight tubules in † *Luisiella feruglioi* [32] is independently derived within this clade and in †*Ascalabos*, ichthyodectiforms and crown group teleosts.

Character 143{0 → 1}: presence of six uroneurals in the caudal fin endoskeleton. The presence of uroneural bones is uniquely derived in Teleostei and appears first in the aspidorhynchiforms, which have a total of three elements (character 143{3}). Uroneurals are unknown in †*Pholidophorus* and †*Siemensichthys*, their total number is highest in the closely related basal teleosts †*Dorsetichthys*, †*Eurycormus*, and †*Leptolepis coryphaenoides* as well as in more crownward taxa like †*Tharsis*, †*Ascalabos*, †*Thrissops,* and †*Allothrissops*. The number of uroneurals is highly variable though always relatively low within Teleocephala and a general trend towards the reduction of these bones is evident. According to Patterson [77], this trend might be the consequence of the loss or fusion of elements in post-Jurassic teleosts. Among non-teleocephalan teleosts, the clade formed by †*Luisiella feruglioi*, †*Cavenderichthys talbragarensis* and †*Leptolepis koonwarri* stands out because of the relatively low number of six uroneurals (character 143{1}).

Character 145{0 → 1} first uroneural extending anterior to the second preural centrum. In the caudal endoskeleton of teleosts, the first uroneural extends above the ural centra reaching anteriorly only up to the first and second ural centra {3}, the first preural centrum {2}, the second preural centrum {1}, or up to the third or fourth preural centra {0}. The phylogenetic relationships depicted in the strict consensus tree imply a general trend towards the shortening of the uroneural within Teleocephala. As for characters 83 and 143, the clade formed by †*Luisiella feruglioi*, †*Cavenderichthys talbragarensis*, and †*Leptolepis koonwarri* presents a peculiar condition among non-teleocephalan teleosts (except †*Pachythrissops*; Fig. 3b), in which the first uroneural only reaches the second preural centrum {1}, instead of the third or fourth preural centra {0} (except the aspidorhynchiforms, †*Dorsetichthys,* and †*Pachythrissops*). This condition appears independently in Teleocephala.

### Node H2 (†*Luisiella feruglioi*, †*Cavenderichthys talbragarensis*)

The sister group relationship between †*Luisiella feruglioi* and †*Cavenderichthys talbragarensis* is supported by two unambiguous but not unique synapomorphies (Fig. 2).

**Fig. 4:**
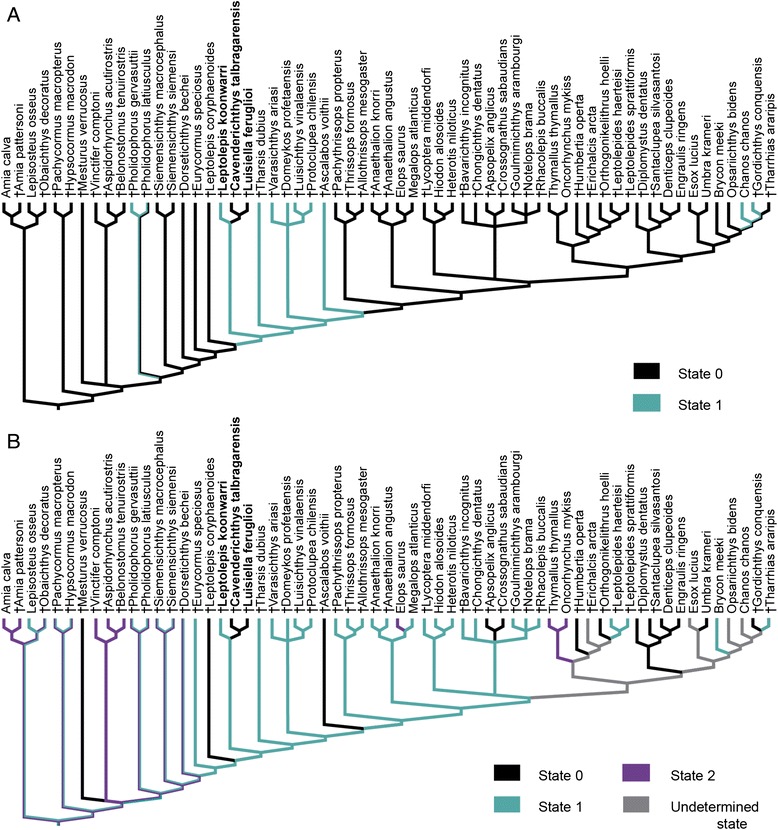
Traced history of characters 75 and 125. **a**. Character 75 – Characteristic notch (so-called leptolepid notch) in the anterodorsal ascending margin of the dentary: absent [0]; present [1]. **b**. Character 125 – Number of vertebrae (including preural centrum 1): fewer than 45 [0], between 45 and 65 [1], more than 65 [2]

Character 75{1 → 0}: absence of a “leptolepid” notch in the anterodorsal, ascending margin of the dentary. The presence of a small notch (called “leptolepid” notch because it was thought to be typical of †*Leptolepis*) characterizes many basal teleost taxa, such as †*Leptolepis coryphaenoides*, †*Ascalabos voithii*, †*Tharsis dubius*, and the varasichthyids ([14, 78, 79]; Fig. 4a). Among non-teleocephalan teleosts, the absence of the “leptolepid” notch is not unique to the clade formed by †*Luisiella feruglioi* and †*Cavenderichthys talbragarensis*, as a notch is also lacking in †*Dorsetichthys bechei*, †*Siemensichthys*, †*Eurycormus*, and in the †Ichthyodectiformes [10]. Within Teleocephala, the general condition is the lack of a notch, which was only reported for some ostariophysan gonorhynchiform taxa and was thus proposed as a synapomorphy of the family Chanidae (e.g. *Chanos*, †*Gordichthys*; [80]).

Character 125{1 → 0}: fewer than 45 vertebrae in the column. The variation in the number of vertebrae (including preural centrum 1) is summarized in the character 125 with three states: fewer than 45 {0}, between 45 and 65 {1}, more than 65 {2}. The occurrence of fewer than 45 vertebrae is unusual among teleosts, which usually have between 45 and 65 vertebrae (see character discussion in the Additional file 1). Among non-teleocephalans, fewer than 45 vertebrae is a condition independently derived in the sister taxa †*Luisiella feruglioi* and †*Cavenderichthys talbragarensis*, the Jurassic †*Leptolepis coryphaenoides* and †*Ascalabos voithii*, and in †*Mesturus* ([14, 46]; Fig. 4b). Within Teleocephala, a vertebral column with fewer than 45 vertebrae occurs in †Crossognathidae (†*Apsopelix*, †*Crossognathus*) and is variably present within Clupeomorpha (Fig. 4b).

Although most synapomorphies at Node H1 are derived conditions absent in other stem teleocephalans (except for †*Pachythrissops* in many cases; Figs. 3, 4), the overall morphology of †*Luisiella feruglioi*, †*Cavenderichthys talbragarensis* and †*Leptolepis koonwarri* resembles that of other basal teleosts because of the presence of numerous plesiomorphic traits. These plesiomorphic traits include a hyomandibular bone bearing a preopercular process, the presence of a small quadrangular or semicircular extrascapular bone; an anteorbital sensory canal present; presence of a gular plate; presence of anterior processes on caudal preural neural and haemal arches; unfused parhypural; three or four uroneurals extending forward beyond the second ural centrum; 8 or more hypurals; epaxial basal fulcra in caudal fin; absence of epaxial procurrent rays; presence of dorsal processes at the bases of innermost principal caudal fin rays; one to five fringing fulcra in the caudal fin; dorsal caudal principal rays located obliquely to the main axis of hypurals and expanded anteriorly crenulated bases of the innermost principal caudal fin rays.

The clade formed by †*Luisiella feruglioi*, †*Cavenderichthys talbragarensis,* and †*Leptolepis koonwarri*, as well as the sister-taxon relationship between †*L. feruglioi* and †*C. talbragarensis* has low support values, but the Bremer and Jackknife values obtained for all internal teleostean nodes are generally low (Fig. 2). This is probably due to the high level of homoplasy evidenced by most characters (80 %) supporting the internal nodes of Teleostei, in a relatively large character-taxon matrix with several missing entries. Because of these generally low support values, we have tested alternative positions for †*Luisiella feruglioi* through constrained analysis in TNT, forcing it as sister taxon to some phylogenetically close basal teleost taxa, like †*Leptolepis coryphaenoides*, †*Tharsis dubius*, and the varasichthyids (Node J1). Each of these alternative positions resulted in suboptimal topologies longer than the 872 steps obtained in the MPTs of the unconstrained analysis. Most parsimonious trees of 878 steps each (six steps more than the MPTs of the original analysis) were obtained for the forced monophyly of †*Luisiella feruglioi* and †*Tharsis dubius*. Even longer trees of 879 steps and 880 steps, resulted after forcing the sister group relationship with †*Leptolepis* coryphaenoides and the varasichthyids, respectively. Therefore, the position of †*Luisiella feruglioi* as the sister taxon of †*Cavenderichthys talbragarensis* within a clade together with †*Leptolepis koonwarri* is by far the most parsimonious hypothesis of phylogenetic relationships for this Patagonian fish. Accordingly, we propose the family name †Luisiellidae, with type genus †*Luisiella* Bocchino [81], for the clade ((†*Luisiella feruglioi*, †*Cavenderichthys talbragarensis*) †*Leptolepis koonwarri*).

**Fig. 5:**
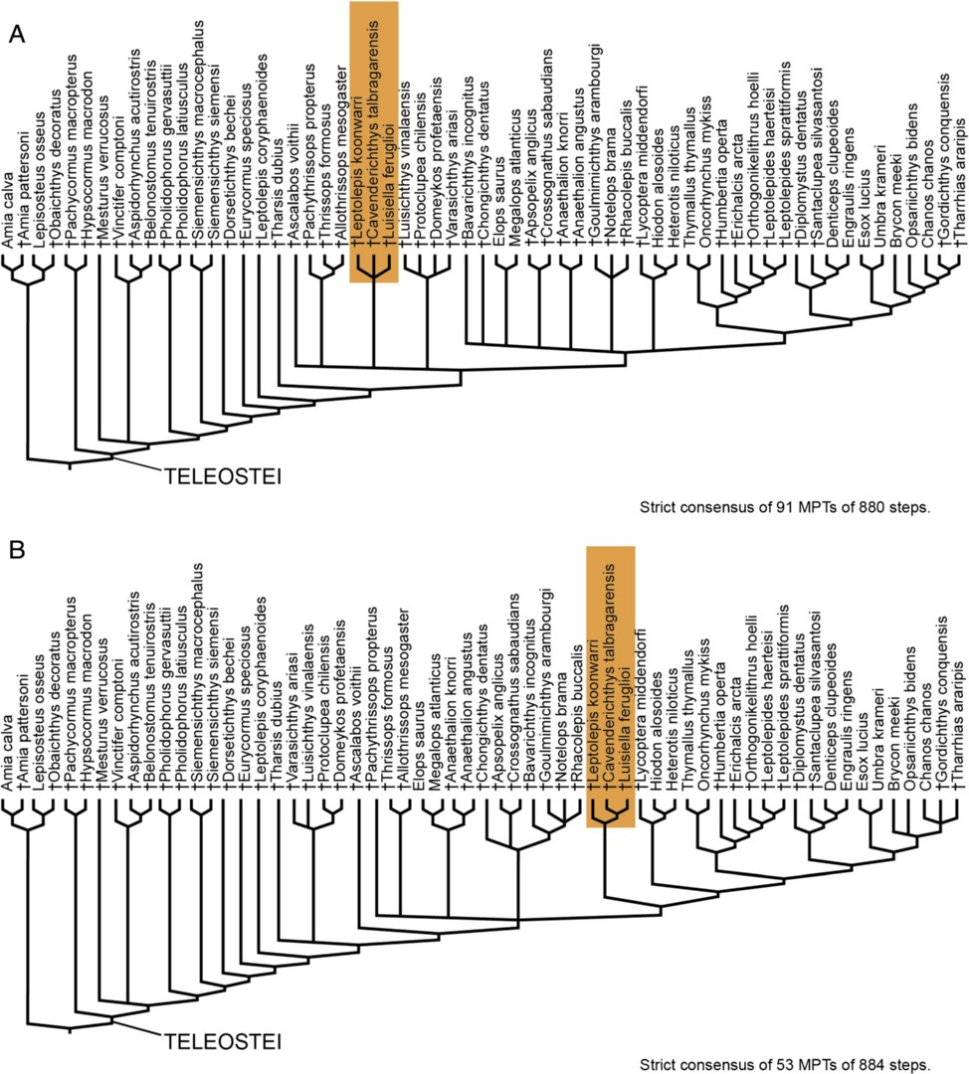
Alternative hypotheses for the relationships of †Luisiellidae fam. nov. **a**. Strict consensus forcing †Luisiellidae fam. nov. as sister group of Teleocephala. **b**. Strict consensus forcing the inclusion of †Luisiellidae fam. nov. within Teleocephala, as sister group of Osteoglossomorpha + Clupeocephala

†Luisiellidae fam. nov. is a Gondwanan clade including only freshwater fishes from Argentina and Australia (Figs. 2, 6) and the sister group relationship between †*L. feruglioi* and †*C. talbragarensis*, both recovered from Late Jurassic beds, indicates that this teleost clade was present in continental environments probably before the separation of East and West Gondwana at the initial stages of the break-up of this megacontinent during the Early Jurassic [82]. The position of †*Leptolepis koonwarri* within the clade †Luisiellidae fam. nov. and its lack of close phylogenetic relationship with †*Leptolepis coryphaenoides* clearly indicate that †”*Leptolepis*” *koonwarri* is not a species of †*Leptolepis* thus leading us to the erection of the nominal genus †*Waldmanichthys* (after Michael Waldman who discovered this fish, and the Greek “ichthys” = fish) clearly indicating the distinct taxonomic status of †*Waldmanichthys koonwarri* (Waldman, 1971; [83]).

We also performed constrained analyses forcing alternative positions of †Luisiellidae fam. nov. to further evaluate the robustness of the phylogenetic relationships of the clade retrieved in our previous analysis. In a first analysis we forced †Luisiellidae fam. nov. as sister group of Teleocephala (i.e. more closely related to the latter clade than to †Ichthyodectiformes) whereas in a second analysis, we forced its inclusion within Teleocephala, as sister group of Osteoglossomorpha + Clupeocephala. Both analyses resulted in much longer MPTs than 872 steps (880 and 884 steps respectively). Also, the first analysis produced less resolution with two major polytomies (†Luisiellidae, †Varasichthyidae, †*Ascalabos voithii*, †Ichthyodectiformes (†*Chongichthys* †*Bavarichthys*, Elopidae, †*Anaethalion*, †Crossognathidae, †Pachyrhizodontoidea, Osteoglossomorpha, Clupeocephala)) and Teleocephala was not retrieved (Fig. 5a). Likewise, the inclusion of †Luisiellidae fam. nov. in Teleocephala produced a polytomy (†Ichthyodectiformes, Elopomorpha, †Crossognathiformes (†Luisiellidae, Osteoglossocephala)) (Fig. 5b).

**Fig. 6:**
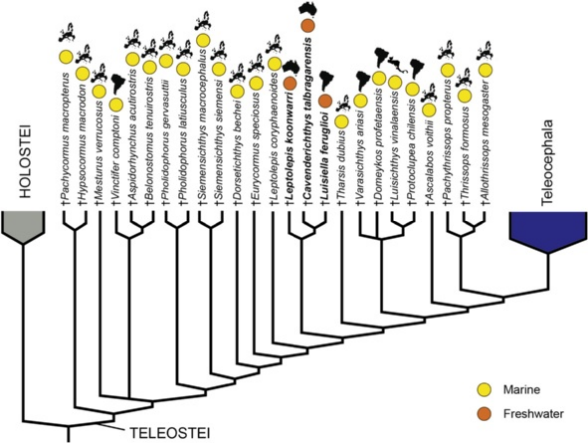
Distribution of marine and freshwater taxa on the stem Teleocephala

### Systematic status of the †Crossognathiformes

The family †Crossognathidae was erected by Woodward [84]. After many taxonomic revisions only two Cretaceous monotypic genera are included in the family: †*Crossognathus* Pictet and †*Apsopelix* Cope (see [10] for a detailed taxonomic compilation). The systematic position of †Crossognathidae was discussed by many authors (e.g. [10, 85, 86]) who pointed out the presence of some features shared with elopomorphs but also some in common with clupeomorphs and euteleosts, thus not being able to indicate the position of this family within the crown group. Also at that time, the extinct Cretaceous teleosts †*Notelops* Woodward, †*Rhacolepis* Agassiz, and †*Pachyrhizodus* Dixon were grouped in the Suborder †Pachyrhizondontoidei by Forey [87], who considered the clade as Teleostei *incertae sedis*. The †Crossognathidae (i.e. †*Crossognathus* and †*Apsopelix*) together with the pachyrhizodontoids were grouped in the Order †Crossognathiformes by Taverne [88] who based his proposal on the presence of a few shared synapomorphies: closed circumorbital ring, first and second hipurals fused with the first ural centrum of the caudal endoskeleton, and the occurrence of two epurals in the caudal fin. Taverne [88] concluded that his newly proposed order †Crossognathiformes is the sister group of a clade including Clupeomorpha and Euteleostei. Some years later, Patterson [89] added the suborder †Tselfatioidei to the †Crossognathiformes and regarded this order as Clupeocephala *incertae sedis*.

The monophyly of †Crossognathiformes sensu Taverne was tested in a phylogenetic analysis by Cavin [60], resulting in a paraphyletic †Crossognathiformes, though †Crossognathidae and †Pachyrhizodontoidea were both recovered as monophyletic groups. In Cavin’s analysis †Crossognathidae were basal to *Elops* (which was recovered as the most basal elopomorph within a paraphyletic Elopomorpha) whereas the †Pachyrhizodontoidea (including the Cretaceous †*Goulmimichthys* Cavin) were included within Clupeocephala, without a close relationship with elopomorphs. Cavin [60] argued that the †Crossognathidae share with the Jurassic †Varasichthyidae some of the synapomorphies proposed by Arratia [14, 15] for this later family. Cavin [60] and Cavin and Grigorescu [90] hence suggested a possible sister group relationship between †Crossognathidae and †Varasichthyidae. More recently, Arratia [17] retrieved these close phylogenetic relationships in a cladistic analysis and thus added the †Varasichthyidae to the †Crossognathiformes. Additionally, this expanded †Crossognathiformes are placed outside Teleocephala in Arratia’s [17] and Arratia and Tischlinger’s [18] trees, at the more basal position on the stem of Teleocephala previously occupied only by the †Varasichthyidae. It is thus noteworthy that in a phylogenetic analysis performed by Mayrinck et al. [91], the inclusion of a Cretaceous teleost, †*Salminops ibericus*, to Arratia and Tischlinger’s character-taxon matrix, produced major changes in the tree topology, including the complete dissociation of the †Crossognathiformes, with †Varasichthyidae, †Crossognathidae and †Pachyrhizodontoidea taking different positions in the phylogeny [91].

**Fig. 7:**
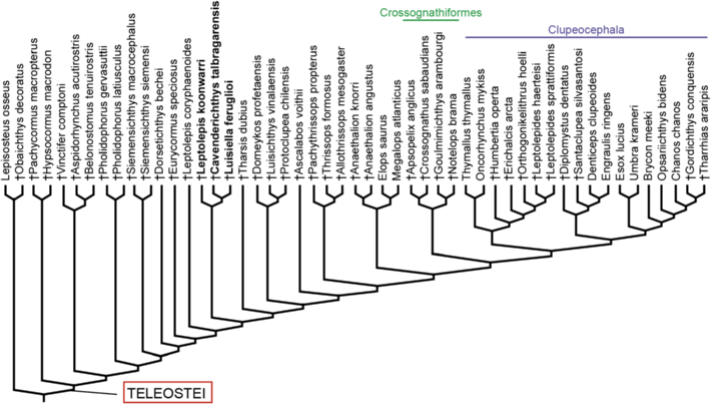
Reduced consensus tree. Osteoglossomorphs have been deleted as well as other unstable taxa including the Jurassic †*Varasichthys*, †*Bavarichthys* and †*Chongichthys*, and the Cretaceous †*Rhacolepis*

Our results do not support the inclusion of †Varasichthyidae in †Crossognathiformes proposed by Arratia [17]. Instead, in agreement with most phylogenetic studies prior to Arratia [17], we retrieve the clade †Varasichthyidae (Node J1) as a member of the stem Teleocephala together with other basal teleosts, and, in agreement with Taverne [88] and Patterson [89], the clade †Crossognathiformes including †Pachyrhizodontoidea, †Crossognathidae, †*Bavarichthys,* and †*Chongichthys* within Teleocephala. In our phylogenetic hypothesis the clade †Crossognathiformes is more closely related to osteoglossomorphs and clupeocephalans than they are to elopomorphs, actually forming part of an unresolved tricotomy with osteoglossomorphs and clupeocephalans (Fig. 2). In the reduced consensus the †Crossognathiformes sensu Taverne [88] is the sister group of Clupeocephala (Fig. 7).

The position of †Varasichthyidae as a stem Teleocephala is consistent with the general morphology present in these basal teleosts: e.g. presence of an antorbital sensory canal, “leptolepid” notch in the dentary, 12 or more tubules in the preopercular sensory canal, anterior processes on caudal preural, neural, and haemal arches, epaxial basal fulcra in the caudal fin, one to five fringing fulcra preceding the caudal fin, 20 or more caudal principal rays, dorsal caudal principal rays located oblique to main axis of hypurals, dorsal processes at the bases of innermost principal caudal fin rays of upper lobe, expanded, anteriorly crenulated bases of the innermost caudal fin rays. On the other hand, the position of the †Crossognathiformes within Teleocephala is consistent with the occurrence of many derived traits in this clade: e.g. absence of an independent antorbital bone, reduction in the number of infraorbital bones to four elements, expanded posterior infraorbitals overlapping the anterior margin of preopercle, presence of two epurals (except †*Bavarichthys* which has three epurals) and absence of caudal epaxial basal fulcra, among others.

The morphological resemblance between †Crossognathidae and †Varasichthyidae noted by previous authors is confirmed by our own observations. These taxa share several features, including the presence of numerous (eight or more) hypurals, four to six uroneurals and a first uroneural reaching anteriorly the third preural centrum [60, 90]. Furthermore, a posttemporal fossa framed by the epiotic, pterotic, exoccipital, and intercalar, a broadly expanded ventroposterior region of the preopercle, and a large, approximately triangular, caudally expanded extrascapular are present almost exclusively in both †Varasichthyidae and †Crossognathiformes [17, 18]. Nonetheless, according to our results, these features evolved independently in the two clades, the resemblance being due to parallelism [92].

### Early evolution of Teleostei

**Fig. 8:**
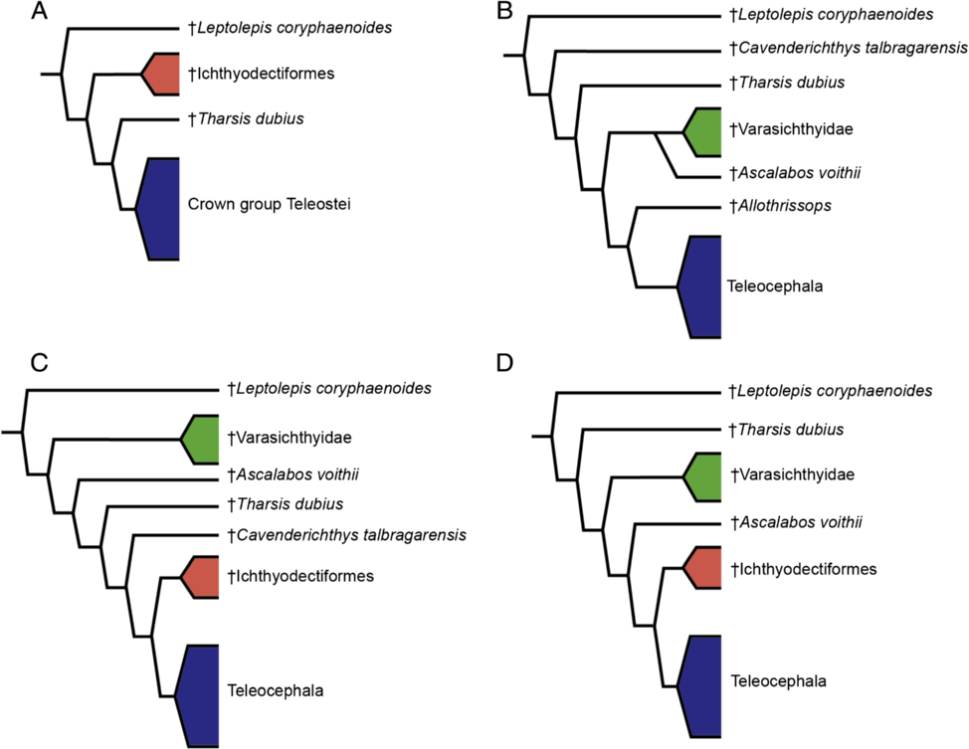
Stability of the node of †*Leptolepis coryphaenoides* and all teleosts more closely related to this taxon than to †*Eurycormus*. **a**. Phylogenetic hypothesis of Patterson [9] and Patterson and Rosen [10]; **b**. Phylogenetic hypothesis of Arratia [13]; **c**. Phylogenetic hypothesis of Arratia [14]; **d**. Phylogenetic hypothesis of Arratia [6, 15]

From their modest origin at the Late Triassic with only a few species represented in Europe, the teleosts underwent a very important radiation during the Jurassic, reaching a cosmopolitan distribution by the end of this period. Despite the large amount of work done on the origin and deep relationships of Teleostei (e.g. [2, 6, 7, 9–15, 17, 18, 20, 46, 55]) basal teleost relationships are not as yet unambiguously resolved, changing drastically according to the character-taxon matrix. Interestingly, however, the node of the most recent ancestor of †*Leptolepis coryphaenoides* and Teleocephala is stable and has been recovered in most phylogenetic studies (see for example [6, 7, 10, 15, 17, 20]; Fig. 8). Even compared to the node uniting all teleocephalans, the node of †*Leptolepis coryphaenoides* and all more crownward teleosts and Teleocephala has the highest support of all teleostean internal nodes in our cladogram (Fig. 2). This node is supported by 16 unambiguous synapomorphies, indicating that there is an important morphologic change at this level of the phylogenetic tree including the occurrence of cycloid scales, first and second hypurals associated with a single ural centrum, a diural caudal skeleton, 19 caudal principal rays, vertebral centra formed by chordacentrum and a basal part of arcocentra surrounded by autocentrum, posttemporal fossa confluent with the fossa Bridgei, tubular nasal bones, and a supraoccipital bone. Moreover, at this node many “holostean-like” characters, like the presence of a prearticular bone in the lower jaw, fringing fulcra in dorsal and anal fins and hypaxial caudal fin rays, disappear from the teleostean lineage. Based on †*Leptolepis coryphaenoides* this node is dated on the Early Jurassic, right before the extraordinary radiation of teleosts during the Late Jurassic, including the origin of the three main lineages of Teleocephala. Thus, it is evident that all these morphological changes currently recorded at the level of †*Leptolepis coryphaenoides* had significant implications for the evolution of Teleostei.

## Conclusions

The analysis of the phylogenetic relationships of †*Luisiella feruglioi* revealed several significant aspects on the early evolution of Teleostei. A stem-based total clade Teleostei is accepted herein and our results emphasize the arbitrariness of selecting a particular node to delimit Teleostei along the stem Teleocephala due to the potential changes of the stem group caused by addition of newly discovered as well as restudied taxa. Actually, there is a general agreement regarding the stem taxa except for the inclusion of the basalmost pycnodontiforms, pachycormiforms, and aspidorhynchiforms. Our study strongly supports the robustness of the node of †*Leptolepis coryphaenoides* and more crownward teleosts and highlights the major morphological changes that appear to have occurred at this level of the phylogeny before the first major radiation of teleosts. Improvements in the knowledge of Triassic and Jurassic teleosts might change our present ideas about the pattern of relationships of early teleosts and show that these morphological changes probably occurred more gradually than currently thought. However, as far as known today it is evident that teleost underwent an important evolutionary process at this level of the phylogeny.

The analysis led us to the recognition of a monophyletic clade of Late Jurassic freshwater fishes from Gondwana. The clade is here named †Luisiellidae fam. nov. and includes so far three taxa: †*Luisiella feruglioi* (Bordas, 1942) [31], †*Cavenderichthys talbragarensis* (Woodward, 1895) [93], and †*Waldmanichthys koonwarri* (Waldman, 1971) [83]. Luisiellids are small basal teleosts, phylogenetically more closely related to †*Tharsis dubius*, the Jurassic †Varasichthyidae, †*Ascalabos voithii* and †Ichthyodectiformes than they are to †*Leptolepis coryphaenoides*. Among the taxa on the stem Teleocephala, luisiellids possess some derived features that are synapomorphies for this clade but have derived independently in Teleocephala and occasionally also in †*Pachythrissops*. However, the overall appearance of luisiellids resembles that of other basal teleosts because of the presence of numerous plesiomorphic traits.

Consequently, taking the hierarchical framework proposed by Wiley and Johnson [34], luisiellids are classified as follow:

- Infraclass Teleostei Müller [36] (sensu De Pinna [33])
- Family †Luisiellidae fam. nov.
- Genus †*Luisiella* Bocchino [81]
- †*Luisiella feruglioi* (Bordas, 1942; [31])
- Genus †*Cavenderichthys* Arratia [14]
- †*Cavenderichthys talbragarensis* (Woodward, 1895; [93])
- Genus †*Waldmanichthys* gen. nov.
- †*Waldmanichthys koonwarri* (Waldman,1971; [83])

According to our cladistic analysis, the Jurassic varasichthyids are not crossognathiforms and the morphological similarity between these two groups is due to parallelism. The †Crossognathiformes sensu Taverne ([88]; i.e. †Crossognathidae and †Pachyrhizodontoidea) are retrieved as a clade with the inclusion of the Late Jurassic †*Bavarichthys* from southern Germany and †*Chongichthys* from Chile. †Crossognathiforms are further confirmed as members of Teleocephala and, although their phylogenetic relationships within this latter clade are uncertain, a possible sister group relationship with Clupeocephala is suggested by the reduced consensus.

## Availability of supporting data

The data sets supporting the results of this article are included within the article and its additional files. The data matrix and complete list of characters are also available in Morphobank under Project P2197 http://morphobank.org/permalink/?P2197 [94].

## Additional files

Additional file 1:
**Discussion of characters.** (DOCX 69 kb) Additional file 2:
**List of studied specimens and consulted literature.** (DOCX 19 kb) Additional file 3:
**List of characters, data matrix, most parsimonious trees and strict consensus tree.** (PDF 389 kb)
