# Supplementary material for: Phylogenetic relationships of †Luisiella feruglioi (Bordas) and the recognition of a new clade of freshwater teleosts from the Jurassic of Gondwana
Source: BMC Evol Biol. 2015 Dec 3;15:268. doi: 10.1186/s12862-015-0551-6 (PMC4668602; doi:10.1186/s12862-015-0551-6)
Supplement: Additional file 1: — Discussion of characters. (DOCX 69 kb) [file 12862_2015_551_MOESM1_ESM.docx]

DISCUSSION OF CHARACTERS

This section includes the complete list of characters used in the cladistic analysis, including brief discussions of those characters that are new or have been modified from previous studies.

Anatomical abbreviations: H1 to H3, hypurals 1 to 3; PH, parhypural; Pu1 to Pu8, preural centrum 1 to 8; U1, “first” ural centrum; U2, “second” ural centrum.

**Braincase and skull roof**

1- Suture between left and right frontal bones: smooth (harmonic suture) (0); serrated (1) ([1]: ch. 19)

2- Anterior margin of frontal: narrower than posterior margin (0); of similar width or slightly wider than posterior margin (1) (modified from ([1]: ch. 3, [2]: ch. 19, [3]: ch. 25, [4]: ch. 188, [5]: ch. 6, [6]: ch. 5, [7]: ch. 179, [8]: ch. 139, [9]: ch. 3, [10]: ch. 175).

In the basal teleosts †*Dorsetichthys bechei*, †*Leptolepis coryphaenoides* and varasichthyids, among others [4],[10], as well as in some teleocephalan taxa like the elopomorphs †*Anaethalion* *knorri* and *Elops* [11], the posterior margins of the frontal bones are distinctly wider than their anterior margins, which are relatively very narrow. This is also the condition present in †*Luisiella feruglioi*. In contrast, the frontal bones of pachycormiforms [2],[12], osteoglossomorphs [3],[9] as well as those of some ostariophysan groups (e.g. Gonorynchiformes, Siluriformes, Characiformes; [4],[13]) have anterior and posterior margins of similar widths (e.g. *Hiodon*, *Brycon*) or even anterior margins slightly wider than posterior margins (e.g. *Heterotis*).

The definition of this character was modified from previous authors to represent the variability observed in our taxonomic sample as accurately as possible. Li and Wilson [3] scored three character states of which only the state (0), i.e. the occurrence of an anterior portion of the frontal narrower or slightly broader than the posterior end of the bone, is represented in our sample. Arratia considered the variation in the shape of the frontal bones in several analyses [1],[4],[7],[8],[10]. However, the different characters that were used to represent this variation either do not reflect the different morphologies in our data set or imply a mixture of non-homologous features (e.g. width and length, frontal width compared with the fusion of the frontals to other cranial bones). In her most recent cladistic analysis, a character representing the variation in the shape of the skull roof as a whole is proposed [1]. According to our observations, however, the shape of the skull roof is mainly determined by the shape of the frontal bones and, thus, Arratia’s [1] character would be equivalent to our character 2. Arratia’s definition might be preferable only when the frontals are fused in the studied taxa, but this is not the case in the present analysis. Finally, Hilton [9] proposed this character in his phylogeny of Osteoglossomorpha, although he included a new state distinguishing the cases in which the anterior margin of the frontal is about equal in width to its posterior margin from those where the anterior margin is wider. This distinction is not obvious in our taxonomic sample and therefore we have not used this coding.

3- Shape of nasal bones: flat, approximately rectangular (0); flat, triangular (1); tubular but straight (2); tubular and strongly curved (3) (modified from [3]: ch. 12, [6]: ch. 2, [9]: ch. 7)

According to our observations, in most teleosts the nasal bones are straight, tubular bones, almost reduced to ossifications around the supraorbital sensory canals, such as those observed in †*Luisiella feruglioi*. However, these bones are not tubular but flat, triangular plates in the basal teleost †*Siemensichthys* *macrocephalus*, whereas they are broad, approximately rectangular plate-like ossifications in the basal teleosts †*Pholidophorus* *gervasutti* and †*Dorsetichthys* *bechei* [1],[8],[14] as well as in some teleocephalan taxa (e.g. *Heterotis*, †*Humbertia,* *Denticeps*, *Thymallus*). Notably, the nasals are tubular and strongly curved in the living osteoglossomorph *Hiodon* [3],[15]. Among the outgroup taxa, the nasals are broad and rectangular in *Amia* [14] and variably broad, but approximately rectangular and not tubular in lepisosteiforms, pachycormids, and aspidorhynchiforms [12],[16],[17]. This character is not applicable to pycnodontiforms because they lack nasal bones (e.g. †*Mesturus*; [18]).

Li and Wilson [3],[6] and Hilton [9] proposed this character for their phylogenies of Osteoglossomorpha. Our character states (2) and (3) are identical to their character states 0 and 1. We have modified their character state 2 as well as Hilton’s state 3 (nasals gutter-like or irregularly subrectangular; nasals flat and broad) to discriminate between approximately rectangular and approximately triangular nasals, which are clearly different character states in our taxonomic sample ((0) and (1), respectively).

4- Relationship between left and right nasal bones: in contact along the midline (0); separated (1) (modified from [1]: ch. 20, [6] ch. 3, [7]: ch. 190, [8]: ch. 138)

Broad nasals that are in contact along the midline is the most generalized condition observed among basal neopterygians [19]. In contrast, most teleosts have the nasals located lateral to the most anterior portion of the frontals and/or the ossifications of the ethmoidal region (e.g. mesethmoid, supraethmoid, [8],[20], as observed in basal teleosts, such as †*Pholidophorus gervasuttii*, †*Siemensichthys* *macrocephalus*, and †*Dorsetichthys* *bechei* [1], as well as in most teleocephalans. This is also the condition present in †*Luisiella feruglioi*. As an exception among the teleocephalan taxa included in the present analysis, the nasal bones are in contact at the midline in the osteoglossomorph *Heterotis* [3],[6],[9]. This condition occurs frequently in basal neopterygians but not in teleosts and was considered by Li and Wilson [6] and posteriorly by Hilton [9] as secondarily derived in osteoglossomorphs.

In amiids and lepisosteiforms the nasals join at the midline [14],[17] but in aspidorhynchiforms these bones are separated by the frontals and the large rostral ossification [16],[21] whereas in pachycormiforms they are mainly separated by a rostrodermethmoid ossification [12].

These different conditions of the nasal bones have been represented in binary characters several times by scoring the presence/absence of nasals separated by the rostrodermethmoid [7], the presence/absence of nasals separated by the frontals [8], or nasals in contact along the midline versus nasals separated by the frontals [1]. Li and Wilson [5] used a similar character distinguishing three character states: nasal bones in contact at the midline, separated by the frontals, and separated by the mesethmoid. We do not agree with this character because we consider that the separation of the nasals is independent from the bone or bones that occupy the area left between the separated nasals in the different taxa.

5- Supraoccipital bone: absent (0); present (1) ([1]: ch. 13, [4]: ch. 5, [10]: ch. 4, [22]: ch. 4)

6- Supraoccipital crest: absent or poorly developed (0); well-developed, spine-like supraoccipital crest (1); well-developed, high and triangular supraoccipital crest (2); hypertrophied, forming a pectinate blade (3)(modified from [5]: ch. 13, [21]: ch. 6, [23]: ch.9, [24]: ch. 2, [25]: ch. 3, [26]: ch. 2)

The skulls of most basal teleosts, such as †*Leptolepis* *coryphaenoides* [27], varasichthyids [10], †*Ascalabos*, and †*Cavenderichthys* *talbragarensis* [20],[28] have poorly developed supraoccipital crests. The same condition is present in †*Luisiella feruglioi* and in certain teleocephalan groups, like clupeomorphs [21],[29], elopomorphs [11],[20],[30], euteleosts, and most osteoglossomorph taxa (except for *Hiodon* in this analysis, see below). In contrast, a well-developed spine-like supraoccipital crest is present in most ostariophysans (e.g. *Brycon*, [13]) as well as in the Cretaceous teleosts †*Notelops* and †*Rhacolepis* [21],[30]. A high and triangular supraoccipital crest has appeared independently in *Hiodon* [15] and the †Ichthyodectoidei, which are derived ichthyodectiforms (e.g. †*Thrissops,* †*Cladocyclus*), being absent in other ichthyodectiform taxa, like †*Allothrissops* [23],[31]. The supraoccipital crest is hypertrophied in the gonorynchiforms *Chanos* and †*Tharrhias*, in which it projects caudally forming a posteriorly pectinate blade [5].

This character was used by Maisey [21] in his phylogenetic analysis of ichthyodectiforms, where he considered the presence/absence of a high, triangular supraoccipital crest (our state (2)). This author also included an additional character that refers to the size of the supraoccipital crest (character 17), but we think that this last character is not independent from the previous one. The occurrence of both a large and triangular supraoccipital crest and a small crest was coded by Stewart [25]. Alvarado-Ortega [23] considered four binary presence/absence characters dealing with the shape and size of the supraoccipital crest (i.e. ch. 9, 33, 35 and 47) including the two proposed by Maisey, but we consider these characters are not independent from each other. On the other hand, our character definition is similar to character 13 of Grande and Poyato-Ariza [5] although the latter included only three states (essentially our states (1), (2) and (3)). The character proposed by Grande and Poyato-Ariza was also modified by Cavin [24], who proposed a binary character for the occurrence of a small versus a large supraoccipital crest, projecting dorsally and/or posteriorly above the occipital region.

7- Condition of skull roof: medioparietal (0); lateroparietal (1) ([10]: ch. 194, [22]: ch. 183). The terminology was modified according to the anatomical nomenclature used in this study.

8- Relative shape of the extrascapular bone(s): quadrangular or semicircular not expanded caudally (0), tubular (1), approximately triangular, expanded caudally (2), expanded rostrally (3) (modified from [3]: ch. 27, [6]: ch. 9, [9]: ch. 2, [10]: ch. 189, [22]: ch. 178)

According to our observations, most basal teleosts have relatively small, quadrangular or semicircular extrascapular bones, which are not expanded caudally or rostrally (e.g. †*Pholidophorus* *gervasutti*, †*Eurycormus* *speciosus*, †*Dorsetichthys* *bechei*, †*Tharsis* *dubius*, †*Ascalabos* *voithii*; [1],[20],[32]). This is also the condition present in †*Luisiella feruglioi*. In particular, large, approximately triangular and caudally expanded extrascapulars occur in the Jurassic varasichthyids (e.g. †*Varasichthys*, †*Protoclupea*; [10].[33]) as well as in the Jurassic †*Bavarichthys* and the Cretaceous teleosts †*Rhacolepis*, †*Goulmimichthys*, †*Crossognathus*, and †*Apsopelix* [10],[24],[30],[34]. In contrast, the extrascapular is reduced to a small tubular bone in most teleocephalans (e.g. *Thymallus*, *Heterotis*, *Chanos*), with the exception of some elopomorphs (e.g. †*Anaethalion*, *Elops*, *Megalops*; [11],[35]) that have small semicircular extrascapulars, and some osteoglossomorphs (e.g. *Hiodon* in our analysis) which have large, rostrally expanded extrascapular bones that partially overlap the parietals and the posterior portion of the frontals [9],[15].

This character was modified from character 27 of Li and Wilson [3] and character 9 of Li and Wilson [6] coding the presence of an expanded quadrangular or triangular extrascapular bone versus the slender and distinctly angular or branched extrascapular bone of the extinct osteoglossomorph †*Phaerodus*, not included in our analysis. Hilton [9] noted that the state 1 of Li and Wilson [3],[6] included more than one possible condition for the shape of the extrascapular bone and, thus, discriminated between an expanded extrascapular bone, a reduced and irregularly shaped extrascapular bone, and a reduced and tubular extrascapular bone. Our observations indicate that there are two distinct conditions of expanded extrascapular bones in our sample (i.e. rostrally and caudally expanded) and that in most basal teleosts and elopomorphs lacking an expanded extrascapular the shape of this bone is not irregular but quadrangular or semicircular.

On the other hand, Arratia [10] and Arratia and Tischlinger [22] coded our character state (2) as a presence/absence character with an unspecified absence, relating the caudal expansion of the extrascapular to the posterior border of the opercle. We have chosen not to relate the shape of the extrascapular to the position of the opercle as we were not able to find any developmental or functional relationship between the extension of the former and the posterior margin of the latter; other taxa might have (not observed in this study) expanded extrascapulars but the position of the opercle is different from that of our sampled taxa.

9- *Recessus lateralis*: absent (0); present (1) ([4]: ch. 21, [6]: ch. 11, [7]: ch. 17, [10]: ch. 17, [20]: ch. 16, [22]: ch. 16, [29]: ch. 9; [36]: ch. 8)

10- Ossified bulla in the prootic bone: absent (0), present (1) (modified from [4]: ch. 22, [6]: ch. 80, [7]: ch. 18, [10]: ch. 18, [20]: ch. 17, [22]: ch. 17, [29]: ch. 2, [36]: ch. 9)

This character refers to a trait that has only been observed within the teleocephalan Clupeomorpha. According to Grande [29] two ossified bullae develop in the prootic and pterotic bones of clupeomorphs, respectively, as observed for example in the living *Engraulis* and *Denticeps* and in the fossil †*Santanaclupea* [29],[37]. However, in some clupeomorphs the pterotic bone does not form a well-developed bulla, and this structure is only present in the prootic bone (e.g. †*Diplomystus* in this analysis). The condition is unknown in most basal teleosts and fossil teleocephalans, including †*Luisiella feruglioi*. Extant elopomorphs, osteoglossomorphs, ostariophysans, and euteleosts lack an ossified bulla in the prootic [29],[31],[38], with the only exception of †*Erichalcis*, which is considered a clupeomorph by Forey [39] and Grande [29], but a euteleost by Arratia [4]. According to Greenwood et al. [38], Patterson and Rosen [31], and Grande [29], the occurrence of a bulla is related to the presence of an otophysic connection involving a diverticulum of the swimbladder that penetrates the exoccipital and extends into the prootic within the lateral wall of the braincase. The otophysic connections (i.e. between the swimbladder and the inner ear) are achieved through the development of two large vesicles at each side of the braincase, which originate from the bifurcation of the left and right swimbladder diverticula and are lodged in the bullae [38].

All cited authors scored the presence of an otophysic connection for this character. However, since to date no fossil has preserved the swimbladder diverticula, which are the organs actually involved in the otophysic connection, we prefer scoring the presence or absence of ossified bullae, which is the attribute that can be assessed in fossil specimens.

11- Posttemporal *fossa*: absent (0); present and separated from the *fossa Bridgei* (1); present and confluent with the *fossa Bridgei* (2) (modified from [16]: chs. 5 and 6, [31]: ch. 5, [40]: ch. 20).

The posttemporal *fossa* is a depression located in the occiput of most neopterygians. In these fishes, the *fossa* is open laterally and houses the anterior extension of the trunk musculature [19]. A similar *fossa* is present in some basal actinopterygians where it opens laterally (e.g. *Acipenser,* †*Saurichthys*) but does not house musculature. On the other hand, the *fossa Bridgei* is an internal depression located in the otic region of the skull and related to the posterior semicircular canal. In aspidorhynchiforms, Triassic and Lower Jurassic “pholidophoriforms”, as well as in †*Dorsetichthys* *bechei* the *fossa Bridgei* and the posttemporal *fossa* are separated from each other by a thin wall of bone, whereas in *Amia,* †*Pachycormus*, and most teleosts the two *fossae* are confluent. In the latter two cases, the anterior portion of the trunk musculature extends into the otic region of the skull occupying both posttemporal *fossa* and *fossa Bridgei* [40],[41]. In contrast, in *Amia* the axial muscles extend anteriorly occupying only the posttemporal *fossa*.

A posttemporal *fossa* is absent in *Lepisosteus* [17] and in clupeid clupeomorphs [29],[38]. Several authors have largely debated its occurrence in osteoglossomorphs and the issue remains unsolved. Ridewood [42], Greenwood [43], and later Li and Wilson [3] considered that the temporal fenestra of osteoglossomorphs might be homologous to the preepiotic *fossa* of clupeoids, and that a posttemporal *fossa* would be absent in these groups of fishes. On the other hand, Taverne [44],[45] used both the posttemporal and temporal fossae as synonyms in his descriptions of osteoglossomorph fishes. In addition, the author proposed that the temporal fenestra of osteoglossomorphs resulted from a transformation of the posttemporal *fossa* of elopids and basal teleosts, mainly based on the presence of the epiotic, pterotic, and exoccipital bones participating in both fossae in basal teleosts and in notopterids and mormyroids among osteoglossomorph taxa. However, and according to their hypothesis of phylogenetic relationships within osteoglossomorphs, Cavin and Forey [46] and Hilton [9],[15] discarded Taverne’s proposal by arguing that the presence of a temporal fenestra framed by those bones is a derived condition for Osteoglossomorpha and that it corresponds to a different depression. Considering these arguments and the lack of consensus, this character has been coded as (?) for the three osteoglossomorph taxa included in this analysis.

This character was proposed by Patterson [40] and Patterson and Rosen [31], who considered our states (1) and (2) only. Patterson [40] also distinguished the invasion of the trunk musculature in both posttemporal fossa and *fossa Bridgei* from the particular condition of *Amia*, in which these fossae are confluent but only the epaxial musculature occupies the posttemporal *fossa*. In the same derived state this author included the case where both fossae are separated and the case of *Amia*. We did not follow this argument because whether the trunk musculature occupied either fossae or not in fossil teleosts cannot be determined unambiguously.

12- Bones participating in the posttemporal fossa: epiotic, pterotic and exoccipital (0); epiotic, pterotic, exoccipital and intercalar (1); epiotic, pterotic and parietal (2) (modified from [10]: ch. 188, [22]: ch. 177)

The posttemporal fossa may be framed by different bones in different groups of teleosts or even within a same group of teleosts, as it occurs among euteleosts. This fossa is framed by the epiotic, pterotic and exoccipital bones in most basal teleosts like †*Siemensichthys* *macrocephalus*, †*Dorsetichthys* *bechei*, †*Leptolepis* *coryphaenoides* [41], and in elopomorphs [47]. In particular, the epiotic, pterotic, exoccipital, and intercalar participate in the formation of a roofed posttemporal fossa in the Jurassic and Cretaceous teleosts †*Notelops*, †*Rhacolepis*, †*Goulmimichthys*, †*Apsopelix*, and †*Luisichthys* [10],[24],[30]. In contrast, in the euteleost and ostariophysan taxa included in this analysis the posttemporal fossa is framed by the epiotic, pterotic, and parietal bones (e.g. *Thymallus,* *Oncorhynchus,* †*Humbertia, Chanos, Opsariichthys*; [48]-[50]). The condition is unknown in †*Luisiella feruglioi*, aspidorhynchiforms, and pachycormiforms*.* Finally*, Amia* has a posttemporal fossa framed by the epiotic and dermopterotic [14]. Although the later is homologous (in part) with the pterotic of teleosts (which results from the fusion of dermopterotic and autopterotic bones) we are not certain whether the region of the pterotic, which frames the posttemporal fossa in teleosts, corresponds to the dermopterotic portion of the bone and, therefore, the character is coded as unknown for *Amia*.

This character has been modified from that used by Arratia [10] and Arratia and Tischlinger [22] because these analyses only scored the presence/absence of our state (1), remaining the absence state unspecified.

13- Position of the foramen for the glossopharyngeal nerve: in the prootic bone (0); in the exoccipital bone (1); in the basioccipital bone (2) (modified from [1]: ch. 32, [4]: ch. 24, [7]: ch. 20, [8]: ch.15, [10]: ch. 21, [20]: ch. 20, [22]: ch. 19, [33]: ch. 8, [36]: ch. 7, [51]: ch. 62)

The foramen for the glossopharyngeal nerve (IX) pierces the prootic bone in the early Mesozoic teleosts such as †*Siemensichthys* *macrocephalus*, †*Dorsetichthys* *bechei*, and †*Leptolepis* *coryphaenoides* and was considered the plesiomorphic condition for Teleostei [4]*.* In more derived teleosts, like †*Tharsis dubius* and teleocephalans*,* the foramen for the exit of the glossopharyngeal nerve is located in the exoccipital [31]. As an exception, in the clupeomorph †*Santanaclupea* the foramina for both this nerve and the vagus nerve open in the basioccipital [37]. The condition in †*Luisiella feruglioi* is unknown. The foramen for the glossopharyngeal nerve is located in the prootic bone in aspidorhynchiforms, as in other basal teleosts, whereas in *Amia* the glossopharyngeal nerve exits the braincase through a cartilaginous bulla, placed posterior to the prootic [14],[31].

This character was modified from previous authors. Arratia [1],[4],[7],[8],[10],[20],[33],[36] and Arratia and Tischlinger [22] originally coded the presence/absence of the foramen for the glossopharyngeal nerve in the exoccipital rather than in the prootic bone. Likewise, Taverne [51] considered only our states (0) and (1).

14- Position of the foramen for the vagus nerve: between the intercalar and exoccipital bones (0); in exoccipital alone (1); in the basioccipital (2); between the intercalar and basioccipital bones (3) (modified from [1]: ch. 33, [4]: ch. 25, [7]: ch. 21, [8]: ch. 16, [10]: ch. 21, [22]: ch. 20)

In general, there are two locations for the exit of the vagus nerve (X) in the braincase of teleosts. In basal teleosts (e.g. †*Dorsetichthys* *bechei*, †*Leptolepis* *coryphaenoides*, †*Tharsis dubius*, aspidorhynchiforms, and pachycormiforms) the foramen for this nerve occurs between the intercalar and exoccipital bones [41], as in the living halecomorph *Amia* and in the living gar *Lepisosteus* [17]. On the other hand, in most teleosts (with the exception of †*Santanaclupea*, see below) the foramen for the vagus nerve pierces the posterolateral face of the exoccipital [31]. In the clupeomorph †*Santanaclupea* the foramen for nerve X is located in the basioccipital bone [37]. As an exception among basal teleosts, the aspidorhynchiform †*Vinctifer* shows an alternative position of this foramen between the intercalar and basioccipital bones [52]. Like in many other fossil taxa, the condition in †*Luisiella feruglioi* is unknown due to incomplete preservation.

Arratia [1],[4],[7],[8],[10] and Arratia and Tischlinger [22] only scored our state (1) as an independent character and thus, more than one alternative position for the vagus nerve is included in the state ‘absence’.

15- Foramina in basioccipital for occipital or spinal arteries: present (0), absent (1) ([53]: ch. 17)

16- Trajectory of the dorsal aorta in relation to the basioccipital: passing through an ossified canal formed by the basioccipital (0); passing through a median groove in the basioccipital (1); canal or groove in the surface of the basioccipital bone absent (2) (modified from [1]: ch. 28, [4]: ch. 14, [7]: ch. 12, [8]: ch. 10, [10]: ch. 12, [20]: ch. 12, [22]: ch. 12, [31]: ch. 27, [33]: ch. 3, [36]: ch. 2, [51]: ch. 49, [53]: ch. 16)

In fishes, the dorsal aorta carries the oxygenated blood from the branchial apparatus to the rest of the body. According to Patterson [41] in many basal actinopterygians (e.g. *Polypterus*) and in the basal teleosts †*Dorsetichthys bechei* and †*Leptolepis* *coryphaenoides* there is a completely closed, ossified canal for the passage of the dorsal aorta that is formed by ventral projections of the basioccipital bone. In contrast, in the basal teleosts †*Siemensichthys* *macrocephalus* and †*Pachycormus* this artery passes through a distinct median groove excavated in the ventral surface of the basioccipital. On the other hand, there is no aortic canal or evidence of a groove for the median dorsal aorta in the ventral surface of the basioccipital of *Amia* and the basal teleosts †*Tharsis dubius,* †*Varasichthys*, †*Thrissops*, and teleocephalans (except for *Hiodon*, see below). According to several authors (e.g. [41],[54],[55]) in most teleosts the dorsal aorta bifurcates in two lateral aortae before entering the braincase. The backward migration of the bifurcation of the median dorsal aorta is probably related to the enlargement of the *circulus cephalicus* (or lengthening of the lateral aortae) that characterizes teleostean fishes [54],[56] and might have resulted in the shortening or obliteration of the aortic canal in this group [55]. According to Allis [54] and Hilton [15], the basioccipital of the basal osteoglossomorph *Hiodon* has a distinct aortic groove along its ventral surface. The condition of †*Luisiella feruglioi* is unknown.

This character was modified from several previous studies [4],[7],[8],[10],[20],[22],[31],[33],[36],[51], because in all cases it was enunciated as a binary character where the presence/absence of an ossified aortic canal was coded, not describing the alternative conditions represented in our taxonomic sample. On the other hand, a similar character was used in the morphological analysis performed by Hurley et al. [53] to investigate the phylogeny of actinopterygians. This character included four possible states, three of which agree with our proposed states ((0), (1) and (2)), the additional state making reference to the condition of *Acipenser* (not included in the present analysis) where a parabasal canal for the dorsal aorta occurs between the parasphenoid and basioccipital.

17- Posterior myodome: extending in basioccipital (0), not extending into basioccipital because it is confined to the prootics (1), absent (2) ([7]: ch. 170, [8]: ch. 124, [10]: ch. 173, [16]: ch. 4, [22]: ch. 165)

18- Ethmopalatine ossifications: absent (0); present (1) ([4]: ch. 1, [7]: ch. 1, [20]: ch. 1, [22]: ch. 1)

19- Proethmoid ossifications: absent (0), present (1) (modified from [4]: ch. 2, [7]: ch. 2, [10]: ch. 2, [20]: ch. 2, [22]: ch. 2)

Proethmoids are paired, elongate ossifications that roof the anterior portion of the ethmoidal region of members of the euteleost families Esocidae, Cyprinidae, and Umbridae [20],[57]. In both *Esox* and *Umbra*, included in this analysis, the proethmoids slightly overlap the anterior portion of the frontals. Particularly in umbrids, the anterior portion of each proethmoid curves ventrolaterally articulating with the premaxilla whereas posteriorly the bone is ventrally grooved and caps the rostral cartilage as well as it articulates with the anteromedial end of the palatine [58]. A proethmoid ossification is absent in †*Luisiella feruglioi*.

Previous authors [4|,[7],[10],[20],[22] used a character to code the presence of proethmoid ossifications in euteleost taxa, considering the occurrence of two paired endoskeletal ethmoidal ossifications. According to Patterson and Rosen [31], two paired endoskeletal ossifications occur in ichthyodectiforms (i.e. lateral ethmoids and ethmopalatine bones) and also in some euteleots, like esocoids and cyprinoids (i.e. lateral ethmoids and proethmoid ossifications). Even though the authors suggested that ethmopalatine bones and proethmoid bones might be homologous, they clearly assessed their different morphology and topography and therefore, they regard them as unique specializations of each group. The homology of these ossifications was also discarded by Arratia [20], who argued that according to their phylogenetic position both groups belong to different teleost linages. Therefore, we have scored the occurrence of proethmoid and ethmopalatine ossifications independently in this and the previous character, respectively.

20- Independent dermopalatine(s) bone(s): present (0); absent (1)([36]: ch. 16)

21- Autopalatine bone: ossifies late in ontogeny (0); ossifies early in ontogeny (1); autopalatine bone absent (2)([8]: ch. 24)

22- Vomer: paired (0); unpaired (1)([1]: ch. 24, [4]: ch. 187, [7]: ch. 178, [8]: ch. 152, [10]: ch. 174, [22]: ch. 166, [40]: ch. 8)

23- Basisphenoid: present (0); absent (1) ([1]: ch. 11, [4]: ch. 7, [7]: ch. 5, [8]: ch. 3, [9]: ch. 13, [10]: ch. 5, [20]: ch. 5, [22]: ch. 5, [33]: ch. 6, [36]: ch. Ch. 5, [50]: ch. 12)

24- Orbitosphenoid bone: reduced or absent (0), present and large (1) ([22]: ch. 8)

25- Teeth on parasphenoid: absent (0); present and small (1), present and large (2) ([1]: ch. 10, [4]: ch. 12, [7]: ch. 10, [10]: ch. 10, [22]: ch. 10)

26- Posterior extent of parasphenoid: reaching the anterior margin of the basioccipital (0); almost reaching the posterior margin of the basioccipital (1); extending posterior to basioccipital (2) ([10]: ch. 11)

27- Basipterygoid process of parasphenoid: present (0); absent (1) ([9]: ch. 9, [24]: ch. 9, [59]: ch. 65)

28- Separation between olfactory organ and eye: narrow (0); broad (1) ([10]: ch. 152, [22]: ch. 146)

**Circumorbital bones**

29- Shape of the most anterior infraorbital bone: laminar or plate-like (0); tubular (1) (modified from [1]: ch. 41, [4]: ch. 33, [7]: ch. 29, [10]: ch. 29, [14]: ch. 58, [20]: ch. 27, [22]: ch. 28)

In most taxa, the first infraorbital bone (also known as lachrymal) is an expanded plate-like bone that carries the most anterior portion of the infraorbital sensory canal. A first infraorbital bone with these characteristics occurs in †*Luisiella feruglioi*, as well as in the basal teleosts (e.g. †*Eurycormus speciosus,* †*Tharsis dubius,* †*Cavenderichthys talbragarensis* [27],[28]), and in most ostariophysans, elopomorphs, and clupeomorphs. In contrast, osteoglossomorphs (except for *Heterotis*, in this analysis; [9]) have an elongated and not expanded first infraorbital bone that is practically reduced to a tube around the infraorbital canal [9],[20].

Among the outgroup taxa the rostralmost infraorbital bone is laminar or plate-like. In *Lepisosteus* and †*Obaichthys* the anterior portion of the series of infraorbital bones is composed of numerous toothed plate-like infraorbital ossifications, known as toothed infraorbitals [60],[61], which are also pierced by the infraorbital sensory canal. Although these toothed ossifications might be serially homologous with the infraorbital bones of other neopterygians, they represent a unique trait of lepisosteids and obaichthyids [61].

30- Fourth and fifth infraorbital bones: separate (0), fused (1) ([3]: ch. 11, [4]: ch. 34, [6]: ch. 19, [7]: ch. 30, [10]: ch. 30, [22]: ch. 29, [24]: ch. 20)

31- Circumorbital ring: incompletely closed (0); completely closed ring, no space left between bones (1) ([10]: ch. 190, [22]: ch. 179)

32- Number of suborbital bone(s): numerous (0); two or three (1); one (2); none (3) ([1]: ch. 47, [10]: ch. 31, [22]: ch. 30)

33- Number of supraorbital bone(s): two or more (0), one (1), none (2) ([1]: ch. 49)

34- Shape of the supraorbital bone (or anterior supraorbital bone): not expanded (0); expanded anteroventrally (1) (modified from [4]: ch. 37, [7]: ch. 33, [10]: ch. 33, [20]: ch. 29, [22]: ch. 32)

In general, the supraorbital bone (or the anterior supraorbital bone in taxa with more than one supraorbital ossification), is ovoid in shape and slightly tapered anteriorly (e.g. †*Leptolepis* *koonwarri*, [62]; †*Leptolepis* *coryphaenoides*, [27]; †*Cavenderichthys* *talbragarensis*, [28], pers.obs.; †*Luisiella* *feruglioi*). In contrast, the supraorbital is anteroventrally expanded in the Jurassic euteleosts †*Leptolepides* and †*Orthogonikleithrus*, a condition that was proposed as a synapomorphy of Orthogonikleithridae by Arratia [20].

A similar character making reference to the occurrence of a large supraorbital bone with an expanded anteroventral portion was used in previous phylogenetic analyses (i.e. [4],[7],[10],[20],[22]). We have modified the character enunciation in order to avoid unspecified absences, and also discarded any reference to the relative size of the supraorbital bone (e.g. large), which we consider an independent feature.

35- Dermosphenotic: small (0); large, well-developed bone (1) ([10]: ch. 191, [22]: ch. 180)

36- Independent antorbital bone: present (0); absent (1) ([1]: ch. 38, [10]: ch. 192, [22]: ch. 181, [24]: ch. 23)

37- Antorbital bone: carrying a portion of the infraorbital canal (0); without sensory canal (1) ([1]: ch. 39, [10]: ch. 24, [22]: ch. 23)

38- Shape of antorbital bone: approximately triangular (0); rectangular (1); tubular and comma-shaped (2) (modified from [4]: ch. 38, [7]: ch. 34, [10]: ch. 34, [20]: ch. 30, [22]: ch. 33)

Most basal teleosts (e.g. †*Tharsis* *dubius*, †*Ascalabos* *voithii*), elopomorphs, and ostariophysans have a small, approximately triangular antorbital bone, which is dorsally expanded. This is also the condition present in †*Luisiella* *feruglioi*. As an exception, the antorbital is rectangular in the basal teleost †*Eurycormus* *speciosus* and also in the pachycormiform taxa included in the outgroup [12],[32]. In contrast, the antorbital bone is not triangular but a tubular, comma-shaped ossification in most clupeomorphs (except for basal taxa like †*Diplomystus*, *Denticeps*, and *Engraulis*; [29]) as well as in some euteleost fishes included in this analysis, like †*Leptolepides* *sprattiformis* and †*Erichalcis* [20],[31],[63]. *Amia* and *Lepisosteus* also have comma-shaped antorbital bones.

A binary character coding the presence/absence of a comma-shaped antorbital bone was used in several previous phylogenetic analyses [4],[7],[10],[20],[22]. This character has been modified in order to represent the observed variation in the shape of the antorbital bone.

39- Posterior infraorbital bones: small and not overlapping or slightly overlapping the anterior margin of preopercle (0); expanded posterior infraorbitals overlapping the anterior margin of preopercle (1) ([10]: ch. 193, [22]: ch. 182)

**Palatoquadrate, Hyoid Arch, and Urohyal**

40- Hyomandibular bone with a preopercular process at its posterior margin: absent (0); present (1) ([1]: ch. 77, [4]: ch. 44, [7]: ch. 40, [8]: ch. 26, [10]: ch. 39, [20]: ch. 36, [22]: ch. 38)

41- Elongation of suspensorium: absent (0), present, due to a lengthening of the symplectic (1), present, due to the occurrence of a cartilage between symplectic and hyomandibula (2), present, due to the ventroposterior inclination of the hyomandibula (3), present, due to the occurrence of the quadratojugal separating quadrate and sympletic (4) (modified from [1]: ch. 76, [4]: chs. 41, 42 and 43, [7]: chs. 37, 38 and 39, [8]: ch. 25, [10]: chs. 37 and 38, [20]: chs. 33, 34 and 35, [22]: chs. 36 and 37)

In most teleost taxa, including †*Luisiella* *feruglioi*, the hyomandibula is vertically or subvertically placed in the skull and articulates anteriorly with the symplectic, which in turn articulates with the quadrate. In these fishes, the suspensorium is vertically oriented and not inclined and both symplectic and quadrate are close to the hyomandibular bone. This generalized condition has been termed by Arratia [1],[4],[7],[8],[10],[20] and Arratia and Tischlinger [22] normal or not elongated. In contrast, a variation from the normal condition of the suspensorium occurs in some teleocephalans, *Lepisosteus*, and †*Obaichthys*, where the suspensorium results elongated as explained below. In ostariophysans (e.g. *Chanos*, *Brycon*, †*Gordichthys*; [49]) the elongation is due to the separation of the quadrate and hyomandibular bones by a lengthening of the symplectic. In some extinct euteleosts (e.g. †*Leptolepides*, †*Orthogonikleithrus*; [20]) the elongation of the suspensorium is due to the occurrence of a cartilage (remanent of the hyosimplectic cartilage), which is placed between the hyomandibular bone and the symplectic. In some taxa, like the clupeomorphs †*Santanaclupea* and *Engraulis*, an elongation of the suspensorium is caused by a different inclination of the hyomandibula, which is not vertically positioned but inclined in an anterodorsal to posteroventral direction [20],[37]. In *Lepisosteus* and †*Obaichthys* the quadrate is separated from the symplectic by the quadratojugal and besides, the symplectic is not in contact with the hyomandibular bone but with the preopercular bone, which in turn contacts the hyomandibular [17],[60].

Arratia [4],[7],[20] proposed three different characters (see above) to describe the different conditions that may cause an elongation of the suspensorium in teleosts (our states (0), (1), (2) and (3)). Subsequently [10],[22], the same information was considered in two different characters, where the elongation of the suspensorium due to the ventroposterior inclination of the hyomandibula was coded in a separate binary presence/absence character. In addition, only this latter condition was taken into account in the phylogenetic analyses performed by Arratia [1],[8]. We have included all the different configurations of the suspensorium found in teleosts in a single character also adding a character state to describe the condition present in gars (our state (4)).

42- Hyomandibular articulation with the neurocranium: by means of one articular surface (0), by means of two distinct articular surfaces (1) (modified from [3]: ch. 18, [5]: ch. 41, [6]: ch. 50, [9]: ch. 28)
 Most basal teleosts have a single-headed dorsal portion of the hyomandibular bone articulating with the neurocranium. This is the condition present in †*Luisiella* *feruglioi* as well as in *Amia* and *Lepisosteus*. However, in the majority of extinct and living teleocephalans a double-headed hyomandibular bone articulates with the neurocranium. In these taxa, the hyomandibular bone bears one anterior and one posteriorly directed processes or heads with articular facets, which in a few species (e.g. some osteoglossomorphs) may be also joined by a thin bony bridge. This is the case of *Hiodon*, for example, where there is a bony bridge between the two heads without being in contact with the neurocranium thereby remaining two distinct articular surfaces [9].

Li and Wilson [3],[6] and Hilton [9] used a similar character coding the presence of a single-headed or a double-headed hyomandibular, although they distinguished between the cases in which the two heads are separated from each other and those in which they are connected (bridged double-headed). Grande and Poyato-Ariza [5] considered a different character with two states based on the relative position of the two hyomandibular heads. The authors distinguished a two-headed hyomandibular bone with two dorsal facets articulating with the neurocranium from a two-headed hyomandibular bone with anterior and dorsal articular surfaces, where only the posterior head articulates with the neurocranium. Even though the first condition described by Grande and Poyato-Ariza [5] corresponds to our state (1), the other condition is not represented in our taxonomic sample and therefore, was not included in our character coding.

43- Elongate posteroventral process of quadrate: absent (0); present (1) ([1]: ch. 78, [4]: ch. 59, [7]: ch. 55, [8]: ch. 34, [10]: ch. 54, [16]: ch. 31, [22]: ch. 53)

44- Shape of the dorsal margin of the quadrate: convex (0); straight (1); concave (2); sinuous or sigmoid (3), notched (4) (modified from [23]: ch. 57, [25]: ch. 17)

In general, the teleost quadrate can be easily described as formed by a fan-shaped main body and an elongated posteroventral process. However, the shape of the dorsal margin of the main body of the quadrate varies within Teleostei. A quadrate with a convex (sometimes slightly convex) dorsal margin occurs in some basal teleosts, like †*Dorsetichthys* *bechei*, †*Siemensichthys siemensi*, †*Cavenderichthys* *talbragarensis*, †*Ascalabos* [8],[20], teleocephalans, †*Luisiella feruglioi*, and most of the outgroup taxa. A straight dorsal margin of the quadrate is present in †*Apsopelix*, †*Domeykos*, and some elopiforms like *Megalops* in this analysis [10],[64], whereas a concave dorsal margin occurs in †*Notelops*, †*Goulmimichthys*, and *Elops* [24],[30]. Also, most ostariophysans (except gonorynchiforms) have a quadrate with a notched dorsal margin (e.g. *Brycon*, *Opsariichthys*; [13],[49]), forming a fenestra together with the notched metapterygoid. Finally, the quadrate of *Amia* has a sinuous or sigmoid dorsal margin [14].

In his phylogenetic analysis on the relationships of †Ichthyodectiformes, Stewart [25] proposed a similar character to code the variation of the dorsal margin of the quadrate. However, the character used by the author comprised only two possible conditions, the first including our states (0), (1) and (2); and the second including our state (3), which occurs in some ichthyodectiform taxa not included in our analysis. We have considered the straight, concave, and convex dorsal margins of the quadrate as different states because these conditions are easily scored in our taxonomic sample. On the other hand, Alvarado-Ortega [23] also coded the presence/absence of a sigmoid-shaped dorsal margin of the quadrate.

45- Symplectic: articulates with lower jaw (0); does not articulate with lower jaw (1) ([1]: ch. 79, [4]: ch. 170, [7]: ch. 162, [8]: ch. 109, [10]: ch. 158, [14]: ch. 61, [16]: ch. 29, [22]: ch. 152)

46- Covering of palatoquadrate area behind and below the orbit by orbital ossifications: partially (0), totally (1) (modified from [3]: chs. 16 and 20, [6]: chs. 21 and 47, [9]: ch. 25, [23]: ch. 14)

In several teleosts the palatoquadrate area behind and below the orbit (e.g. quadrate, metapterygoid) is partially covered by infraorbital bones and, thus, the hyopalatine bones are laterally exposed. This condition is present in basal teleosts such as †*Leptolepis* *coryphaenoides* and †*Tharsis* *dubius*, elopomorphs, clupeiforms, and also in †*Luisiella* *feruglioi* [27],[29]. In contrast, the hyopalatine bones are completely covered laterally by the infraorbital bones in pachycormiforms, ichthyodectiforms, and osteoglossomorphs, forming the cheek wall [3],[9],[12],[23]. In addition, the palatoquadrate area behind and below the orbit of *Lepisosteus*, †*Obaichthys*, and aspidorhynchiforms is totally covered by both suborbital and infraorbital bones [16],[17],[61], whereas the hyopalatine bones are partially covered by the infraorbital bones, and therefore exposed, in *Amia* and the pycnodontid †*Mesturus* [14],[18].

A similar character was used in previous phylogenetic analyses of the relationships of osteoglossomorphs ([3]: ch. 16, [6]: ch. 47, [9]) and ichthyodectiforms [23] but only referring to the partial or complete covering of the palatoquadrate bones by infraorbital bones. Because our taxon sampling is different, in order to include all the observed variation we have modified this character, by considering the partial or total covering of the palatoquadrate area by orbital bones (i.e. infraorbitals and suborbitals). As noted by Hilton [9], a seemingly similar character is used by Li and Wilson ([3]: ch. 20, [6]: ch. 21), who coded the presence/absence of a cheek wall formed by the enlargement of the 1^st^ to 3^rd^ infraorbitals.

47- Shape of branchiostegal rays: variable, including acinaciform and spathiform branchiostegals (0); uniform, only spathiform branchiostegal rays (1)

Branchiostegal rays are the elements located ventral to the bones of the opercular series associated with the hyoid arch (generally anterior and posterior ceratohyals). Mc Allister [65] described the three most common shapes of teleostean branchiostegal rays that may occur in a single individual: filiform, scimitar-like or acinaciform, and spatulated or spathiform. According to our observations, spathiform branchiostegal rays are widely distributed among neopterygians, including teleosts, and are the only type of branchiostegal rays forming this series in most ostariophysans, clupeomorphs, euteleosts (e.g. umbrids), as well as in pachycormiforms, †*Aspidorhynchus*, †*Mesturus*, and *Amia*. In contrast, in most teleostean groups, including most basal representatives, the branchiostegal series is not formed by rays of uniform shape, but variable, with acinaciform and filiform branchiostegal, in addition to spathiform, branchiostegal rays. Acinaciform branchiostegal rays are generally absent in non-teleostean neopterygians, except for *Lepisosteus*, but also occur in some basal actinopterygians, like †*Propterus*. These rays are placed anterior to spathiform branchiostegal rays, generally associated with the anterior ceratohyal. Filiform branchiostegal rays occur less frequently and have been only observed in some teleosts, in which they are located anteriorl and followed by acinaciform and spathiform branchiostegal rays. The latter is the condition present in †*Luisiella* *feruglioi*.

48- Beryciform foramen in anterior ceratohyal: present (0), absent (1) (modified from [23]: chs. 32 and 41, [25]: ch. 13)

The name beryciform was used by Mc Allister [65] to describe the oval foramen occurring in the middle section of the anterior ceratohyal of beryciform teleosts. Besides this particular group of euteleosts, a large beryciform foramen occurs in the anterior ceratohyal of most basal teleosts, like †*Tharsis* *dubius* and †*Thrissops* *formosus* and in non-clupeiforms clupeomorphs (e.g. †*Diplomystus*; [29]), among teleocephalans. Clupeiforms (e.g. *Denticeps*, *Engraulis* in this analysis) and the remaining teleocephalan taxa (e.g. ostariophysans, elopomorphs, osteoglossomorphs, and euteleosts, except for beryciforms) lack a beryciform foramen in the anterior ceratohyal. A beryciform foramen is present in the anterior ceratohyal of †*Luisiella* *feruglioi*, whereas this foramen is absent in the outgroup taxa.

The loss of the beryciform foramen in the anterior ceratohyal was first discussed by Grande [29] in relation to clupeomorph fishes (see above). A different character was used by Stewart [25] in his phylogenetic analysis of †Ichthyodectiformes, coding the absence of a beryciform foramen in this bone and distinguishing between the presence of a wide foramen (fenestra in the original publication) or a narrow foramen in the anterior ceratohyal. Our taxonomic sample does not include specimens with a narrow foramen in the anterior ceratohyal, as defined by Bardack [66] and later used by Stewart [25]: a “longitudinal groove incised on each side of the ceratohyal just above the ventral margin of this bone”. The presence of a narrow fenestra in the ceratohyal was also coded by Alvarado-Ortega [23] in two characters. One makes reference to a narrow fenestra in the ceratohyal and the other to the presence of this fenestra in the anterior ceratohyal. Alvarado-Ortega did not include a discussion of his characters so we may interpret these characters as if they both refer to the same trait on the same ossification, or as if they refer to the presence of a foramen in both anterior and posterior ceratohyal bones. However, according to our observations, the occurrence of a fenestra or foramen in the posterior ceratohyal (epihyal) is not common among teleosts (at least), and was only described by Forey [30] in †*Notelops* and †*Rhacolepis*.

49- Number of hypohyal ossifications: one pair (0), two pairs (1) (modified from [1]: ch. 82, [4]: ch. 179, [7]: ch. 170, [8]: ch. 118, [9]: ch. 51, [10]: ch. 166, [22]: ch. 160, [59]: ch. 239)

A single hypohyal is present in each half of the hyoidean arch of sarcopterygians, basal actinopterygians, and non-teleostean neopterygians (e.g. *Polypterus*, chondrosteans, *Lepisosteus*; [2]), whereas in the majority of teleosts including †*Luisiella* *feruglioi* there are two hypohyals in each half of the hyoidean arch, namely, a dorsal and a ventral hypohyal [67]. Teleosts with only one hypohyal in each half of the hyoidean arch are osteoglossomorphs (with the only exception of *Hiodon*; [15]) and some siluriform taxa (e.g. Loricariidae, Callichthydae; [2]). The single hypohyal of osteoglossomorphs is homologous to the dorsal hypohyal of other teleosts, whereas the hypohyal that ossifies in siluriforms is the ventral hypohyal of other teleosts.

The living bowfin, *Amia* *calva,* presents a particular condition. According to Arratia and Schultze [67] in this fish the ventral portion of the hypohyal cartilage is ossified, corresponding to the ventral hypohyal of teleosts; in some adult individuals this cartilage exhibits a small posterodorsal ossification, which the authors identified as an incipient dorsal hypohyal. However, Grande and Bemis [14] argued that they did not find two pairs of hypohyals in *Amia* *calva* or in fossil amiids. In the present analysis, *Amia* *calva* was coded as having only one pair of hypohyals, as the remaining taxa included in the outgroup.

Arratia [1],[4],[7],[8],[10] and Arratia and Tischlinger [22] coded the presence/absence of two ossified hypohyals in their phylogenetic analyses without refering to the alternative condition. Although in this particular case the non-specified absence in the character used by the cited authors did not include more than one option, we have modified it in order to clearly describe the observed conditions in our taxon sample. Hilton [9] used a similar character that included our states (0) and (1) but also considered an alternative condition where only one pair of greatly reduced in size ossified hypohyals occur. This latter condition is present in some mormyrid osteoglossomorph taxa that are not included in our analysis. On the other hand, Diogo et al. [59] used a rather different character considering the ossification of a ventral and dorsal hypohyal; they proposed a state where “at least one ossified hypohyal is present”, which characterizes all our taxa including *Amia* and *Lepisosteus*, and another state where the ossified hypohyals are absent, a condition that occurs in teleost taxa not included in our analysis (e.g. *Conger*, *Anguilla*).

50- Trajectory of the hyoidean artery in relation to the hypohyals: not piercing hypohyal (0), piercing both dorsal and ventral hypohyals (0), piercing only the ventral hypohyal (1) ([4]: ch. 61, [7]: ch. 57, [10]: ch. 56)

51- Urohyal formed as an unpaired tendon-bone: absent (0); present (1) ([1]: ch. 83, [4]: ch. 180, [7]: ch. 171, [8]: ch. 119, [10]: ch. 167, [22]: ch. 161)

52- Gular plate: present (0); absent (1) ([1]: ch. 93, [4]: ch. 60, [6]: ch. 52, [7]: ch. 56, [8]: ch. 35, [9]: ch. 36, [10]: ch. 55, [16]: ch. 27, [20]: ch. 49, [22]: ch. 54)

**Opercular bones**

53- Suprapreopercle: absent (0); present (1) ([4]: ch. 68, [7]: ch. 64, [10]: 63, [20]: ch. 53, [22]: ch. 62, [33]: ch. 19, [36]: ch. 23, [50]: ch. 32)

54- Ventroposterior (excluding dorsal limb) region of preopercle: narrow or slightly expanded (0); broadly expanded (1) ([4]: ch. 69, [7]: ch. 66, [8]: ch. 36, [10]: ch. 64, [20]: ch. 54, [22]: ch. 63)

55- Shape of preopercle: “L” shaped, with tapering ends (0), crescent-shaped, long and narrow (1); triangular (2) (modified from [1]: ch. 90, [14]: ch. 20, [16]: ch. 21)

The actinopterygian preopercle may have very different shapes. Several basal teleosts (e.g. †*Tharsis* *dubius*, †*Ascalabos* *voithii*, †*Cavenderichthys* *talbragarensis*; [20]) as well as teleocephalans (except for *Esox* and *Engraulis* in this analysis) have an ‘L’ shaped preopercle, where two distinct limbs (horizontal and vertical) with tapering distal ends are clearly defined. This is the condition present in †*Luisiella* *feruglioi* as well as in *Lepisosteus* and pachycormiforms among the outgroup taxa. In contrast, the preopercle is a triangular bone in several Mesozoic basal teleosts and fossil teleocephalans, like varasichthyids, †*Dorsetichthys* *bechei*, †*Eurycormus* *speciosus*, †*Pholidophorus* *latiusculus*, †*Siemensichthys*, aspidorhynchiforms, pycnodontiforms, †*Notelops*, †*Rhacolepis*, and †*Lycoptera* [1],[10],[18],[30]. This bone is crescent-shaped and lacks distinct horizontal and vertical limbs in the teleocephalans *Esox* and *Engraulis* and also in *Amia* [14],[20],[29].

A binary character distinguishing a crescent-shaped preopercle and a preopercle with two limbs was proposed by Brito [16]. The first condition described by the author corresponds to our state (1), but our states (0) and (2) are considered by Brito as similar (i.e. two-limbed preopercle). We have modified Brito’s character because we consider that two limbs are only well defined in an ‘L’ shaped preopercle whereas they are not clearly defined in a triangular-shaped preopercle. Grande and Bemis [14] used a similar character including our states (0) and (1) but also two alternative conditions present in some amiiforms and parasemionotiforms that are not represented in our taxonomic sample (i.e. preopercle crescent-shaped, wide in the middle, tapering dorsally and ventrally; ovoid preopercle). On the other hand, Arratia [1] coded only the presence/absence of a crescent-shaped preopercle, without making any reference to the other recorded shapes of this bone.

56- Interopercle: present (0); absent (1) ([1]: ch. 92, [4]: ch. 195, [7]: ch. 186, [8]: ch. 131, [10]: ch. 182, [14]: ch. 68, [16]: ch. 23, [22]: ch. 173)

57- Anteroventral corner of the opercle: of 70° (0), more than 70° (1) ([6]: ch. 28)

**Jaws**

58- Relative position of premaxillary bones: forming part of the tip of the snout, in contact with each other at the midline (0), forming part of the tip of the snout but slightly separated by ethmoidal ossifications (1), constituting the main element of an elongated snout, in contact with each other at the midline (2), laterally placed and not forming part of the tip of the snout, separated by ethmoidal ossifications (3)

According to our observations, the premaxillary bones of most teleost taxa, including †*Luisiella feruglioi,* are small ossifications located at the anterior end of the skull, forming part of the tip of the snout. Among these taxa, both premaxillary bones are in contact with each other at the midline in most studied teleost species and in *Amia,* whereas they are slightly separated from each other by ethmoidal ossifications in some teleocephalans, like *Heterotis* [9],[20]. In aspidorhynchiforms, the premaxillary bones are large ossifications in contact with each other at the midline and constitute the main elements of an elongated snout [16]. Particularly, the premaxillary bones of pachycormiforms (†*Pachycormus* and †*Hypsocormus* in this analysis) are laterally placed without having contact with each other and do not form part of the snout, which is formed almost completely by a large rostrodermethmoid bone [12],[68].

59- Premaxillary ascending process: absent (0), present (1) ([1]: ch. 53, [5]: ch. 25)

60- Maxillary teeth: present (0), absent (1) ([20]: ch. 39, [50]: ch. 21, [59]: ch. 155)

61- Ventral margin of maxilla: straight or almost straight (0); slightly convex (1); slightly concave (2) ([22]: ch. 185)

62- Shape of the maxilla (excluding articular process): uniformly deep (0), approximately triangular (1), bulbous (2) (modified from [5]: ch. 23 in part)

Although the maxillary bones have several shapes in teleosts, the morphology of the main portion of the bone (i.e. excluding the articular process) can be represented by three main general shapes. Most basal teleosts and teleocephalans have a maxilla which is uniformly deep from its anterior to its posterior termini, that is, the depth of the bone is constant along the length of the bone. This is the condition present in †*Luisiella* *feruglioi*. Nonetheless, the main portion of the maxilla is triangular in shape in some osteoglossomorphs (*Hiodon*, *Heterotis*, this analysis; [9]) and salmoniforms (*Esox*, this analysis). In particular, the maxilla of the gonorynchiforms *Chanos,* †*Gordichthys*, and †*Tharrhias* is posteriorly expanded, resulting in a bulbous outline [5],[69],[70]. The maxilla of amiids, aspidorhynchiforms, and pachycormiforms is triangular in shape [12],[14],[16],[71], whereas the maxilla of the pycnodontiform †*Mesturus* is uniformly deep ([18]: fig. 4.22). This character (as well as ch. 63 and 64) has been coded as inapplicable for *Lepisosteus* and †*Obaichthys*, the maxilla of which is extremely reduced and fused to the most posterior toothed infraorbitals [61].

The character proposed by Grande and Poyato-Ariza [5] referred to the shape of the posterior margin of the maxilla (our character 63) and is discussed below. However, it included information on the shape of the maxilla of some gonorynchiforms (i.e. expanded, forming a bulbous outline’), which we have incorporated in the present character.

63- Shape of the posterior border of the maxilla: concave (0), convex or straight (1), acute (2), notched (3) (modified from [1]: ch. 57, [5]: ch. 23, [8]: ch. 108, [14]: ch. 62)

In general, the posterior border of the maxilla in most basal teleosts and teleocephalans like †*Tharsis* *dubius*, †*Leptolepides* *sprattiformis*, †*Diplomystus*, *Elops*, *Thymallus*, *Oncorhynchus* is convex or straight [20],[27],[50]; this is also the shape of the posterior border of the maxilla of †*Luisiella* *feruglioi*. A maxilla with an acute posterior border occurs in the Cretaceous †*Rhacolepis* and †*Notelops* [21],[30], whereas a maxilla with a concave posterior border occurs in some cypriniforms, like *Opsariichthys* [49] and in pachycormiforms [12]. A maxilla with a notched posterior border is present in *Amia* *calva* and †*Amia* *pattersoni* [14].

A similar character distinguishing a convexly rounded or straight posterior border of the maxilla from an excavated posterior border (concave or with a posterior maxillary notch) was used by Grande and Bemis [14]. This character was modified by Arratia [8] who included an additional state for a sharp posterior border of the maxilla, and, some years later, Arratia [1] recognized an additional state for the irregularly-shaped maxillary posterior border of *Lepisosteus*. We have modified these character enunciations by separating the notched posterior border from the concave posterior border of the maxilla, because they represent different conditions that can be easily discriminated. Also, we have excluded Arratia’s [1] irregularly-shaped condition because the maxilla of lepisosteids is extremely reduced, and fused to the most posterior toothed infraorbitals, and therefore, the shape of its posterior border cannot be described unambiguously. On the other hand, Grande and Poyato-Ariza [5] proposed a character for the shape of the posterior margin of the maxilla, distinguishing a straight from a swollen to bulbous outline representing the shape of the posterior border of the maxilla of members of Chanoidei. We have modified this character because we consider that the posterior border of the maxilla of Chanoidei is convex (our state (1)) and that the ‘expanded, forming a bulbous outline’ attribute describes the shape of the maxilla and not the shape of the posterior border of this bone. The latter attribute has already been discussed in the previous character (ch. 62).

64- Number of supramaxillary bones: two (0), one (1), none (2) ([1]: ch. 59, [16]: ch. 19)

65- Relative position of supramaxilla (ae): placed dorsal to the dorsal margin of maxilla (0); placed posterodorsal to the maxilla (1) ([1]: ch. 61, [10]: ch. 42, [22]: ch. 61)

66- Quadrate-mandibular articulation: posterior to orbit (0); placed below the posterior half of orbit (1); placed below anterior half of orbit (2); anterior to orbit (3) ([1]: ch. 62, [4]: ch. 51, [7]: ch. 47, [8]: ch. 30, [10]: ch. 43, [20]: ch. 41, [22]: ch. 45)

67- Predentary bone: absent (0); present (1) ([16]: ch. 24, [21]: ch. 23, [23]: ch. 48)

68- Independent retroarticular bone: present (0); absent (1) ([59]: ch. 247)

69- Retroarticular and quadrato-mandibular joint: retroarticular included in the joint facet for quadrate (0), retroarticular excluded from the joint facet for quadrate (1) ([9]: ch. 43, [20]: ch. 42, [24]: ch. 36, [33]: ch. 15, [36]: ch. 19, [59]: ch. 248)

70- Coronoid bone(s) in lower jaw: present (0); absent (1) ([1]: ch. 66, [4]: ch. 184, [7]: ch. 175, [8]: ch. 122, [10]: ch. 171, [22]: ch. 163)

71- Surangular bone in lower jaw: present (0); absent (1) ([1]: ch. 67, [4]: ch. 185, [7]: ch. 176, [8]: ch. 123, [10]: ch. 172, [16]: ch. 26, [22]: ch. 164)

72- Independent articular bone: present (0), absent, fused with angular (1), absent, fused with angular and retroarticular (2) ([1]: ch. 63, [8]: ch. 31)

73- Prearticular bone in lower jaw: present (0), absent (1) ([1]: ch. 68, [40]: ch. 26)

74 - Postarticular process of lower jaw: poorly developed (0); well developed, extending posterior to the articular facet for quadrate (1) ([22]: ch. 48)

75- Characteristic notch (so-called leptolepid notch) in the anterodorsal ascending margin of the dentary: absent (0); present (1) ([1]: ch. 65, [4]: ch. 56, [5]: ch. 29, [7]: ch. 51, [8]: ch. 33, [10]: ch. 50, [20]: ch. 45, [22]: ch. 49)

76- Marginal dentition on the jaws: uniform (0), variable (1)

Among teleosts there are many different types of dentition on the jaws; however a distinction can be made between those cases where the marginal teeth are of similar shape and size in the premaxilla, maxilla and dentary, from other cases where teeth are of different shape and size in these bones in the same individual. Generally in basal teleosts as well as basal teleocephalans, the marginal dentition on the jaws is uniform, including small conical teeth, like in †*Leptolepis* *coryphaenoides*, †*Tharsis* *dubius*, †*Cavenderichthys* *talbragarensis*, †*Diplomystus*, *Megalops* [20],[27],[31], and †*Luisiella* *feruglioi*. In contrast, some living teleosts exhibit variable marginal dentition, including different-sized conical, styliform or fang types of teeth, like those observed in *Brycon*, *Elops*, and *Esox* [13],[35],[49]. Among the outgroup taxa; *Amia* *calva* and aspidorhynchiforms have a variable marginal dentition on the jaws [14],[16].

**Cephalic sensory canal system**

77- Condition of cephalic sensory canals: with branched tubules (0), with simple tubules (1) ([8]: ch. 17)

78- Cephalic sensory canal components: continuous (0); interrupted between them (1) ([4]: ch. 26, [7]: ch. 22, [10]: ch. 22, [22]: ch. 21)

79- Middle pit line: leaving a groove or pore-line on the parietal and pterotic (or dermopterotic) (0), leaving a groove or pore-line on the parietal only (1), leaving no trace on the bones (2) (modified from [1]: ch. 36, [4]: ch. 30, [7]: ch. 26, [8]: ch. 19, [10]: ch. 26, [20]: ch. 25, [22]: ch. 25, [31]: ch. 31, [33]: ch. 10, [36]: ch. 12, [40]: ch. 40)

The middle pit line is one of the pit lines that form part of the teleostean cephalic sensory system [72], which might leave a visible groove or a pore line in the parietal and pterotic in the basal teleosts †*Pholidophorus* *gervasutti*, †*Eurycormus*, †*Dorsetichthys* *bechei*, †*Leptolepis* *coryphaenoides* [1],[31], and some members of the †Varasichthyidae (e.g. *Varasichthys*, *Protoclupea*; [10]). In other basal teleosts as well as fossil and living teleocephalans the middle pit line leaves a groove or a pore line only in the parietal bone, without extending onto the pterotic bone. This occurs in the basal teleosts †*Allothrissops* and †*Tharsis* *dubius*, among others, and in some elopomorphs (e.g. *Megalops*), ostariophysans (e.g. *Chanos*, *Brycon*, *Opsariithys*); clupeomorphs (e.g. *Diplomystus*), osteoglossomorphs (e.g. *Heterotis*), and euteleosts (†*Orthogonikleithrus*, †*Humbertia*, †*Leptolepides*; [20],[48]). In contrast, there is no trace of a middle pit line on the parietal of some living teleocephalans, like *Elops*, *Hiodon*, and *Esox* [15],[20]. Particularly, the middle pit line leaves a deep groove on the parietal of †*Luisiella* *feruglioi* and extends onto the pterotic bone in a shallower groove accompanied by a pore line [73].

The teleostean pterotic bone is a compound ossification formed by the fusion of the autopterotic and dermopterotic bones of non-teleostean actinopterygians [68]. Among the outgroup taxa, a pterotic bone is present in †*Pachycormus* where the middle pit line crosses this bone and the parietal [12] but is absent in the other outgroup taxa including aspidorhynchids. In these taxa only a dermopterotic is found in *Amia*, *Lepisosteus,* and aspidorhynchiforms whereas both bones are present in pycnodontiforms, although not observed in †*Mesturus* [18]. A middle pit line leaving a groove on both parietal and dermopterotic bones has been described in *Amia*, *Lepisosteus,* and aspidorhynchiforms [14],[16],[17].

This character has been first proposed by Patterson [40] and Patterson and Rosen [31] to code the presence or absence of a middle pit line groove on the dermopterotic bone and it was used in several subsequent phylogenetic analyses by Arratia [1],[4],[7],[8],[10],[20],[33],[36] and Arratia and Tischlinger [22]. We have modified this character in order to describe all the variation contained within our taxon sample.

80- Relative association of the ethmoidal commissure and ossifications of the ethmoidal region: not associated, there is no trace of an ethmoidal commissure (0), the ethmoidal commissure is completely enclosed in a bony canal (1) (modified from [4]: ch. 29, [6]: ch. 1, [7]: ch. 25, [10]: ch. 25, [20]: ch. 24, [22]: ch. 24, [33]: ch. 9, [36]: ch. 11)

In the majority of basal teleosts as well as non-teleostean neopterygians the ethmoidal commissure is completely enclosed in a canal within the rostral bone, in the anterior part of the skull. This is the condition present in *Amia calva* [14], *Lepisosteus* [17], and in the basal teleosts †*Pholidophorus* *gervasuttii* [1], †*Eurycormus* [32], and †*Dorsetichthys* *bechei* [41], where the rostral bone is an independent ossification of the ethmoidal region. However, in most teleosts, the rostral bone and other ethmoidal ossifications are fused to each other forming different bones (i.e. supraethmoid, compound mesethmoid, rostrodermethmoid, see [41]: 473, for an explanation of fusion of bones in the teleostean ethmoidal region). Although the ethmoidal ossifications are variable in teleosts, a bone-enclosed ethmoidal commissure occurs in those taxa that have a compound mesethmoid forming part of the snout, like the basal teleosts, †*Leptolepis* *coryphaenoides* and †*Tharsis* *dubius* [27],[41] and the fossil and living elopomorphs (e.g. †*Anaethalion*, *Elops*, *Megalops*; [20]). In contrast, there is no trace of an ethmoidal commissure in the ethmoidal region of some basal teleosts (ichthyodectiforms, varasichthyids; [10],[31]) as well as in most living groups of teleosts, like osteoglossomorphs, ostariophysans, clupeomorphs, and euteleosts. This is also the condition of †*Luisiella* *feruglioi*. In particular, pachycormiforms have a bone-enclosed ethmoidal commissure in the compound rostrodermethmoid, which is the only ethmoidal ossification present in the snout [12].

This character is a modification of that used by Arratia in several phylogenetic analyses [4],[7],[10],[20],[33],[36] and Arratia and Tischlinger [22], where the presence or absence of an ethmoidal commissure that penetrates and passes through the entire width of a broad mesethmoid were coded. As this condition describes mainly that of elopomorphs but not those of the remaining taxa (with or without trace of an ethmoidal commissure) and because it does not include a reference to other ethmoidal ossifications present in teleosts, we have modified the character in order to describe the different conditions present in our taxon sample. On the other hand, Li and Wilson [6] proposed a character to record the presence or absence of an ethmoidal commissure within Osteoglossomorpha (their taxon sample). Although our character is rather similar to their character, we have preferred to consider the relative association of this commissure and the ethmoidal ossifications, which is what we can score in fossil specimens.

81- Relative position of supratemporal commissure: it pierces extrascapular bones (0), it pierces parietal bones only, or parietals and supraoccipital (1) (modified from [4]: ch. 31, [6]: ch. 10, [7]: ch. 27, [9]: ch. 10, [10]: ch. 27, [20]: ch. 26, [22]: ch. 26, [29]: ch. 3, [36]: ch. 13)

In most neopterygians, including basal teleosts as well as basal teleocephalans, the supratemporal commissure pierces the extrascapular bones, as described in †*Eurycormus*, †*Pholidophorus* *gervasutti*, †*Leptolepis* *coryphaenoides*, †*Tharsis* *dubius*, †*Cavenderichthys*, †*Notelops*, †*Lycoptera*, †*Anaethalion knorri,* among others [20],[28],[30],[68]. This is also the condition observed in some living euteleosts (e.g. *Thymallus*), even when the extrascapular bones are reduced to small tubular ossifications [50] and it is the condition present in †*Luisiella* *feruglioi*. On the other hand, in clupeomorphs, some osteoglossomorphs (e.g. *Heterotis*; [9]), and most ostariophysans [74] the supratemporal commissure might pierce the parietal bones (e.g. †*Santanaclupea*, *Denticeps*; [29],[31],[37]) or both parietals and supraoccipital bones, like in clupeiforms (e.g. *Engraulis*). In these taxa the extrascapular bones are reduced and, according to Patterson [48] and later Grande [29], at least in clupeomorphs the passage of the supratemporal commissure through parietals and supraoccipital is the result of the fusion of the lateral and median extrascapular to these bones, respectively.

Grande [29] first proposed a character to describe the condition observed in clupeomorphs, where some basal taxa exhibit a supratemporal commissure passing through parietals alone or through both parietals and supraoccipital bones. This character was used later by Arratia [4],[7],[10],[20],[36] and Arratia and Tischlinger [22] in several phylogenetic analyses of the relationships of basal teleosts. Besides, in their phylogenetic analyses of the relationships of osteoglossomorphs, Li and Wilson [6] and later Hilton [9] coded the occurrence of a supratemporal commissure passing through parietals. We have modified these previous characters in order to accurately describe the observed conditions of the supratemporal commissure in the taxa included herein.

82- Parietal portion of supraorbital sensory canal: present (0), absent (1) ([5]: ch. 11, [9]: ch. 11, [24]: ch. 4)

83- Composition of preopercular sensory canal: four or less, short simple tubules (0), seven or eight (up to 10), short simple or branched tubules (1), 12 or more long, simple or branched tubules (2) (modified from [4]: chs. 70 and 71, [7]: chs. 66 and 67, [8]: chs. 38 and 39, [10]: chs. 65 and 66, [20]: chs. 55 and 56, [22]: chs. 64 and 65)

The preopercular sensory canal produces only four or less short and simple tubules in *Amia* and *Lepisosteus* and in the pycnodontiform †*Mesturus* [14],[17],[18]. The condition is unknown in pachycormiforms and most aspidorhynchiforms except †*Vinctifer*, the canal of which gives off seven or eight tubules. The preopercular sensory canal includes at least 12 long, simple or branched tubules in the basal teleosts †*Eurycormus*, †*Siemensichthys*, †*Pholidophorus*, †*Dorsetichthys* *bechei*, †*Leptolepis* *coryphaenoides*, and the varasichthyids [1],[8],[10],[27]. In these taxa, the preopercular tubules reach the ventral margin of the preopercle (or are very close to this margin). In contrast, in the Jurassic †*Ascalabos*, †*Thrissops*, the Cretaceous †*Rhacolepis*, the basal elopomorph †*Anaethalion*, and the basal euteleost †*Humbertia*, among others ([20],[30],[48], pers. obs.), the preopercular sensory canal includes only seven or eight, relatively short tubules that do not reach the ventral margin of the preopercle and might be simple or branched, as is the case in the more basal †*Vinctifer* (pers. obs.). This is also the condition present in †*Luisiella* *feruglioi*. The number of tubules is further reduced in more derived teleocephalans and the most frequent condition among living taxa resembles that of holosteans and †*Mesturus* with only few (four or less), short and simple tubules in the preopercular sensory canal, as has been described in ostariophysans, euteleosts, clupeomorphs, and osteoglossomorphs [50],[58],[72],[75]. Also, the preopercular sensory canal of †*Cavenderichthys* *talbragarensis* and †*Leptolepis* *koonwarri* includes only four or less short and simple tubules [20],[28],[62].

This character merges two characters used in previous analyses [4],[7],[8],[10],[20],[22], which were enunciated as binary characters coding essentially the presence/absence of our states (0) and (2). We have proposed a new character merging this information and adding the new character state (1) to improve the representation of the observed morphological variation.

84- Mandibular canal: enclosed in bone along the whole lower jaw (0); partially running in a bony tube and a groove (1) ([6]: ch. 46, [22]: ch. 52)

**Branchial apparatus**

85- Endoskeletal basihyal: absent (0), present (1) ([40]: ch. 42)

86- Covering of basibranchials 1 – 3 by tooth plates: not covered, basibranchials tooth plates absent (0), basibranchials overlain by paired distributed tooth plates (1); basibranchials overlain by median tooth plates (2) (modified from [4]: ch. 62, [7]: ch. 58, [10]: ch. 57, [20]: ch. 50, [22]: ch. 56, [36]: ch. 24, [40]: ch. 6)

Although the configuration of the teleostean gill arch might be variable in different groups, in general, the first basibranchial elements (1-3) are covered by median, unpaired tooth plates [40],[75],[76]. This is the condition observed in most living groups of teleosts, like elopomorphs, osteoglossomorphs, clupeomorphs, and euteleosts [40],[75],[77],[78]. In contrast, most ostariophysan taxa lack tooth plates covering the basibranchials 1-3, as observed in the cypriniform *Opsariichthys* [49],[79]. Basibranchial 1-3 tooth plates are also absent in the branchial apparatus of *Amia* *calva* [14],[75], whereas these plates occur as paired elements in *Lepisosteus* [60],[75]. The condition of most fossil teleosts (including †*Luisiella* *feruglioi*) and more basal taxa incorporated in this analysis is unknown.

A similar character was proposed by Patterson [40] including only our character states (1) and (2) and, thus, the absence of basibranchial tooth plates was not taken into account. Also, the author included in the definition of state (2) a reference to those cases where basibranchial tooth plates are not paired elements but smaller asymmetrical elements, a condition that occurs in basal actinopterygians (e.g. *Polypterus*). Because these taxa are not included in our taxon sample, we did not incorporate this attribute to this character. In addition, several phylogenetic analyses performed by Arratia [4],[7],[10],[20],[36] and Arratia and Tischlinger [22] included a binary character that coded the presence/absence of basibranchials 1-3 and basihyal cartilages covered by median tooth plates. We have modified this character, removing the reference to the basihyal because the basyhial is only present in teleost taxa whereas it is absent in non-teleostean neopterygians. Also, this character has been modified because different conditions are included within the absence state (i.e. absence of tooth plates, presence of paired tooth plates).

87- Tooth plate(s) associated (attached, ankylosed or fused) to pharyngobranchial 1: present (0); absent (1) ([22]: ch. 57)

88- Tooth plate(s) associated (attached or fused) to pharyngobranchial 2: present (0); absent (1) ([22]: ch. 58)

89- Tooth plate(s) associated (attached or fused) to pharyngobranchial 3: present (0); absent (1) ([22]: ch. 59)

**Postcranium**

**Vertebrae and intermuscular bones**

90- Types of vertebral centra: amphicoelous (0), opisthocoelous (1) (modified from [1]: ch. 95, [4]: ch. 74, [7]: ch. 70, [8]: ch. 40, [10]: ch. 69, [22]: ch. 68)

Usually in actinopterygians, including teleosts, vertebral centra are of the amphicoelous type, that is, both anterior and posterior articular surfaces are concave. However, lepisosteids and obaichthyids, recently grouped together within the Superfamily Lepisosteoidea [61], have opisthocoelous vertebral centra, which are characterized by anteriorly convex and posteriorly concave surfaces. Opisthocoelous vertebrae are commonly found in different groups of tetrapods, but are very rare in actinopterygians and, apart from the gars, they are only found in the blennid perciform *Andamia* [80].

This character was slightly modified from that of Arratia [1],[4],[7],[8],[10] and Arratia and Tischlinger [22], where the presence/absence of opisthocoelous centra was coded. Herein we coded the alternative condition to an opisthocoelous vertebra (i.e. amphicoelous vertebra), commonly present in actinopterygians.

91- Composition of caudal vertebral centra: mineralized chordacentrum and arcocentra (0), chordacentrum and basal part of arcocentra surrounded by autocentrum (1), basal part of arcocentra surrounded by autocentrum (2) ([4]: ch. 75, [7]: ch. 71, [8]: ch. 41, [10]: ch. 70, [20]: ch. 57, [22]: ch. 69)

92- Relative condition of midcaudal vertebrae autocentrum: thin and smooth (0), thick and sculptured (1), thick and smooth (2) ([20]: ch. 58)

93- Relationship between autocentrum of midcaudal vertebrae and notochord: autocentrum not constricting or slightly constricting the notochord (0), autocentrum strongly constricting the notochord (1) ([20]: ch. 60, [36]: ch. 33, [81]: ch. 5)

94- Relationship between abdominal neural arches and vertebral centra: unfused (0), fused, except for the first five or six (1), all abdominal neural arches fused (2) ([4]: ch. 79, [10]: ch. 74, [20]: ch. 61, [24]: ch. 46, [36]: ch. 31)

95- Relative condition of the neural arches of the anterior abdominal vertebral centra (3 to 5): separated from each other (0), in contact, abutting each other (1) (modified from [4]: ch. 80, [5]: ch. 61, [7]: ch. 76, [10]: ch. 75, [20]: ch. 62, [22]: ch. 74, [36]: ch. 28)

In the teleostean vertebral column, the neural arches of the anterior abdominal centra are generally small ossifications that are not in contact with the neural arches of the immediately preceding and succeeding vertebral centra. This is the common condition observed in most teleosts, and it is also the condition present in †*Luisiella* *feruglioi*. Particularly, in some ostariophysans, especially in gonorynchiforms, the dorsomedial portions of the anterior neural arches of the abdominal region are expanded and they abut against each other and with the posterior margin of the exoccipital, forming a roof over the neural canal [5],[49].

A binary character coding the absence/presence of expanded dorsomedial portions of the anterior neural arches, which abut on each other and with the posterior margin of the exoccipital bone, was used in previous phylogenetic analyses of teleosts [4],[7],[10],[20],[22],[36]. We have slightly modified this character to describe the opposite conditions of having separated versus contacting neural arches in the anterior portion of the abdominal region of the vertebral column. A similar character was proposed by Grande and Poyato-Ariza [5] including the states proposed here, and an additional state to code those cases where the adjoining neural arches laterally overlap one another. We have not included this last condition because it is not present in our taxon sample.

96- Number of neural spines in each abdominal vertebra: two (0), one (1) (modified from [4]: ch. 81, [7]: ch. 77, [8]: ch. 45, [10]: ch. 75, [20]: ch. 63, [22]: ch. 74, [33]: ch. 24, [36]: ch. 32)

In basal teleosts and basal teleocephalans (e.g. *Elops*; [20],[35]) each of most (if not all) abdominal vertebral centra bears the two separate halves of the neural arch, each of which, in turn, forms a neural spine that remains separated from its counterpart during the whole life. Consequently, two distinct neural spines can be observed in the abdominal column of these taxa. This is the condition present in †*Luisiella* *feruglioi* as well as in *Amia*, *Lepisosteus,* and the pycnodontid †*Mesturus* [14],[17],[18]. In contrast, fusion of these halves producing a median neural spine in the abdominal region occurs in many living teleocephalan taxa, like ostariophysans (e.g. *Brycon*, *Chanos*; [5],[13]), some clupeomorphs (e.g. *Denticeps*, *Engraulis*; [29]), and osteoglossomorphs (e.g. *Hiodon*, *Heterotis*; [9],[15]). The condition in pachycormids and aspidorhynchiforms is unknown.

Although a similar character was proposed in several previous phylogenetic analyses of basal teleostean relationships [4],[7],[8],[10],[20],[22],[33],[36], it made reference to the presence of separate or fused halves (forming a median spine) of abdominal neural arches. Provided that two separate halves of the neural arches exist, two distinct neural spines are present in each vertebra of the abdominal region of the column. Because this last feature is easier to be observed in fossils, the original enunciation of the character was slightly modified to refer to the presence of two versus one neural spine in each abdominal vertebra.

97- Scaphium: absent (0), present (1) ([22]: ch. 190)

98- Shape of proximal portion of first pleural rib (on third vertebral centrum): narrow (0), slightly expanded (1), greatly expanded, forming the tripus (2) (modified from [5]: ch. 66, [22]: ch. 191)

In most teleosts, except for ostariophysans, the first pleural rib is similar in shape to the consecutive ribs, which contact the vertebral centra by means of a narrow proximal portion. This is the condition present in the anterior axial skeleton of †*Luisiella* *feruglioi* and in the taxa included in the outgroup. However, in ostariophysan taxa the first pleural rib is greatly modified [49],[82]. In gonorynchiform ostariophysans, and in comparison with the following ribs and with the same element of other groups of teleosts, this rib is expanded proximally and it is shortened in length distally [5],[21]. Also in these fishes, the parapophysis of the third vertebra is anteriorly elongated. In contrast, in otophysans (i.e. characiforms, siluriforms, cypriniforms and gymnotiforms; [49]) both the parapophysis and rib of the third vertebral centrum are greatly expanded proximally and anteriorly elongated. Besides, the rib is blunt distally, and forms a thin, curved posterior process (termed the transformator process), which is attached to the gasbladder. The resulting ossification, which is part of the Webberian apparatus, is named the tripus and is a unique feature of otophysans [49],[82]. Rosen and Greenwood [82] proposed that the modified first pleural rib of Gonorynchiformes might correspond to a previous stage to the occurrence of the tripus of otophysans.

Grande and Poyato-Ariza [5] proposed a similar character to distinguish a widened and shortened first pleural rib from a first pleural rib similar to the posterior ones. Even though the authors mentioned that this rib (on the third vertebral centrum) is both widened and shortened as in Gonorynchiformes or further modified forming the tripus in otophysans, they did not include a different state to code the latter condition. We have modified the original character proposed by Grande and Poyato-Ariza in order to include all the observed conditions in our taxon sample. On the other hand, Arratia and Tischlinger [22] included a binary character where the presence/absence of the tripus of otophysans was coded.

99- Shape of abdominal neural arches in lateral view: tapering distally (0), distally expanded, hourglass-shaped (1)

In the abdominal region of teleosts, the neural arches are generally concise ossifications. In most basal teleosts, like †*Pachycormus*, †*Tharsis*, †*Thrissops*, †*Leptolepis* *coryphaenoides* ([20], pers. obs.), as well as in most teleocephalans (except for *Hiodon*, *Oncorhynchus*, and *Thymallus* in this analysis; [9],[50]), these neural arches taper dorsally when in lateral view, distally forming the neural spines. However, in some taxa the distal portions of the neural arches of abdominal vertebrae are laterally expanded and also anterodorsally elongated, this resulting in hourglass-shaped neural arches. In these particularly shaped neural arches, the neural spines arise from the posterodorsal margin of the laterally expanded neural arch. This condition occurs in some Jurassic and Cretaceous teleost taxa, like †*Luisiella* *feruglioi*, †*Domeykos*, †*Notelops*, †*Rhacolepis*, †*Bavarichthys*, †*Ascalabos*, and †*Orthogonikleithrus* ([10],[20],[22],[30],[73],[83], pers.obs). Among the outgroup taxa, *Amia*, *Lepisosteus*, and †*Obaichthys* have distally tapering abdominal neural arches whereas the condition is unknown in the remaining groups.

100- Number of transitional vertebrae (between abdominal and caudal regions): four or more (0), three or less (1) ([84]: ch. 228)

101- Supraneural bones: absent (0), present (1) ([6]: ch. 66)

102- Epineural bones: absent (0), present (1) (modified from [1]: ch. 102, [4]: ch. 87, [7]: ch. 84, [8]: ch. 48, [10]: ch. 82, [20]: ch. 65, [22]: ch. 80, [31]: ch. 16)

Epineurals are intermuscular bones that are associated with the neural arches, especially in the abdominal region of the vertebral column of actinopterygians. Its occurrence is common in teleosts, including basal teleost taxa, where they are elongated, solid bones, equal in length to several vertebrae [85]. The epineurals are absent in acantomorph euteleosts (not included in this analysis) as well as in the outgroup taxa. In non-teleostean actinopterygian fishes, short epineurals occur only in a few taxa, like some palaeoniscoids, the chondrostean †*Boreosomus*, and the amiiform †*Caturus* [2],[85],[86]. In these taxa, the bones are preformed in cartilage and ossify perichondrally [85]. †*Luisiella* *feruglioi* has epineural bones on all the abdominal portion of the column [73].

This character was modified from several previously used characters that in general referred to the presence/absence of elongated, solid epineural bones on neural arches [1],[8],[20],[40] or included an additional “other condition” state, to describe the cases where the epineurals are joined to the neural arches by ligaments [4],[7],[10],[22]. We consider that the enunciation of these characters is ambiguous, because it is uncertain whether the absence of elongated, solid epineural processes on neural arches represents the absence of epineurals, or the presence of epineurals of other characteristics. On the other hand, and referring to the “other condition” state, as explained by Patterson and Johnson [85], teleostean epineurals may be fused to the neural arches, articulate with the corresponding arch or be joined to the neural arch by a ligament, but in all cases the epineurals are elongated, solid bones.

103- Epipleural bones: absent (0), present (1) ([1]: ch. 103, [24]: ch. 49)

104- Position of epipleural bones relative to the column: located in anterior caudal region (0), located in abdominal and anterior caudal regions (1) ([40]: ch. 47)

105- Series of dorsal intermuscular bones in caudal region: absent (0), present (1) ([4]: ch. 91, [7]: ch. 87, [10]: ch. 86, [20]: ch. 69, [22]: ch. 83, [36]: ch. 35)

**Pectoral and Pelvic Girdles**

106- Position of emergence of lateral line system relative to supracleithrum: emerging at its upper half (0), emerging at its posteroventral margin (1), lateral line not piercing the supracleithrum (2) ([4]: ch. 92, [7]: ch. 88, [10]: ch. 87, [20]: ch. 70, [22]: ch. 84)

107- Postsupracleithrum: absent (0), present (1) ([4]: ch. 93, [7]: ch. 89, [8]: ch. 52, [10]: ch. 88, [20]: ch, 71, [22]: ch. 85, [33]: ch. 28)

108- Postcleithra: one to three (0), four or more (1), none (2) ([4]: ch. 94, [22]: ch. 86)

109- Relative position of coracoid bones: separated from each other at the midline (0), contacting each other and forming a midventral coracoid symphysys (1) (modified from [4]: ch. 95, [7]: ch. 91, [10]: ch. 90, [20]: ch. 73, [21]: ch. 2, [22]: ch. 87, [23]: ch. 2, [26]: ch. 47)

In most fossil and living teleosts the coracoid bone is usually a small, subtriangular ossification that forms the ventroposterior portion of the pectoral girdle without being in contact with its counterpart. This is the condition present in †*Luisiella* *feruglioi* and in the taxa included in the outgroup. In particular, in the Mesozoic ichthyodectiforms (e.g. †*Allothrissops*, †*Thrissops*, †*Cladocyclus*), as well as in the living osteoglossomorph *Hiodon* (this analysis; [15]), the coracoid is a large, ventrally expanded bone that contacts its counterpart in a long, midventral symphysis [21],[31].

A similar character was first used in a cladistic analysis by Maisey [21], and later by other authors in several phylogenetic analyses [4],[7],[10],[20],[22],[23] to code the presence/absence of a large coracoid bone, meeting its fellow in a long, midventral symphysis. This character definition does not specify the “absence” state, and besides it combines size and position information; thus, it was modified to describe the two conditions regarding the relative position of the bones. In addition, Cavin et al. [26] used a somewhat similar character, although their states referred to a normal coracoid opposite to an enlarged bone, broadly meeting its antimere in the ventral midline. We chose to describe the particular condition of ichthyodectiforms by distinguishing a state where the coracoids are separate from each other from another state where they are in contact at the ventral midline, avoiding any mention to the relative size of the bones.

110- Relationship between pectoral propterygium and first pectoral ray: unfused, propterygium free (0), fused (1) ([16]: ch. 34, [40]: ch. 4)

111- Number of proximal pectoral radials: five or more (0), four or less (1) (slightly modified from [1]: ch. 110, [4]: ch. 191, [7]: ch. 182, [8]: ch. 127, [10]: ch. 178, [22]: ch. 169, [40]: ch. 22)

Among actinopterygians, the total number of proximal pectoral radials (whether cartilaginous or ossified) varies from five to ten proximal pectoral radials in basal actinopterygians, *Amia*, *Lepisosteus* [2],[4],[14],[87] to generally four proximal pectoral radials in teleosts, including basal taxa (except for aspidorhynchiforms; [16]). Although the most frequent number of proximal pectoral radials in teleosts is four, in some living teleost taxa, like some elopomorphs (e.g. anguillids) and paracantopterygian euteleosts [2] there are more than four radials, whereas in some clupeomorphs (e.g. *Denticeps*, this analysis), ostariophysans (siluroids), and euteleosts (e.g. *Umbra*, this analysis) there are less than four pectoral radials [4]. Four proximal radials occur in the pectoral girdle of †*Luisiella* *feruglioi* as well as that of the pachycormiform †*Pachycormus* [12].

A similar character was proposed by Patterson [40], where the presence of four pectoral radials versus five or more of these ossifications was coded. We have slightly modified the first state of this character to describe the condition observed in some fishes of our taxon sample (see above) where less than four pectoral radials occur. The presence of four proximal pectoral radials was also coded by Arratia [1],[4],[7],[8],[10] and Arratia and Tischlinger [22].

112- Bifurcation pattern of pectoral fin rays: symmetric (0), asymmetric, resulting in two unequal secondary rays (1)

A symmetric bifurcation pattern is observed in teleostean pectoral fins, where each ray divides into two secondary rays, which are equivalent in size and shape (e.g. *Diplomystus*, †*Cavenderichthys*, †*Thrissops,* †*Luisiella* *feruglioi*; [20],[88]). Generally, this bifurcation pattern takes place at least two or three times at midlength of the corresponding ray or even more distally. In particular, in pachycormids (†*Pachycormus* and †*Hypsocormus* in this analysis; [12],[89]) as well as in †*Aspidorhynchus* (pers. obs), the pectoral fin shows an asymmetric bifurcation pattern. In these fins, the branching of each ray does not result in equivalent secondary rays but it produces rays of different shapes: a narrow secondary ray laterally and a wide secondary ray medially [89]. In this way, when a pachycormid pectoral fin is observed in a lateral to medial direction at the level where only one branching has taken place, a regular alternating series between narrow and wide rays is observed. This asymmetric pattern has not been observed in other neopterygians.

113- Pectoral axillary process: absent (0), present (1) ([1]: ch. 113)

114- Pelvic axillary process: absent (0), present (1) ([1]: ch. 116)

115- Fringing fulcra on pectoral fins: present (0), absent (1) ([1]: ch. 114, [61]: ch. 75)

116- Abdominal scutes: absent (0), present (1) ([4]: ch. 155, [6]: ch. 75, [7]: ch. 148, [8]: ch. 98, [10]: ch. 144, [20]: ch. 129, [22]: ch. 138, [36]: ch. 86, [59]: ch. 271)

**Dorsal and anal fins**

117- Position of dorsal fin origin relative to the origin of the pelvic fin: posterior, located above boundary between abdominal and caudal regions (0), posterior, located above caudal region (1), anterior (2) (modified from [1]: ch. 188, [4]: chs. 99 and 100, [7]: chs. 95 and 96, [8]: ch. 57, [10]: chs. 94 and 95, [20]: chs. 77 and 78, [22]: chs. 91 and 92, [23]: ch. 3, [25]: ch. 2)

In most teleosts, the dorsal fin is located approximately at midlength of the body, above the posterior portion of the abdominal region and the anterior portion of the caudal region of the column. In these taxa, the dorsal fin is opposite to the pelvic fins and anterior to the anal fin, although the dorsal fin origin might be located anterior to the pelvic fins in a few taxa (e.g. the Cretaceous †*Apsopelix*, †*Crossognathus*, and †*Notelops*, and the Jurassic †*Leptolepides* and †*Orthogonikleithrus*; [10],[20]) or it might be located posterior to the pelvic fins, as it occurs in most teleosts, including †*Luisiella* *feruglioi*. Notwithstanding, in some extinct and living teleost taxa, like ichthyodectiforms, some osteoglossomorphs, esocids, and umbrids, the origin of the dorsal fin is located posterior to the pelvic fins but also posteriorly in the body, above the caudal region of the column (totally or partially) and opposite to the anal fin [23],[31],[44],[57]. This is also the condition found in lepisosteoid lepisosteiforms (*sensu* López-Arbarello [61]) as well as aspidorynchiforms [16], whereas the dorsal fin is placed above the abdominal and caudal regions and posterior to the pelvic fins in amiids [14].

The present character combines two previously proposed characters, one referring to the posterior placement of dorsal and anal fins and another to the occurrence of a dorsal fin origin anterior to that of pelvic fins [4],[7],[8],[10],[20],[22]. Because the occurrence of a posteriorly placed dorsal fin is reliant on a dorsal fin origin posterior to pelvic fins, we have chosen to combine these two characters in order to avoid dependence of traits. Stewart [25] proposed a different character with three states, one coding a dorsal fin placed well ahead of anal fin (similar but not equal to our states (0) and (2) because the relative position of the dorsal fin with respect to the pelvic fins is not determined) and other two states referring to a short, remote dorsal fin opposed to a long, falcate anal fin or to a short anal fin (also coded by Alvarado-Ortega [23]). These states include information about size and position of the fins that have been treated separatedly herein. Finally, Arratia [1] proposed a character (similar but not equal to our state (1)) where the presence of posteriorly placed dorsal fin, closer to caudal fins than to pelvic fins is coded.

118- Shape of dorsal fin margin: straight or slightly convex (0), concave (acuminate dorsal fin) (1) (modified from [4]: ch. 101, [6]: ch. 70, [7]: ch. 97, [8]: ch. 58, [10]: ch. 96, [20]: ch. 79, [22]: ch. 93)

Commonly in teleosts, the dorsal fin margin is straight or slightly convex. In a dorsal fin with straight margin the fin rays gradually decrease in length in a rostral to caudal direction, whereas in that with a convex margin the rays decrease in length from the middle of the fin to both anterior and posterior ends. Dorsal fins of these characteristics are found in most basal as well as living teleosts, including †*Luisiella* *feruglioi*. However, the dorsal fin has a concave margin in ichthyodectiforms, the Cretaceous †*Apsopelix* and †*Bavarichthys*, *Elops*, *Megalops*, and also in the pachycormid †*Hypsocormus*, among teleost taxa [12],[20],[22],[64],[88]. In these particularly shaped dorsal fins, the anterior fin rays are much longer than those of the middle portion producing a concave border.

The occurrence of dorsal and anal fins with concave margins was coded by Arratia [4],[7],[8],[10],[20], who subsequently [22] modified the original character to code only the presence of an acuminate dorsal fin (independently from the shape of the anal fin). On the other hand, Li and Wilson [6] proposed two different states to code the several shapes of the dorsal fin present in osteoglossomorphs: triangular and falcate versus rounded. Considering the taxa included in our taxon sample, we have modified these characters in order to represent the different dorsal fin shapes that we observed.

119- Condition of first dorsal proximal pterygiophore: single structure (0), bipartite or tripartite (1) ([20]: ch. 76, [24]: ch. 40, [33]: ch. 32, [36]: ch. 40)

120- Relationship between first dorsal proximal pterygiophore and dorsal fin rays: it articulates with dorsal procurrent rays and first dorsal ray (0), it articulates with procurrent rays only (1)

In teleosts, the dorsal fin is composed of two different types of rays: procurrent rays and principal rays. Procurrent rays are located anteriorly in the fin and are followed by the principal rays [90]. In non-teleostean neopterygians fringing fulcra might also occur in the dorsal fin, being located on the leading margin of the fin. There are generally one to three unsegmented procurrent rays in teleostean dorsal fins and, unlike dorsal principal rays which have a one to one relationship with proximal pterygiophores, they all articulate with the first proximal pterygiophore; in some taxa also the first principal ray articulates with the first proximal pterygiophore. According to Arratia [90] the most common condition in the most basal teleosts is the one in which the first dorsal pterygiophore articulates only with the procurrent rays, as observed in *Elops*, *Hiodon*, *Brycon*, †*Leptolepides* *haertesi*, *Umbra* [13],[20], among many others. Although less frequently, a first dorsal pterygiophore articulating with procurrent rays and also with the first principal ray was observed in some teleocephalans included in our taxon sample (e.g. †*Diplomystus*, *Heterotis*, *Esox*; [91]). Among stem teleocephalans, in †*Ascalabos*, †*Leptolepis* *coryphaenoides*, and †*Protoclupea* the first dorsal pterygiophore articulates with the procurrent rays as well as with the first principal ray whereas in †*Varasichthys*, †*Pachythrissops*, †*Mesturus*, and †*Cavenderichthys* *talbragarensis* this pterygiophore only articulates with the dorsal procurrent rays. The latter is also the condition present in †*Luisiella* *feruglioi* and in *Amia* and *Lepisosteus* [14],[17],[73].

121- Fringing fulcra on leading margin of dorsal fin: present (0), absent (1) ([1]: ch. 119)

122- Shape of anal fin margin: straight or slightly convex (0), falcate, concave (1) (modified from [4]: ch. 102, [7]: ch. 98, [10]: ch. 97, [20]: ch. 80, [21]: ch. 3, [22]: ch. 94, [25]: ch. 2)

In the majority of the teleosts examined, the anal fin is relatively short (supported by eight to 16 proximal pterygiophores) and has a straight to slightly convex margin [20],[31]. This is the condition present in †*Luisiella* *feruglioi*. However, the members of the extinct order †Ichthyodectiformes have a long, falcate anal fin (supported by c. 20 pterygiophores or even more; [21],[31]). In this fin, a concave margin is defined by the occurrence of a few long anterior rays, followed by many shorter rays that decrease in length gradually (see for example †*Allothrissops*, †*Thrissops*; [31],[88]). The outgroup taxa have an anal fin with a straight or slightly convex margin.

Patterson and Rosen [31] proposed the occurence of a long, falcate anal fin, opposed to a short, remote dorsal fin as a synapomorphy of †Ichthyodectiformes. Thereupon the occurrence of this attribute (so defined) has been used in several phylogenetic analyses including ichthyodectiforms (e.g. [4],[7],[10],[20],[21],[22]). Stewart [25] also coded the occurrence of a long, falcate anal fin opposed to a short, remote dorsal fin but in contraposition to the occurrence of a short anal fin opposed to a short, remote, dorsal fin, and also to those cases where the dorsal fin is located far anteriorly to the anal fin. However, in all cases, the character definition includes more than one trait for two different fins (morphology, length, and position of dorsal and anal fins) that might be independent from one another. Therefore, we have decided to describe the different morphologies and positions of each fin separatedly, as different characters (ch. 117, 118, 120 and present one).

123- Relative position of first anal proximal pterygiophore: placed anterior to fourth haemal spine in the transitional portion of the column (0), placed posterior to fourth haemal spine in the caudal portion of the column (1) (slightly modified from [4]: ch. 103, [7]: ch. 99, [10]: ch. 98, [20]: ch. 81, [22]: ch. 95)

In most teleosts, the first proximal anal pterygiophore is placed close to or immediately behind the last pair of ribs, in association with the transitional portion of the vertebral column (i.e. transition between abdominal and caudal regions). This is the condition observed in most of the teleost taxa examined for this analysis, like †*Leptolepis* *coryphaenoides*, †*Allothrissops*, †*Ascalabos*, *Engraulis* [20],[29],[92], †*Luisiella* *feruglioi* and the taxa included in the outgroup. A different condition is present in some Jurassic euteleosts (e.g. †*Leptolepides*, †*Orthogonikleithrus*) where the first proximal pterygiophore is placed posterior to the fourth haemal spine and therefore, associated with the caudal region of the column [20].

A similar character was used by Arratia in several teleost phylogenetic analyses [4],[7],[10],[20],[22] although it referred to the presence/absence of the first anal pterygiophore placed posterior to the fourth of fifth haemal spine. Even though this is not the situation, we modified the definition of this character to avoid the inclusion of different conditions in the“absent” state.

124- Fringing fulcra on leading margin of anal fin: present (0), absent (1) ([1]: ch. 120)

**Caudal skeleton**

125- Number of vertebrae (including Pu1): < 45 (0), > or = 45 to < or = 65(1), > 65 (2) (modified from [7]: ch. 188, [10]: ch. 184, [14]: ch. 40, [23]: chs. 34, 38 and 50, [26]: ch. 69, [84]: ch. 227)

Although the number of vertebrae has a broad range of variation in teleosts, most taxa studied for this analysis have between 45 and 65 vertebrae in the column [20],[27],[29],[31]. This is the condition present, for example, in the extinct teleosts †*Allothrissops*, †*Protoclupea*, †*Notelops*, and †*Anaethalion* [11],[20],[30],[31], in the living teleosts *Hiodon*, *Brycon*, *Opsariichthys* [13],[15],[84] as well as in the living gar *Lepisosteus* [17]. A few taxa, like †*Cavenderichthys* *talbragarensis*, †*Ascalabos*, †*Apsopelix*, †*Orthogonikleithrus*, and the living mudminnow *Umbra* ([20],[58],[64], pers. obs.) have a lower number of vertebrae, generally between 38 and 43. This is the condition present in †*Luisiella* *feruglioi*, in which an average of 41 vertebrae was determined [73]. Exceptionally, some teleocephalans (e.g. *Elops*, *Oncorhynchus*, *Thymallus*; [50]) as well as *Amia* [14] and the aspidorhynchiforms †*Belonostomus* and †*Vinctifer* [16] have a higher number of vertebrae, ranging from 68 to 78 elements.

A similar character was proposed by many authors [7],[10],[14],[26],[84] but using different ranges for the observed number of vertebrae according to their respective taxon sample (i.e. [7],[10]: c. 40 vertebrae; [14]: 40 to 73 and 75 to 82 vertebrae; [26]: less than 90 and more than 100; [84]: 40 or fewer and 41 or more). Three distinct characters were proposed by Alvarado-Ortega [23] to code the number of vertebrae in †Ichthyodectiformes (i.e. ch. 34, 38 and 50), considering different ranges of total vertebrae (up to 68; 70 to 100; and nearly 100). Because they are not independent from each other, we consider that the total number of vertebrae is being overscored in the analysis. Even though all these ranges are rather subjective, because the number of vertebrae is an almost continuous variable across the fish taxa, the limits of the states used herein are based on the average of the total number of vertebrae we observed in our taxon sample, taking into account the most common range (state (1)) and the less frequent ranges (state (0) and (2)).

126- Number of neural spines in each caudal vertebra: two (0), one (1) ([1]: ch. 101)

127- Shape of neural spines from preural caudal vertebrae 3-5: narrow, without outgrowths (0), distally expanded by anterior and posterior membranous outgrowths (1) (slightly modified from [4]: ch. 107, [7]: ch. 103, [10]: ch. 102, [20]: ch. 85, [22]: ch. 99, [33]: ch. 36, [36]: ch. 44, [81]: ch. 15)

The neural spines born by preural caudal vertebrae 3-5 of some euteleosts, such as †*Erichalcis*, †*Leptolepides*, †*Orthogonikleithrus*, and *Oncorhynchus* [20],[39],[50] are distally expanded by means of anterior and posterior membranous outgrowths, so that the neural spines almost contact each other laterally [20],[81],[83]. In general, the teleostean neural spines of the preural caudal vertebrae 3-5 are narrow and lack membranous outgrowths, although they are laterally compresed in comparison to the anterior midcaudal neural spines. The latter condition is present in the remaining teleocephalan groups included in the present analysis (i.e. elopomorphs, osteoglossomorphs, ostariophysans, and clupeomorphs), in all of the basal teleost taxa as well as in †*Luisiella* *feruglioi* and the outgroup taxa.

A similar character was used in several phylogenetic analyses by Arratia [4],[7],[10],[20],[33],[36],[81] and Arratia and Tischlinger [22]. We have slightly modified the original enunciation of the character to define the alternative condition.

128- Angle formed between neural spine and dorsal margin of Pu3: less than 45° (0), more than 45° (1) ([4]: ch. 109, [7]: ch. 105, [8]: ch. 62, [10]: ch. 104, [20]: ch. 87, [22]: ch. 101, [33]: ch. 37, [36]: ch. 46, [81]: ch. 17)

129- Relative length of neural spine on Pu2: shorter than neural spine on Pu3 (0), as long as neural spine on Pu3 (1) ([1]: ch. 122, [3]: ch. 1, [4]: ch. 110, [6]: ch. 61, [7]: ch. 106, [8]: ch. 63, [20]: ch. 88, [22]: ch. 101, [24]: ch. 52, [33]: ch. 38, [36]: ch. 47, [81]: ch. 19)

130- Anterior processes on caudal preural neural and haemal arches: absent (0), present (1) (modified from [14]: ch. 3, [81]: ch. 14)

Commonly, the neural and haemal arches of the caudal fin of basal teleosts have small anteriorly projecting processes, at the base of the corresponding spines in some caudal preural centra (generally from Pu5 to Pu2). These processes are relatively short and do not reach the neural or haemal spine of the preceding preural centrum. Such processes are present in basal teleosts (except for pycnodontids, pachycormiforms, and aspdorhynchiforms), such as Jurassic ichthyodectiforms (e.g. †*Allothrissops*, †*Thrissops*; [31],[88]), varasichthyids (e.g. †*Protoclupea*, †*Domeykos*; [93]), the Late Jurassic †*Tharsis* *dubius*, †*Ascalabos* *voithii*, and †*Cavenderichthys* *talbragarensis* ([20],[81], pers. obs.), and the Cretaceous †*Notelops* [30]. Anterior processes on preural neural and haemal arches are not widely distributed among teleocephalans and, within our taxon sample, are only present in the extant osteoglossomorph *Hiodon* [15] and in the fossil euteleost genera †*Leptolepides* and †*Orthogonikleithrus* ([20],[81], pers.obs.). Anteriorly projected processes of this kind have been also observed in the caudal fin of †*Luisiella* *feruglioi* [73], whereas they are absent in amiids and lepisosteoids.

The presence of anterior processes of neural and haemal arches was also coded by Arratia [81] and Grande and Bemis [14]. Arratia’s character considers three states regarding the presence of elongate and truncate anterior processes versus short, more or less rounded or sharp processes, or the absence of these processes. Grande and Bemis [14] proposed a different character, to code the absence/presence of anteriorly projecting spine-like processes on neural and /or haemal arches. According to our observations, it is difficult to determine the shape of these processes and, thus, we have opted to score the presence-absence of the elements only.

131- Neural arch on Pu1: present and well developed (0), absent or greatly reduced (1) ([40]: ch. 52)

132- Neural spine on Pu1: absent (0), present and short (1), present and long, close to, or reaching the dorsal margin of the body (2) ([1]: ch. 123, [4]: ch. 111, [7]: ch. 107, [8]: ch. 64, [10]: ch. 106, [20]: ch. 89, [22]: ch. 103, [33]: ch. 39, [36]: ch. 48, [81]: ch. 20)

133- Condition of preural haemal arches (excluding Pu1) in relation to their respective centra: unfused (0), laterally fused (1), medially fused, laterally unfused (2) ([81]: ch. 12)

Although we have taken this character directly from Arratia, we replaced the term “autogenous” with “unfused” because of the developing connotation of the former.

134- Relationship between PH and Pu1: unfused (0), laterally fused (1) medially fused, laterally unfused (2) ([4]: ch. 105, [7]: ch. 101, [8]: ch. 60, [10]: ch. 100, [20]: ch. 83, [22]: ch. 97, [33]: ch. 34, [36]: ch. 42)

As in the previous character, we replaced the term “autogenous” with “unfused”.

135- Hypurapophysis: absent (0), present (1) ([4]: ch. 106, [7]: ch. 102, [8]: ch. 61, [10]: ch. 101, [20]: ch. 84, [22]: ch. 98, [24]: ch. 50, [33]: ch. 35, [36]: ch. 43)

136- Number of ural centra: three or more (0), two (1), none (2) ([1]: ch. 127)

137- Neural arch on U1: present (0), absent (1) ([20]: ch. 92, [36]: ch. 51, [40]: ch. 52, [81]: ch. 23)

138- Neural spine on U1: present (0), absent (1) ([1]: ch. 125, [20]: ch. 91, [33]: ch. 40, [36]: ch. 50, [81]: ch. 22)

139- Uroneural cartilage: absent (0), present (1) ([4]: ch. 116, [7]: ch. 112, [8]: ch. 69, [10]: ch. 111, [22]: ch. 108)

140- Compound cartilaginous neural arch over Pu1 and U1: absent (0), present (1) ([1]: ch. 128, [4]: ch. 117, [7]: ch. 113, [10]: ch. 112, [20]: ch. 94, [22]: ch. 109, [33]: ch. 42, [36]: ch. 53, [94]: ch. 4)

141- Origin of ural centra: from an expansion of ventral arcualia (occasionally also dorsal arcualia) (0), from an expansion of dorsal arcualia only (1) (slightly modified from [4]: ch. 118, [7]: ch. 114, [10]: ch. 113, [22]: ch. 110)

The character was proposed by Arratia [4] and used in subsequent analyses [7],[10],[22] to code the presence/absence of ural centra formed by the expansion of the dorsal arcualia (basidorsal) only, which is a unique feature of *Lepisosteus* [95]. We have slightly modified the original enunciation of the character to define the alternative condition. The ural centra of living teleosts and *Amia* *calva* originate from expansions of the ventral arcualia (basiventral). Even though the dorsal arcualia has a brief participation in the formation of the ural centra of *Amia* and some teleosts, like the euteleost *Thymallus*, the centra are mainly formed by the ventral arcualia [83],[95].

142- Number of epurals: four or more (0), three (1), two (2), one (3), none (4) ([22]: ch. 111)

143- Number of uroneurals: seven or more (0), six (1), five or four (2), three or less (3), none (4) ([4]: ch. 124, [7]: ch. 119, [8]: ch. 76, [10]: ch. 118, [22]: ch. 113)

144- Arrangement of uroneural bones: forming a uniform horizontally inclined series (0), forming two series in different angles (1) ([1]: ch. 134, [5]: ch. 80)

145- Anterior extent of first uroneural: Pu4 or Pu3 (0), Pu2 (1), Pu1 (2), U1 or U2 (3) (modified from [4]: ch. 129, [5]: ch. 82, [7]: ch. 124, [8]: ch. 80, [10]: ch. 122, [20]: ch. 103, [22]: ch. 116, [23]: ch. 21, [24]: ch. 57, [26]: ch. 65, [33]: ch. 50, [36]: ch. 61, [81]: ch. 42)

According to Arratia [4],[10],[20],[36],[81] and our own observations, in the caudal skeleton of basal teleosts the first uroneural generally extends forward up to the Pu3 and Pu4, like in †*Ascalabos*, †*Allothrissops*, †*Luisichthys*, †*Protoclupea.* Less frequently, the first uroneural reaches only up to the Pu2 (e.g. †*Pachythrissops*, †*Leptolepis* *koonwarri*, †*Cavenderichthys* *talbragarensis*; [20],[62]). The last condition is also present in †*Luisiella* *feruglioi*, some elopomorphs, and Jurassic euteleosts among teleocephalans. In living teleosts, the first uroneural is even shorter and reaches up to the Pu1 (e.g. †*Diplomystus*, *Esox*, *Brycon*, *Chanos*). More exceptionally, the first uroneural of the basal teleosts †*Dorsetichthys* *bechei*, †*Aspidorhynchus* and †*Belonostomus* extends laterally over the U1 or even U2 but does not reach any preural centra [16],[81],[96].

This character was modified from those proposed previously to include all the observed variation of the anterior extension of the first uroneural. Many authors only coded our states (0), (1), and (2) [4],[5],[20],[23],[24],[26],[33],[36],[81], or included our state (4), but also scoring the existence of uroneurals, which was already coded in ch. 143 (e.g. [7],[8],[10],[22]).

146- Number or uroneurals extending forward beyond U2: three or four (0), two (1), one (2) (modified from [1]: ch. 135, [4]: ch. 130, [7]: ch. 125, [8]: ch. 81, [10]: ch. 123, [20]: ch. 104, [22]: ch. 117, [31]: ch. 40, [33]: ch. 51, [36]: ch. 62, [40]: ch. 46, [81]: ch. 45)

Patterson [40] and Patterson and Rosen [31] first referred to the condition of two uroneurals extending rostrally beyond the U2 as a synapomorphy of Elopocephala (sensu Patterson and Rosen [31]), which included the living groups Elopomorpha, Clupeomorpha, and Euteleostei. The authors distinguished this condition from that present in osteoglossomorphs, where there are three or even four uroneurals extending rostrally beyond the U2. According to Arratia [10],[20] and our observations, there are also only two uroneurals extending beyond the U2 in the varasichthyid †*Protoclupea*, and the Cretaceous †*Notelops*, whereas most basal teleosts including †*Luisiella* *feruglioi* and the aspidorhynchiforms †*Aspidorhynchus* and †*Belonostomus* [16] have three or more uroneurals extending beyond the U2 (e.g. †*Leptolepis* *coryphaenoides*, †*Tharsis* *dubius*, ichthyodectiforms). In some clupeomorphs and euteleosts, like *Denticeps*, †*Diplomystus*, *Oncorhynchus*, *Esox* [20],[31] only one uroneural extends beyond the U2.

A character dealing with the occurrence of only two uroneurals, rather than three or four, extending forward beyond the U2 was used by many authors (e.g. [1],[4],[7],[8],[20],[31][33],[36],[40]), although without scoring other conditions (i.e. our states (0) and (2)). A somewhat different character definition was given by Arratia [81] and Arratia and Tischlinger [22], who included as states other possible conditions for this character (e.g. two or one uroneural extending forward beyond U2, stegural extending forward beyond U2). Our character is based essentially on these latter character definitions, and was modified in order to include all the observed conditions for the trait.

147- Relative position of anterior uroneurals in relation to ural and preural centra: placed dorsolaterally to the centra, not covering their lateral surfaces (0), placed laterally, covering the lateral surfaces of centra (1) (modified from [21]: ch. 4, [22]: ch. 193, [23]: ch. 5)

In most teleosts the uroneurals are placed dorsolaterally to the preural and ural vertebrae of the vertebral column and, thus, the lateral surfaces of these centra are exposed and visible. This condition occurs in †*Luisiella* *feruglioi*, in most basal teleosts, like †*Tharsis* *dubius*, †*Varasichthys,* and †*Cavenderichthys* *talbragarensis*, as well as in extinct and extant teleocephalans, like †*Anaethalion*, †*Leptolepides*, †*Lycoptera*, *Brycon*, *Chanos*, and *Engraulis*, among many others [11],[13],[20],[49]. In particular, the uroneurals of the extinct order †Ichthyodectiformes (e.g. †*Allothrissops*, †*Thrissops*, †*Cladocyclus*) are placed laterally to the preural and ural vertebrae of the caudal skeleton and, therefore, the lateral surfaces of the centra are not exposed, as they are covered by these ossifications [21],[23],[31]. Although this latter condition has been proposed as a unique synapomorphy of †Ichthyodectiformes by Patterson and Rosen [31], it also occurs in the Cretaceous non-ichthyodectiform teleosts †*Apsopelix* and †*Crossognathus* [34],[64].

Maisey [21] and later Alvarado-Ortega [23] proposed a character to code the presence or absence of anterior uroneurals extending anteroventrally over the sides of preural centra (the latter author referred to the uroneurals covering the lateral surfaces of preural centra). Although it is similar to our state (1), their character definition did not include a reference to the covering of the ural centra, which are also laterally covered. In contrast, Arratia and Tischlinger [22] referred to both preural and ural centra in their character definition of the same condition (i.e. uroneurals covering the lateral surface of centra). We have modified these previously used characters in order to describe both observed placements of the uroneurals with respect to the preural and ural centra.

148- Pleurostyle: absent (0), present (1) ([4]: ch. 141, [6]: ch. 63, [7]: ch. 132, [10]: ch. 129, [20]: ch. 118, [33]: ch. 63, [36]: ch. 76)

149- Stegural: absent (0), present (1) ([4]: ch. 139, [6]: ch. 65, [7]: ch. 134, [10]: ch. 130, [20]: ch. 116, [22]: ch. 124, [33]: ch. 62, [36]: ch. 75, [81]: ch. 54)

150- Uroneural-like bones (modified preural neural arches): absent (0), present (1) ([1]: ch. 130, [4]: ch. 123, [7]: ch. 118, [8]: ch. 75, [10]: ch. 117, [22]: ch. 112)

151- Number of hypurals: eight or more (0), seven (1), six or less (2), all hypurals fused in an hypural plate (3) ([22]: ch. 118)

152- Relationship between H1 and H2, and ural centra: H1 and H2 supported by a single centrum (0), H1 and H2 supported by two different centra (1) ([16]: ch. 39, [81]: ch. 9)

153- Relationship between H1 and H2 and their corresponding centra (a): laterally unfused to the centrum (a) (0), only H2 fused (1) both hypurals laterally fused to the centrum (a) (2) (modified from [4]: ch. 134, [7]: ch. 127, [10]: ch. 125, [20]: chs. 111 and 112, [22]: ch. 119, [24]: ch. 63, [33]: chs. 58 and 59, [36]: chs. 69 and 70, [81]: chs. 35 and 36)

In the outgroup taxa and in most basal teleosts like †*Dorsetichthys* *bechei*, †*Protoclupea*, ichthyodectiforms, among others, as well as in the majority of non-clupeomorph teleocephalans H1 and H2 are unfused (at least laterally) to the U1. This is also the condition present in the caudal endoskeleton of †*Luisiella* *feruglioi*. Less frequently, both hypurals are laterally fused to the U1, as it occurs in †*Leptolepis* *coryphaenoides*, †*Rhacolepis*, †*Notelops*, †*Crossognathus*, and †*Bavarichthys* [10],[22],[30],[81]. In clupeomorphs (e.g. †*Diplomystus*, *Engraulis*, this analysis) only H2 is fused to U1, whereas H1 is fused to H2 but not to U1 [29],[31]. Outside clupeomorphs, the condition of having only H2 fused to U1 has been described only in some ostariophysans characiforms. Patterson and Rosen [31] and Grande [29] proposed this last condition as a synapomorphy of the Clupeomorpha.

The present character merges two different characters proposed initially by Arratia [20],[33],[36],[81] that we consider that are not independent from one another (i.e. only H2 fused with U1; both H1 and H2 laterally unfused or laterally fused to ural centra 1 and 2 or U1). In subsequent phylogenetic analyses Arratia [4],[7],[10],[22] included only one of these characters. A different character incorporating our states (0) and (2) was proposed by Cavin [24].

154- Relationship between H1 and H2: independent from each other (0), fused at their bases only (1), fused to each other along their entire length (2) ([22]: ch. 120, [24]: ch. 64)

155- Relationship between Pu1 and U1: independent from each other (0), fused, forming a compound centrum (1) ([3]: ch. 48, [50]: ch. 50)

156- A space or diastema between H2 and H3: absent (0), present (1) ([1]: ch. 141, [20]: ch. 114)

157- Number of caudal fin rays articulating with each hypural: one (0), two or more (1) ([14]: ch. 46)

158- Dorsal scute(s) preceding caudal fin: present (0), absent (1) ([4]: ch. 156, [6]: ch. 57, [7]: ch. 149, [8]: ch. 99, [10]: ch. 145, [20]: ch. 130, [22]: ch. 139, [33]: ch. 73, [36]: ch. 87)

159- Epaxial basal fulcra: present (0), absent (1) ([1]: ch. 143, [22]: ch. 129)

160- Hipaxial basal fulcra: present (0), absent (1) ([1]: ch. 144)

161- Epaxial procurrent rays: absent (0), present (1) ([1]: ch. 146)

162- Number of fringing fulcra on the first caudal fin ray: numerous (0), one to five (1), none (2) ([22]: ch. 128)

163- Number of principal caudal fin rays: 20 or more (0), 19 (1), 18 or less (2) ([4]: ch. 148, [7]: ch. 141, [8]: ch. 92, [10]: ch. 138, [20]: ch. 123, [22]: ch. 132, [33]: ch. 68, [36]: ch. 81, [81]: ch. 64)

164- Branched rays on the caudal fin: 17 or more (0), 16 (1), 15 or fewer (2) ([3]: ch. 7, [6]: ch. 74, [9]: ch. 65)

165- Number of principal rays in lower lobe of caudal fin: ten or more (0), nine (1), eight or less (2) ([20]: ch. 124, [33]: ch. 69, [36]: ch. 82)

166- Number of caudal fin rays forming the dorsal and ventral leading margin of the fin: two or more (branched and unbranched) (0), one unbranched caudal fin ray (1), one branched caudal fin ray (modified from [1]: ch. 149)

In most teleosts, such as †*Cavenderichthys* *talbragarensis*, †*Allothrissops* *mesogaster*, *Elops* *saurus*, *Brycon* *meeki*, among many others, one long dorsal and one long ventral unbranched ray (principal ray) forms the leading margin of the caudal fin. This typical teleostean caudal fin configuration, which is also present in †*Luisiella* *feruglioi*, has been proposed by Arratia [90] as a synapomorphy of teleosts. However, in some basal teleosts (†*Eurycormus* *speciosus*, †*Pholidophorus* *gervasutti*, aspidorhynchiforms, and pachycormiforms) as well as in *Amia* the leading margin of the fin is formed by two or more (branched and unbranched) rays, which delineate the caudal fin outline. On the other hand, only one branched ray leads the caudal fin margin of *Lepisosteus*.

This character was modified from the analisis by Arratia [1], who coded the presence/absence of first and last principal caudal rays forming the leading margins of the caudal fin, which is our state (1) to include all the different caudal fin leading margin configurations we found in our taxon sample.

167- Arrangement of dorsal principal rays of caudal fin and dorsal hypurals: dorsal principal rays located oblique to main axis of hypurals (0), dorsal principal rays parallel to main axis of hypurals so that no fin-ray overlies more than one hypural (1) ([20]: ch. 125, [24]: ch. 67, [33]: ch. 70, [36]: ch. 83)

168- Dorsal processes of the bases of innermost principal caudal fin rays of upper lobe: absent (0), present (1) ([1]: ch. 150, [4]: ch. 152, [7]: ch. 145, [8]: ch. 95, [10]: ch. 141, [20]: ch. 126, [22]: ch. 126, [33]: ch. 71, [36]: ch. 84, [81]: ch. 66)

169- Relative shape of the bases of innermost principal caudal fin rays: narrow, with rounded anterior margin (0), expanded, with crenulated anterior margin (1) (modified from [81]: ch. 65)

The bases of the innermost principal rays of the caudal fin of teleocephalans are generally narrow and have a rounded anterior margin, as observed for example in †*Anaethalion*, †*Diplomystus*, *Hiodon*, *Oncorhynchus*, †*Santanaclupea* [4],[11],[15],[37],[50]. However, in more basal teleosts (e.g. †*Allothrissops*, †*Tharsis*, †*Cavenderichthys*, †*Domeykos,* pachycormids ([12],[20],[81], pers. obs.), as well as in a few teleocephalans, like *Elops* and †*Orthogonikleithrus* (this analysis), the bases of the innermost principal caudal rays are expanded into fan-like shapes and have crenulated anterior margins. The latter is the condition present in †*Luisiella* *feruglioi*.

A somewhat similar character was proposed by Arratia [81] although it coded different attributes (i.e. length and shape) of the bases of the innermost principal rays by means of three different states (i.e. long, broadly expanded bases and commonly with crenulated anterior margins; elongated bases and moderately or slightly expanded; short and narrow bases). Since length and shape are independent traits, we limited the character to code the shape of the bases of these caudal rays and, therefore, modified the character accordingly.

170- Type of segmentation of marginal principal caudal rays: “Z” or step-like (0), straight (1) ([22]: ch. 137)

171- Relationship between last principal caudal ray and preural haemal spines: last principal caudal ray in contact with PH (0), last principal caudal ray in contact with haemal spine of Pu2 (1), last principal caudal ray in contact with haemal spines of more anterior preural centra (i.e. Pu3 to Pu8) (2)

The relationship between the last principal caudal fin ray and the haemal spine of Pu2 has been proposed by Schultze and Arratia [95] and later by Arratia [90] as one of the landmarks to recognize the teleostean homocercal tail (“true teleosts” caudal fin). Accordingly, in most teleosts (basal teleosts as well as teleocephalans) the haemal spine of Pu2 supports the lowermost principal caudal ray, like in †*Leptolepis* *coryphaenoides*, †*Ascalabos*, †*Domeykos*, †Aspidorhynchiformes, †*Anaethalion*, †*Diplomystus*, *Elops*, *Esox*, *Hiodon* [11],[15],[16],[20],[27],[35],[81],[90],[96], and also in †*Luisiella* *feruglioi*. However, the last principal caudal fin ray is supported by the parhypural in some anguilliform-bodied teleosts [95], as well as in the Cretaceous †*Notelops*, †*Rhacolepis*, †*Santanaclupea,* and †*Tharrhias* [21],[30],[37] included in the present analysis. Generally in non-teleostean actinopterygians, the last principal caudal fin ray is supported by the haemal spine of some of the preural centra anterior to Pu2 (i.e. Pu3 to Pu8), a condition observed in *Lepisosteus*, †*Obaichthys*, and *Amia* species [14],[17].

172- Caudal fin margin: deeply forked (0); higher than long and unforked (1); convexly rounded (2) ([5]: ch. 76)

173- Urodermals in the caudal fin: absent (0), present (1) ([14]: ch. 13)

174- Number of ‘urodermals’ (i.e. tendon-bones), associated with dorsalmost principal caudal rays: two (0), one (1), none (2) ([1]: ch. 152)

**Scales**

175- Type of scales: ganoid of lepisosteoid type (0), elasmoid of cycloid type (1), elasmoid of amioid type (2) ([1]: ch. 156)

176- Shape of posterior margin of cycloid scales: smooth (0), crenulated (1) (slightly modified from [1]: ch. 158, [4]: ch. 161, [7]: ch. 154, [8]: ch. 104, [10]: ch. 150, [20]: ch. 132, [22]: ch. 144, [33]: ch. 75, [36]: ch. 89)

In most teleosts the posterior margins of the cycloid scales are smooth, as observed for example in the Jurassic †*Leptolepis* *coryphaenoides* and †*Tharsis* *dubius*, among basal teleosts, and in *Engraulis*, †*Humbertia*, †*Erichalcis*, *Heterotis*, and *Thymallus*, among many other teleocephalans [15],[20],[27],[36]. Similarly, the scales covering the trunk of †*Luisiella* *feruglioi* have smooth posterior margins. In contrast, in the Late Jurassic varasichthyids, †*Pachythrissops*, basal elopomorphs (e.g. †*Anaethalion*, *Elops*), and a few fossil euteleosts (e.g. †*Leptolepides* *sprattiformis*) the posterior margins of the scales are crenulated [20].

We have slightly modified the original enunciation of the character to define the alternative condition.

177- Type of ornamentation of cycloid scales: circuli or circuli and radii (0), circuli and transverse lines (1), circuli and squamulae (2) (modified from [1]: ch. 157, [4]: ch. 160, [7]: ch. 153, [8]: ch. 103, [10]: ch. 149, [20]: ch. 131, [22]: ch. 131, [33]: ch. 74, [36]: ch. 88)

Although the cycloid scales of teleosts have in general different types of ornamentation, in most teleost taxa the scales are ornamented with circuli only (concentric lines that follow the outline of the scale), or with circuli and radii (grooves that radiate from the focus to the scale margin) [97]. This is the condition present in †*Leptolepis* *coryphaenoides*, †*Tharsis*, †*Ascalabos* [20],[27],[89] and in elopomorphs, clupeomorphs, ostariophysans, and euteleosts among living groups of teleosts [20],[98]. The scales of †*Luisiella* *feruglioi* are ornamented only with circuli. However, members of the Jurassic family Varasichthyidae (e.g. †*Domeykos*, †*Varasichthys*, †*Protoclupea*) as well as the ichthyodectiform †*Allothrissops* have a distinct ornamentation pattern with transverse lines crossing the circuli in their middle field (i.e. between anterior and posterior fields; [10],[20],[33]). Exclusively in osteoglossomorphs, furrows (squamulae) form, together with the circuli, a network over the whole scale [98].

The occurrence of cycloid scales with circuli crossed by transverse lines (our state (1)) was coded as a binary presence-absence character by Arratia [1],[4],[7],[8],[10],[20],[33],[36] and Arratia and Tischlinger [22]. Besides, Arratia and Tischlinger [22] proposed a different character to score for the occurrence of reticulated scales (our state (2)) in osteoglossomorph taxa. Therefore, our character is the result of merging those previous characters because we consider the different types of ornamentation are not mutually independent.

178- Leptocephalous larva: absent (0), present (1) ([4]: ch. 162, [7]: ch. 155, [10]: ch. 153, [20]: ch. 133, [22]: ch. 145, [59]: ch. 268, [94]: ch. 6)

**References**

1. Arratia G. Morphology, taxonomy, and phylogeny of Triassic pholidophorid fishes (Actinopterygii, Teleostei). J Vert Pal. 2013;33 suppl 1:1-138.

2. De Pinna MCC. Teleostean monophyly. In: Stiassny MLJ, Parenti LD, Johnson GD, editors. Interrelationships of fishes*.* San Diego: Academic Press; 1996:147-162.

3. Li GQ, Wilson MVH. Phylogeny of Osteoglossomorpha. In: Stiassny MLJ, Parenti LD, Johnson GD, editors. Interrelationships of fishes*.* San Diego: Academic Press; 1996:163-174.

4. Arratia G. The monophyly of Teleostei and stem-group teleosts. Consensus and disagreements. In: Arratia G, Schultze HP, editors. Mesozoic Fishes 2 – Systematics and Fossil Record. München: Verlag Dr Friedrich Pfeil; 1999:265-334.

5. Grande T, Poyato-Ariza FJ. Phylogenetic relationships of fossil and recent gonorynchiform fishes (Teleostei: Ostariophysi). Zool J Linn Soc. 1999;125:197-238.

6. Li GQ, Wilson MVH. Early divergence of Hiodontiformes sensu stricto in East Asia and phylogeny of some Late Mesozoic teleosts from China. In: Arratia G, Schultze HP, editors. Mesozoic Fishes 2 – Systematics and Fossil Record. München: Verlag Dr Friedrich Pfeil; 1999:369–384.

7. Arratia G. Remarkable teleostean fishes from the Late Jurassic Southern Germany and their phylogenetic relationships. Mitt Mus Natkd Berl, Geowiss Reihe. 2000;3:137-179.

8. Arratia G. New teleostean fishes from the Jurassic of southern Germany and the systematics problems concerning the “pholidophoriforms”. Paläontol Z. 2000;74:113-143.

9. Hilton EJ. Comparative osteology and phylogenetic systematics of fossil and living bony-tongue fishes (Actinopterygii, Teleostei, Osteoglossomorpha). Zool J Linn Soc. 2003;137:1-100.

10. Arratia G. The varasichthyid and other crossognathiform fishes, and the break-up of Pangaea. In: Cavin L, Longbottom A, Richter M, editors. Fishes and the break-up of Pangaea: Special Publication 295. London: Geological Society; 2008:71-92.

11. Arratia G. *Anaethalion* and similar teleosts (Actinopterygii, Pisces) from the Late Jurassic (Tithonian) of southern Germany and their relationships. Palaeontographica A. 1987;200:1-44.

12. Mainwaring AJ. Anatomical and systematic revision of the Pachycormidae, a family of Mesozoic fossil fishes*.* Westfield College; 1978. [PhD Thesis].

13. Weitzman SH. The osteology of *Brycon meeki*, a generalized characid fish, with an osteological definition of the family. Stanford Icthyol Bull. 1962;8:1-77.

14. Grande L, Bemis WE. A comprehensive phylogenetic study of amiid fishes (Amiidae) based on comparative skeletal anatomy: an empirical search for interconected patterns of natural history. J Vert Pal, suppl 1. 1998;18:1-690.

15. Hilton EJ. Osteology of the extant North American fishes of the genus *Hiodon* Lesueur, 1818 (Teleostei: Osteoglossomorpha: Hiodontiformes). Fieldiana *(*Zoology) New Series. 2002;100:1-142.

16. Brito P. Révision des Aspidorhynchidae (Pisces, Actinopterygii) du Mésozoïque: ostéologie, relations phylogénétiques, données environnementales et biogéographiques. Geodiversitas. 1997;19:681-672.

17. Grande L. An empirical synthetic pattern study of gars (Lepisosteiformes) and closely related species, based mostly on skeletal anatomy. The resurrection of Holostei. Copeia*,* suppl 10. 2010:1-871.

18. Kriwet J. A comprehensive study of Pycnodont fishes (Neopterygii, Pycnodontiformes). Humboldt Universität; 2001. [PhD Thesis].

19. Gardiner BG, Schaeffer B. Interrelationships of lower actinopterigyan fishes. Zool J Linn Soc. 1989;97:135-187.

20. Arratia G. Basal teleosts and teleostean phylogeny. Palaeo Ichthyologica. 1997;7:5-168.

21. Maisey JG. Santana Fossils: an illustrated atlas. Neptune: TFH Publications Inc; 1991.

22. Arratia G, Tischlinger H. The first record of Late Jurassic crossognathiform fishes from Europe and their phylogenetic importance for teleostean phylogeny. Foss Rec. 2010;13:317-341.

23. Alvarado-Ortega J. Description and relationships of a new Ichthyodectiform fish from the Tlayúa Formation (Early Cretaceous: Albian), Puebla, Mexico. J Vert Pal. 2004;24:502-813.

24. Cavin L. Osteology and phylogenetic relationships of the teleost *Goulmimichthys arambourgi* Cavin 1995, from the Upper Cretaceous of Goulmima, Morocco. Eclogae Geol Helv. 2001;94:509-535.

25. Stewart JD. A new genus of Saurodontidae (Teleostei: Icthyodectiformes) from Upper Cretaceous rocks of the Western Interior of North America. In: Arratia G, Schultze HP, editors. Mesozoic Fishes 2 – Systematics and Fossil Record. München: Verlag Dr Friedrich Pfeil; 1999:335-360.

26. Cavin L, Forey PL, Giersch S. Osteology of *Eubiodectes libanicus* (Pictet & Humbert, 1866) and some other ichthyodectiformes (Teleostei): phylogenetic implications. J Syst Paleont. 2012;11:1-63.

27. Nybelin O. A revision of the leptolepid fishes. Acta Reg Soc Sci Litt Goth Zool. 1974;9:1-202.

28. Bean LB. The leptolepid fish *Cavenderichthys talbragarensis* (Woodward, 1895) from the Talbragar Fish Bed (Late Jurassic) near Gulgong, New South Wales. Rec Aust Mus. 2006;23:43-76.

29. Grande L. Recent and fossil clupeomorph fishes with materials for revision of the subgroups of clupeoids. Bull Am Mus Nat Hist. 1985;181:231-372.

30. Forey PL. The osteology of *Notelops* Woodward, *Rhacolepis* Agassiz and *Pachyrhizodus* Dixon (Pisces: Teleostei). Bull Brit Mus (Nat Hist) Geol. 1977;28:125-204.

31. Patterson C, Rosen DE. Review of the ichthyodectiform and other Mesozoic fishes and the theory and practice of classifying fossils. Bull Am Mus Nat Hist. 1977;158:81-172.

32. Arratia G, Schultze HP. *Eurycormus* – *Eurypoma*, two Jurassic actinopterygians genera with mixed identity. Fossil Record. 2007;10:17-37.

33. Arratia G. Phylogenetic and paleogeographic relationships of the varasichthyid group (Teleostei) from the Late Jurassic of Central and South America. Rev Geol Chile. 1994;21:119-165.

34. Taverne L. *Crossognathus* Pictet, 1858 du Cretacé inférieur de l’Europe et systématique, paleozoogeographie et biologie des Crossognathiformes nov. ord. (Téléostéens) du Cretacé et du Tertiare. Palaeontographica A. 1989;207:79-105.

35. Taverne L. L'ostéologie d'*Elops* Linné, C., 1766 (Pisces, Elopiformes) et son intérêt phylogénétique. Mémoires de la Classe des Sciences Collection. 1974;8:1-96.

36. Arratia G. Reassessment of the phylogenetic interrelationships of certain Jurassic teleosts and their implications on teleostean phylogeny. In: Arratia G, Viohl G, editors. Mesozoic fishes – Systematics and paleoecology. München: Verlag Dr Friedrich Pfeil; 1996:219-242.

37. Maisey JG. A new clupeomorph fish from the Santana Formation (Albian) of NE Brazil. Am Mus Novit. 1993;3076:1-16.

38. Greenwood PH, Rosen DE, Weitzman SH, Myers GS. Phyletic studies of teleostean fishes, with a provisional classification of living forms. Bull Am Mus Nat Hist. 1966;131:339-455.

39. Forey PL. A fossil clupeomorph fish from the Albian of the Northwest Territories of Canada, with notes on cladistic relationships of clupeomorphs. J Zool. 1975;175: 151-177.

40. Patterson C. The contribution of paleontology to teleostean phylogeny. In: Hecht MK, Goody PC, Hecht BM, editors. Major patterns in vertebrate evolution. New York: Plenum Press; 1977:579-643.

41. Patterson C. The braincase of pholidophorid and leptolepid fishes, with a review of the actinopterygian braincase. Phil Trans R Soc Lond. 1975;269:275–597.

42. Ridewood WG. On the cranial osteology of the fishes of the families Elopidae and Albulidae, with remarks on the morphology of the skull in the lower teleostean fishes generally. Proc Zool Soc Lond. 1904;1904:35-81.

43. Greenwood PH. On the genus *Lycoptera* and its relationship with the family Hiodontidae (Pisces, Osteoglossomorpha). Bull Brit Mus (Nat Hist) Zool. 1970;19:257-285.

44. Taverne L. Ostéologie, phylogénèse et systématique des Téléostéens fossiles et actuels du super-ordre des Ostéoglossomorphes. Acad Roy Belgique Cl Sci Collect. 1977;42:1-234.

45. Taverne L. Les ostéoglossomorphes marins de l’Éocène du Monte Bolca (Italie): *Monopteros* Volta 1796, *Thrissopterus* Heckel, 1856 et *Foreyichthys* Taverne, 1979. Considérations sur la phylogénie des téléostéens ostéoglossomorphes. Studie e Ricerche sui Giacimenti Terziari di Bolca. 1998;7:67-158.

46. Cavin L, Forey PL. Osteology and systematic affinities of *Palaeonotopterus greenwoodi* Forey 1997 (Teleostei: Osteoglossomorpha). Zool J Linn Soc. 2001;133:25-52.

47. Forey PL. Relationships of elopomorphs. In: Greenwood PH, Miles RS, Patterson C, editors. Interrelationships of Fishes. Zool J Linn Soc Suppl 1; 1973:351-368.

48. Patterson C. Two Upper Cretaceous salmoniform fishes from the Lebanon. Bull Brit Mus (Nat Hist) Geol. 1970;19:205-296.

49. Fink SV, Fink WL. Interrelationships of the ostariophysan fishes (Teleostei). Zool J Linn Soc. 1981;72:297-353.

50. Sanford CPJ. Salmonoid fish osteology and phylogeny (Teleostei: Salmonoidei). Ruggell/Liechtenstein: ARG Gantner*.* 2000.

51. Taverne L. Position systématique et relations phylogénétiques de *Paraclupavus (“Leptolepis”) caheni*, téléostéen marin du Jurassique moyen de Kisangani (Calcaires de Songa, Étage de Stanleyville), République Démocratique du Congo. Mus Roy Afr Centr, Tervuren (Belg), Dépt Géol Min Rapp Ann 1999-2000. 2001:55-76.

52. Brito PM. L’endocrâne et la moulage endocrânienne de *Vinctifer comptoni* (Actinopterygii, Aspidorhynchiformes) du Crétacé inferieur du Brésil. Ann Paléontol. 1992;78:129-157.

53. Hurley IA, Mueller RL, Dunn KA, Schmidt KJ, Friedman M, Ho RK, Prince VE, Yang Z, Thomas MG, Coates MI. A new time-scale for ray-finned fish evolution. Proceedings B. 2007;274:489-498.

54. Allis EP. The myodome and trigemino-facialis chamber of fishes and the corresponding cavities in higher vertebrates. J Morphol. 1919;32:207-326.

55. Gardiner BG. The relationships of the palaeoniscid fishes, a review based on new specimens of *Mimia* and *Moythomasia* from the Upper Devonian of Western Australia. Bull Brit Mus (Nat Hist) Geol. 1985;37:173-428.

56. Goodrich ES. Studies on the structure and development of Vertebrates. London: Macmillan and Co; 1930.

57. Grande L. The first *Esox* (Esocidae: Teleostei) from the Eocene Green River Formation, and a brief review of esocid fishes. J Vert Pal. 1999;19:271-292.

58. Wilson MVH, Veilleux P. Comparative osteology and relationships of the Umbridae (Pisces: Salmoniformes). Zool J Linn Soc. 1982;76:321-352.

59. Diogo R, Doadrio I, Vandewalle P. Teleostean phylogeny based on osteological and myological characters. Int J Morphol. 2008;26:463-522.

60. Wiley EO. The phylogeny and biogeography of fossil and Recent gars (Actinopteryii: Lepisosteidae). Misc publ Univ Kans Mus Nat Hist. 1976;64:1-111.

61. López-Arbarello A. Phylogenetic interrelationships of ginglymodian Fishes

(Actinopterygii: Neopterygii). Plos One. 2012;7:e39370.

62. Waldman M. Fish from the freshwater Lower Cretaceous of Victoria, Australia with comments on the palaeo-environment. Spec Pap Palaeontol. 1971;9:1-62.

63. Nelson GJ. Infraorbital bones and their bearing on the phylogeny and geography of osteoglossomorph fishes. Am Mus Novit. 1969;2394:1-37.

64. Teller-Marshall S, Bardack D. The morphology and relationships of the Cretaceous teleost *Apsopelix*. Fieldiana. 1978;41:1-35.

65. McAllister DE. The evolution of branchiostegal rays in teleostome fishes. University of British Columbia; 1964. [PhD Thesis].

66. Bardack D. Anatomy and evolution of chirocentrid fishes. Univ Kans Paleontol Contrib Vertebr. 1965;10:1-88.

67. Arratia G, Schultze HP. The urohyal: development and homology within osteichthyans. J Morphol. 1990;203:247-282.

68. Patterson C. Interrelationships of holosteans. In: Greenwood PH, Miles RS, Patterson C, editors. Interrelationships of Fishes. Zool J Linn Soc Suppl 1; 1973:233-305.

69. Patterson C. Family Chanidae and other teleostean fishes as living fossils. In: Eldredge N, Stanley SM, editors. Living Fossils. New York: Springer-Verlag; 1984: 132-139.

70. Poyato-Ariza FJ. A new Cretaceous gonorynchiform fish (Teleostei: Ostariophysi) from Las Hoyas (Cuenca, Spain). Occ Pap Mus Nat Hist Univ Kansas. 1994;164:1-37.

71. López-Arbarello A, Schröder KM. The species of *Aspidorhynchus* Agassiz, 1833 (Neopterygii, Aspidorhynchiformes) from the Jurassic plattenkalks of Southern Germany. Paläontol Z. 2014;88:167-185.

72. Nelson GJ. Cephalic sensory canals, pitlines, and the classification of esocoid fishes, with notes on galaxiids and other teleosts. Am Mus Novit. 1972;2492:1-50.

73. Sferco E, López-Arbarello A, Báez AM. Anatomical description and taxonomy of †*Luisiella* *feruglioi* (Bordas) new combination, a freshwater teleost (Actinopterygii, Teleostei) from the Upper Jurassic of Patagonia. J Vert Pal. 2015;DOI 10.1080/02724634.2014.924958.

74. Lecointre G, Nelson G. Clupeomorpha, sister-group of Ostariophysii. In: Stiassny MLJ, Parenti LD, Johnson GD, editors. Interrelationships of fishes*.* San Diego: Academic Press; 1996:193-207.

75. Nelson GJ. Origin and diversification of teleostean fishes. Ann New York Acad Sci. 1969;167:18-30.

76. Rosen DE. Teleostean interrelationships, morphological function and evolutionary inference. Amer Zool. 1982;22:261-273.

77. Greenwood PH. The osteology and relationships of the Denticipitidae, a family of clupeomorph fishes. Bull Brit Mus (Nat Hist) Zool. 1968;16:213-273.

78. Lauder GV, Liem KF. The evolution and interrrelationships of the actinopterygian fishes. Bull. Mus. Comp. Zool. 1983;150:95-197.

79. Fink SV, Fink WL. Interrelationships of ostariophysan fishes (Teleostei). In: Stiassny MLJ, Parenti LD, Johnson GD, editors. Interrelationships of fishes*.* San Diego: Academic Press; 1996:209-249.

80. Schaeffer B. Osteichthyan vertebrae. Zool J Linn Soc. 1967;47:185-195.

81. Arratia G. The caudal skeleton of Jurassic teleosts: A phylogenetic analysis. In: Chang MM, Liu YH, Zhang, GR, editors. Early vertebrates and related problems in evolutionary biology. Beijing: Science Press; 1991:249-340.

82. Rosen DE, Greenwood PH. Origin of the Weberian apparatus and the relationships of the ostariophysan and gonorvnchiform fishes. Am Mus Novit. 1970;2428:1-25.

83. Arratia G, Schultze HP. Reevaluation of the caudal skeleton of certain actinopterygian fishes. III. Salmonidae. Homologization of caudal skeletal structures. J Morphol. 1992;214:187-249.

84. Mirande M. Phylogeny of the family Characidae (Teleostei: Characiformes): from characters to taxonomy. Neotrop Ichthyol*.* 2010;8:385-568.

85. Patterson C, Johnson GD. The intermuscular bones and ligaments of teleostean fishes. Smithson Contrib Zool. 1995;559:1-85.

86. Patterson C. Cartilage bones, dermal bones, and membrane bones, or the exoskeleton versus the endoskeleton. In: Andrews SM, Miles RS, Walker AD, editors. Problems in vertebrate evolution. London: Academic Press; 1977:77-121.

87. Jessen H. Schultergurtel und Pectoralflosse bei Actinopterygiern. Fossils and Strata. 1972;1:1-101.

88. Taverne L. Ostéologie et position systématique du genre *Thrissops* Agassiz, 1833 (sensu stricto) (Jurassique Supérieur de l'Europe Occidentale) au sein des téléostéens primitifs. Geobios. 1977;10:5-33.

89. Wenz S. Compléments a l’étude des poisons actinoptérygiens du Jurassique française. Cahiers de Paléontologie. 1968:1-276.

90. Arratia G. Actinopterygian postcranial skeleton with special reference to the diversity of fin ray elements, and the problem of identifying homologies. In: Arratia G, Schultze HP, Wilson MVH, editors. Mesozoic Fishes 4 – Homology and Phylogeny. München: Verlag Dr. Friedrich Pfeil; 2008:49-101.

91. Taverne L. Ostéologie, phylogénèse et systématique des Téléostéens fossiles et actuels du super-ordre des Ostéoglossomorphes. Mém Acad Roy Cl Sci Collect 8º, 2e series. 1979;1-175.

92. Taverne L. Considérations sur la position systématique des genres fossils *Leptolepis* et *Allothrissops* au sein des Téléostéens primitives et sur lórige et le polyphylétisme des Poissons Téléostéens. Acad Roy Belgique. 1975;5:336-371.

93. Arratia G, Schultze HP. Late Jurassic teleosts (Actinopterygii, Pisces) from Northern Chile and Cuba. Palaeontogr Abt A. 1985;189:29-61.

94. Filleul A. Analyse critique des synapomorphies des Elopomorphes et analyse phylogénétique du groupe. Cybium. 2000;24(3) suppl:75-83.

95. Schultze HP, Arratia G. The composition of the caudal skeleton of teleosts (Actinopterygii: Osteichthyes). Zool J Linn Soc. 1989;97:189-231.

96. Patterson C. The caudal skeleton in Lower Liassic pholidophorid fishes. Bull Brit Mus (Nat Hist) Geol. 1968;16:201-239.

97. Schultze HP. The scales of Mesozoic actinopterygians. In: Arratia G, Viohl G, editors. Mesozoic fishes – Systematics and paleoecology. München: Verlag Dr Friedrich Pfeil; 1996:243-259.

98. Meunier FJ, Brito PM. Histology and morphology of the scales in some extinct and extant teleosts. Cybium. 2004;28:225-235.
