# Supplementary material for: Phylogenetic relationships of †Luisiella feruglioi (Bordas) and the recognition of a new clade of freshwater teleosts from the Jurassic of Gondwana
Source: BMC Evol Biol. 2015 Dec 3;15:268. doi: 10.1186/s12862-015-0551-6 (PMC4668602; doi:10.1186/s12862-015-0551-6)
Supplement: Additional file 2: — List of studied specimens and consulted literature. (DOCX 19 kb) [file 12862_2015_551_MOESM2_ESM.docx]

LIST OF EXAMINED MATERIAL AND CONSULTED LITERATURE

**Institutional abbreviations**

BSPG**,** Bayerische Staatssammlung für Paläontologie und Geologie, Munich, Germany; CPBA-V, Vertebrate Paleontology Collection, Universidad de Buenos Aires, Argentina; JME-ETT, Jura-Museum Eischttät, Germany (Ettling); JME-SOS, Jura-Museum Eischttät, Germany (Eischttät); MACN, Museo Argentino de Ciencias Naturales “Bernardino Rivadavia”, Buenos Aires, Argentina; MB. f. Museum für Naturkunde, Berlín, Germany; MLP, Museo de La Plata, La Plata, Argentina; MPEF-PV, Museo Paleontológico Egidio Feruglio – Colección de Paleovertebrados, Trelew, Argentina; NHMUK, Natural History Museum, London, UK; TRF, Helmut Tischlinger, private collection, Germany; UBA, Universidad de Buenos Aires, Vertebrate Collection, Buenos Aires, Argentina.

†*Allothrissops mesogaster*: [1]-[6].

*Amia calva*: UBA P.670; [7].

†*Amia pattersoni*: [7].

†*Anaethalion angustus*: [3]-[6],[8]-[11].

†*Anaethalion* *knorri*: JME-SOS 2267a; JME-SOS 2282; JME-SOS 2270a; JME-SOS 2281; JME-SOS 2280; BSPG 1960 XVIII 93; BSPG 1964 XXIII 394, BSPG 1969 XVI 31; BSPG 1969 XI 39; BSPG 1968 XV 129; 1991 I 122; BSPG 1968 XV 3; BSPG AS I 1473; [3]-[6],[8]-[10].

†*Apsopelix anglicus*: [12].

†*Ascalabos* *voithii*: JME-SOS 537, JME-SOS 2496, JME-SOS 3015, JME-SOS 2996, JME-SOS 2891, JME-SOS 2364, JME-SOS 2458 114 4; JME-SOS 2497a, JME-SOS 3230 114 1; [3]-[5],[10],[13],[14].

†*Aspidorhynchus acutirostris*: BSPG AS V 509, BSPG AS VII 1107, BSPG AS VII 1109, BSPG 1954 I 230, BSPG 1960 VIII 46, BSPG 1960 XVIII 59, BSPG 1976 I 17, BSPG 1988 I 22; [15]-[17].

†*Bavarichthys incognitus*: [18].

†*Belonostomus tenuirostris*: BSPG AS I 1207, BSPG 1961 I 212; [16].

*Brycon meeki*: [19],[20].

†*Cavenderichthys talbragarensis*: MB.f. 4917, MB.f. 5001, MB.f. 5006, MB.f. 5004, MB.f. 5005, MB.f. 5002, MB.f. 5003. NHMUK 12425, NHMUK 12451, NHMUK 12438, NHMUK 12428, NHMUK 12439, NHMUK 12426, NHMUK 37977, NHMUK 12450, NHMUK 12444, NHMUK 12441, NHMUK 12443, NHMUK 12424, NHMUK 12427, NHMUK 12445, NHMUK 12446, NHMUK 12424, NHMUK 12427, NHMUK 12437, NHMUK 12434, NHMUK 12442, NHMUK 12432, NHMUK 37975, NHMUK 12449, NHMUK 12435, NHMUK 12431, NHMUK 37973, NHMUK 37974, NHMUK 12430, NHMUK 12429, NHMUK 37976, NHMUK 12436, NHMUK 18113, NHMUK 12448. BSPG 1939 X 321, TRF 68, TRF 60, TRF 61, TRF 64; [5],[21],[22].

*Chanos chanos*: [5],[10],[23]-[25].

†*Chongichthys dentatus*: [5],[26],[27].

†*Crossognathus sabaudians*: [3],[18],[28],[29].

*Denticeps clupeoides*: [5],[10],[30],[31].

†*Diplomystus dentatus*: BSPG 1889 I 18, BSPG 1889 I 503, BSPG 1957 I 128, BSPG 2008 I 81; [3]-[6],[10],[31],[32].

†*Domeykos profetaensis*: [4]-[6],[29],[33],[34].

†*Dorsetichthys* *bechei*: [35]-[37].

†*Erichalcis arcta*: [5],[6],[10],[31],[38].

*Elops saurus*: [5],[6],[9],[10],[39]-[41].

*Engraulis ringens*: [5],[6].

*Esox lucius*: [5],[6],[42],[43].

†*Eurycormus* *speciosus*: BSPG AS I 1275, BSPG AS V 510, BSPG 1960 XVIII 61, BSPG 1960 XVIII 106, JME-SOS 2339, JME-SOS 2341, JME-SOS 4614a-b, JME-SOS 4615; [44],[45].

†*Gordichthys conquensis*: [46],[47].

†*Goulmimichthys arambourgi*: [48].

*Heterotis niloticus*: [49]-[51].

*Hiodon alosoides*: [50]-[54].

†*Humbertia operta*: [55].

†*Hypsocormus macrodon*: [56]-[58].

*Lepisosteus osseus*: UBA P.671; [59]-[62].

†*Leptolepis coryphaenoides*: [3]-[5],[10],[13],[37].

†*Leptolepis* *koonwarri*: [63].

†*Leptolepides haertesi*: JME-SOS 2473, JME-SOS 2471, JME-SOS 2554; [5].

†*Leptolepides sprattiformis*: CPBA-V-282, JME-SOS 2305, JME-SOS 2422, JME-SOS 2463, JME-SOS 2472, JME-SOS 2956; [3]-[5],[10],[13],[64].

†*Luisichthys vinalesensis*: [29],[33],[34].

†*Luisiella feruglioi*: MACN 13026-13056, 13058-13069, 14430, 14431, 14435, MLP 35-III-1-4, MLP 67-III-1-1 a MLP 67-III-1-15, MLP 67-III-1-17 to MLP 67-III-1-21, MLP 67-III-1-23 to MLP 67-III-1-26, MLP 67-III-1-29, MLP 67-III-1-30, MLP 67-III-1-32 to MLP 67-III-1-35, MLP 67-III-1-37, MLP 67-III-1-40 to MLP 67-III-1-45, MLP 67-III-1-49 to MLP 67-III-1-53, MLP 67-III-1-56 to MLP 67-III-1-60, MLP 67-III-1-63, MLP 67-III-1-65, MLP 67-III-1-66, MLP 67-III-1-68, MLP 67-III-1-70 a MLP 67-III-1-74, MLP 76- X-7-16/2, MLP 92-XI-30-1 to MLP 92-XI-30-8, 89-XI-30-1, MPEF-PV 1354, 1385, 1388, 1389, 1469#3-5, 1469#7-10, 1470#2, 1470#3, 1470#5, 1471, 1472#3, 1472#4, 1472#6, 1472#8-10, 1473, 1474#1-8, 1475#5, 1476#1-4, 1476#6-8, 1476#11, 1477#4, 1477#6, 1477#7, 1478#1, 1478#3-6, 1478#8, 1480, 1481, 1482, 1483, 1484#1, 1484#2, 1484#5, 1484#7-9, 1484#11, 1485#3-5, 1485#7, 1485#8, 1486 A-B, 1487#1, 1487#2, 1487#4-7, 1487#10, 1488#1-8, 1489#2-5, 1489#9, 1491#1, 1491#2, 1491#4, 1491#6, 1492#3, 1493#5, 1494#1, 1494#3, 1608, 1611, 1654, 1768, 1769, 3096, 3097, 3170, 3185, 3186, 3192, 3193, 3195, 3196, 3198, 3200, 3202, 3411-3415, 3417-3421, 3426, 3428, 3429, 3431, 3432, 3837, 3904, 3905, 3908, 3909, 3911, 3912, 3930-3939, 3942-3949, 3950-3953, 3956, 3957, 3960, 3962-3967, 3977, 3979-3981, 3983-3987. 3422-3425, 3427, 3846-3849, 3850-3856, 3858-3861, 3864-3873, 3876, 3877, 3879-3885, 3887, 3910, 3927, 3940, 3954, 3968, 3988, 3989. 3433, 3875, 3888, 3891, 3974, 3906, 3928, 3941, 3955, 3959, 3961, 3969, 3982, 3986, 10534, 10535; [65].

†*Lycoptera middendorffi*: [5],[10],[50],[51],[53],[66],[67].

*Megalops atlanticus*: [5],[6],[10],[68].

†*Mesturus verrucosus*: BSPG AS V 508; [69],[70].

†*Notelops brama*: [71],[72].

†*Obaichthys decoratus*: [61],[62],[73].

*Oncorhynchus mykiss*: [74].

*Opsariichthys bidens*: [20],[23],[68].

†*Orthogonikleithrus* *leichi*: JME-SOS 2301, JME-SOS 2632; [5].

†*Orthogonikleithrus* *hoelli*: JME-SOS 3954, JME-SOS 3955, JME-SOS 3957, JME-SOS 3961, JME-SOS 3964; [5],[75].

†*Pachycormus macropterus*: [56]-[58].

†*Pachythrissops propterus*: BSPG 1964 XXIII 154; [5].

†*Pholidophorus* *gervasuttii*: [45].

†*Pholidophorus* *latiusculus*: [35],[36],[45].

†*Protoclupea chilensis*: [4],[29],[33],[34].

†*Rhacolepis buccalis*: BSPG 1967 I 159, BSPG 1972 I 16, BSPG 1972 I 17, BSPG 1975 I 167, BSPG 1989 XII 209; [71],[72].

†*Santanaclupea silvasantosi*: [76].

†*Siemensichthys* *macrocephalus*: [37],[45],[77].

†Siemensichthys siemensi: [45],[77].

†*Tharrhias araripis*: [25],[72],[78].

†*Tharsis* *dubius*: BSPG 1986 XV 125; BSPG 1953 I 9; BSPG 1953 I 97; BSPG 1954 I 226; BSPG 1964 XXIII 395; BSPG 1964 XXIII 399; BSPG 1964 XXIII 407; BSPG 1964 XXIII 415; BSPG 1964 XXIII 417; BSPG 1964 XXIII 414, BSPG 1964 XXIII 430; BSPG 1964 XXIII 280; BSPG 1964 XXIII 160, BSPG 1986 I 41, BSPG 1986 I 37; BSPG 1960 XVIII 90; BSPG 1960 XVIII 91; BSPG 1960 XVIII 97; BSPG 1960 XVIII 98, BSPG 1961 III 23, BSPG 1961 I 466; BSPG 1968 XV 29; BSPG 1969 XVI 32, BSPG 1969 XVI 39; BSPG 1994 V 5; BSPG AS VI 25; MB.f. 15685, MB.f. 2831, CPBA-V-1383, CPBA-V-1502, CPBA-V-1505; JME-SOS 3899; [3],[4],[10][13],[37].

†*Thrissops formosus*: JME-ETT 103, JME-ETT 126, JME-ETT 887, JME-ETT 972, JME-ETT 1805a-b, JME-ETT 3371a, JME-ETT 3476; [1],[79].

*Thymallus thymallus*: [74].

*Umbra krameri*: [80].

†*Varasicthys ariasi*: [34],[29].

†*Vinctifer comptoni*: BSPG 1971 I 169a-b, BSPG 1972 I 14, BSPG 1972 I 28, CPBA-V-14066. [72],[81],[82].

**References**

1. Nybelin O. Versuch einer taxonomischen Revision der Jurassischen Fishgattung *Thrissops* Agassiz. Meddel Göteb Mus Zoolog Avdeln 1964;135:1-33.

2. Taverne L. Considérations sur la position systématique des genres fossils *Leptolepis* et *Allothrissops* au sein des Téléostéens primitives et sur lórige et le polyphylétisme des Poissons Téléostéens. Acad Roy Belgique. 1975;5:336-371.

3. Patterson C, Rosen DE. Review of the ichthyodectiform and other Mesozoic fishes and the theory and practice of classifying fossils. Bull Am Mus Nat Hist. 1977;158:81-172.

4. Arratia G. The caudal skeleton of Jurassic teleosts: A phylogenetic analysis. In: Chang MM, Liu YH, Zhang, GR, editors. Early vertebrates and related problems in evolutionary biology. Beijing: Science Press; 1991:249-340.

5. Arratia G. Basal teleosts and teleostean phylogeny. Palaeo Ichthyologica. 1997;7:5-168.

6. Arratia G. The monophyly of Teleostei and stem-group teleosts. Consensus and disagreements. In: Arratia G, Schultze HP, editors. Mesozoic Fishes 2 – Systematics and Fossil Record. München: Verlag Dr Friedrich Pfeil; 1999:265-334.

7. Grande L, Bemis WE. A comprehensive phylogenetic study of amiid fishes (Amiidae) based on comparative skeletal anatomy: an empirical search for interconected patterns of natural history. J Vert Pal, suppl 1. 1998;18:1-690.

8. Gaudant J. Contribution à une revision des *Anaethalion* de Cerin (Ain). Bull Bur Rech geól miner Paris. 1968;4:95-115.

9. Arratia G. *Anaethalion* and similar teleosts (Actinopterygii, Pisces) from the Late Jurassic (Tithonian) of southern Germany and their relationships. Palaeontographica A. 1987;200:1-44.

10. Arratia G. Reassessment of the phylogenetic interrelationships of certain Jurassic teleosts and their implications on teleostean phylogeny. In: Arratia G, Viohl G, editors. Mesozoic fishes – Systematics and paleoecology. München: Verlag Dr Friedrich Pfeil; 1996:219-242.

11. Poyato-Ariza FJ. The elopiform fish *Anaethalion* *angustus* restored, with comments on individual variation. In: Arratia G, Schultze HP, editors. Mesozoic Fishes 2 – Systematics and Fossil Record. München: Verlag Dr Friedrich Pfeil; 1999:361-368.

12. Teller-Marshall S, Bardack D. The morphology and relationships of the Cretaceous teleost *Apsopelix*. Fieldiana. 1978;41:1-35.

13. Nybelin O. A revision of the leptolepid fishes. Acta Reg Soc Sci Litt Goth Zool. 1974;9:1-202.

14. Taverne L. Sur *Leptolepis* (*Ascalabos*) *voithi* (von Münster, G., 1839), teleosteen fossile du jurassique superieur d leurope et ses affinites systematiques. Biol Jaarb Dodonaea 1975;43:233-245.

15. Lehman JP. Actinopterygii. In: Piveteau J, editor. Traité de paleontologie. Paris: Masson et Cie; 1966:1-242.

16. Brito P. Révision des Aspidorhynchidae (Pisces, Actinopterygii) du Mésozoïque: ostéologie, relations phylogénétiques, données environnementales et biogéographiques. Geodiversitas. 1997;19:681-672.

17. López-Arbarello A, Schröder KM. The species of *Aspidorhynchus* Agassiz, 1833 (Neopterygii, Aspidorhynchiformes) from the Jurassic plattenkalks of Southern Germany. Paläontol Z. 2014;88:167-185.

18. Arratia G, Tischlinger H. The first record of Late Jurassic crossognathiform fishes from Europe and their phylogenetic importance for teleostean phylogeny. Foss Rec. 2010;13:317-341.

19. Weitzman SH. The osteology of *Brycon meeki*, a generalized characid fish, with an osteological definition of the family. Stanford Icthyol Bull. 1962;8:1-77.

20. Mirande M. Phylogeny of the family Characidae (Teleostei: Characiformes): from characters to taxonomy. Neotrop Ichthyol*.* 2010;8:385-568.

21. Cavender TM. A comparison of coregonines and other salmonids with the earliest known teleostean fishes. In: Linsey CC, Woods CS, editors. Biology of Coregonid Fishes. Winnipeg: University of Manitoba Press; 1970:1-32.

22. Bean LB. The leptolepid fish *Cavenderichthys talbragarensis* (Woodward, 1895) from the Talbragar Fish Bed (Late Jurassic) near Gulgong, New South Wales. Rec Aust Mus. 2006;23:43-76.

23. Fink SV, Fink WL. Interrelationships of the ostariophysan fishes (Teleostei). Zool J Linn Soc 1981;72:297-353.

24. Fink SV, Fink WL. Interrelationships of ostariophysan fishes (Teleostei). In: Stiassny MLJ, Parenti LD, Johnson GD, editors. Interrelationships of fishes*.* San Diego: Academic Press; 1996:209-249.

25. Patterson C. Family Chanidae and other teleostean fishes as living fossils. In: Eldredge N, Stanley SM, editors. Living Fossils. New York: Springer-Verlag; 1984: 132-139.

26. Arratia G. *Chongichthys dentatus*, new genus and species, from the Late Jurassic of Chile (Pisces: Teleostei: Chongichthyidae, New Family). J Vert Pal. 1982;2: 133-149.

27. Arratia G. New Jurassic fishes (Teleostei) of Cordillera de Domeyko, Northern Chile. Palaeontographica A 1986;192:75-91.

28. Taverne L. *Crossognathus* Pictet, 1858 du Cretacé inférieur de l’Europe et systématique, paleozoogeographie et biologie des Crossognathiformes nov. ord. (Téléostéens) du Cretacé et du Tertiare. Palaeontographica A. 1989;207:79-105.

29. Arratia G. The varasichthyid and other crossognathiform fishes, and the break-up of Pangaea. In: Cavin L, Longbottom A, Richter M, editors. Fishes and the break-up of Pangaea: Special Publication 295. London: Geological Society; 2008:71-92.

30. Greenwood PH. The osteology and relationships of the Denticipitidae, a family of clupeomorph fishes. Bull Brit Mus (Nat Hist) Zool. 1968;16:213-273.

31. Grande L. Recent and fossil clupeomorph fishes with materials for revision of the subgroups of clupeoids. Bull Am Mus Nat Hist. 1985;181:231-372.

32. Grande L. A revision of the fossil genus †*Diplomystus*, with comments on the interrelationships of clupeomorph fishes. Am Mus Novit. 1982;2728:1-34.

33. Arratia G, Schultze HP. Late Jurassic teleosts (Actinopterygii, Pisces) from Northern Chile and Cuba. Palaeontogr Abt A. 1985;189:29-61.

34. Arratia G. Phylogenetic and paleogeographic relationships of the varasichthyid group (Teleostei) from the Late Jurassic of Central and South America. Rev Geol Chile. 1994;21:119-165.

35. Nybelin O. On certain Triassic and Liassic representatives of the family Pholidophoridae s.str. Bull Brit Mus (Nat Hist) Geol. 1966;11:351-432.

36. Patterson C. The caudal skeleton in Lower Liassic pholidophorid fishes. Bull Brit Mus (Nat Hist) Geol. 1968;16:201-239.

37. Patterson C. The braincase of pholidophorid and leptolepid fishes, with a review of the actinopterygian braincase. Phil Trans R Soc Lond. 1975;269:275–597.

38. Forey PL. A fossil clupeomorph fish from the Albian of the Northwest Territories of Canada, with notes on cladistic relationships of clupeomorphs. J Zool. 1975;175: 151-177.

39. Nybelin O. On the caudal skeleton in *Elops* with remarks on other teleostean fishes. Acta Reg Soc Sci Litt Goth Zool. 1971;7:1-52.

40. Forey PL. Relationships of elopomorphs. In: Greenwood PH, Miles RS, Patterson C, editors. Interrelationships of Fishes. Zool J Linn Soc Suppl 1; 1973:351-368.

41. Taverne L. L'ostéologie d'*Elops* Linné, C., 1766 (Pisces, Elopiformes) et son intérêt phylogénétique. Mém Acad Roy Cl Sci Collect. 1974;8:1-96.

42. Wilson MVH. Oldest known *Esox* (Pisces: Esocidae), part of a new Paleocene teleost fauna from western Canada. Can J Earth Sci. 1980;17: 307-312.

43. Wilson MVH. Osteology of the Palaeocene Teleost *Esox tiemani*. Palaeontology 1984;27:597-608.

44. Arratia G, Schultze HP. *Eurycormus* – *Eurypoma*, two Jurassic actinopterygians genera with mixed identity. Foss Rec. 2007;10:17-37.

45. Arratia G. Morphology, taxonomy, and phylogeny of Triassic pholidophorid fishes (Actinopterygii, Teleostei). J Vert Pal. 2013;33 suppl 1:1-138.

46. Poyato-Ariza FJ. A new Cretaceous gonorynchiform fish (Teleostei: Ostariophysi) from Las Hoyas (Cuenca, Spain). Occ Pap Mus Nat Hist Univ Kansas. 1994;164:1-37.

47. Poyato-Ariza FJ. The phylogenetic relationships of *Rubiesichthys* *gregalis* and *Gordichthys* *conquesis* (Ostariophysi, Chanidae), from the Early Cretaceous of Spain. In: Arratia G, Viohl G, editors. Mesozoic fishes – Systematics and paleoecology. München: Verlag Dr Friedrich Pfeil; 1996:329-348.

48. Cavin L. Osteology and phylogenetic relationships of the teleost *Goulmimichthys arambourgi* Cavin 1995, from the Upper Cretaceous of Goulmima, Morocco. Eclogae Geol Helv. 2001;94:509-535.

49. Taverne L. Ostéologie, phylogénèse et systématique des Téléostéens fossiles et actuels du super-ordre des Ostéoglossomorphes. Acad Roy Belgique Cl Sci Collect. 1977;42:1-234.

50. Li GQ, Wilson MVH. Early divergence of Hiodontiformes sensu stricto in East Asia and phylogeny of some Late Mesozoic teleosts from China. In: Arratia G, Schultze HP, editors. Mesozoic Fishes 2 – Systematics and Fossil Record. München: Verlag Dr Friedrich Pfeil; 1999:369–384.

51. Hilton EJ. Comparative osteology and phylogenetic systematics of fossil and living bony-tongue fishes (Actinopterygii, Teleostei, Osteoglossomorpha). Zool J Linn Soc. 2003;137:1-100.

52. Greenwood PH. Interrelationships of osteoglossomorphs. In: Greenwood PH, Miles RS, Patterson C, editors. Interrelationships of Fishes. Zool J Linn Soc Suppl 1; 1973:307–332.

53. Li GQ, Wilson MVH. Phylogeny of Osteoglossomorpha. In: Stiassny MLJ, Parenti LD, Johnson GD, editors. Interrelationships of fishes*.* San Diego: Academic Press; 1996:163-174.

54. Hilton EJ. Osteology of the extant North American fishes of the genus *Hiodon* Lesueur, 1818 (Teleostei: Osteoglossomorpha: Hiodontiformes). Fieldiana *(*Zoology) New Series. 2002;100:1-142.

55. Patterson C. Two Upper Cretaceous salmoniform fishes from the Lebanon. Bull Brit Mus (Nat Hist) Geol. 1970;19:205-296.

56. Mainwaring AJ. Anatomical and systematic revision of the Pachycormidae, a family of Mesozoic fossil fishes*.* Westfield College; 1978. [PhD Thesis].

57. Arratia G, Lambers P. The caudal skeleton of pachycormiforms: parallel evolution. In: Arratia G, Viohl G, editors. Mesozoic fishes – Systematics and paleoecology. München: Verlag Dr Friedrich Pfeil; 1996:191-218.

58. Liston JJ. A fish fit for *Ozymandias*?: the ecology, growth and osteology of *Leedsichthys* (Pachycormidae, Actinopterygii). University of Glasgow; 2006. [PhD Thesis].

59. Regan CT. The skeleton of *Lepidosteus*, with remarks on the origin and evolution of the lower neopterygian fishes. Proc Zool Soc Lond. 1923;1923:445-461.

60. Wiley EO. The phylogeny and biogeography of fossil and Recent gars (Actinopteryii: Lepisosteidae). Misc publ Univ Kans Mus Nat Hist. 1976;64:1-111.

61. Grande L. An empirical synthetic pattern study of gars (Lepisosteiformes) and closely related species, based mostly on skeletal anatomy. The resurrection of Holostei. Copeia*,* suppl 10. 2010:1-871.

62. López-Arbarello A. Phylogenetic interrelationships of ginglymodian Fishes

(Actinopterygii: Neopterygii). Plos One. 2012;7:e39370.

63. Waldman M. Fish from the freshwater Lower Cretaceous of Victoria, Australia with comments on the palaeo-environment. Spec Pap Palaeontol. 1971;9:1-62.

64. Taverne L. Osteólogie et affinities systemmatiques de Leptolepides sprattiformis (Pisces, Teleostei) du Jurassique Superieur de l’ Europe. Ann Soc Royal Zool Belgique. 1981;110:7-28.

65. Sferco E, López-Arbarello A, Báez AM. Anatomical description and taxonomy of †*Luisiella* *feruglioi* (Bordas) new combination, a freshwater teleost (Actinopterygii, Teleostei) from the Upper Jurassic of Patagonia. J Vert Pal. 2015;DOI 10.1080/02724634.2014.924958.

66. Gaudant J. Recherches sur l'anatomie et la position systematique du genre *Lycoptera* (poisson Teleosteen). Mem Soc Geol France (Nouvelle Série). 1968;109:1-41.

67. Greenwood PH. On the genus *Lycoptera* and its relationship with the family Hiodontidae (Pisces, Osteoglossomorpha). Bull Brit Mus (Nat Hist) Zool. 1970;19:257-285.

68. Nelson GJ. Relationships of clupeomorphs, with remarks on the structure of the lower jaws in fishes. In: Greenwood PH, Miles RS, Patterson C, editors. Interrelationships of Fishes. Zool J Linn Soc Suppl 1; 1973:333-349.

69. Nursall JR. The phylogeny of pycnodont fishes. The scales of Mesozoic actinopterygians. In: Arratia G, Viohl G, editors. Mesozoic fishes – Systematics and paleoecology. München: Verlag Dr Friedrich Pfeil; 1996:125-152.

70. Kriwet J. A comprehensive study of Pycnodont fishes (Neopterygii, Pycnodontiformes). Humboldt Universität; 2001. [PhD Thesis].

71. Forey PL. The osteology of *Notelops* Woodward, *Rhacolepis* Agassiz and *Pachyrhizodus* Dixon (Pisces: Teleostei). Bull Brit Mus (Nat Hist) Geol. 1977;28:125-204.

72. Maisey JG. Santana Fossils: an illustrated atlas. Neptune: TFH Publications Inc; 1991.

73. Wenz S, Brito PM. New data about lepisosteids and semionotids from the Early Cretaceous of Chapada do Araripe (NE Brazil): Phylogenetic implications. In: Arratia G, Viohl G, editors. Mesozoic fishes – Systematics and paleoecology. München: Verlag Dr Friedrich Pfeil; 1996:153-165.

74. Sanford CPJ. Salmonoid fish osteology and phylogeny (Teleostei: Salmonoidei). Ruggell/Liechtenstein: ARG Gantner*.* 2000.

75. Ebert M, Koelbl-Ebert M. Raeuber-Beute-Beziehungen bei *Orthogonikleithrus* *hoelli* Arratia 1997. Archaeopteryx. 2008;26:11-18.

76. Maisey JG. A new clupeomorph fish from the Santana Formation (Albian) of NE Brazil. Am Mus Novit. 1993;3076:1-16.

77. Arratia G. Remarkable teleostean fishes from the Late Jurassic Southern Germany and their phylogenetic relationships. Mitt Mus Natkd Berl, Geowiss Reihe. 2000;3:137-179.

78. Oliveira AF. O gênero *Tharrhias* no Cretáceo da Chapada do Araripe. Anais Acad Brasil Ci. 1978;50:537-552.

79. Taverne L. Ostéologie et position systématique du genre *Thrissops* Agassiz, 1833 (sensu stricto) (Jurassique Supérieur de l'Europe Occidentale) au sein des téléostéens primitifs. Geobios 1977;10:5-33.

80. Wilson MVH, Veilleux P. Comparative osteology and relationships of the Umbridae (Pisces: Salmoniformes). Zool J Linn Soc. 1982;76:321-352.

81. Brito PM. La structure du suspensorium de *Vinctifer*, poisson actinoptérygien mésozoïque: remarques sur les implications phylogénétiques. Geobios. 1988;21:819-823.

82. Brito PM. L’endocrâne et la moulage endocrânienne de *Vinctifer comptoni* (Actinopterygii, Aspidorhynchiformes) du Crétacé inferieur du Brésil. Ann Paléontol. 1992;78:129-157.
