# Supplementary material for: Phylogenetic relationships of †Luisiella feruglioi (Bordas) and the recognition of a new clade of freshwater teleosts from the Jurassic of Gondwana
Source: BMC Evol Biol. 2015 Dec 3;15:268. doi: 10.1186/s12862-015-0551-6 (PMC4668602; doi:10.1186/s12862-015-0551-6)
Supplement: Additional file 3: — List of characters, data matrix, most parsimonious trees and strict consensus tree. (PDF 389 kb) [file 12862_2015_551_MOESM3_ESM.pdf]

List of Characters

Character name	character states	
1. Suture between left and right frontal bones	0	smooth (harmonic suture)
	1	serrated
2. Anterior margin of frontal	0	narrower than posterior margin
	1	of similar width or slightly wider than posterior margin
3. Shape of nasal bones	0	flat, approximately rectangular
	1	flat, triangular
	2	tubular but straight
	3	tubular and strongly curved
4. Relationship between left and right nasal bones	0	in contact along the midline
	1	separated
5. Supraoccipital bone	0	absent
	1	present
6. Supraoccipital crest	0	absent or poorly developed
	1	well-developed, spine-like supraoccipital crest
	2	well-developed, high and triangular supraoccipital crest
	3	hypertrophied, forming a pectinate blade
7. Condition of skull roof	0	medioparietal
	1	lateroparietal
8. Relative shape of the extraescapular bone/s	0	quadrangular or semicircular not expanded caudally
	1	tubular shape
	2	approximately triangular, expanded caudally
	3	expanded rostrally
9. Recessus lateralis	0	absent
	1	present
10. Ossified bulla in the prootic bone	0	absent
	1	present
11. Posttemporal fossa	0	absent
	1	present and separated from the fossa Bridgei
	2	present and confluent with the fossa Bridgei
12. Bones participating in the posttemporal fossa	0	epiotic, pterotic and exoccipital
	1	epiotic, pterotic, exoccipital and intercalar
	2	epiotic, pterotic and parietal
13. Position of the foramen for the glossopharyngeal nerve	0	in the prootic bone
	1	in the exoccipital bone
	2	in the basioccipital bone
14. Position of the foramen for the vagus nerve	0	between the intercalar and exoccipital bones
	1	in exoccipital bone
	2	in the basioccipital
	3	between the intercalar and basioccipital bones
15. Foramina in basioccipital for occipital or spinal arteries	0	present
	1	absent
16. Trajectory of the dorsal aorta in relation to the basioccipital	0	passing through an ossified canal formed by the basioccipital
	1	passing through a median groove in the basioccipital
	2	canal or groove in the surface of the basioccipital bone absent
17. Posterior myodome	0	extending in basioccipital
	1	not extending into basioccipital because it is confined to the prootics
	2	absent
18. Ethmopalatine ossifications	0	absent
	1	present
19. Proethmoid ossifications	0	absent
	1	present
20. Independent dermopalatine(s) bone(s)	0	present
	1	absent
21. Autopalatine bone	0	ossifies late in ontogeny
	1	ossifies early in ontogeny
	2	autopalatine bone absent
22. Vomer	0	paired

Character name		character states
	1	unpaired
23. Basisphenoid	0	present
	1	absent
24. Orbitosphenoid bone	0	reduced or absent
	1	present and large
25. Teeth on parasphenoid	0	absent
	1	present and small
	2	present and large
26. Posterior extent of parasphenoid	0	reaching the anterior margin of the basioccipital
	1	almost reaching the posterior margin of the basioccipital
	2	extending posterior to basioccipital
27. Basipterygoid process of parasphenoid	0	present
	1	absent
28. Separation between olfactory organ and eye	0	narrow
	1	broad
29. Shape of the most anterior infraorbital bone	0	laminar or plate-like
	1	tubular
30. Fourth and fifth infraorbital bones	0	separate
	1	fused
31. Circumorbital ring	0	incompletely closed
	1	completely closed ring, no space left between bones
32. Number of suborbital bone(s)	0	numerous
	1	two or three
	2	one
	3	none
33. Number of supraorbital bone(s)	0	two or more
	1	one
	2	none
34. Shape of the supraorbital bone (or anterior supraorbital bone)	0	not expanded
	1	expanded anteroventrally
35. Dermosphenotic	0	small
	1	large, well-developed bone
36. Independent antorbital bone	0	present
	1	absent
37. Antorbital bone	0	carrying a portion of the infraorbital canal
	1	without sensory canal
38. Shape of antorbital bone	0	approximately triangular
	1	rectangular
	2	tubular and comma-shaped
39. Posterior infraorbital bones	0	small and not overlapping or slightly overlapping the anterior margin of preopercle
	1	expanded posterior infraorbitals overlapping the anterior margin of preopercle
40. Hyomandibular bone with a preopercular process	0	absent
at its posterior margin	1	present
41. Elongation of suspensorium	0	absent
	1	present, due to a lengthening of the symplectic
	2	present, due to the occurrence of a cartilage between symplectic and hyomandibula
	3	present, due to the ventroposterior inclination of the hyomandibula
	4	present, due to the occurrence of the quadratojugal separating quadrate and sympletic
42. Hyomandibular articulation with the neurocranium	0	by means of one articular surface
	1	by means of two distinct articular surfaces
43. Elongate posteroventral process of quadrate	0	absent
	1	present
44. Shape of the dorsal margin of the quadrate	0	convex
	1	straight
	2	concave
	3	sinuous or sigmoid
	4	notched
45. Symplectic	0	articulates with lower jaw
	1	does not articulate with lower jaw

Character name		character states
46. Covering by orbital ossifications of palatoquadrate area	0	parcially
behind and below the orbit	1	totally
47. Shape of branchiostegal rays	0	variable, including acinaciform and spathiform branchiostegals
	1	uniform, only spathiform branchiostegal rays
48. Beryciform foramen in anterior ceratohyal	0	present
	1	absent
49. Number of hypohyal ossifications	0	one pair
	1	two pairs
50. Trajectory of the hyoidean artery in relation to the hypohyals	0	not piercing hipohyal
	1	piercing both dorsal and ventral hypohyals
	2	piercing only the ventral hypohyal
51. Urohyal formed as an unpaired tendon-bone	0	absent
	1	present
52. Gular plate	0	present
	1	absent
53. Suprapreopercle	0	absent
	1	present
54. Ventroposterior (excluding dorsal limb) region of preopercle	0	narrow or slightly expanded
	1	broadly expanded
55. Shape of preopercle	0	"L" shaped, with tapering ends
	1	crescent-shaped, long and narrow
	2	triangular
56. Interopercle	0	present
	1	absent
57. Anteroventral corner of the opercle	0	of 70°
	1	of more than 70°
58. Relative position of premaxillary bones	0	forming part of the tip of the snout, in contact with each other at the midline
	1	forming part of the tip of the snout but slightly separated by ethmoidal ossifications
	2	constituting the main element of an elongated snout, in contact with each other at the midline
	3	laterally placed and not forming part of the tip of the snout, separated by ethmoidal ossifications
59. Premaxillary ascending process	0	absent
	1	present
60. Maxillary teeth	0	present
	1	absent
61. Ventral margin of maxilla	0	straight or almost straight
	1	slightly convex
	2	slightly concave
62. Shape of the maxilla (excluding articular process)	0	uniformly deep
	1	approximately triangular
	2	bulbous
63. Shape of the posterior border of the maxilla	0	concave
	1	convex or straight
	2	acute
	3	notched
64. Number of supramaxillary bones	0	two
	1	one
	2	none
65. Relative position of supramaxilla(ae)	0	placed dorsal to the dorsal margin of maxilla
	1	placed posterodorsal to the maxilla
66. Quadrate-mandibular articulation	0	posterior to orbit
	1	placed below the posterior half of orbit
	2	placed below anterior half of orbit
	3	anterior to orbit
67. Predentary bone	0	absent
	1	present
68. Independent retroarticular bone	0	present
	1	absent
69. Retroarticular and quadrato-mandibular joint	0	retroarticular included in the joint facet for quadrate
	1	retroarticular excluded from the joint facet for quadrate

Character name		character states
70. Coronoid bone(s) in lower jaw	0	present
	1	absent
71. Surangular bone in lower jaw	0	present
	1	absent
72. Independent articular bone	0	present
	1	absent, fused with angular
	2	absent, fused with angular and retroarticular
73. Prearticular bone in lower jaw	0	present
	1	absent
74. Postarticular process of lower jaw	0	poorly developed
	1	well developed, extending posterior to the articular facet for quadrate
75. Characteristic notch (so-called leptolepid notch)	0	absent
in the anterodorsal ascending margin of the dentary	1	present
76. Marginal dentition on the jaws	0	uniform
	1	variable
77. Condition of cephalic sensory canals	0	with branched tubules
	1	with simple tubules
78. Cephalic sensory canal components	0	continuous
	1	interrupted between them
79. Middle pit line	0	leaving a groove or pore-line on the parietal and pterotic (or dermopterotic)
	1	leaving a groove or pore-line on the parietal only
	2	leaving no trace on the bones
80. Relative association of the ethmoidal commisure	0	not associated, there is no trace of an ethmoidal commissure
and ossifications of the ethmoidal region	1	the ethmoidal commissure is completely bone-enclosed in a canal
81. Relative position of supratemporal commisure	0	it pierces extrascapular bones
	1	it pierces parietal bones only, or parietals and supraoccipital
82. Parietal portion of supraorbital sensory canal	0	present
	1	absent
83. Composition of preopercular sensory canal	ß	four or less, short simple tubules
	1	seven or eight (up to 10), short simple or branched tubules
	2	12 or more long, simple or branched tubules
84. Mandibular canal	0	enclosed in bone along the whole lower jaw
	1	partially running in a tube bone and a groove
85. Endoskeletal basihyal	0	absent
	1	present
86. Covering of basibranchials 1 ? 3 by tooth plates	0	0 not covered, basibranchial tooth plates absent
	1	1 basibranchials overlain by paired distributed tooth plates
	2	2 basibranchials overlain by median tooth plates
87. Tooth plate(s) associated (attached, ankylosed or fused) to	0	present
pharyngobranchial 1	1	absent
88. Tooth plate(s) associated (attached or fused) to	0	present
pharyngobranchial 2	1	absent
89. Tooth plate(s) associated (attached or fused) to	0	present
pharyngobranchial 3	1	absent
90. Types of vertebral centra	0	amphicoelous
	1	opistocoelus
91. Composition of caudal vertebral centra	0	mineralized chordacentrum and arcocentra
	1	chordacentrum and basal part of arcocentra surrounded by autocentrum
	2	basal part of arcocentra surrounded by autocentrum
92. Relative condition of midcaudal vertebrae autocentrum	0	thin and smooth
	1	thick and sculptured
	2	thick and smooth
93. Relationship between autocentrum of midcaudal vertebrae	0	autocentrum not constricting or slightly constricting the notochord
and notochord	1	autocentrum strongly constricting the notochord
94. Relationship between abdominal neural arches and	0	unfused
vertebral centra	1	fused, except for the first five or six
	2	all abdominal neural arches fused
95. Relative condition of the neural arches of the anterior	0	separated from each other
abdominal vertebral centra (3 to 5)	1	in contact, abutting each other

Character name		character states
96. Number of neural spines in each abdominal vertebra	0	two
	1	one
97. Scaphium	0	absent
	1	present
98. Shape of proximal portion of first pleural rib	0	narrow
(on third vertebral centrum)	1	slightly expanded
	2	greatly expanded, forming the tripus
99. Relative shape of abdominal neural arches (lateral view)	0	tapering distally
	1	distally expanded, hourglass-shaped
100. Number of transitional vertebrae (between abdominal	0	four or more
and caudal regions)	1	three or less
101. Supraneural bones	0	absent
	1	present
102. Epineural bones	0	absent
	1	present
103. Epipleural bones	0	absent
	1	present
104. Position of epipleural bones relative to the column	0	located in anterior caudal region
	1	located in abdominal and anterior caudal region
105. Series of dorsal intermuscular bones in caudal region	0	absent
	1	present
106. Relationship between lateral line system and supracleithrum	0	emerging at its upper half
	1	emerging at its posteroventral margin
	2	lateral line not piercing the supracleithrum
107. Postsupracleitrum	0	absent
	1	present
108. Postcleitra	0	one to three
	1	four or more
	2	none
109. Relative position of coracoid bones	0	separated from each other at the midline
	1	contacting each other forming a midventral coracoid symphisys
110. Relationship between pectoral propterygium and first	0	unfused, propterygium free
pectoral ray	1	fused
111. Number of proximal pectoral radials	0	five or more
	1	four or less
112. Bifurcation pattern of pectoral fin rays	0	symmetric
	1	asymmetric, resulting in two unequal secondary rays
113. Pectoral axillary process	0	absent
	1	present
114. Pelvic axillary process	0	absent
	1	present
115. Fringing fulcra on pectoral fins	0	present
	1	absent
116. Abdominal scutes	0	absent
	1	present
117. Position of dorsal fin origin relative to the origin of the	0	posterior, located above boundary between abdominal and caudal regions
pelvic fin	1	posterior, located above caudal region
	2	anterior
118. Shape of dorsal fin margin	0	straight or slightly convex
	1	concave (acuminate dorsal fin)
119. Condition of first dorsal proximal pterygiophore	0	single structure
	1	bipartite or tripartite
120. Relationship between first dorsal proximal pterygiophore	0	it articulates with dorsal procurrent rays and first dorsal ray
and dorsal fin rays	1	it articulates with procurrent rays only
121. Fringing fulcra on leading margin of dorsal fin	0	present
	1	absent
122. Shape of anal fin margin	0	straight or slightly convex
	1	falcate, concave
123. Relative position of first anal proximal pterygiophore	0	placed anterior to fourth haemal spine in the transitional portion of the column

Character name		character states
	1	placed posterior to fourth haemal spine in the caudal portion of the column
124. Fringing fulcra on leading margin of anal fin	0	present
	1	absent
125. Number of vertebrae (including preural centrum 1)	0	less than 45
	1	45 to 65
	2	more than 65
126. Number of neural spines in each caudal vertebra	0	two
	1	one
127. Shape of neural spines from preural caudal vertebrae 3-5	0	narrow, without outgrowths
	1	distally expanded by anterior and posterior membranous outgrowths
128. Angle formed between neural spine and dorsal margin	0	less than 45°
of third preural vertebral centrum (Pu3)	1	more than 45°
129. Relative length of neural spine on second	0	shorter than neural spine on third preural centrum
preural centrum (Pu2)	1	as long as neural spine on third preural centrum
130. Anterior processes on caudal preural neural and	0	absent
haemal arches	1	present
131. Neural arch on preural centrum 1 (Pu1)	0	present and well developed
	1	absent or greatly reduced
132. Neural spine on preural centrum 1 (Pu1)	0	absent
	1	present and short
	2	present and long, close to, or reaching the dorsal margin of the body
133. Condition of preural haemal arches (excluding centrum Pu1)	0	unfused
in relation to their respective centra	1	laterally fused
	2	medially fused, laterally unfused
134. Relationship between parhypural and centrum Pu1	0	unfused
	1	laterally fused
	2	medially fused, laterally unfused
135. Hypurapophysis	0	absent
	1	present
136. Number of ural centra	0	three or more
	1	two
	2	none
137. Neural arch on first ural centrum	0	present
	1	absent
138. Neural spine on first ural centrum	0	present
	1	absent
139. Uroneural cartilage	0	absent
	1	present
140. Compound cartilaginous neural arch over preural centrum 1	0	absent
and first ural centrum	1	present
141. Origin of ural centra	0	from an expansion of ventral arcualia (occasionally dorsal arcualia too)
	1	from an expansion of dorsal arcualia only
142. Number of epurals	0	four or more
	1	three
	2	two
	3	one
	4	none
143. Number of uroneurals	0	seven or more
	1	six
	2	five or four
	3	three or less
	4	none
144. Arrangement of uroneural bones	0	forming a uniform horizontally inclined series
	1	forming two series in different angles
145. Anterior extent of first uroneural	0	preural centra 4 or 3
	1	preural centrum 2
	2	preural centrum 1
	3	centra U1 or U2
146. Number or uroneurals extending forward beyond ?second?	0	three or four

Character name		character states
ural centrum (U2)	1	two
	2	one
147. Relative position of anterior uroneurals in relation to ural	0	placed dorsolaterally to the centra, not covering their lateral surface
and preural centra	1	placed laterally, covering the lateral surface of centra
148. Pleurostyle	0	absent
	1	present
149. Stegural	0	absent
	1	present
150. Uroneural-like bones (modified preural neural arches)	0	absent
	1	present
151. Number of hypurals	0	eight or more
	1	seven
	2	six or less
	3	all hypurals fused in an hypural plate
152. Relationship between H1 and H2, and ural centra	0	first and second hypurals supported by a single centrum
	1	first and second hypurals supported by two different centra
153. Relationship between the first and second hypurals	0	laterally unfused to the centra
(H1 and H2, respectively) and their corresponding centrum(a)	1	only H2 fused
	2	both hypurals laterally fused to the centrum(a)
154. Relationship between H1 and H2	0	independent from each other
	1	fused at their bases only
	2	fused to each other along their length
155. Relationship between centra Pu1 and U1	0	independent from each other
	1	fused, forming a compound centrum
156. A space or diastema between H2 and H3	0	absent
	1	present
157. Number of caudal fin rays articulating with each hypural	0	one
	1	two or more
158. Dorsal scute(s) preceding caudal fin	0	present
	1	absent
159. Epaxial basal fulcra	0	present
	1	absent
160. Hipaxial basal fulcra	0	present
	1	absent
161. Epaxial procurrent rays	0	absent
	1	present
162. Number of fringing fulcra on the first caudal fin ray	0	numerous
	1	one to five
	2	none
163. Number of principal caudal fin rays	0	20 or more
	1	19
	2	18 or less
164. Branched rays on the caudal fin	0	17 or more
	1	16
	2	15 or fewer
165. Number of principal rays in lower lobe of caudal fin	0	ten or more
	1	nine
	2	eight or less
166. Number of caudal fin rays forming the dorsal and ventral	0	two or more (branched and unbranched)
leading margin of the fin	1	one unbranched caudal fin ray
	2	one branched caudal fin ray
167. Arrangement of dorsal principal rays of caudal fin and	0	dorsal principal rays located oblique to main axis of hypurals
dorsal hypurals	1	dorsal principal rays parallel to main axis of hypurals so that no fin-ray overlies more than one hypural
168. Dorsal processes of the bases of innermost principal	0	absent
caudal fin rays of upper lobe	1	present
169. Relative shape of the bases of innermost principal	0	narrow, with rounded anterior margin
caudal fin rays	1	expanded, with crenulated anterior margin
170. Type of segmentation of marginal principal caudal rays	0	Z or step-like
	1	straight

Character name		character states
171. Relationship between last principal caudal ray and	0	last principal caudal ray in contact with parhypural
preural haemal spines	1	last principal caudal ray in contact with haemal spine of preural centrum 2 (Pu2)
	2	last principal caudal ray in contact with haemal spines of more anterior preural centra
172. Caudal fin margin	0	deeply forked
	1	higher than long and unforked
	2	convexly rounded
173. Urodermals in the caudal fin	0	absent
	1	present
174. Number of ?urodermals?(i.e., tendon-bones), associated	0	two
with dorsalmost principal caudal rays	1	one
	2	none
175. Type of scales	0	ganoid of lepisosteoid type
	1	elasmoid of cycloid type
	2	elasmoid of amioid type
176. Shape of posterior margin of cycloid scales	0	smooth
	1	crenulate
177. Type of ornamentation of cycloid scales	0	circuli or circuli and radii
	1	circuli and transverse lines
	2	circuli and squamulae
178. Leptocephalous larva	0	absent
	1	present

Character matrix

Taxon / Character number	1	2	3	4	5	6	7	8	9	10	11	12	13	14	15
Amia_calva	0	1	0	0	0	-	0	0	0	0	2	?	-	0	0
Amia_pattersoni	0	1	0	1	0	-	0	0	0	0	?	?	-	0	?
Lepisosteus_osseus	0	0	0	0	0	-	0	0	0	0	0	-	0	0	0
Obaichthys_decoratus	0	0	0	0	0	-	0	0	0	?	?	?	?	0	?
Aspidorhynchus_acutirostris	?	0	0	1	1	0	0	0	0	0	1	?	0	0	?
Belonostomus_tenuirostris	-	0	0	1	1	0	0	0	0	0	1	?	0	0	?
Hypsocormus_macrodon	0	1	0	1	0	-	1	-	0	0	?	?	0	0	?
Mesturus_verrucosus	-	0	-	-	0	-	1	?	0	?	0	-	0	0	?
Pachycormus_macropterus	0	1	0	1	0	-	1	-	0	?	2	?	0	?	0
Vinctifer_comptoni	0	1	0	1	1	0	0	0	0	0	1	?	0	3	0
Apsopelix_anglicus	0	0	2	1	1	0	0	2	0	?	2	1	1	1	1
Allothrissops_mesogaster	0	0	2	1	1	0	0	0&1	0	?	2	0	1	1	?
Anaethalion_angustus	0	0	2	1	1	0	0	?	0	?	?	?	?	?	?
Anaethalion_knorri	0	0	2	1	1	0	0	0	0	?	?	?	?	?	?
Ascalabos_voithii	?	0	2	1	1	0	0	0	0	?	?	?	?	?	?
Bavarichthys_incognitus	?	0	?	?	1	0	0	2	0	?	?	?	?	?	?
Cavenderichthys_talbragarensis	0	0	2	1	1	0	0	0	0	?	?	?	?	?	?
Brycon_meeki	-	1	2	1	1	1	1	1	0	0	2	2	1	1	1
Chanos_chanos	0	0	2	1	1	3	1	1	0	0	2	2	1	1	1
Chongichthys_dentatus	0	?	?	?	1	0	?	?	0	?	?	?	1	1	?
Crossognathus_sabaudians	0	0	2	1	1	0	0	2	0	?	?	?	?	?	?
Denticeps_clupeoides	0	0	0	0	1	0	1	1	1	1	0	-	1	1	1
Diplomystus_dentatus	0	0	2	1	1	0	0	0&1	0	1	1&2	?	1	1	1
Domeykos_profetaensis	?	0	2	?	1	0	1	2	0	?	?	?	1	?	?
Dorsetichthys_bechei	0	0	0	1	0	-	0	0	0	0	1	?	0	0	0
Engraulis_ringens	?	0	2	?	1	0	1	1	1	1	0	-	1	1	1
Elops_saurus	0	0	2	1	1	0	0	0	0	0	2	0	1	1	1
Erichalcis_arcta	0	0	2	1	1	0	0	0&1	0	1	?	?	?	?	?
Esox_lucius	0	0	?	?	1	?	1	?	0	0	?	?	1	1	1
Eurycormus_speciosus	?	1	?	1	0	-	?	0	?	?	?	?	?	?	?
Gordichthys_conquensis	?	0	0	?	1	0	1	?	0	?	?	?	?	?	?

Taxon / Character number	1	2	3	4	5	6	7	8	9	10	11	12	13	14	15
Goulmimichthys_arambourgi	1	0	2	?	1	1	1	2	0	?	2	1	1	1	?
Hiodon_alosoides	1	1	3	1	1	2	0	3	0	0	?	?	1	1	1
Heterotis_niloticus	0	1	0	0	1	0	0	1	0	0	?	?	1	1	1
Humbertia_operta	0	0	0	1	1	0	0	0&1	0	?	2	2	1	1	1
Leptolepis_coryphaenoides	0	0	2	1	1	0	0	0	0	0	2	0	0	0	0
Leptolepis_koonwarri	0	0	2	1	1	0	0	0	?	?	?	?	?	?	?
Leptolepides_haerteisi	0	0	?	?	?	?	0	1	0	?	?	?	?	?	?
Leptolepides_sprattiformis	0	0	2	1	1	0	0	1	0	?	?	?	1	?	1
Luisichthysvinalaensis	1	0	2	?	1	0	0	2	0	?	2	1	1	1	1
Luisiella_feruglioi	0	0	2	1	1	0	0	0	?	?	?	?	?	?	?
Lycoptera_middendorfi	0	0	2	1	1	0	0	0	0	?	?	?	?	?	?
Megalops_atlanticus	0	0	2	1	1	0	0	0	0	0	2	0	1	1	1
Notelops_brama	1	0	2	1	1	1	0	2	0	?	2	1	1	1	?
Oncorhynchus_mykiss	0	0	2	1	1	0	?	1	0	0	2	2	1	1	1
Opsariichthys_bidens	1	?	0	?	1	0	0	?	0	0	2	2	1	1	1
Orthogonikleithrus_hoelli	?	0	?	?	1	?	0	?	0	?	?	?	?	?	?
Pachythrissops_propterus	?	0	?	?	1	0	?	0	0	?	?	?	?	?	?
Pholidophorus_gervasutti	0	?	0	1	0	-	0	?	?	?	?	?	?	?	?
Pholidophorus_latiusculus	?	?	?	1	?	?	0	?	?	?	?	?	?	?	?
Protoclupea_chilensis	0	0	?	1	1	?	0	2	0	?	?	?	?	?	?
Rhacolepis_buccalis	1	0	2	1	1	1	1	2	0	?	2	1	1	1	?
Santanaclupea_silvasantosi	0	0	?	?	1	0	1	?	1	1	?	?	2	2	?
Siemensichthys_macrocephalus	0	0	1	1	1	?	0	?	?	?	1	0	0	0	0
Siemensichthys_siemensi	0	0	?	1	?	?	?	?	?	?	?	?	?	?	?
Tharrhias_araripis_	0	0	2	1	1	3	1	1	0	?	?	?	?	?	?
Tharsis_dubius	0	0	2	1	1	0	0	0	0	0	2	0	1	0	1
Thrissops_formosus	?	0	2	?	1	2	0	1	0	?	?	?	?	?	?
Thymallus_thymallus	0	0	2	1	1	0	0	1	0	0	2	2	1	1	1
Umbra_krameri	0	0	-	-	1	0	1	?	0	0	2	2	1	1	1
Varasichthys_ariasi	0	0	2	1	1	0	0	2	0	?	?	?	1	1	1

Taxon / Character number	16	17	18	19	20	21	22	23	24	25	26	27	28
Amia_calva	2	1	0	0	0	0	0	0	0	1	0	1	0
Amia_pattersoni	?	?	0	0	0	?	0	0	0	1	0	1	0
Lepisosteus_osseus	?	2	0	0	0	2	0	1	0	1	0	1	1
Obaichthys_decoratus	?	1	0	0	?	2	0	1	0	0	0	1	1
Aspidorhynchus_acutirostris	?	1	0	0	?	?	1	0	0	1	0	1	0
Belonostomus_tenuirostris	?	?	0	0	0	?	1	?	?	1	0	1	0
Hypsocormus_macrodon	?	1	0	0	?	?	0	0	1	?	?	1	0
Mesturus_verrucosus	?	?	0	0	1	2	1	0	0	0	1	0	1
Pachycormus_macropterus	1	1	0	0	?	?	0	0	1	1	0	1	0
Vinctifer_comptoni	?	?	0	0	?	?	1	0	0	0	0	1	0
Apsopelix_anglicus	2	?	0	0	1	?	1	0	?	0	2	?	0
Allothrissops_mesogaster	?	0	1	0	1	?	1	?	0	1	0	0	0
Anaethalion_angustus	?	0	0	0	0	0	1	0	0	1	0	1	0
Anaethalion_knorri	?	?	0	0	0	?	1	0	0	?	0	1	0
Ascalabos_voithii	?	?	0	0	?	?	1	?	0	?	0	?	0
Bavarichthys_incognitus	?	?	0	0	?	?	?	?	0	0	?	?	0
Cavenderichthys_talbragarensis	?	?	0	0	?	?	1	?	0	?	?	?	0
Brycon_meeki	2	0	0	0	1	1	1	1	0	0	0	?	0
Chanos_chanos	2	0	0	0	1	1	1	1	0	0	0	?	0
Chongichthys_dentatus	2	?	?	?	?	?	?	0	0	0	0	?	0
Crossognathus_sabaudians	?	?	0	0	1	?	?	0	0	0	?	?	0
Denticeps_clupeoides	2	1	0	0	0	1	1	0	1	0	0	1	0
Diplomystus_dentatus	2	0	0	0	?	?	1	?	0	0	0	0	0
Domeykos_profetaensis	?	?	0	0	?	?	1	?	0	0	0	?	0
Dorsetichthys_bechei	0	0	0	0	0	0	1	0	0	1	0	0	0
Engraulis_ringens	2	0	0	0	0	1	1	0	0	0	0	1	0
Elops_saurus	2	0	0	0	0	0	1	0	0	1	0	1	0
Erichalcis_arcta	?	?	0	0	1	?	1	?	0	0	?	0	0
Esox_lucius	2	0	0	1	0	1	1	0	0	0	0	?	0
Eurycormus_speciosus	?	?	0	0	?	?	1	?	0	0	0	?	0
Gordichthys_conquensis	?	?	0	0	1	?	1	1	0	1	?	?	0

Taxon / Character number	16	17	18	19	20	21	22	23	24	25	26	27	28
**Goulmimichthys_arambourgi**	2	0	0	0	0	?	1	?	0	0	1	?	0
**Hiodon_alosoides**	1	0	0	0	0	2	1	0	1	2	0	1	0
**Heterotis_niloticus**	2	1	0	0	0	2	1	1	0	0	0	0	0
**Humbertia_operta**	2	0	0	0	0	?	1	0	1	0	0	0	0
**Leptolepis_coryphaenoides**	0&1	0	0	0	0	0	1	0	0	1	0	0	0
**Leptolepis_koonwarri**	?	?	0	0	?	?	1	?	0	?	?	?	0
**Leptolepides_haerteisi**	?	?	0	0	?	?	?	0	1	1	0	?	0
**Leptolepides_sprattiformis**	2	?	0	0	1	?	1	0	0	1	0	?	0
**Luisichthysvinalaensis**	2	?	0	0	?	?	1	?	0	?	0	?	0
**Luisiella_feruglioi**	?	?	0	0	?	?	1	?	0	?	?	?	0
**Lycoptera_middendorfi**	?	?	0	0	0	?	1	0	0	2	0	1	0
**Megalops_atlanticus**	2	0	0	0	0	0	1	0	0	1	0	1	0
**Notelops_brama**	2	0	0	0	0	?	?	0	0	0	1	?	0
**Oncorhynchus_mykiss**	2	0	0	0	0	1	1	0	1	0	0	?	0
**Opsariichthys_bidens**	2	0	0	0	1	1	1	1	1	0	0	1	0
**Orthogonikleithrus_hoelli**	?	?	0	0	1	?	?	0	1	0	0	1	0
**Pachythrissops_propterus**	?	?	0	0	0	?	?	?	0	1	0	?	0
**Pholidophorus_gervasutti**	?	?	?	?	?	?	?	?	?	?	?	?	0
**Pholidophorus_latiusculus**	?	?	?	?	?	?	?	?	?	?	?	?	0
**Protoclupea_chilensis**	?	?	?	?	?	?	?	?	0	0	0	?	0
**Rhacolepis_buccalis**	2	0	0	0	1	?	1	0	0	0	1	?	0
**Santanaclupea_silvasantosi**	?	0	0	0	?	?	1	?	0	0	0	?	0
**Siemensichthys_macrocephalus**	1	?	0	0	0	0	1	?	0	0	0	?	0
**Siemensichthys_siemensi**	?	?	0	0	0	?	?	?	?	?	?	?	0
**Tharrhias_araripis_**	?	?	0	0	?	?	1	1	0	?	?	0	0
**Tharsis_dubius**	2	0	0	0	0	0	1	0	0	0	0	0	0
**Thrissops_formosus**	2	?	1	0	?	?	?	0	0	?	0	0	0
**Thymallus_thymallus**	2	0	0	0	0	1	1	0	?	0	0	1	0
**Umbra_krameri**	2	0	0	1	0	1	1	?	0	0	0	1	0
**Varasichthys_ariasi**	2	?	0	0	?	?	?	0	0	0	2	?	0

Taxon / Character number	29	30	31	32	33	34	35	36	37	38	39	40
Amia_calva	0	0&1	0	3	2	-	0	0	0	2	0	0
Amia_pattersoni	0	0	0	3	2	-	0	0	0	?	0	0
Lepisosteus_osseus	0	0	1	0	0	0	0	0	0	2	?	0
Obaichthys_decoratus	0	0	1	0	0	0	0	?	?	?	?	0
Aspidorhynchus_acutirostris	?	0	0	1	1	0	0	0	0	0	1	0
Belonostomus_tenuirostris	?	0	0	1	1	0	0	?	-	-	0	0
Hypsocormus_macrodon	0	0	0	1	2	-	0	0	0&1	1	0	0
Mesturus_verrucosus	?	?	0	3	2	-	0	?	-	-	0	?
Pachycormus_macropterus	0	0	0	1	2	-	0	0	0	1	0	0
Vinctifer_comptoni	?	0	0	1	1	0	0	1	-	-	0	0
Apsopelix_anglicus	?	1	1	3	0&1	1	1	?	?	0	1	?
Allothrissops_mesogaster	?	0	1	3	1	0	1	?	1	0	1	1
Anaethalion_angustus	0	0	0	3	1	0	?	0	0	0	0	?
Anaethalion_knorri	0	0	0	3	1	0	0	0	0	0	0	?
Ascalabos_voithii	?	0	0	3	?	?	0	?	?	?	0	0
Bavarichthys_incognitus	0	1	?	3	1	1	?	1	-	-	1	?
Cavenderichthys_talbragarensis	0	0	0	3	1	0	0	0	0	0	0	1
Brycon_meeki	0	0	0	3	1	0	?	0	1	0	0	0
Chanos_chanos	0	0	0	3	1	0	0	0	1	0	1	0
Chongichthys_dentatus	?	1	?	?	?	?	?	?	?	?	1	0
Crossognathus_sabaudians	0	1	1	3	1	1	1	1	-	-	1	?
Denticeps_clupeoides	?	0	0	3	1	0	0	0	1	0	0	0
Diplomystus_dentatus	?	0	0	3	1	0	0	0	1	0	0	0
Domeykos_profetaensis	?	?	?	?	?	?	?	?	?	?	?	?
Dorsetichthys_bechei	0	0	0	2	0	0	0	0	0	0	0	0
Engraulis_ringens	0	0	0	3	2	-	0	0	1	0	0	0
Elops_saurus	?	0	1	3	1	0	1	0	0	0	1	0
Erichalcis_arcta	?	0	0	3	1	0	0	0	1	2	?	0
Esox_lucius	?	0	0	3	1	0	0	0	1	0	0	0
Eurycormus_speciosus	0	0	0	1&2	0	0	0	0	0	1	0	0
Gordichthys_conquensis	?	0	0	3	1	0	0	0	0	?	0	?

Taxon / Character number	29	30	31	32	33	34	35	36	37	38	39	40
Goulmimichthys_arambourgi	?	0	1	3	1	1	1	1	-	-	1	0
Hiodon_alosoides	1	1	0	3	2	-	0	1	-	-	0	0
Heterotis_niloticus	0	1	0	3	2	-	0	1	-	-	0	0
Humbertia_operta	?	0	0	3	1	0	0	0	1	2	0	0
Leptolepis_coryphaenoides	0	0	0	2	0	0	0	0	0	0	0	1
Leptolepis_koonwarri	0	0	0	3	1	0	0	0	0&1	?	0	1
Leptolepides_haerteisi	0	0	0	3	0	1	0	0	1	2	0	0
Leptolepides_sprattiformis	0	0	0	3	1	1	0	0	1	2	0	0
Luisichthysvinalaensis	?	0	?	?	?	?	?	0	0	?	0	?
Luisiella_feruglioi	0	0	0	3	1	0	0	0	0	0	0	1
Lycoptera_middendorfi	1	0	0	3	2	-	0	0	1	0	0	0
Megalops_atlanticus	?	0	1	3	1	0	0	0	0	0	1	0
Notelops_brama	0	1	1	3	1	1	1	1	-	-	1	0
Oncorhynchus_mykiss	0	0	0	3	1	0	0	0	1	0	0	0
Opsariichthys_bidens	0	0	0	3	1	0	0	0	1	0	0	0
Orthogonikleithrus_hoelli	0	0	0	3	1	?	0	?	?	?	0	0
Pachythrissops_propterus	0	0	?	3	1	0	0	?	?	?	0	?
Pholidophorus_gervasutti	0	0	0	1&2	0&1	?	0	0	0	?	?	0
Pholidophorus_latiusculus	0	0	?	2	0	0	0	?	?	?	0	?
Protoclupea_chilensis	0	0	?	3	?	0	?	0	0	0	0	0
Rhacolepis_buccalis	0	0	1	3	1	1	1	1	-	-	1	0
Santanaclupea_silvasantosi	?	?	?	3	?	?	?	?	?	?	?	0
Siemensichthys_macrocephalus	0	0&1	0	3	1	0	?	?	?	?	0	?
Siemensichthys_siemensi	0	?	?	3	?	?	?	0	0	?	0	?
Tharrhias_araripis_	0	0	0	3	1	0	1	0	1	0	0	0
Tharsis_dubius	?	?	0	3	1	0	0	0	0	0	?	1
Thrissops_formosus	?	0	1	3	1	0	1	0	1	0	0	?
Thymallus_thymallus	?	0	0	3	1	0	0	0	0	0	0	0
Umbra_krameri	?	0	?	3	1	0	0	0	1	-	0	0
Varasichthys_ariasi	?	0	?	2	1	0	?	0	0	0	0	0

Taxon / Character number	41	42	43	44	45	46	47	48	49	50	51	52
Amia_calva	0	0	0	3	0	0	1	1	0	0	0	0
Amia_pattersoni	0	?	0	3	0	0	1	?	0	0	0	0
Lepisosteus_osseus	4	0	0	0	1	1	0	1	0	0	0	1
Obaichthys_decoratus	4	0	0	0	?	1	0	1	0	?	0	1
Aspidorhynchus_acutirostris	0	0	0	0	1	1	1	1	0	0	0	1
Belonostomus_tenuirostris	0	0	0	0	1	1	?	1	0	0	?	1
Hypsocormus_macrodon	0	0	0	0	1	1	1	1	0	?	?	0
Mesturus_verrucosus	?	0	?	0	0	?	1	1	0	?	0	1
Pachycormus_macropterus	0	0	0	0	1	1	1	1	0	?	?	0
Vinctifer_comptoni	0	0	0	0	0	1	?	1	0	0	0	1
Apsopelix_anglicus	0	0	1	1	1	0	?	?	?	?	?	1
Allothrissops_mesogaster	0	0	1	0	1	0	0	0	1	?	1	1
Anaethalion_angustus	0	?	1	3	1	0	0	0	1	?	1	0
Anaethalion_knorri	0	?	1	0	1	0	0	0	?	?	1	?
Ascalabos_voithii	0	0	1	0	1	0	0	0	1	?	1	1
Bavarichthys_incognitus	0	0	?	?	1	0	?	?	?	?	?	1
Cavenderichthys_talbragarensis	0	0	1	0	1	0	0	0	1	?	1	0
Brycon_meeki	1	0	1	4	1	1	1	1	1	?	1	1
Chanos_chanos	1	0	1	0	1	0	?	?	1	2	1	1
Chongichthys_dentatus	0	1	1	0	1	?	0	?	?	?	?	1
Crossognathus_sabaudians	0	?	?	?	1	?	?	?	?	?	?	0&1
Denticeps_clupeoides	0	1	1	3	1	1	0	1	1	2	1	1
Diplomystus_dentatus	0	?	1	?	1	0	0	0	?	?	?	1
Domeykos_profetaensis	0	?	1	1	1	0	?	?	?	?	?	1
Dorsetichthys_bechei	0	?	1	0	1	1	?	1	?	?	-	0
Engraulis_ringens	3	1	1	0	1	0	1	1	1	2	1	1
Elops_saurus	0	1	1	2	1	0	0	1	1	1	1	0
Erichalcis_arcta	0	0	1	0	1	0	0	0	1	?	?	1
Esox_lucius	0	?	1	3	1	?	0	?	1	2	1	1
Eurycormus_speciosus	?	?	1	?	1	0	?	?	?	?	?	0
Gordichthys_conquensis	1	1	1	0	1	0	1	?	?	?	?	1

Taxon / Character number	41	42	43	44	45	46	47	48	49	50	51	52
Goulmimichthys_arambourgi	0	0	1	2	1	0	0	0	?	?	1	1
Hiodon_alosoides	0	1	1	3	1	0	0	1	1	1	1	1
Heterotis_niloticus	0	1	1	0	1	1	0	1	0	1	1	1
Humbertia_operta	0	0	1	0	1	0	0	0	1	?	1	1
Leptolepis_coryphaenoides	0	0	1	?	1	0	?	?	1	?	1	0
Leptolepis_koonwarri	0	0	1	0	1	0	0	0	0&1	?	?	0
Leptolepides_haerteisi	2	0	1	0	?	0	?	?	?	?	?	0
Leptolepides_sprattiformis	2	0	1	0	1	0	0	0	1	?	1	0
Luisichthysvinalaensis	0	?	1	?	1	?	?	?	?	?	?	0
Luisiella_feruglioi	0	0	1	0	1	0	0	0	1	1&2	1	0
Lycoptera_middendorfi	0	0	1	?	1	0	0	1	0&1	?	?	0
Megalops_atlanticus	0	0	1	1	1	0	?	1	1	1	1	0
Notelops_brama	0	0	1	2	1	1	0	0	1	?	1	0
Oncorhynchus_mykiss	0	1	1	0	1	?	0	?	1	2	1	1
Opsariichthys_bidens	1	1	1	4	1	?	?	?	1	2	1	1
Orthogonikleithrus_hoelli	2	0	1	0	1	0	0	0	1	?	1	1
Pachythrissops_propterus	0	0	1	0	1	?	?	0	1	?	?	0
Pholidophorus_gervasutti	?	0	0	0	0&1	?	?	1	0	?	?	0
Pholidophorus_latiusculus	0	?	?	?	?	?	?	?	?	?	?	0
Protoclupea_chilensis	0	0	1	0	1	0	?	?	?	?	?	1
Rhacolepis_buccalis	0	0	1	1	1	0	?	0	1	?	1	1
Santanaclupea_silvasantosi	3	1	1	1	1	?	?	0	1	2	1	1
Siemensichthys_macrocephalus	0	?	1	?	1	?	?	1	1	?	?	0
Siemensichthys_siemensi	0	?	1	0	1	?	?	?	?	?	?	0
Tharrhias_araripis_	1	1	1	3	1	0	?	?	1	?	1	?
Tharsis_dubius	0	0	1	0	1	0	0	0	1	?	1	0
Thrissops_formosus	0	0	1	0	1	0	0	0	1	?	1	1
Thymallus_thymallus	0	1	1	0	1	?	0	?	1	2	1	1
Umbra_krameri	0	0	1	0	1	0	1	1	1	2	1	1
Varasichthys_ariasi	0	?	1	?	1	0	?	?	1	?	?	0

Taxon / Character number	53	54	55	56	57	58	59	60	61	62	63	64
Amia_calva	0	0	1	0	1	0	0	0	0	1	3	1
Amia_pattersoni	0	0	1	0	1	0	0	0	0	1	3	1
Lepisosteus_osseus	0	0	0	1	1	2	0	0	-	-	-	1
Obaichthys_decoratus	0	0	0	0	1	2	?	0	-	-	-	?
Aspidorhynchus_acutirostris	0	0	2	0	1	2	?	0	2	1	1	1
Belonostomus_tenuirostris	0	0	2	1	1	2	?	0	0	1	1	1
Hypsocormus_macrodon	0	0	0	0	?	3	-	0	0	1	0&1	1
Mesturus_verrucosus	0	0	2	1	0	0	?	1	2	0	0&1	2
Pachycormus_macropterus	0	0	0	0	?	3	-	0	0	1	0&1	1
Vinctifer_comptoni	0	0	2	1	1	2	?	0	2	1	1	2
Apsopelix_anglicus	0	1	0	0	0	0	?	0	0	0	1	0
Allothrissops_mesogaster	0	1	0	0	0	0	1	0	1	0	1	0
Anaethalion_angustus	0	0	0	0	0	0	0	0	1	0	1	0
Anaethalion_knorri	0	0	0	0	0	0	?	0	1	0	1	0
Ascalabos_voithii	0	0	0	0	0	0	0&1	0	1	0	1	0
Bavarichthys_incognitus	0	0	2	0	0	0	0	0	2	0	1	0
Cavenderichthys_talbragarensis	0	0	0	0	0	0	?	0	1	0	1	0
Brycon_meeki	0	0	0	0	?	0	0&1	1	0	0	1	2
Chanos_chanos	1	0	0	0	1	0	0	1	1	2	1	2
Chongichthys_dentatus	0	0	?	0	?	?	?	0	0	0	1	?
Crossognathus_sabaudians	0	1	0	0	0	0	0	0	0	0	1	?
Denticeps_clupeoides	0	0	0	0	0	0	?	0	?	1	2	2
Diplomystus_dentatus	0	0	0	0	0	0	0&1	0	1	0	1	0
Domeykos_profetaensis	0	1	2	0	0	?	?	0	1	0	1	0
Dorsetichthys_bechei	0	0	2	0	0	0	0	0	1	0	1	0
Engraulis_ringens	0	0	1	0	?	?	?	0	0	0	1	0
Elops_saurus	0	0	0	0	1	0	1	0	0	0	1	0
Erichalcis_arcta	0	0	0	0	0	0	0	0	1	0	1	0
Esox_lucius	0	0	1	0	0	0	0	1	0	1	1	1
Eurycormus_speciosus	?	0	2	0	1	0	?	0	1	0	1	0
Gordichthys_conquensis	1	1	0	0	0	0	1	1	1	1	1	2

Taxon / Character number	53	54	55	56	57	58	59	60	61	62	63	64
Goulmimichthys_arambourgi	0	1	?	0	0	0	?	0	0	0	1	1
Hiodon_alosoides	0	0	0	0	1	0	0	0	0	1	1&2	2
Heterotis_niloticus	0	0	0	0	1	1	0	0	0	1	1	2
Humbertia_operta	0	0	0	0	0	0	1	0	1	0	1&2	0
Leptolepis_coryphaenoides	0&1	0	0	0	0	0	0	0	1	0	1	0
Leptolepis_koonwarri	0	0	0	0	0	0	?	1	1	0	1	0
Leptolepides_haerteisi	0	0	0	0	0	0	?	0	1	0	1	0
Leptolepides_sprattiformis	0	0	0	0	0	0	0	0	1	0	1	0
Luisichthysvinalaensis	0	1	2	0	0	0	0	0	1	0	1	0
Luisiella_feruglioi	0	0	0	0	0	0	?	0	1	0	1	0
Lycoptera_middendorfi	0	0	2	0	1	0	0	0	0	0	1	1
Megalops_atlanticus	0	0	0	0	0	0	?	0	1	0	1	0
Notelops_brama	0	0	2	0	0	0	?	0	0	0	2	1
Oncorhynchus_mykiss	1	0	0	0	1	0	0	0	2	0	1	1
Opsariichthys_bidens	0	0	0	0	0	0	1	1	1	0	0	2
Orthogonikleithrus_hoelli	0	0	0	0	0	0	?	0	1	0	1	0
Pachythrissops_propterus	?	0	?	0	?	0	?	0	1	?	1	0
Pholidophorus_gervasutti	?	0	2	0	0	?	0	0	1	0	1&3	0
Pholidophorus_latiusculus	?	0	2	0	0	?	?	?	1	0	?	0
Protoclupea_chilensis	0	1	2	0	0	?	?	0	1	0	?	?
Rhacolepis_buccalis	0	1	2	0	0	0	0	0	0	0	2	1
Santanaclupea_silvasantosi	0	?	?	0	1	0	?	0	?	?	1	?
Siemensichthys_macrocephalus	?	0	2	0	0	?	1	0	1	0	2	1
Siemensichthys_siemensi	?	0	2	0	?	0	1	0	1	0	1	1
Tharrhias_araripis_	1	0	0	0	1	0	1	1	?	2	1	2
Tharsis_dubius	0	0	0	0	0	0	?	0	1	0	1	0
Thrissops_formosus	0	0	0	0	0	0	0	0	0&1	0	1	0
Thymallus_thymallus	1	0	0	0	1	0	0	0	1	0	1	?
Umbra_krameri	0	0	0	0	0	0	0	1	0	0	1	2
Varasichthys_ariasi	0	1	2	0	0	0	0	0	1	0	1	1

Taxon / Character number	65	66	67	68	69	70	71	72	73	74	75	76
Amia_calva	0	0	0	0	1	0	0	0	0	0	0	1
Amia_pattersoni	0	0	0	0	1	0	0	0	0	0	0	1
Lepisosteus_osseus	0	3	0	0	1	0	0	0	0	0	0	0
Obaichthys_decoratus	?	3	0	0	1	0	0	0	0	0	0	0
Aspidorhynchus_acutirostris	1	0	1	1	-	?	0	?	?	0	0	1
Belonostomus_tenuirostris	1	0	1	1	-	?	0	?	?	0	0	1
Hypsocormus_macrodon	?	0	0	1	-	?	?	?	0	0	0	0
Mesturus_verrucosus	-	1&2	0	1	-	1	?	?	0	0	0	?
Pachycormus_macropterus	1	0	0	1	-	0	0	0	0	0	0	0
Vinctifer_comptoni	-	0	1	0	0	?	0	0	?	0	0	1
Apsopelix_anglicus	0	2	0	0	?	1	1	1	1	1	0	0
Allothrissops_mesogaster	0	1	0	0	0	1	1	0	1	1	0	0
Anaethalion_angustus	0	1	0	1	-	1	1	1	1	1	0	0
Anaethalion_knorri	0	1	0	1	-	1	1	1	1	1	0	0
Ascalabos_voithii	0	1	0	1	-	1	1	1	1	1	1	0
Bavarichthys_incognitus	0	0	0	0	1	1	1	1	1	1	0	0
Cavenderichthys_talbragarensis	0	1&2	0	0	0	1	1	1	1	1	0	0
Brycon_meeki	-	2	0	0	1	1	1	1	1	0	0	1
Chanos_chanos	-	3	0	0	0&1	1	1	1	1	0	1	-
Chongichthys_dentatus	0	0	?	1	-	1	1	0	1	1	0	?
Crossognathus_sabaudians	0	1	0	?	?	1	1	1	1	0	0	0
Denticeps_clupeoides	-	2	0	0	1	1	1	1	1	1	0	0
Diplomystus_dentatus	0	1	0	0	1	1	1	1	1	1	?	0
Domeykos_profetaensis	0	1	0	1	-	1	1	0	1	1	?	?
Dorsetichthys_bechei	0	0	0	1	-	1	0	2	?	0	0	0
Engraulis_ringens	0	0	0	0	1	1	1	1	1	1	0	0
Elops_saurus	0	0	0	1	-	1	1	2	1	1	0	1
Erichalcis_arcta	0	1	0	0	1	1	1	1	1	1	0	0
Esox_lucius	0	0	0	0	1	1	1	1	1	1	0	1
Eurycormus_speciosus	0	1	0	?	?	?	?	?	?	0	0	1
Gordichthys_conquensis	-	3	0	0	1	1	1	1	1	0	1	?

Taxon / Character number	65	66	67	68	69	70	71	72	73	74	75	76
Goulmimichthys_arambourgi	0	0	0	?	?	1	1	1	1	1	0	0
Hiodon_alosoides	-	1	0	1	-	1	1	0	1	1	0	1
Heterotis_niloticus	-	2	0	0	0&1	1	1	0	1	1	0	0
Humbertia_operta	0	1	0	0	1	1	1	1	1	1	0	0
Leptolepis_coryphaenoides	0	1	0	0&1	-	1	1	1	1	0	1	0
Leptolepis_koonwarri	0	1	0	1	-	1	1	1	1	1	1	0
Leptolepides_haerteisi	0	2	0	0	?	?	1	1	1	1	?	0
Leptolepides_sprattiformis	0	2	0	0	0	1	1	1	1	1	0	?
Luisichthysvinalaensis	0	1	0	?	?	1	1	?	?	1	?	0
Luisiella_feruglioi	0	1	0	1	-	1	1	1	1	1	0	0
Lycoptera_middendorfi	0	1	0	?	?	1	1	?	?	1	0	0
Megalops_atlanticus	0	0	0	1	-	1	1	2	1	1	0	0
Notelops_brama	0	0	0	0	1	1	1	0	1	1	0	0
Oncorhynchus_mykiss	0	0	0	0	1	1	1	1	1	1	0	?
Opsariichthys_bidens	-	2	0	0	1	1	1	1	1	1	0	-
Orthogonikleithrus_hoelli	0	1	0	0	1	1	1	1	1	0	0	?
Pachythrissops_propterus	0	1	0	1	-	?	1	1	1	1	0	1
Pholidophorus_gervasutti	0	0&1	0	1	-	?	0	?	?	0	1	0
Pholidophorus_latiusculus	0	0	0	?	?	?	?	?	?	0	?	?
Protoclupea_chilensis	0	1	0	1	-	1	1	0	1	1	1	?
Rhacolepis_buccalis	0	0	0	0	1	1	1	1	1	1	0	0
Santanaclupea_silvasantosi	0	0	0	?	?	1	1	?	1	1	0	?
Siemensichthys_macrocephalus	0	0	0	1	-	?	0	2	?	0	0	0
Siemensichthys_siemensi	0	0	0	?	?	?	0	2	0	0	0	?
Tharrhias_araripis_	-	3	0	0	1	1	1	0	1	0	0	-
Tharsis_dubius	0	1	0	0	0	1	1	1	1	1	1	0
Thrissops_formosus	0	1	0	0	1	1	1	0	1	1	0	0
Thymallus_thymallus	0	1	0	0	1	1	1	1	1	1	0	?
Umbra_krameri	-	2	0	0	1	1	1	1	1	0	0	0
Varasichthys_ariasi	0	1	0	0	1	1	1	1	1	0	1	0

Taxon / Character number	77	78	79	80	81	82	83	84	85	86	87	88	89
Amia_calva	0	0	0	1	0	0	0	0	0	0	0	0	0
Amia_pattersoni	?	0	?	?	?	0	0	0	0	?	?	?	?
Lepisosteus_osseus	0	0	0	1	0	?	0	0	0	1	0	0	0
Obaichthys_decoratus	?	0	?	?	?	?	?	0	?	?	?	?	?
Aspidorhynchus_acutirostris	0	0	0	?	0	?	?	0	?	?	?	?	?
Belonostomus_tenuirostris	0	0	0	?	0	?	?	0	?	?	?	?	?
Hypsocormus_macrodon	0	0	?	1	-	0	?	0	0	2	?	?	?
Mesturus_verrucosus	1	0	?	0	1	?	0	?	0	?	?	?	?
Pachycormus_macropterus	0	0	0	1	-	0	-	0	0	2	?	?	?
Vinctifer_comptoni	0	0	0	1	0	1	1	0	?	?	?	?	?
Apsopelix_anglicus	1	0	1	0	0	?	1	0	1	?	?	?	?
Allothrissops_mesogaster	1	0	1	0	0	0	1	0	?	?	?	?	?
Anaethalion_angustus	1	0	?	1	?	0	1	0	?	?	?	?	?
Anaethalion_knorri	1	0	?	1	0	0	1	0	?	?	?	?	?
Ascalabos_voithii	1	0	?	0	?	0	1	0	?	?	?	?	?
Bavarichthys_incognitus	?	?	?	?	?	?	0	0	?	?	?	?	?
Cavenderichthys_talbragarensis	1	0	?	0	0	0	0	0	?	?	?	?	?
Brycon_meeki	1	0	1	0	1	0&1	0	0	1	0	1	1	1
Chanos_chanos	1	0	1	0	1	1	1	0	1	2	1	1	1
Chongichthys_dentatus	1	0	1	?	?	0	0	0	?	?	?	?	?
Crossognathus_sabaudians	1	0	1	0	0	0	1	0	?	?	?	?	?
Denticeps_clupeoides	1	0	1	?	1	?	?	0	1	2	1	1	1
Diplomystus_dentatus	1	0	1	0	1	0&1	0	0	?	2	?	?	?
Domeykos_profetaensis	1	0	1	?	0	?	2	0	?	?	?	?	?
Dorsetichthys_bechei	0	0	0	1	0	0	2	0	?	?	?	?	?
Engraulis_ringens	0	0	1	0	1	?	?	0	1	2	1	1	1
Elops_saurus	0	0	2	1	0	1	1	0	1	2	0	?	0
Erichalcis_arcta	1	0	1	0	1	0	1	0	?	?	?	?	?
Esox_lucius	1	1	2	0	?	?	0	1	1	2	1	0	0
Eurycormus_speciosus	0	0	0	1	0	?	2	0	?	?	?	?	?
Gordichthys_conquensis	1	0	1	?	?	1	?	0	?	?	?	?	?

Taxon / Character number	77	78	79	80	81	82	83	84	85	86	87	88	89
Goulmimichthys_arambourgi	?	?	1	?	0	?	?	0	?	?	?	?	?
Hiodon_alosoides	1	0	2	0	0	0&1	1	0	1	2	1	1	0
Heterotis_niloticus	1	0	1	0	1	1	0	0	1	2	1	?	1
Humbertia_operta	1	0	1	0	1	1	1	0	?	?	?	?	?
Leptolepis_coryphaenoides	0&1	0	0	1	0	0	2	0	?	?	?	?	?
Leptolepis_koonwarri	1	0	?	?	0	0	0	0	?	?	?	?	?
Leptolepides_haerteisi	1	0	0	0	?	1	0	0	?	?	?	?	?
Leptolepides_sprattiformis	1	0	1	0	0	0	0	0	1	?	?	?	?
Luisichthysvinalaensis	1	?	1	0	?	?	2	0	?	?	?	?	?
Luisiella_feruglioi	1	0	0	0	0	0	1	0	?	?	?	?	?
Lycoptera_middendorfi	1	0	0	0	0	0	0	0	?	?	?	?	?
Megalops_atlanticus	0	0	1	1	-	?	1	0	1	2	0	0	0
Notelops_brama	?	0	1	1	0	1	0	0	?	?	?	?	?
Oncorhynchus_mykiss	1	0	1	0	0	1	0	0	1	2	1	1	1
Opsariichthys_bidens	1	0	1	0	?	1	0	0	1	0	1	1	1
Orthogonikleithrus_hoelli	1	0	1	0	?	1	0	0	?	?	?	?	?
Pachythrissops_propterus	1	0	?	0	?	?	?	0	?	?	?	?	?
Pholidophorus_gervasutti	0	?	0	1	0	?	?	0	?	?	?	?	?
Pholidophorus_latiusculus	0	0	1	?	?	0	2	0	?	?	?	?	?
Protoclupea_chilensis	1	0	0	?	0	0	2	0	?	?	?	?	?
Rhacolepis_buccalis	?	0	1	0	0	1	1	0	?	2	?	?	?
Santanaclupea_silvasantosi	?	0	1	?	1	0	0&1	0	?	?	?	?	?
Siemensichthys_macrocephalus	0	0	?	?	?	0	2	0	?	?	?	?	?
Siemensichthys_siemensi	0	0	?	?	?	?	2	0	?	?	?	?	?
Tharrhias_araripis_	1	0	?	0	1	1	?	0	?	?	?	?	?
Tharsis_dubius	0	0	1	1	0	0	2	0	?	?	?	?	?
Thrissops_formosus	1	0	1	0	0	0	1	0	?	?	?	?	?
Thymallus_thymallus	1	0	1	0	0	1	0	0	1	2	1	1	1
Umbra_krameri	1	1	1	0	?	1	0	1	1	2	1	1	1
Varasichthys_ariasi	1	0	0	0	0	0	2	0	?	?	?	?	?

Taxon / Character number	90	91	92	93	94	95	96	97	98	99	100	101	102
Amia_calva	0	?	?	?	0	0	0	0	0	0	0	1	0
Amia_pattersoni	0	?	?	?	0	0	0	0	0	0	0	?	0
Lepisosteus_osseus	1	0	-	-	2	0	0	0	0	?	0	1	0
Obaichthys_decoratus	1	0	-	-	2	0	?	0	0	0	?	1	0
Aspidorhynchus_acutirostris	0	0	-	-	0	0	?	0	0	?	?	?	0
Belonostomus_tenuirostris	0	0	-	-	0	0	?	0	0	?	?	?	0
Hypsocormus_macrodon	0	0	-	-	0	0	?	0	0	?	1	1	0
Mesturus_verrucosus	0	0	-	-	0	?	0	0	0	?	?	0	0
Pachycormus_macropterus	0	0	-	-	0	0	?	0	0	0	1	1	0
Vinctifer_comptoni	0	0	-	-	0	0	?	0	0	?	?	?	0
Apsopelix_anglicus	0	1	1	1	0	?	0	0	0	0	?	0	1
Allothrissops_mesogaster	0	1	1	1	0	0	0	0	0	?	1	1	1
Anaethalion_angustus	0	1	1	1	0	0	0	0	0	0	?	1	1
Anaethalion_knorri	0	1	1	1	0	0	0	0	0	0	1	1	1
Ascalabos_voithii	0	1	1	1	0	0	0	0	0	1	1	1	1
Bavarichthys_incognitus	0	1	1	1	0	0	0	0	0	1	1	1	1
Cavenderichthys_talbragarensis	0	1	0	0	?	0	0	0	0	0	1	1	1
Brycon_meeki	0	?	1	1	1	0	1	1	2	0	1	1	1
Chanos_chanos	0	1	1	1	0	1	1	0	1	1	?	1	1
Chongichthys_dentatus	0	1	1	1	0	0	0	0	0	0	0&1	1	1
Crossognathus_sabaudians	0	1	1	1	0	?	0	0	0	?	1	1	1
Denticeps_clupeoides	0	2	1	1	1	0	1	0	0	0	?	0	?
Diplomystus_dentatus	0	1	1	1	1	0	0	0	0	0	?	1	1
Domeykos_profetaensis	0	1	1	1	0	0	0	0	0	1	?	0	1
Dorsetichthys_bechei	0	0	-	-	0	0	0	0	0	?	?	?	1
Engraulis_ringens	0	2	1	1	1	0	1	0	0	?	?	1	1
Elops_saurus	0	1	1	1	0	0	0	0	0	0	?	1	1
Erichalcis_arcta	0	1	1	1	0	0	1	0	0	?	?	?	?
Esox_lucius	0	1	1	1	0	0	0	0	0	0	1	1	1
Eurycormus_speciosus	0	0	-	-	?	0	0	0	0	1	?	1	1
Gordichthys_conquensis	0	1	1	1	0	1	0	0	1	?	1	1	1

Taxon / Character number	90	91	92	93	94	95	96	97	98	99	100	101	102
Goulmimichthys_arambourgi	0	1	1	1	0	0	0	0	0	?	?	?	1
Hiodon_alosoides	0	1	1	1	0	0	1	0	0	1	0	1	1
Heterotis_niloticus	0	1	0	1	1	0	1	0	0	0	?	1	1
Humbertia_operta	0	1	1	1	0	0	0	0	0	0	?	1	1
Leptolepis_coryphaenoides	0	1	0	0	0	0	0	0	0	?	?	1	1
Leptolepis_koonwarri	0	1	?	0	0	0	0	0	0	?	1	1	1
Leptolepides_haerteisi	0	1	2	1	0	0	0	0	0	0	1	1	1
Leptolepides_sprattiformis	0	1	2	1	0	0	0	0	0	0	1	1	1
Luisichthysvinalaensis	0	1	1	1	0	0	0	0	0	?	?	1	1
Luisiella_feruglioi	0	1	0&1	1	0	0	0	0	0	1	1	1	1
Lycoptera_middendorfi	0	1	1	1	0	0	0	0	0	?	?	1	1
Megalops_atlanticus	0	1	1	1	0	0	0	0	0	?	?	?	1
Notelops_brama	0	1	1	1	0	0	0	0	0	1	0	1	1
Oncorhynchus_mykiss	0	1	1	1	0	0	0	0	0	1	?	1	1
Opsariichthys_bidens	0	1	2	1	1	0	1	1	2	0	?	1	1
Orthogonikleithrus_hoelli	0	1	1	1	0	0	0	0	0	1	1	1	1
Pachythrissops_propterus	0	1	1	1	0	0	0	0	0	0	?	1	1
Pholidophorus_gervasutti	0	0	-	-	?	?	?	?	?	?	?	?	?
Pholidophorus_latiusculus	0	0	-	-	?	?	?	?	?	?	?	?	?
Protoclupea_chilensis	0	1	1	1	0	0	0	0	0	?	1	?	1
Rhacolepis_buccalis	0	1	1	1	0	0	0	0	0	1	0	0	1
Santanaclupea_silvasantosi	0	?	1	1	0	?	0	0	0	0	?	1	1
Siemensichthys_macrocephalus	0	0	-	-	?	?	?	?	?	?	?	?	1
Siemensichthys_siemensi	0	?	?	?	?	?	?	?	?	?	?	?	?
Tharrhias_araripis_	0	1	1	1	0	1	1	0	1	1	0	1	?
Tharsis_dubius	0	1	1	1	0	0	0	0	0	0	1	?	1
Thrissops_formosus	0	1	1	1	0	0	0	0	0	0	1	1	1
Thymallus_thymallus	0	1	1	1	0	0	0	0	0	1	?	1	1
Umbra_krameri	0	1	1	1	1	0	0	0	0	0	1	1	?
Varasichthys_ariasi	0	1	1	1	0	0	0	0	0	?	1	0	1

Taxon / Character number	103	104	105	106	107	108	109	110	111	112	113
Amia_calva	0	-	0	0	0	0	0	0	0	0	0
Amia_pattersoni	0	-	0	0	0	0	0	0	?	0	0
Lepisosteus_osseus	0	-	0	0	0	0	0	0	0	0	0
Obaichthys_decoratus	0	-	0	0	0	0	0	0	0	0	0
Aspidorhynchus_acutirostris	?	?	0	0	0	?	0	0	?	1	0
Belonostomus_tenuirostris	?	?	0	?	0	2	0	?	?	0	0
Hypsocormus_macrodon	?	?	0	0	0	?	0	?	1	1	0
Mesturus_verrucosus	0	-	0	?	0	?	0	?	0	0	0
Pachycormus_macropterus	?	?	0	0	0	0	0	1	1	1	0
Vinctifer_comptoni	?	?	0	0	0	2	0	0	1	0	0
Apsopelix_anglicus	1	1	1	?	0	?	1	?	1	0	?
Allothrissops_mesogaster	0	-	0	0	0	0	1	1	1	0	0
Anaethalion_angustus	1	1	0	?	0	?	0	1	1	0	0
Anaethalion_knorri	?	?	0	1	0	?	0	?	?	0	0
Ascalabos_voithii	1	0	0	0	0	0	0	1	1	0	0
Bavarichthys_incognitus	1	1	1	?	0	0	0	?	?	0	?
Cavenderichthys_talbragarensis	1	0	0	?	0	0	0	1	1	0	?
Brycon_meeki	1	1	1	0	0	0	0	1	1	0	0
Chanos_chanos	1	1	1	0	0	2	0	1	1	0	1
Chongichthys_dentatus	1	0	0	?	0	?	0	?	?	0	?
Crossognathus_sabaudians	1	1	1	0	0	?	0	?	?	0	?
Denticeps_clupeoides	1	1	1	0	1	0	0	1	1	0	0
Diplomystus_dentatus	1	1	1	0	?	0	0	?	1	0	0
Domeykos_profetaensis	1	0	0	1	0	1	0	1	?	0	1
Dorsetichthys_bechei	0	-	0	?	0	0	0	?	?	0	0
Engraulis_ringens	1	1	1	0	1	0	0	1	1	0	1
Elops_saurus	1	1	1	0	0	1	0	1	1	0	1
Erichalcis_arcta	?	?	?	0	0	0	0	?	?	0	0
Esox_lucius	1	1	1	0	0	0	0	1	1	0	0
Eurycormus_speciosus	0	-	0	0	0	0	?	1	1	0	0
Gordichthys_conquensis	1	0	0	0	0	0	0	?	1	0	?

Taxon / Character number	103	104	105	106	107	108	109	110	111	112	113
Goulmimichthys_arambourgi	?	?	0	?	0	?	0	?	?	0	?
Hiodon_alosoides	-	?	0	0	0	0	1	1	1	0	1
Heterotis_niloticus	1	1	0	2	0	0	0	1	1	0	?
Humbertia_operta	1	1	1	?	0	0	0	1	1	0	0
Leptolepis_coryphaenoides	0	-	0	1	0	0	0	1	1	0	0
Leptolepis_koonwarri	0	-	?	0	0	?	0	1	1	0	?
Leptolepides_haerteisi	1	1	0	?	0	0	0	?	?	0	0
Leptolepides_sprattiformis	1	0	0	0	0	0	0	1	1	0	0
Luisichthysvinalaensis	1	0	0	?	0	1	0	?	?	0	?
Luisiella_feruglioi	0	-	0	0	0	0	0	1	1	0	0
Lycoptera_middendorfi	0	-	0	0	0	0	0	?	?	0	?
Megalops_atlanticus	?	?	1	0	0	1	0	1	1	0	1
Notelops_brama	0	-	0	0	0	0	0	?	?	0	?
Oncorhynchus_mykiss	1	0	0	0	0	1	0	1	1	0	0
Opsariichthys_bidens	?	?	1	0	0	0	0	1	1	0	0
Orthogonikleithrus_hoelli	?	?	0	?	0	0	0	?	1	0	?
Pachythrissops_propterus	1	1	0	?	0	?	1	1	?	0	0
Pholidophorus_gervasutti	?	?	?	0	?	0	?	0	?	?	1
Pholidophorus_latiusculus	?	?	?	?	?	0	?	?	?	0	?
Protoclupea_chilensis	?	?	0	1	0	1	0	?	?	0	1
Rhacolepis_buccalis	0	-	0	1	0	0	0	?	?	0	?
Santanaclupea_silvasantosi	?	?	0	?	0	0	?	?	?	0	?
Siemensichthys_macrocephalus	?	?	?	0	?	0	?	1	?	0	0
Siemensichthys_siemensi	?	?	?	?	?	0	?	?	?	0	?
Tharrhias_araripis_	0	-	0	?	0	0	0	1	1	0	?
Tharsis_dubius	1	0	0	0	0	0	0	1	1	0	0
Thrissops_formosus	0	-	0	?	0	0	1	1	?	0	0
Thymallus_thymallus	1	0	0	0	0	0	0	1	1	0	0
Umbra_krameri	1	1	?	1	0	0	0	1	1	0	0
Varasichthys_ariasi	1	0	0	1	1	1	0	0	?	0	1

Taxon / Character number	114	115	116	117	118	119	120	121	122	123	124
Amia_calva	0	1	0	0	0	0	1	1	0	0	1
Amia_pattersoni	0	1	0	0	0	0	1	1	0	0	1
Lepisosteus_osseus	0	0	0	1	0	0	1	0	0	0	0
Obaichthys_decoratus	0	0	0	1	0	?	?	0	0	0	0
Aspidorhynchus_acutirostris	0	1	0	1	0	?	?	0	0	0	0
Belonostomus_tenuirostris	0	1	0	1	0	?	?	0	0	0	0
Hypsocormus_macrodon	0	0	0	0	1	?	?	?	0	?	?
Mesturus_verrucosus	0	?	1	1	?	0	1	?	0	0	?
Pachycormus_macropterus	0	0	0	0	0	0	1	?	0	0	?
Vinctifer_comptoni	0	?	0	1	0	?	?	0	0	0	0
Apsopelix_anglicus	?	1	0	2	1	0	0	1	0	0	1
Allothrissops_mesogaster	0	1	0	1	1	1	0	1	1	0	1
Anaethalion_angustus	?	1	0	0	0	1	0	1	0	0	1
Anaethalion_knorri	0	1	0	0	0	1	0	1	0	0	1
Ascalabos_voithii	0	1	0	0	1	1	0	1	0	0	1
Bavarichthys_incognitus	?	1	0	0	1	1	1	1	0	0	1
Cavenderichthys_talbragarensis	?	1	0	0	0	1	1	1	0	0	1
Brycon_meeki	?	1	0	?	?	0	1	1	0	0	1
Chanos_chanos	1	1	0	0	0	0	?	1	0	0	1
Chongichthys_dentatus	0	1	0	0	1	0	1	1	0	0	1
Crossognathus_sabaudians	0	1	0	2	?	?	1	1	0	0	1
Denticeps_clupeoides	0	1	1	1	0	1	0	1	0	0	1
Diplomystus_dentatus	0	1	1	0	0	0	0	1	0	0	1
Domeykos_profetaensis	1	1	0	0	?	?	?	1	0	0	1
Dorsetichthys_bechei	0	0	0	0	0	?	?	0	0	0	0
Engraulis_ringens	?	1	1	0	0	1	0	1	0	0	1
Elops_saurus	1	1	0	0	1	0	1	1	0	0	1
Erichalcis_arcta	0	1	0	?	?	?	?	1	0	1	1
Esox_lucius	0	1	0	1	0	0&1	0	1	0	0	1
Eurycormus_speciosus	0	0	0	0	0	?	?	0	1	0	0
Gordichthys_conquensis	?	1	0	0	0	0	1	1	0	0	1

Taxon / Character number	114	115	116	117	118	119	120	121	122	123	124
Goulmimichthys_arambourgi	0	1	0	2	?	0	0	1	0	0	1
Hiodon_alosoides	1	1	0	1	1	0	1	1	0	0	1
Heterotis_niloticus	?	1	0	1	0	1	0	1	0	0	1
Humbertia_operta	?	1	0	0	0	1	1	1	0	1	1
Leptolepis_coryphaenoides	1	1	0	0	0	?	0	1	0	0	1
Leptolepis_koonwarri	?	1	0	0	0	?	?	1	0	0	1
Leptolepides_haerteisi	0	1	0	2	0	1	1	1	0	1	1
Leptolepides_sprattiformis	0	1	0	2	0	1	1	1	0	1	1
Luisichthysvinalaensis	?	1	0	?	?	?	?	1	0	0	1
Luisiella_feruglioi	?	1	0	0	0	1	0&1	1	0	0	1
Lycoptera_middendorfi	?	1	0	1	0	0	?	1	0	0	1
Megalops_atlanticus	1	1	0	0	1	?	?	1	0	0	1
Notelops_brama	0	1	0	2	1	1	0	1	0	0	1
Oncorhynchus_mykiss	1	1	0	0	0	0	?	1	0	0	1
Opsariichthys_bidens	1	1	0	0	0	?	?	1	0	0	1
Orthogonikleithrus_hoelli	?	1	0	2	0	1	1	1	0	1	1
Pachythrissops_propterus	0	1	0	1	1	1	1	1	1	0	1
Pholidophorus_gervasutti	1	0	?	0	0	?	?	0	?	?	?
Pholidophorus_latiusculus	?	0	?	0	?	?	?	0	?	?	0
Protoclupea_chilensis	1	1	0	0	0	?	0	1	0	0	1
Rhacolepis_buccalis	0	1	0	2	?	1	?	1	0	0	1
Santanaclupea_silvasantosi	?	1	1	0	0	1	0	1	0	0	1
Siemensichthys_macrocephalus	?	0	0	0	?	?	?	0	?	?	0
Siemensichthys_siemensi	?	0	0	0	?	?	?	0	?	?	?
Tharrhias_araripis_	?	1	0	2	0	?	?	1	0	0	1
Tharsis_dubius	0	1	0	0	0	1	?	1	0	0	1
Thrissops_formosus	0	1	0	1	1	1	1	1	1	0	1
Thymallus_thymallus	1	1	0	0	0	0	?	1	0	0	1
Umbra_krameri	0	1	0	1	0	0	1	1	0	0	1
Varasichthys_ariasi	1	1	0	0	1	1	1	1	0	0	1

Taxon / Character number	125	126	127	128	129	130	131	132	133	134
Amia_calva	2	1	0	0	1	0	0	2	0	0
Amia_pattersoni	2	1	0	0	1	0	?	?	0	0
Lepisosteus_osseus	1	0	0	0	0&1	0	0	2	0	0
Obaichthys_decoratus	?	?	?	?	?	?	0	?	0	0
Aspidorhynchus_acutirostris	?	1	0	0	1	0	0	?	0	0
Belonostomus_tenuirostris	2	1	0	0	?	0	0	0	0	0
Hypsocormus_macrodon	?	0	0	?	1	0	0	1	0	0
Mesturus_verrucosus	0	1	?	?	?	0	?	?	0	0
Pachycormus_macropterus	?	0	0	1	1	0	0	1	0	0
Vinctifer_comptoni	2	1	?	0	1	0	0	2	0	0
Apsopelix_anglicus	0	1	0	0	0	0	0	1	1	1
Allothrissops_mesogaster	1	1	0	0	1	1	0	1	2	2
Anaethalion_angustus	1	1	0	0	0&1	0	?	1	1	?
Anaethalion_knorri	1	1	0	0	0	0	0	1	1	1
Ascalabos_voithii	0	1	0	0	0	1	0	1	1	1
Bavarichthys_incognitus	1	1	0	0	?	1	0	1	1	1
Cavenderichthys_talbragarensis	0	1	0	0	0&1	1	0	1	0	0
Brycon_meeki	1	1	0	?	1	0	0	1	1	1
Chanos_chanos	?	1	0	0	1	0	0	0	1&2	2
Chongichthys_dentatus	1	1	?	?	?	?	?	?	?	?
Crossognathus_sabaudians	0	1	0	0	0	0	0	1	1	1
Denticeps_clupeoides	0	1	0	0	1	0	0	1	1	1
Diplomystus_dentatus	0	1	0	1	1	0	0	1	1	1
Domeykos_profetaensis	1	1	0	0	1	1	0	1	1	1
Dorsetichthys_bechei	?	1	0	0	0	0	0	1	0	0
Engraulis_ringens	?	1	0	?	?	0	0	1	1	2
Elops_saurus	2	1	0	0	0	0	0	1	2	2
Erichalcis_arcta	0&1	1	1	0	1	0	0	0	2	2
Esox_lucius	1&2	1	0	0	1	0	1	0	2	2
Eurycormus_speciosus	1	1	0	0	?	0	1	0	0	0
Gordichthys_conquensis	0	1	0	0	1	0	0	1	?	1

Taxon / Character number	125	126	127	128	129	130	131	132	133	134
Goulmimichthys_arambourgi	1	1	0	0	?	?	0	?	1	1
Hiodon_alosoides	1	1	0	0	1	1	0	2	1	1&2
Heterotis_niloticus	1&2	1	0	0	1	0	0	2	1	2
Humbertia_operta	0	1	0	0	0	0	1	0	2	2
Leptolepis_coryphaenoides	0	1	0	0	0	1	0	1	1	1
Leptolepis_koonwarri	1	1	0	0	1	1	0	1	0	0
Leptolepides_haerteisi	1	1	1	0	?	1	0	1	1	1
Leptolepides_sprattiformis	1	1	1	0	0&1	1	0	1	1	1
Luisichthysvinalaensis	?	1	0	0	0	0	0	1	2	2
Luisiella_feruglioi	0	1	0	0	0	1	0	1	0	0
Lycoptera_middendorfi	1	1	0	0	1	0	0	2	1	1&2
Megalops_atlanticus	1	1	0	0	1	?	0	1	1	2
Notelops_brama	1	1	0	0	0	1	0	1	2	1
Oncorhynchus_mykiss	2	1	1	1	1	0	1	0	1&2	2
Opsariichthys_bidens	0&1	1	0	0	1	0	0	1	1	2
Orthogonikleithrus_hoelli	0	1	1	1	1&2	1	0	1	1	2
Pachythrissops_propterus	1	1	0	0	1	1	0	2	1	2
Pholidophorus_gervasutti	?	1	?	?	?	?	?	?	?	?
Pholidophorus_latiusculus	?	?	?	?	?	?	?	?	?	?
Protoclupea_chilensis	1	1	0	0	0	1	0	1	2	2
Rhacolepis_buccalis	1	1	0	0	0	0	0	1	1	1
Santanaclupea_silvasantosi	0	1	0	0	1	0	0	1	1	1
Siemensichthys_macrocephalus	?	1	?	?	?	?	?	?	0	0
Siemensichthys_siemensi	?	?	?	?	?	?	?	?	?	?
Tharrhias_araripis_	1	1	0	0	1	0	0	?	0&2	0&2
Tharsis_dubius	1	1	0	0	0	1	0	1	2	2
Thrissops_formosus	1	1	0	0	0&1	1	0	1	2	2
Thymallus_thymallus	2	1	0	1	0	0	1	0	2	2
Umbra_krameri	0	1	0	0	1	0	0	0	1	2
Varasichthys_ariasi	1	1	?	0	1	0	0	2	1	2

Taxon / Character number	135	136	137	138	139	140	141	142	143	144
Amia_calva	0	0	0	0	1	0	0	1	4	-
Amia_pattersoni	0	0	0	0	1	0	0	1	4	-
Lepisosteus_osseus	0	0	0	0	0	0	1	0	4	-
Obaichthys_decoratus	0	0	0	?	0	0	1	?	4	-
Aspidorhynchus_acutirostris	0	0	1	1	0	0	0	4	3	0
Belonostomus_tenuirostris	0	0	1	1	0	0	0	4	3	0
Hypsocormus_macrodon	0	2	-	0	0	0	?	0	4	-
Mesturus_verrucosus	0	0	?	?	0	0	-	?	4	-
Pachycormus_macropterus	0	2	-	0	0	0	?	0	4	-
Vinctifer_comptoni	0	0	0	0	?	0	0	4	3	0
Apsopelix_anglicus	0	1	0	1	0	0	?	2&3	1	0
Allothrissops_mesogaster	0	1	0	0&1	0	0	0	1	0	0
Anaethalion_angustus	0	1	0	0	0	1	0	?	2	0
Anaethalion_knorri	0	1	?	1	0	?	0	1	2	?
Ascalabos_voithii	0	1	0	0	0	0	0	1	0	0
Bavarichthys_incognitus	0	1	0	1	0	0	0	1	2	0
Cavenderichthys_talbragarensis	0	1	0	0	0	0	0	1	1&2	0
Brycon_meeki	0	1	1	?	0	0	0	1	3	1
Chanos_chanos	1	1	0	?	0	0	0	3	3	?
Chongichthys_dentatus	?	?	?	?	0	?	?	?	?	?
Crossognathus_sabaudians	0	1	0	1	0	0	?	2&3	1	0
Denticeps_clupeoides	0	1	0	1	0	0	0	2	3	?
Diplomystus_dentatus	0	1	0	1	0	0	0	1	2	0
Domeykos_profetaensis	0	1	0	0	0	0	0	0	2	0
Dorsetichthys_bechei	0	?	0	0	0	0	0	0	0&1	0
Engraulis_ringens	1	1	1	0&1	0	0	0	2	3	1
Elops_saurus	1	1	0	0	0	1	0	1	3	0
Erichalcis_arcta	?	1	1	1	0	0	0	1	3	?
Esox_lucius	0	1	1	1	0	0	0	2	3	1
Eurycormus_speciosus	0	0	1	1	0	0	?	0	0	0
Gordichthys_conquensis	0	1	0	0	0	0	0	2	3	?

Taxon / Character number	135	136	137	138	139	140	141	142	143	144
Goulmimichthys_arambourgi	1	1	?	?	0	0	?	?	?	?
Hiodon_alosoides	0	1	0	0	0	0	0	3	3	0
Heterotis_niloticus	0	1	0	0	0	0	0	4	4	-
Humbertia_operta	1	1	1	1	0	0	0	1	2	1
Leptolepis_coryphaenoides	0	1	0	0	0	0	0	0&1	0	0
Leptolepis_koonwarri	0	1	?	?	0	0	0	1	1	0
Leptolepides_haerteisi	0	1	0	0	0	0	0	1	2	1
Leptolepides_sprattiformis	0	1	0	0	0	0	0	1	2	1
Luisichthysvinalaensis	0	1	0	0	0	0	0	1	2	0
Luisiella_feruglioi	0	1	0	?	0	0	0	1	1&2	0
Lycoptera_middendorfi	0	0&1	0	0	0	0	0	1	1	0
Megalops_atlanticus	1	1	0	0	0	1	0	1	3	0
Notelops_brama	0	1	1	1	0	0	0	2	?	?
Oncorhynchus_mykiss	1	1	1	1	0	0	0	1	3	1
Opsariichthys_bidens	1	1	0	0	0	0	0	3	3	1
Orthogonikleithrus_hoelli	0	1	1	1	0	0	0	1	3	1
Pachythrissops_propterus	0	1	0	0	0	0	0	1&2	1	0
Pholidophorus_gervasutti	?	?	?	?	?	?	?	?	?	?
Pholidophorus_latiusculus	?	?	?	?	?	?	?	?	?	?
Protoclupea_chilensis	0	1	0	?	0	0	0	1	1	0
Rhacolepis_buccalis	1	1	0	1	0	0	?	2	?	?
Santanaclupea_silvasantosi	?	1	1	1	0	0	?	?	?	?
Siemensichthys_macrocephalus	?	?	?	?	?	?	?	?	?	?
Siemensichthys_siemensi	?	?	?	?	?	?	?	?	?	?
Tharrhias_araripis_	0	1	?	?	0	0	0	2	3	0
Tharsis_dubius	1	1	0	0	0	0	0	1	0	1
Thrissops_formosus	0	1	0	0	0	0	0	1	0	0
Thymallus_thymallus	1	1	1	1	0	0	0	1	3	1
Umbra_krameri	0	1	1	1	?	0	0	2	3	1
Varasichthys_ariasi	0	1	0	?	?	0	0	3	0	0

Taxon / Character number	145	146	147	148	149	150	151	152	153	154
Amia_calva	-	-	-	-	-	0	0	1	0	0
Amia_pattersoni	-	-	-	-	-	0	0	1	0	0
Lepisosteus_osseus	-	-	-	-	-	0	0	1	0	0
Obaichthys_decoratus	-	-	-	-	-	0	?	-	-	0
Aspidorhynchus_acutirostris	2&3	0	0	0	0	1	2	1	0	0
Belonostomus_tenuirostris	1&3	0	0	0	0	1	2	1	0	0
Hypsocormus_macrodon	-	-	-	-	-	1	3	1	-	-
Mesturus_verrucosus	-	-	-	-	-	0	?	?	?	0
Pachycormus_macropterus	-	-	-	-	-	1	3	1	-	-
Vinctifer_comptoni	-	1	0	?	0	?	2	1	0	1
Apsopelix_anglicus	0	0	1	0	0	0	0	0	?	2
Allothrissops_mesogaster	0	1	1	0	0	0	0	0	0	0
Anaethalion_angustus	?	1	0	0	0	?	0&1	0	0	1
Anaethalion_knorri	1	1	0	0	0	0	?	0	0	1
Ascalabos_voithii	0	0	0	0	0	0	0	0	0&2	1
Bavarichthys_incognitus	1	1	0	0	0	0	2	0	2	2
Cavenderichthys_talbragarensis	1	0	0	0	0	0	0	0	0	1
Brycon_meeki	2	?	0	1	0	0	2	0	0	0
Chanos_chanos	2	?	0	1	0	0	2	0	0	0
Chongichthys_dentatus	?	?	?	?	?	?	?	0	?	?
Crossognathus_sabaudians	0	0	1	0	0	0	0	0	2	2
Denticeps_clupeoides	2	2	0	0	0	0	2	0	1	0
Diplomystus_dentatus	2	2	0	0	0	0	1	0	1	0
Domeykos_profetaensis	0	0	0	0	0	0	0	0	2	1
Dorsetichthys_bechei	3	?	0	0	0	0	0	1	0	0
Engraulis_ringens	2	2	0	1	0	0	?	0	1	0
Elops_saurus	1	1	0	0	0	0	1	0	0	0
Erichalcis_arcta	1	2	0	0	1	0	2	0	0	1
Esox_lucius	2	2	0	0	1	0	2	0	0	0
Eurycormus_speciosus	0&1	?	0	0	0	1	0	1	0	0
Gordichthys_conquensis	1	2	0	0	0	0	2	0	0	0

Taxon / Character number	145	146	147	148	149	150	151	152	153	154
Goulmimichthys_arambourgi	0	1	?	0	0	?	2	0	0	2
Hiodon_alosoides	1	1	0	0	0	0	1	0	0	0
Heterotis_niloticus	-	-	-	-	-	0	2	0	0	0
Humbertia_operta	1	1	0	0	1	0	2	0	0	0
Leptolepis_coryphaenoides	0	0	0	0	0	0	0	0	2	1
Leptolepis_koonwarri	1&2	1	0	0	0	0	0	0	0&2	0
Leptolepides_haerteisi	1	1	0	0	1	0	?	0	0&2	1
Leptolepides_sprattiformis	1	1	0	0	1	0	0&1	0	0	1
Luisichthysvinalaensis	0	1	0	0	0	0	0	0	1	?
Luisiella_feruglioi	1	0	0	0	0	0	0	0	0	0&1
Lycoptera_middendorfi	1	0	0	0	0	0	1	0&1	0	0
Megalops_atlanticus	1	1	0	0	0	0	1	0	?	0
Notelops_brama	2	1	0	0	0	0	?	0	2	2
Oncorhynchus_mykiss	1	2	0	0	1	0	2	0	0	0
Opsariichthys_bidens	2	2	0	1	0	0	2	0	?	0
Orthogonikleithrus_hoelli	1	1	0	0	1	0	2	0	0	1
Pachythrissops_propterus	1	0	0	0	0	0	0	0	0	0&1
Pholidophorus_gervasutti	?	?	?	?	?	?	?	?	?	?
Pholidophorus_latiusculus	?	?	?	?	?	?	?	?	?	?
Protoclupea_chilensis	0	1	0	0	0	0	0	0	0	?
Rhacolepis_buccalis	2	1	0	0	0	0	?	0	2	0&2
Santanaclupea_silvasantosi	2	2	0	0	0	0	2	0	1	0
Siemensichthys_macrocephalus	?	?	?	?	?	?	?	?	?	?
Siemensichthys_siemensi	?	?	?	?	?	?	?	?	?	?
Tharrhias_araripis_	1	2	0	0	0	0	2	0	?	0
Tharsis_dubius	0	0	0	0	0	0	0	0	0	1
Thrissops_formosus	0	0	1	?	0	0	0	0	0	0
Thymallus_thymallus	1	2	0	?	1	0	2	0	0	0
Umbra_krameri	2	2	0	?	1	0	2	0	0	0
Varasichthys_ariasi	0	0	0	?	0	0	?	0	0	0

Taxon / Character number	155	156	157	158	159	160	161	162	163	164
Amia_calva	-	0	0	1	1	1	1	2	1&2	0
Amia_pattersoni	-	0	0	1	1	1	1	2	2	0
Lepisosteus_osseus	-	0	0	1	0	0	0	0	2	2
Obaichthys_decoratus	-	0	?	1	?	0	0	0	?	?
Aspidorhynchus_acutirostris	-	0	1	0	0	0	0&1	0	0&2	0
Belonostomus_tenuirostris	-	1	1	0	0	0	0	0	1&2	2
Hypsocormus_macrodon	-	-	-	0	0	0	0	0	0	0
Mesturus_verrucosus	-	?	1	1	?	1	0	2	0	0
Pachycormus_macropterus	-	-	-	0	0	0	0	0	0	0
Vinctifer_comptoni	-	0	1	0	0	1	1	?	0	0
Apsopelix_anglicus	0	0	?	1	1	1	1	?	1	0
Allothrissops_mesogaster	0	1	1	0	1	1	1	2	1	0
Anaethalion_angustus	?	1	1	0	1	1	0	1	1	0
Anaethalion_knorri	0	1	1	0	1	1	0	1	1	0
Ascalabos_voithii	0	1	1	0	0	1	1	2	1	0
Bavarichthys_incognitus	0	1	1	0	1	1	0	1	1	0
Cavenderichthys_talbragarensis	0	1	1	0	0	1	0	1	1	0
Brycon_meeki	1	1	1	0	1	1	1	2	1	0
Chanos_chanos	1	1	1	0	1	?	1	2	1	0
Chongichthys_dentatus	?	?	?	?	?	1	?	?	?	?
Crossognathus_sabaudians	0	0	1	0	1	1	1	?	1	0
Denticeps_clupeoides	0	1	1	1	1	?	1	2	2	0
Diplomystus_dentatus	0	1	1	0	1	1	1	2	1	0
Domeykos_profetaensis	0	1	1	?	0	1	1	?	0	0
Dorsetichthys_bechei	0	0	1	0	0	0	0	0	0	0
Engraulis_ringens	0	1	1	1	1	1	1	2	1	0
Elops_saurus	0	1	1	0	1	?	1	2	1	0
Erichalcis_arcta	0	0	1	0	1	1	1	2	1	0
Esox_lucius	0	1	1	1	1	?	1	2	1	0
Eurycormus_speciosus	0	1	1	0	0	0	0	0	0	0
Gordichthys_conquensis	0	1	1	1	1	1	1	2	1	0

Taxon / Character number	155	156	157	158	159	160	161	162	163	164
Goulmimichthys_arambourgi	0	1	1	0	?	1	?	?	?	?
Hiodon_alosoides	0	1	1	1	1	1	1	2	2	1
Heterotis_niloticus	0	1	1	1	1	1	1	2	2	2
Humbertia_operta	1	1	1	0	1	?	1	2	1	0
Leptolepis_coryphaenoides	0	0	1	0	0	1	0	0	1	0
Leptolepis_koonwarri	0	0	1	0	0	1	?	?	1	0
Leptolepides_haerteisi	0	0	1	0	1	1	1	2	1	0
Leptolepides_sprattiformis	0	0	1	0	1	1	1	2	1	0
Luisichthysvinalaensis	0	1	1	0	0	?	1	1	0	0
Luisiella_feruglioi	0	0	1	0	0	1	0	1	1	0
Lycoptera_middendorfi	0	1	1	1	1	1	1	?	2	1
Megalops_atlanticus	0	1	1	0	1	?	1	?	1	0
Notelops_brama	0	0	1	0	1	1	1	2	1	0
Oncorhynchus_mykiss	0	1	1	1	1	1	1	2	1	0
Opsariichthys_bidens	1	1	1	0	1	1	1	2	1	0
Orthogonikleithrus_hoelli	0	0	1	0	1	1	1	2	1	0
Pachythrissops_propterus	0	1	1	0	0	1	0	0	1	0
Pholidophorus_gervasutti	?	?	?	0	0	0	0	0	0	0
Pholidophorus_latiusculus	?	?	?	0	0	0	0	0	0	0
Protoclupea_chilensis	0	1	1	0	1	1	1	2	0	0
Rhacolepis_buccalis	0	0	1	0	1	1	1	2	1	0
Santanaclupea_silvasantosi	0	1	1	0	1	1	1	2	1	0
Siemensichthys_macrocephalus	?	?	?	0	0	0	0	?	?	?
Siemensichthys_siemensi	?	?	?	0	?	0	0	0	0	0
Tharrhias_araripis_	0	1	1	0	?	1	?	?	1	?
Tharsis_dubius	0	0	1	0	0	1	0	1	1	0
Thrissops_formosus	?	1	1	0	1	1	1	2	1	0
Thymallus_thymallus	?	1	1	1	1	?	1	2	1	0
Umbra_krameri	?	1	1	0	1	?	1	2	2	2
Varasichthys_ariasi	?	1	1	0	0	1	1	1	0	0

Taxon / Character number	165	166	167	168	169	170	171	172	173	174
Amia_calva	-	0	1	0	0	1	2	1	0	2
Amia_pattersoni	-	0	1	0	0	1	2	1	0	2
Lepisosteus_osseus	-	2	1	0	0	1	2	1	0	2
Obaichthys_decoratus	-	?	1	?	?	1	2	1	?	2
Aspidorhynchus_acutirostris	0&2	0	0	0	0	0	1	0	0	2
Belonostomus_tenuirostris	?	0	0	0	0	0	1	0	0	2
Hypsocormus_macrodon	0	0	-	1	?	0	?	0	0	2
Mesturus_verrucosus	0	1	?	?	?	0	?	0	?	?
Pachycormus_macropterus	0	0	-	1	0	0	?	0	0	2
Vinctifer_comptoni	0	0	0	0	0	0	1	0	0	2
Apsopelix_anglicus	1	1	1	1	1	0	1	0	0	0
Allothrissops_mesogaster	1	1	0	1	1	0	1	0	0	0&1
Anaethalion_angustus	1	1	1	0	0	0	1	0	0	1
Anaethalion_knorri	1	1	1	0	0	0	1	0	0	1
Ascalabos_voithii	1	1	0	1	1	0	1	0	0	0
Bavarichthys_incognitus	1	1	0	0	0	0	1	0	0	0
Cavenderichthys_talbragarensis	1	1	0	1	1	0	1	0	0	0
Brycon_meeki	1	1	1	0	?	1	?	0	0	2
Chanos_chanos	1	1	0	0	?	0	?	0	0	2
Chongichthys_dentatus	?	?	?	?	?	?	1	0	?	?
Crossognathus_sabaudians	1	1	1	1	1	0	1	0	0	0
Denticeps_clupeoides	1	1	1	0	0	1	?	0	0	2
Diplomystus_dentatus	1	1	1	0	0	?	1	0	0	2
Domeykos_profetaensis	0	1	0	1	1	0	1	0	0	?
Dorsetichthys_bechei	0	0	0	1	1	0	1	0	0	2
Engraulis_ringens	1	1	1	0	?	1	?	0	0	2
Elops_saurus	1	1	1	0	1	0	1	0	0	1
Erichalcis_arcta	1	1	1	1	?	1	?	0	0	1
Esox_lucius	1	1	1	0	0	1	1	0	0	2
Eurycormus_speciosus	0	0	0	1	?	0	?	0	1	2
Gordichthys_conquensis	1	1	0	?	?	1	?	0	0	2

Taxon / Character number	165	166	167	168	169	170	171	172	173	174
Goulmimichthys_arambourgi	?	?	?	?	?	?	1	0	0	?
Hiodon_alosoides	2	1	1	0	0	0	1	0	0	2
Heterotis_niloticus	2	1	1	0	?	1	?	0	0	2
Humbertia_operta	1	1	1	0	0	1	1	0	0	1
Leptolepis_coryphaenoides	1	1	0	1	1	0	1	0	0	0
Leptolepis_koonwarri	1	1	0	?	?	0	1	0	0	?
Leptolepides_haerteisi	1	1	1	1	0	1	1	0	0	1
Leptolepides_sprattiformis	1	1	0	1	0	1	1	0	0	1
Luisichthysvinalaensis	0	1	0	?	1	0	1	0	0	?
Luisiella_feruglioi	1	1	0	1	1	0	1	0	0	0
Lycoptera_middendorfi	2	1	1	0	0	0	1	0	0	2
Megalops_atlanticus	1	1	1	0	?	0	?	0	0	1
Notelops_brama	1	1	0	?	0	0	0	0	0	?
Oncorhynchus_mykiss	1	1	1	0	0	1	1	0	0	2
Opsariichthys_bidens	1	1	1	0	?	0	?	0	0	2
Orthogonikleithrus_hoelli	1	1	1	0	1	1	1	0	0	1
Pachythrissops_propterus	1	1	0	1	1	0	1	0	0	1
Pholidophorus_gervasutti	0	0	?	0	?	1	?	0	0	2
Pholidophorus_latiusculus	?	0	?	0	?	1	?	0	?	2
Protoclupea_chilensis	0	1	?	?	0	0	1	0	?	?
Rhacolepis_buccalis	1	1	1	?	1	0	0	0	0	?
Santanaclupea_silvasantosi	1	1	1	?	0	?	0	0	0	?
Siemensichthys_macrocephalus	?	?	?	?	?	0	?	0	?	?
Siemensichthys_siemensi	?	?	?	?	0	0	?	0	?	?
Tharrhias_araripis_	1	1	?	?	?	0	0	0	0	?
Tharsis_dubius	1	1	0	1	1	0	1	0	0	0
Thrissops_formosus	1	1	0	1	1	0	1	0	0	1
Thymallus_thymallus	1	1	1	0	?	1	?	0	0	2
Umbra_krameri	2	1	1	0	?	1	1	1	0	2
Varasichthys_ariasi	0	1	0	?	1	0	1	0	0	0

Taxon / Character number	175	176	177	178
Amia_calva	2	-	-	0
Amia_pattersoni	2	-	-	?
Lepisosteus_osseus	0	-	-	0
Obaichthys_decoratus	0	-	-	?
Aspidorhynchus_acutirostris	0	-	-	?
Belonostomus_tenuirostris	0	-	-	?
Hypsocormus_macrodon	2	-	-	?
Mesturus_verrucosus	0	-	-	0
Pachycormus_macropterus	2	-	-	?
Vinctifer_comptoni	0	-	-	?
Apsopelix_anglicus	1	?	0	?
Allothrissops_mesogaster	1	0	1	0
Anaethalion_angustus	1	1	0	1
Anaethalion_knorri	1	1	0	1
Ascalabos_voithii	1	0	0	0
Bavarichthys_incognitus	?	?	?	?
Cavenderichthys_talbragarensis	1	0	0	?
Brycon_meeki	1	0	0	0
Chanos_chanos	1	1	0	0
Chongichthys_dentatus	1	0	0	?
Crossognathus_sabaudians	1	0	0	?
Denticeps_clupeoides	1	?	0	0
Diplomystus_dentatus	1	0	0	0
Domeykos_profetaensis	1	1	1	?
Dorsetichthys_bechei	0	-	-	?
Engraulis_ringens	1	0	0	0
Elops_saurus	1	1	0	1
Erichalcis_arcta	?	0	0	?
Esox_lucius	1	0	0	0
Eurycormus_speciosus	2	1	0	?
Gordichthys_conquensis	1	?	0	0

Taxon / Character number	175	176	177	178
Goulmimichthys_arambourgi	1	?	0	?
Hiodon_alosoides	1	0	2	0
Heterotis_niloticus	1	0	2	0
Humbertia_operta	1	0	0	0
Leptolepis_coryphaenoides	1	0	0	0
Leptolepis_koonwarri	1	?	0	?
Leptolepides_haerteisi	1	?	0	?
Leptolepides_sprattiformis	1	1	0	0
Luisichthysvinalaensis	1	1	1	?
Luisiella_feruglioi	1	0	0	?
Lycoptera_middendorfi	1	0	2	0
Megalops_atlanticus	1	1	0	1
Notelops_brama	1	0	0	?
Oncorhynchus_mykiss	1	0	0	0
Opsariichthys_bidens	1	1	0	0
Orthogonikleithrus_hoelli	?	?	?	?
Pachythrissops_propterus	1	1	0	?
Pholidophorus_gervasutti	0	-	-	?
Pholidophorus_latiusculus	0	-	-	?
Protoclupea_chilensis	1	1	1	?
Rhacolepis_buccalis	1	?	1	?
Santanaclupea_silvasantosi	1	?	0	?
Siemensichthys_macrocephalus	0	-	-	?
Siemensichthys_siemensi	0	-	-	?
Tharrhias_araripis_	1	0	0	?
Tharsis_dubius	1	0	0	0
Thrissops_formosus	1	?	0	0
Thymallus_thymallus	1	0	0	0
Umbra_krameri	1	0	0	0
Varasichthys_ariasi	1	1	1	0

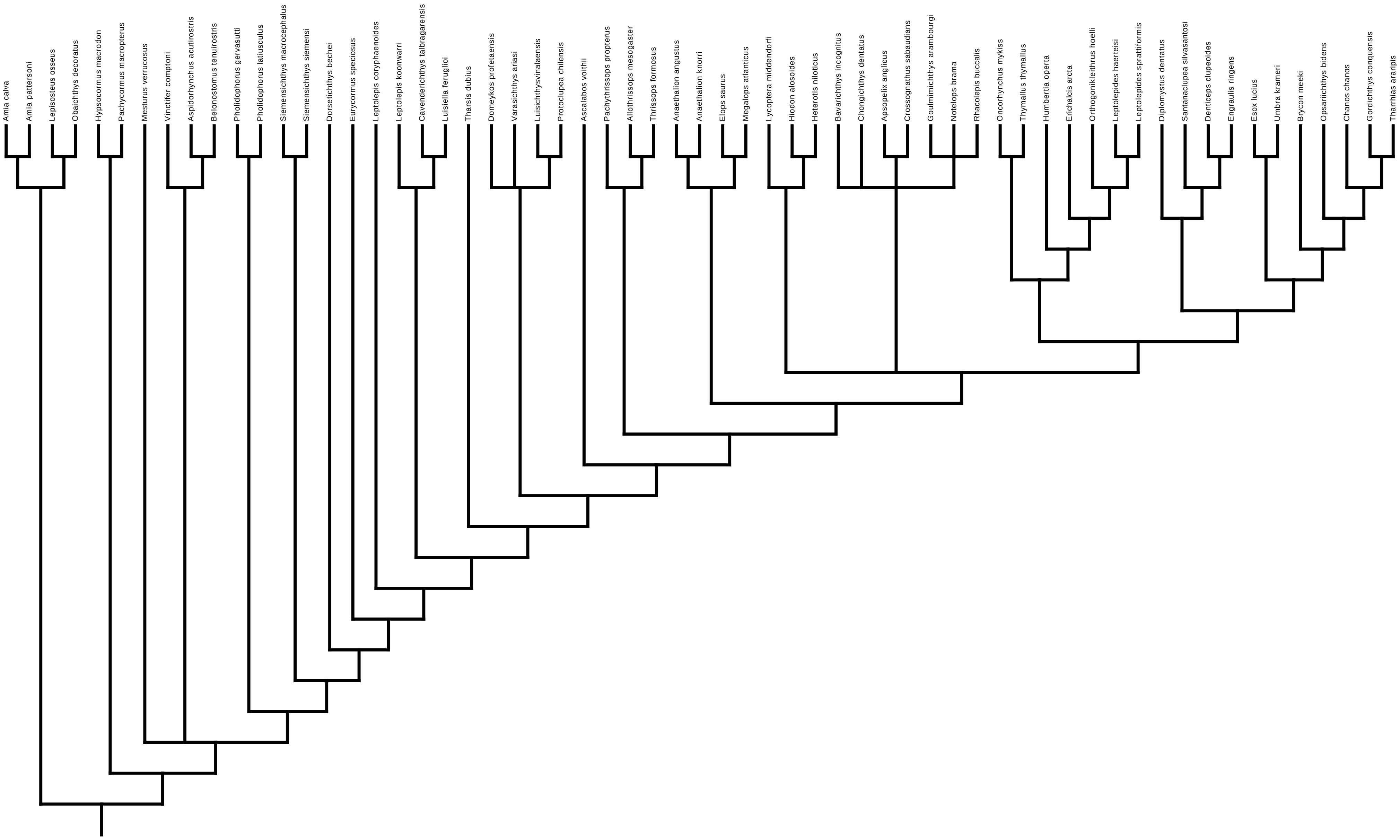

Strict Consensus Tree

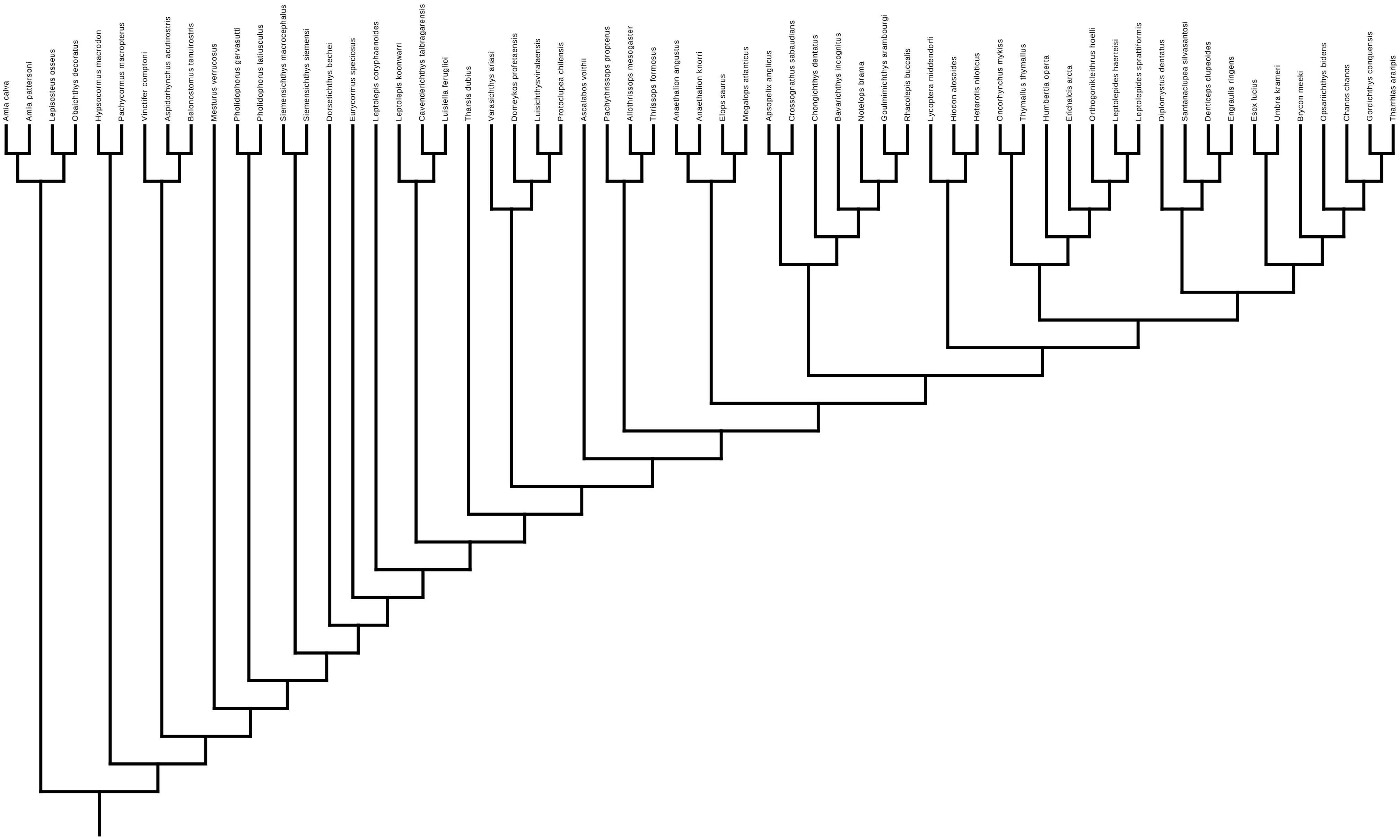
Amia calva
Amia pattersoni
Lepisosteus osseus
Obaichthys decoratus
Hypsocormus macrodon
Pachycormus macropterus
Vinctifer comptoni
Aspidorhynchus acutirostris
Belonostomus tenuirostris
Mesturus verrucosus
Pholidophorus gervasutti
Pholidophorus latiusculus
Siemensichthys macrocephalus
Siemensichthys siemensi
Dorsetichthys bechei
Eurycormus speciosus
Leptolepis coryphaenoides
Leptolepis koonwarri
Cavenderichthys talbragarensis
Luisiella feruglioi
Tharsis dubius
Varasichthys ariasi
Domeykos profetaensis
Luisichthysvinalaensis
Protoclupea chilensis
Ascalabos voithii
Pachythrissops propterus
Allothrissops mesogaster
Thrissops formosus
Anaethalion angustus
Anaethalion knorri
Elops saurus
Megalops atlanticus
Apsopelix anglicus
Crossognathus sabaudians
Chongichthys dentatus
Bavarichthys incognitus
Notelops brama
Goulmimichthys arambourgi
Rhacolepis buccalis
Lycoptera middendorfi
Hiodon alosoides
Heterotis niloticus
Oncorhynchus mykiss
Thymallus thymallus
Humbertia operta
Erichalcis arcta
Orthogonikleithrus hoelli
Leptolepides haerteisi
Leptolepides sprattiformis
Diplomystus dentatus
Santanaclupea silvasantosi
Denticeps clupeoides
Engraulis ringens
Esox lucius
Umbra krameri
Brycon meeki
Opsariichthys bidens
Chanos chanos
Gordichthys conquensis
Tharrhias araripis

MPT 1

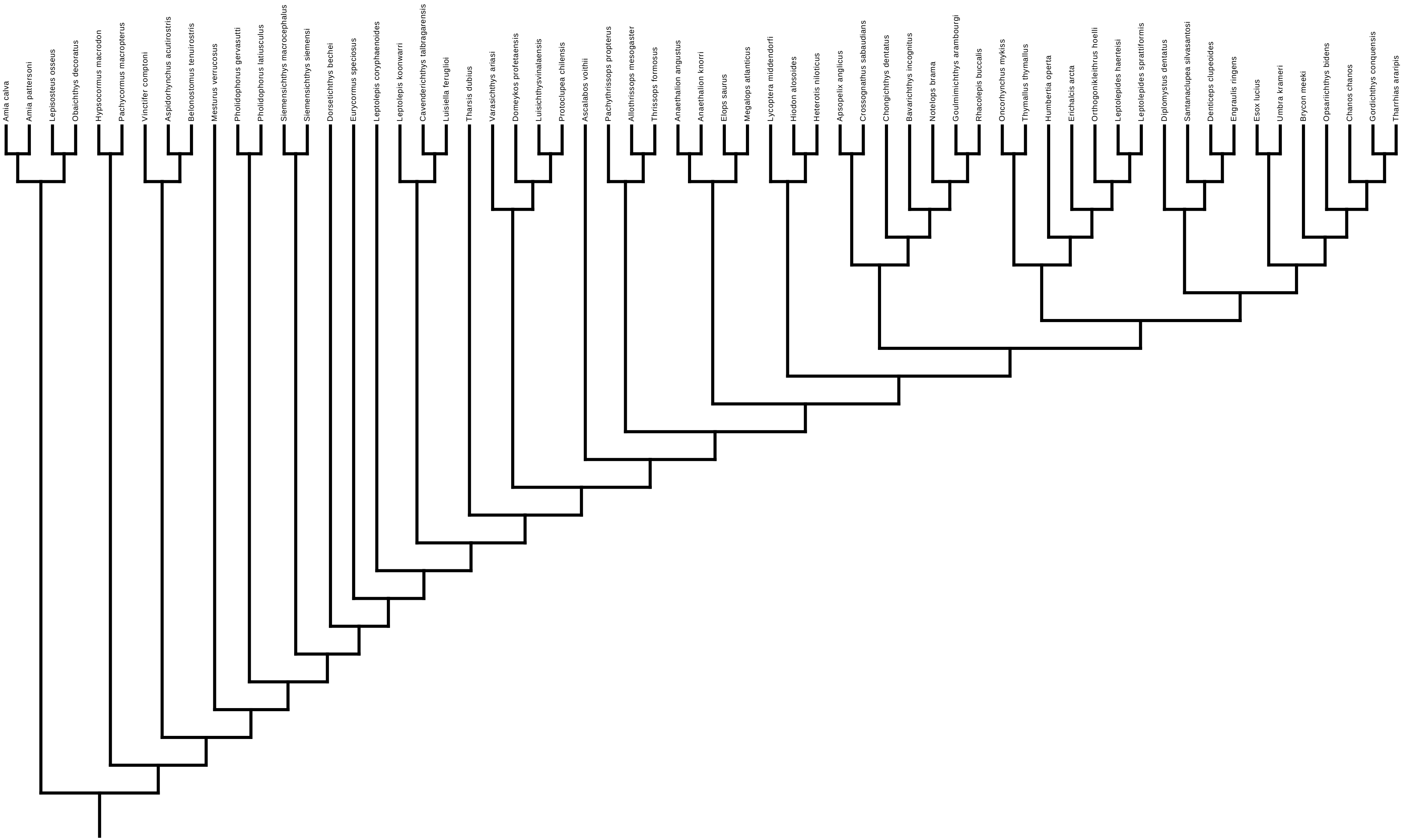
Amia calva
Amia pattersoni
Lepisosteus osseus
Obaichthys decoratus
Hypsocormus macrodon
Pachycormus macropterus
Vinctifer comptoni
Aspidorhynchus acutirostris
Belonostomus tenuirostris
Mesturus verrucosus
Pholidophorus gervasutti
Pholidophorus latiusculus
Siemensichthys macrocephalus
Siemensichthys siemensi
Dorsetichthys bechei
Eurycormus speciosus
Leptolepis coryphaenoides
Leptolepis koonwarri
Cavenderichthys talbragarensis
Luisiella feruglioi
Tharsis dubius
Varasichthys ariasi
Domeykos profetaensis
Luisichthysvinalaensis
Protoclupea chilensis
Ascalabos voithii
Pachythrissops propterus
Allothrissops mesogaster
Thrissops formosus
Anaethalion angustus
Anaethalion knorri
Elops saurus
Megalops atlanticus
Lycoptera middendorfi
Hiodon alosoides
Heterotis niloticus
Apsopelix anglicus
Crossognathus sabaudians
Chongichthys dentatus
Bavarichthys incognitus
Notelops brama
Goulmimichthys arambourgi
Rhacolepis buccalis
Oncorhynchus mykiss
Thymallus thymallus
Humbertia operta
Erichalcis arcta
Orthogonikleithrus hoelli
Leptolepides haerteisi
Leptolepides sprattiformis
Diplomystus dentatus
Santanaclupea silvasantosi
Denticeps clupeoides
Engraulis ringens
Esox lucius
Umbra krameri
Brycon meeki
Opsariichthys bidens
Chanos chanos
Gordichthys conquensis
Tharrhias araripis

MPT 2

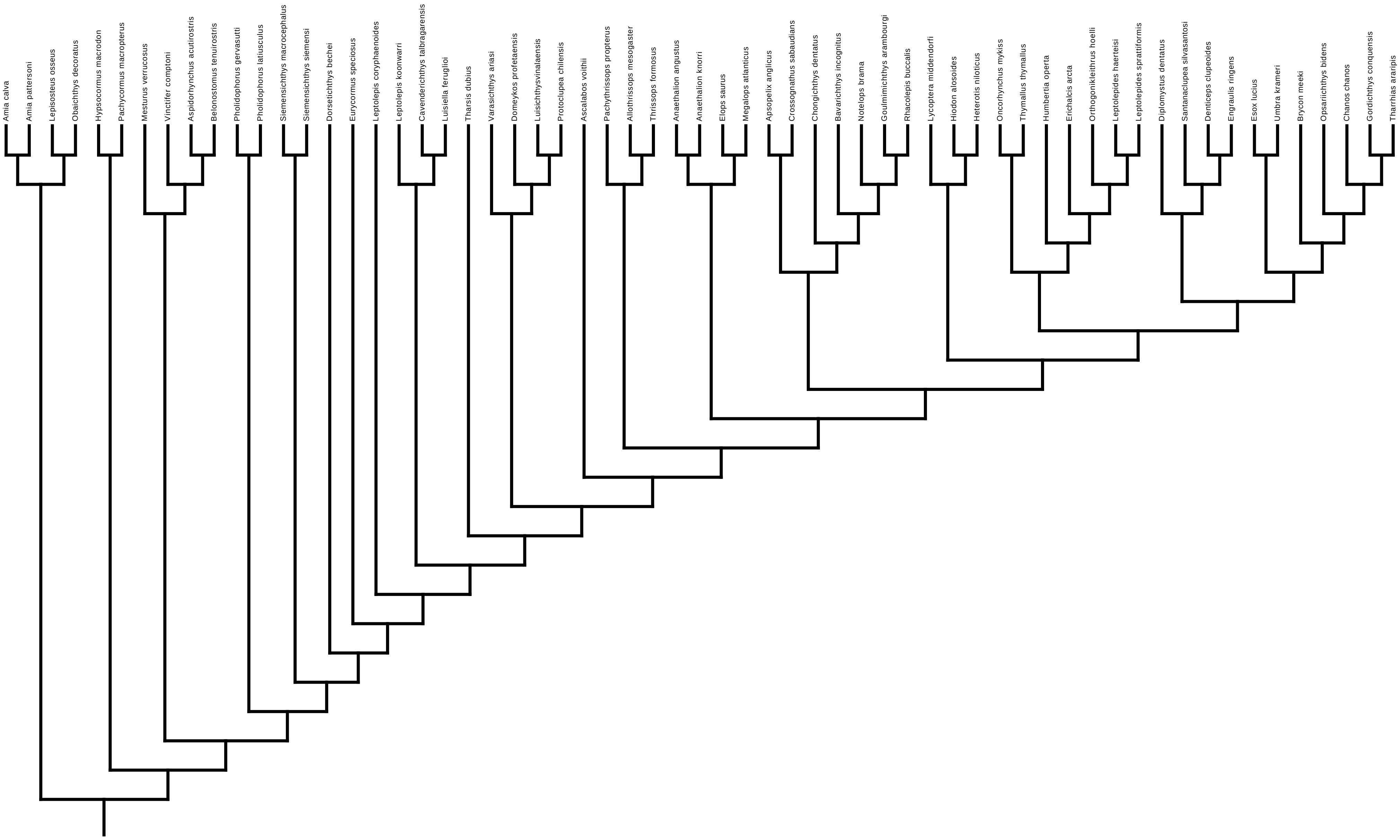

Amia calva
Amia pattersoni
Lepisosteus osseus
Obaichthys decoratus
Hypsocormus macrodon
Pachycormus macropterus
Mesturus verrucosus
Vinctifer comptoni
Aspidorhynchus acutirostris
Belonostomus tenuirostris
Pholidophorus gervasutti
Pholidophorus latiusculus
Siemensichthys macrocephalus
Siemensichthys siemensi
Dorsetichthys bechei
Eurycormus speciosus
Leptolepis coryphaenoides
Leptolepis koonwarri
Cavenderichthys talbragarensis
Luisiella feruglioi
Tharsis dubius
Varasichthys ariasi
Domeykos profetaensis
Luisichthysvinalaensis
Protoclupea chilensis
Ascalabos voithii
Pachythrissops propterus
Allothrissops mesogaster
Thrissops formosus
Anaethalion angustus
Anaethalion knorri
Elops saurus
Megalops atlanticus
Apsopelix anglicus
Crossognathus sabaudians
Chongichthys dentatus
Bavarichthys incognitus
Notelops brama
Goulmimichthys arambourgi
Rhacolepis buccalis
Lycoptera middendorfi
Hiodon alosoides
Heterotis niloticus
Oncorhynchus mykiss
Thymallus thymallus
Humbertia operta
Erichalcis arcta
Orthogonikleithrus hoelli
Leptolepides haerteisi
Leptolepides sprattiformis
Diplomystus dentatus
Santanaclupea silvasantosi
Denticeps clupeoides
Engraulis ringens
Esox lucius
Umbra krameri
Brycon meeki
Opsariichthys bidens
Chanos chanos
Gordichthys conquensis
Tharrhias araripis

MPT 3

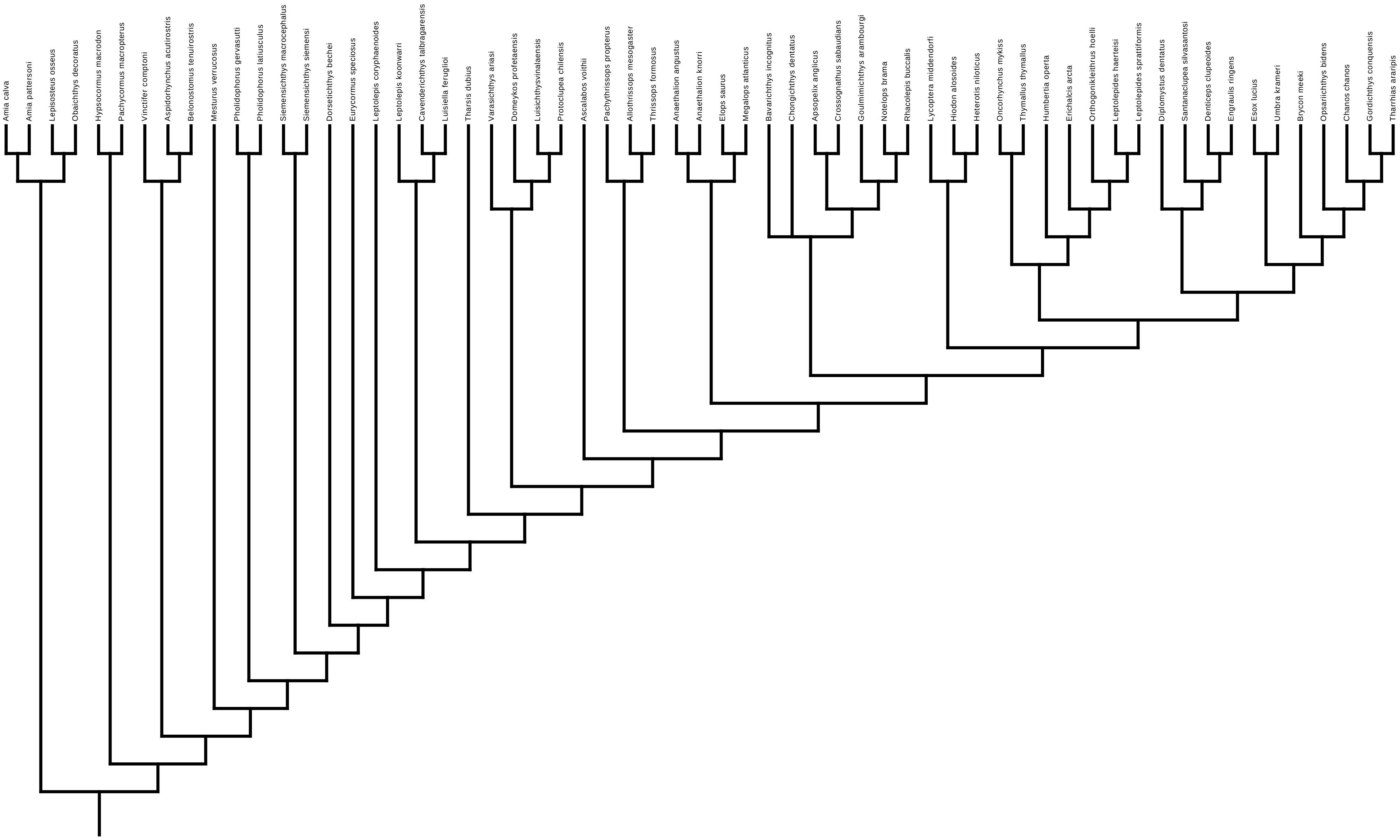

Amia calva
Amia pattersoni
Lepisosteus osseus
Obaichthys decoratus
Hypsocormus macrodon
Pachycormus macropterus
Vinctifer comptoni
Aspidorhynchus acutirostris
Belonostomus tenuirostris
Mesturus verrucosus
Pholidophorus gervasutti
Pholidophorus latiusculus
Siemensichthys macrocephalus
Siemensichthys siemensi
Dorsetichthys bechei
Eurycormus speciosus
Leptolepis coryphaenoides
Leptolepis koonwarri
Cavenderichthys talbragarensis
Luisiella feruglioi
Tharsis dubius
Varasichthys ariasi
Domeykos profetaensis
Luisichthysvinalaensis
Protoclupea chilensis
Ascalabos voithii
Pachythrissops propterus
Allothrissops mesogaster
Thrissops formosus
Anaethalion angustus
Anaethalion knorri
Elops saurus
Megalops atlanticus
Bavarichthys incognitus
Chongichthys dentatus
Apsopelix anglicus
Crossognathus sabaudians
Goulmimichthys arambourgi
Notelops brama
Rhacolepis buccalis
Lycoptera middendorfi
Hiodon alosoides
Heterotis niloticus
Oncorhynchus mykiss
Thymallus thymallus
Humbertia operta
Erichalcis arcta
Orthogonikleithrus hoelli
Leptolepides haerteisi
Leptolepides sprattiformis
Diplomystus dentatus
Santanaclupea silvasantosi
Denticeps clupeoides
Engraulis ringens
Esox lucius
Umbra krameri
Brycon meeki
Opsariichthys bidens
Chanos chanos
Gordichthys conquensis
Tharrhias araripis

MPT 4

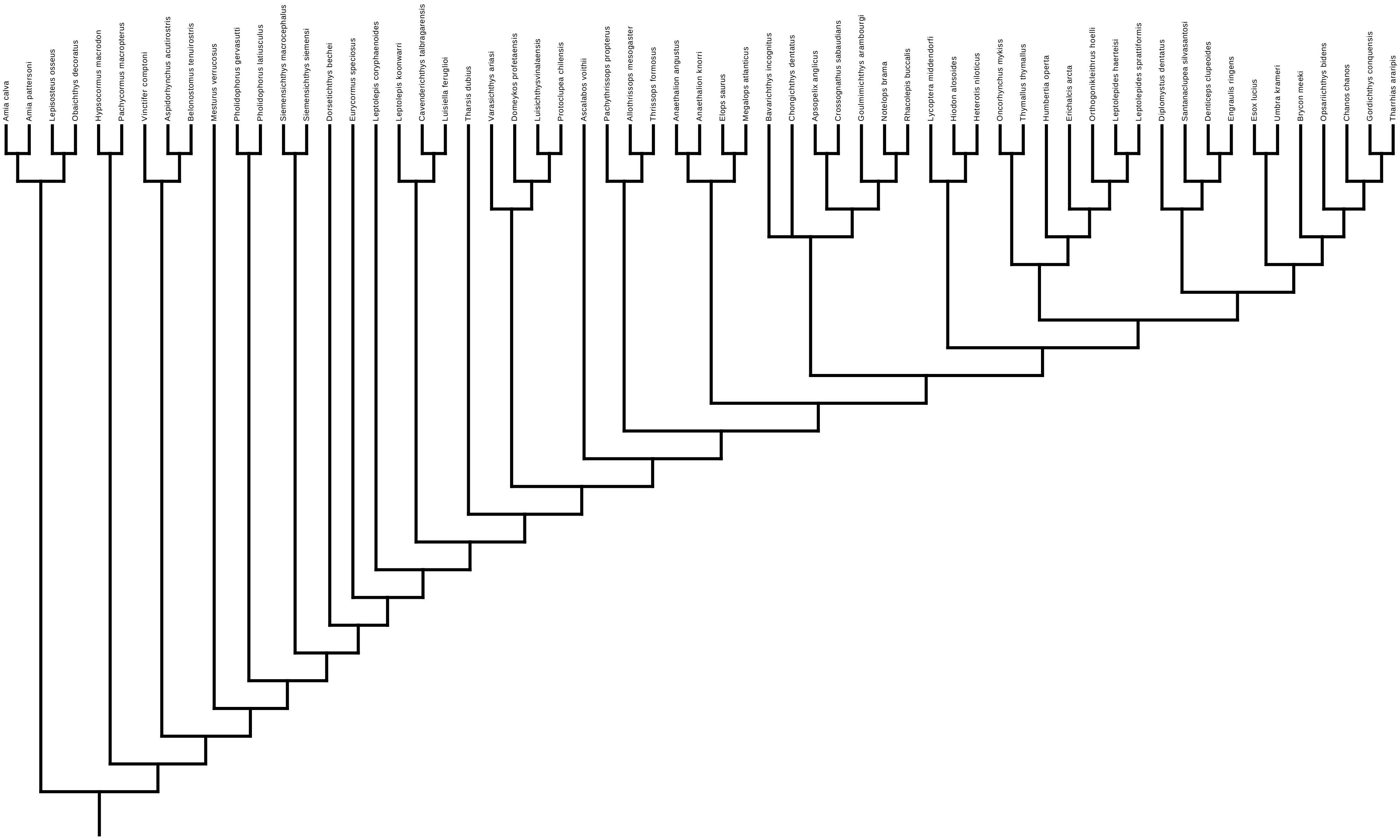
Amia calva
Amia pattersoni
Lepisosteus osseus
Obaichthys decoratus
Hypsocormus macrodon
Pachycormus macropterus
Vinctifer comptoni
Aspidorhynchus acutirostris
Belonostomus tenuirostris
Mesturus verrucosus
Pholidophorus gervasutti
Pholidophorus latiusculus
Siemensichthys macrocephalus
Siemensichthys siemensi
Dorsetichthys bechei
Eurycormus speciosus
Leptolepis coryphaenoides
Leptolepis koonwarri
Cavenderichthys talbragarensis
Luisiella feruglioi
Tharsis dubius
Varasichthys ariasi
Domeykos profetaensis
Luisichthysvinalaensis
Protoclupea chilensis
Ascalabos voithii
Pachythrissops propterus
Allothrissops mesogaster
Thrissops formosus
Anaethalion angustus
Anaethalion knorri
Elops saurus
Megalops atlanticus
Bavarichthys incognitus
Chongichthys dentatus
Apsopelix anglicus
Crossognathus sabaudians
Goulmimichthys arambourgi
Notelops brama
Rhacolepis buccalis
Lycoptera middendorfi
Hiodon alosoides
Heterotis niloticus
Oncorhynchus mykiss
Thymallus thymallus
Humbertia operta
Erichalcis arcta
Orthogonikleithrus hoelli
Leptolepides haerteisi
Leptolepides sprattiformis
Diplomystus dentatus
Santanaclupea silvasantosi
Denticeps clupeoides
Engraulis ringens
Esox lucius
Umbra krameri
Brycon meeki
Opsariichthys bidens
Chanos chanos
Gordichthys conquensis
Tharrhias araripis

MPT 5

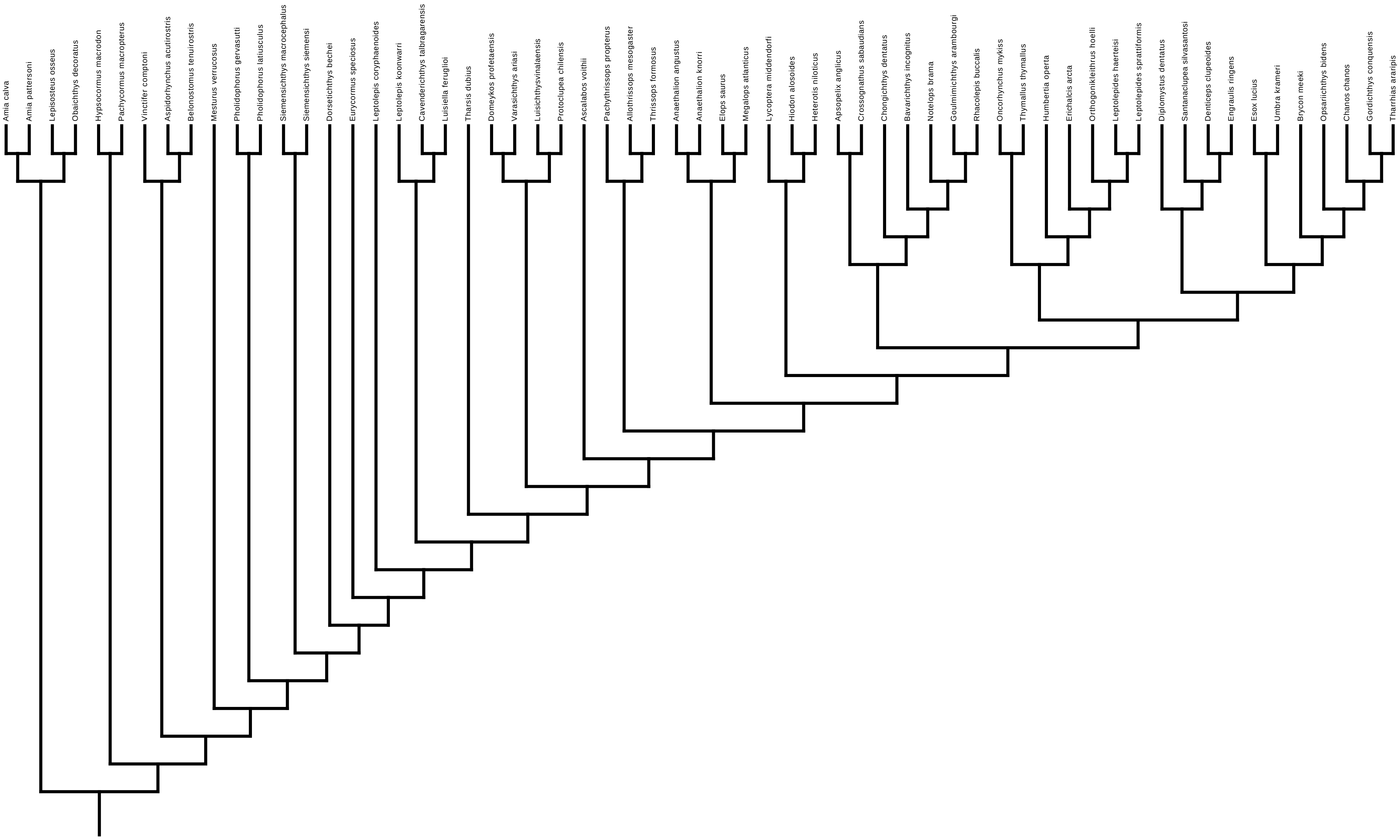
Amia calva
Amia pattersoni
Lepisosteus osseus
Obaichthys decoratus
Hypsocormus macrodon
Pachycormus macropterus
Vinctifer comptoni
Aspidorhynchus acutirostris
Belonostomus tenuirostris
Mesturus verrucosus
Pholidophorus gervasutti
Pholidophorus latiusculus
Siemensichthys macrocephalus
Siemensichthys siemensi
Dorsetichthys bechei
Eurycormus speciosus
Leptolepis coryphaenoides
Leptolepis koonwarri
Cavenderichthys talbragarensis
Luisiella feruglioi
Tharsis dubius
Domeykos profetaensis
Varasichthys ariasi
Luisichthysvinalaensis
Protoclupea chilensis
Ascalabos voithii
Pachythrissops propterus
Allothrissops mesogaster
Thrissops formosus
Anaethalion angustus
Anaethalion knorri
Elops saurus
Megalops atlanticus
Lycoptera middendorfi
Hiodon alosoides
Heterotis niloticus
Apsopelix anglicus
Crossognathus sabaudians
Chongichthys dentatus
Bavarichthys incognitus
Notelops brama
Goulmimichthys arambourgi
Rhacolepis buccalis
Oncorhynchus mykiss
Thymallus thymallus
Humbertia operta
Erichalcis arcta
Orthogonikleithrus hoelli
Leptolepides haerteisi
Leptolepides sprattiformis
Diplomystus dentatus
Santanaclupea silvasantosi
Denticeps clupeoides
Engraulis ringens
Esox lucius
Umbra krameri
Brycon meeki
Opsariichthys bidens
Chanos chanos
Gordichthys conquensis
Tharrhias araripis

MPT 6

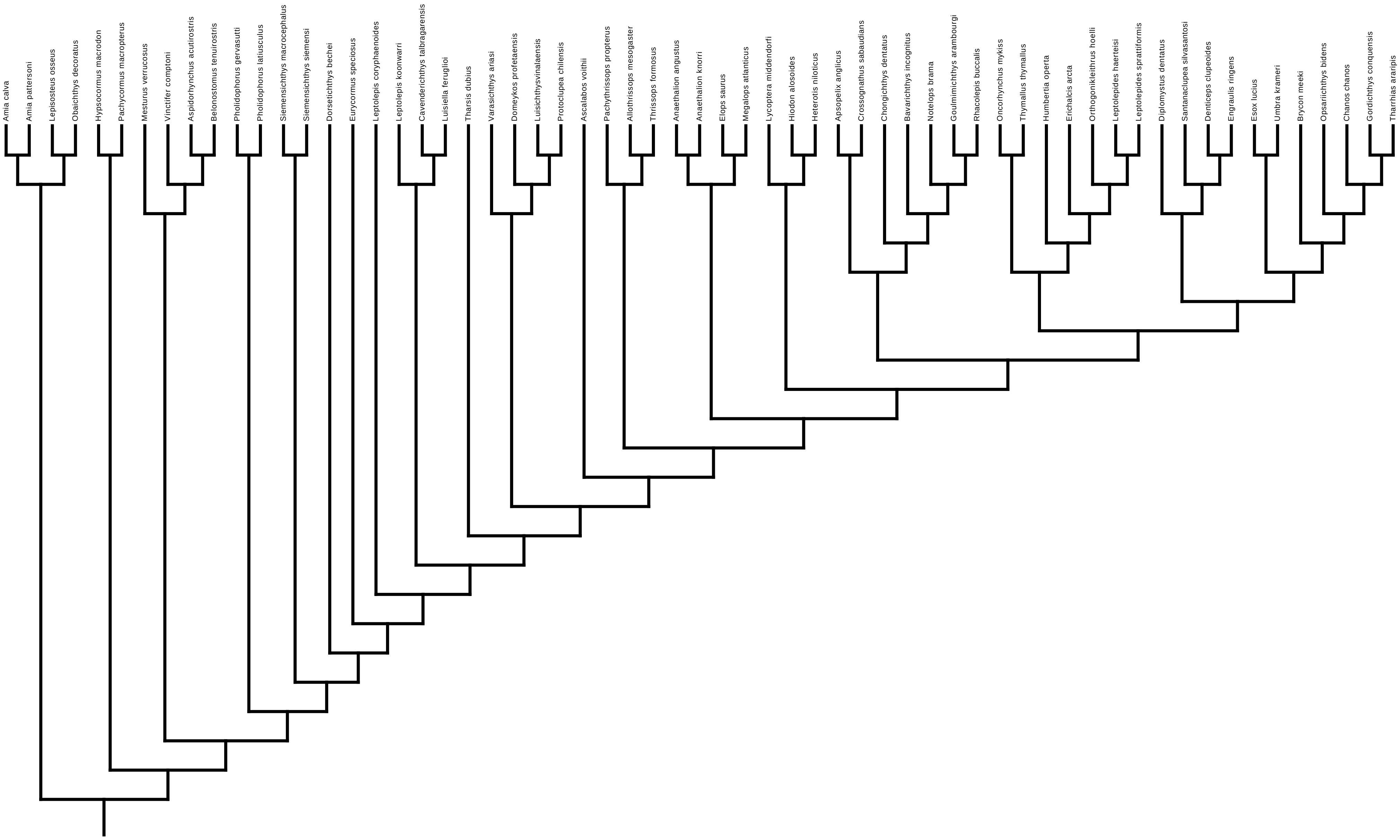
Amia calva
Amia pattersoni
Lepisosteus osseus
Obaichthys decoratus
Hypsocormus macrodon
Pachycormus macropterus
Mesturus verrucosus
Vinctifer comptoni
Aspidorhynchus acutirostris
Belonostomus tenuirostris
Pholidophorus gervasutti
Pholidophorus latiusculus
Siemensichthys macrocephalus
Siemensichthys siemensi
Dorsetichthys bechei
Eurycormus speciosus
Leptolepis coryphaenoides
Leptolepis koonwarri
Cavenderichthys talbragarensis
Luisiella feruglioi
Tharsis dubius
Varasichthys ariasi
Domeykos profetaensis
Luisichthysvinalaensis
Protoclupea chilensis
Ascalabos voithii
Pachythrissops propterus
Allothrissops mesogaster
Thrissops formosus
Anaethalion angustus
Anaethalion knorri
Elops saurus
Megalops atlanticus
Lycoptera middendorfi
Hiodon alosoides
Heterotis niloticus
Apsopelix anglicus
Crossognathus sabaudians
Chongichthys dentatus
Bavarichthys incognitus
Notelops brama
Goulmimichthys arambourgi
Rhacolepis buccalis
Oncorhynchus mykiss
Thymallus thymallus
Humbertia operta
Erichalcis arcta
Orthogonikleithrus hoelli
Leptolepides haerteisi
Leptolepides sprattiformis
Diplomystus dentatus
Santanaclupea silvasantosi
Denticeps clupeoides
Engraulis ringens
Esox lucius
Umbra krameri
Brycon meeki
Opsariichthys bidens
Chanos chanos
Gordichthys conquensis
Tharrhias araripis

MPT 7

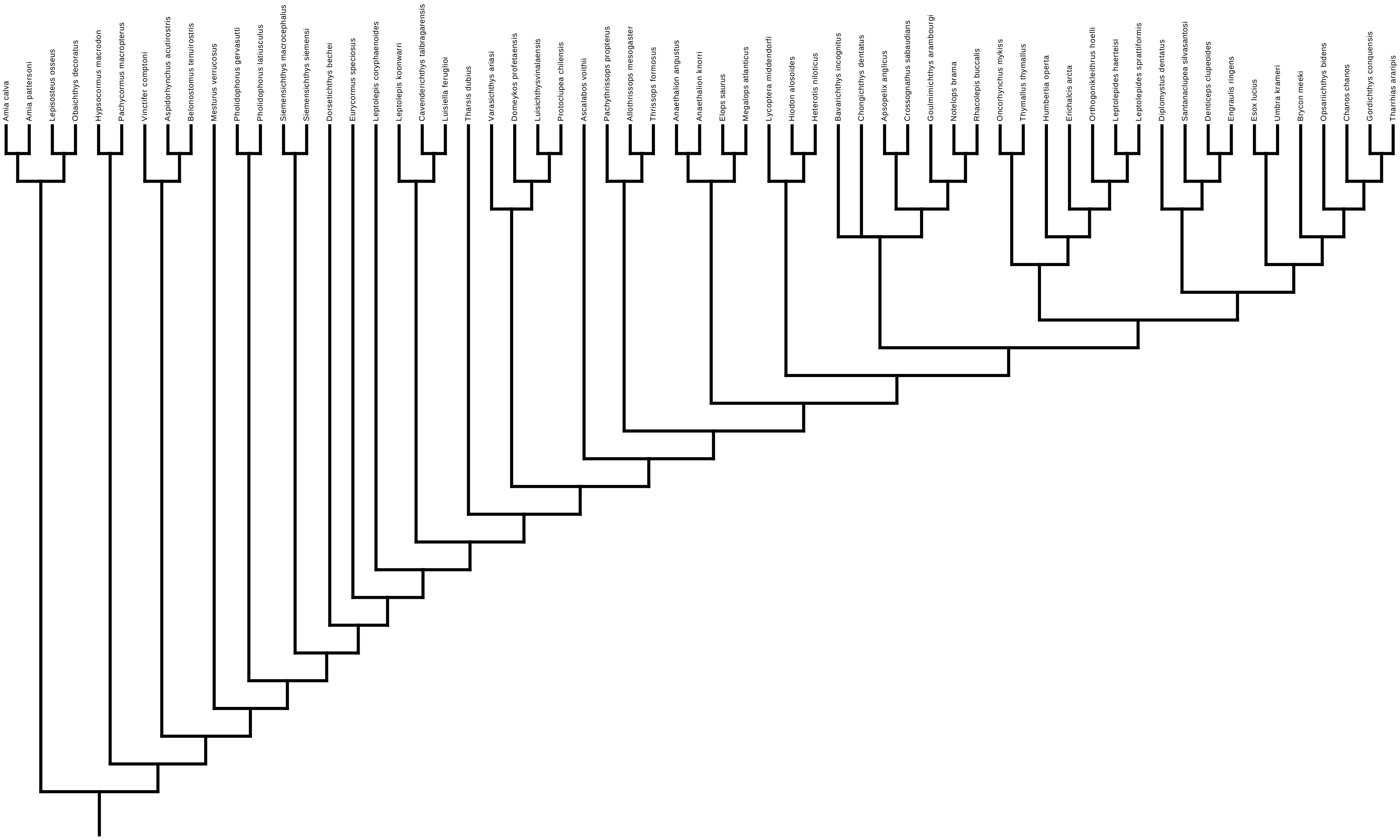
Amia calva
Amia pattersoni
Lepisosteus osseus
Obaichthys decoratus
Hypsocormus macrodon
Pachycormus macropterus
Vinctifer comptoni
Aspidorhynchus acutirostris
Belonostomus tenuirostris
Mesturus verrucosus
Pholidophorus gervasutti
Pholidophorus latiusculus
Siemensichthys macrocephalus
Siemensichthys siemensi
Dorsetichthys bechei
Eurycormus speciosus
Leptolepis coryphaenoides
Leptolepis koonwarri
Cavenderichthys talbragarensis
Luisiella feruglioi
Tharsis dubius
Varasichthys ariasi
Domeykos profetaensis
Luisichthysvinalaensis
Protoclupea chilensis
Ascalabos voithii
Pachythrissops propterus
Allothrissops mesogaster
Thrissops formosus
Anaethalion angustus
Anaethalion knorri
Elops saurus
Megalops atlanticus
Lycoptera middendorfi
Hiodon alosoides
Heterotis niloticus
Bavarichthys incognitus
Chongichthys dentatus
Apsopelix anglicus
Crossognathus sabaudians
Goulmimichthys arambourgi
Notelops brama
Rhacolepis buccalis
Oncorhynchus mykiss
Thymallus thymallus
Humbertia operta
Erichalcis arcta
Orthogonikleithrus hoelli
Leptolepides haerteisi
Leptolepides sprattiformis
Diplomystus dentatus
Santanaclupea silvasantosi
Denticeps clupeoides
Engraulis ringens
Esox lucius
Umbra krameri
Brycon meeki
Opsariichthys bidens
Chanos chanos
Gordichthys conquensis
Tharrhias araripis

MPT 8

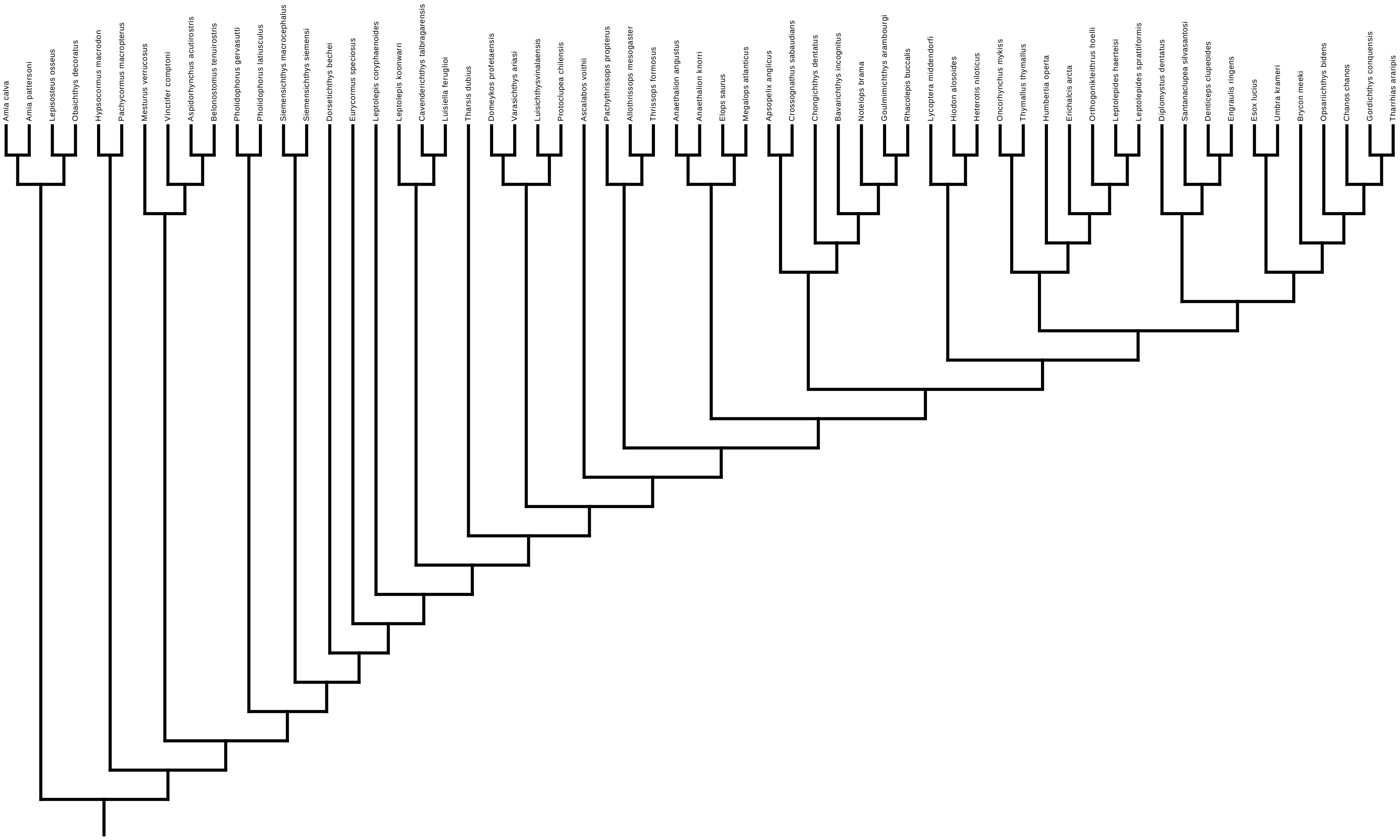
Amia calva
Amia pattersoni
Lepisosteus osseus
Obaichthys decoratus
Hypsocormus macrodon
Pachycormus macropterus
Mesturus verrucosus
Vinctifer comptoni
Aspidorhynchus acutirostris
Belonostomus tenuirostris
Pholidophorus gervasutti
Pholidophorus latiusculus
Siemensichthys macrocephalus
Siemensichthys siemensi
Dorsetichthys bechei
Eurycormus speciosus
Leptolepis coryphaenoides
Leptolepis koonwarri
Cavenderichthys talbragarensis
Luisiella feruglioi
Tharsis dubius
Domeykos profetaensis
Varasichthys ariasi
Luisichthysvinalaensis
Protoclupea chilensis
Ascalabos voithii
Pachythrissops propterus
Allothrissops mesogaster
Thrissops formosus
Anaethalion angustus
Anaethalion knorri
Elops saurus
Megalops atlanticus
Apsopelix anglicus
Crossognathus sabaudians
Chongichthys dentatus
Bavarichthys incognitus
Notelops brama
Goulmimichthys arambourgi
Rhacolepis buccalis
Lycoptera middendorfi
Hiodon alosoides
Heterotis niloticus
Oncorhynchus mykiss
Thymallus thymallus
Humbertia operta
Erichalcis arcta
Orthogonikleithrus hoelli
Leptolepides haerteisi
Leptolepides sprattiformis
Diplomystus dentatus
Santanaclupea silvasantosi
Denticeps clupeoides
Engraulis ringens
Esox lucius
Umbra krameri
Brycon meeki
Opsariichthys bidens
Chanos chanos
Gordichthys conquensis
Tharrhias araripis

MPT 9

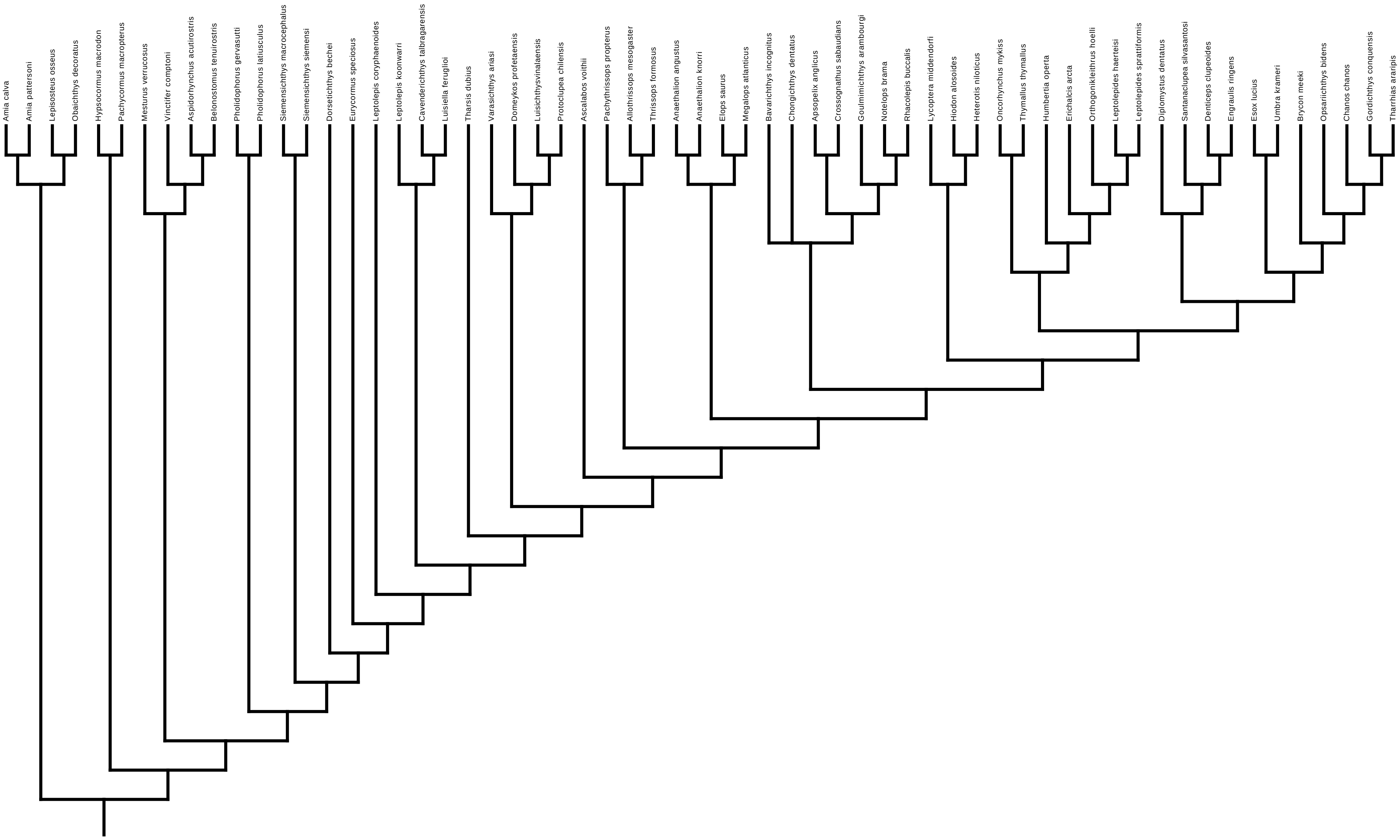
Amia calva
Amia pattersoni
Lepisosteus osseus
Obaichthys decoratus
Hypsocormus macrodon
Pachycormus macropterus
Mesturus verrucosus
Vinctifer comptoni
Aspidorhynchus acutirostris
Belonostomus tenuirostris
Pholidophorus gervasutti
Pholidophorus latiusculus
Siemensichthys macrocephalus
Siemensichthys siemensi
Dorsetichthys bechei
Eurycormus speciosus
Leptolepis coryphaenoides
Leptolepis koonwarri
Cavenderichthys talbragarensis
Luisiella feruglioi
Tharsis dubius
Varasichthys ariasi
Domeykos profetaensis
Luisichthysvinalaensis
Protoclupea chilensis
Ascalabos voithii
Pachythrissops propterus
Allothrissops mesogaster
Thrissops formosus
Anaethalion angustus
Anaethalion knorri
Elops saurus
Megalops atlanticus
Bavarichthys incognitus
Chongichthys dentatus
Apsopelix anglicus
Crossognathus sabaudians
Goulmimichthys arambourgi
Notelops brama
Rhacolepis buccalis
Lycoptera middendorfi
Hiodon alosoides
Heterotis niloticus
Oncorhynchus mykiss
Thymallus thymallus
Humbertia operta
Erichalcis arcta
Orthogonikleithrus hoelli
Leptolepides haerteisi
Leptolepides sprattiformis
Diplomystus dentatus
Santanaclupea silvasantosi
Denticeps clupeoides
Engraulis ringens
Esox lucius
Umbra krameri
Brycon meeki
Opsariichthys bidens
Chanos chanos
Gordichthys conquensis
Tharrhias araripis

MPT 10

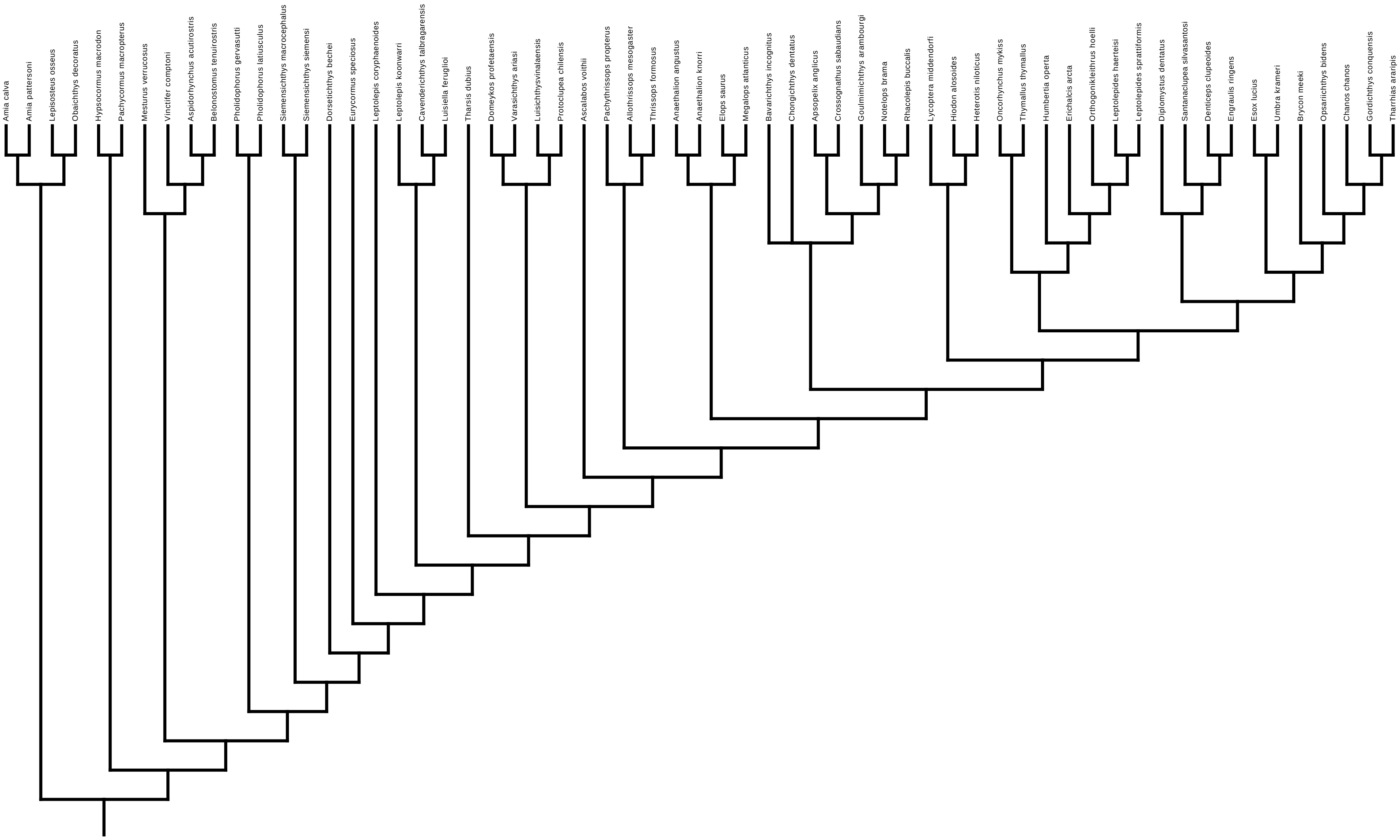
Amia calva
Amia pattersoni
Lepisosteus osseus
Obaichthys decoratus
Hypsocormus macrodon
Pachycormus macropterus
Mesturus verrucosus
Vinctifer comptoni
Aspidorhynchus acutirostris
Belonostomus tenuirostris
Pholidophorus gervasutti
Pholidophorus latiusculus
Siemensichthys macrocephalus
Siemensichthys siemensi
Dorsetichthys bechei
Eurycormus speciosus
Leptolepis coryphaenoides
Leptolepis koonwarri
Cavenderichthys talbragarensis
Luisiella feruglioi
Tharsis dubius
Domeykos profetaensis
Varasichthys ariasi
Luisichthysvinalaensis
Protoclupea chilensis
Ascalabos voithii
Pachythrissops propterus
Allothrissops mesogaster
Thrissops formosus
Anaethalion angustus
Anaethalion knorri
Elops saurus
Megalops atlanticus
Bavarichthys incognitus
Chongichthys dentatus
Apsopelix anglicus
Crossognathus sabaudians
Goulmimichthys arambourgi
Notelops brama
Rhacolepis buccalis
Lycoptera middendorfi
Hiodon alosoides
Heterotis niloticus
Oncorhynchus mykiss
Thymallus thymallus
Humbertia operta
Erichalcis arcta
Orthogonikleithrus hoelli
Leptolepides haerteisi
Leptolepides sprattiformis
Diplomystus dentatus
Santanaclupea silvasantosi
Denticeps clupeoides
Engraulis ringens
Esox lucius
Umbra krameri
Brycon meeki
Opsariichthys bidens
Chanos chanos
Gordichthys conquensis
Tharrhias araripis

MPT 11

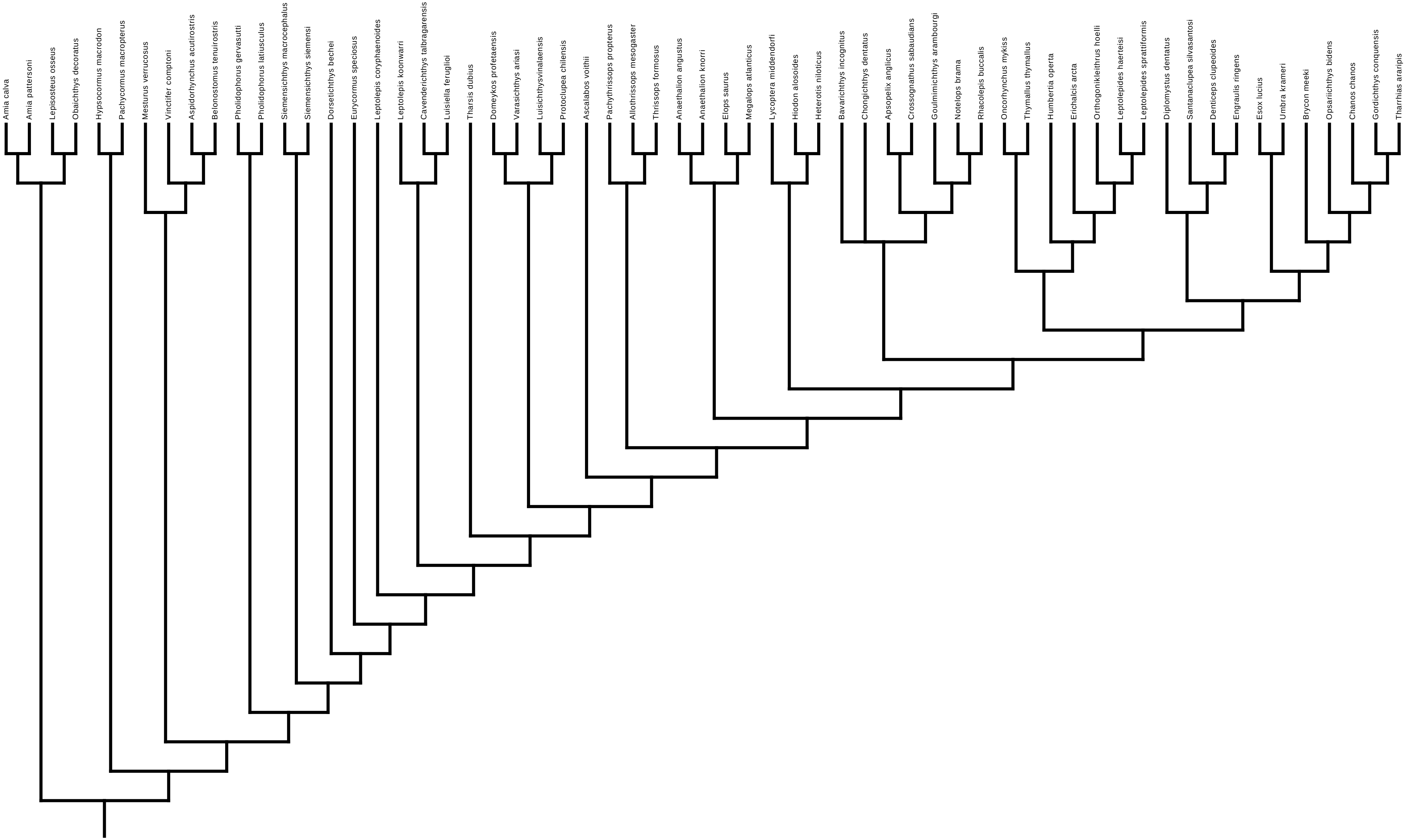
Amia calva
Amia pattersoni
Lepisosteus osseus
Obaichthys decoratus
Hypsocormus macrodon
Pachycormus macropterus
Mesturus verrucosus
Vinctifer comptoni
Aspidorhynchus acutirostris
Belonostomus tenuirostris
Pholidophorus gervasutti
Pholidophorus latiusculus
Siemensichthys macrocephalus
Siemensichthys siemensi
Dorsetichthys bechei
Eurycormus speciosus
Leptolepis coryphaenoides
Leptolepis koonwarri
Cavenderichthys talbragarensis
Luisiella feruglioi
Tharsis dubius
Domeykos profetaensis
Varasichthys ariasi
Luisichthysvinalaensis
Protoclupea chilensis
Ascalabos voithii
Pachythrissops propterus
Allothrissops mesogaster
Thrissops formosus
Anaethalion angustus
Anaethalion knorri
Elops saurus
Megalops atlanticus
Lycoptera middendorfi
Hiodon alosoides
Heterotis niloticus
Bavarichthys incognitus
Chongichthys dentatus
Apsopelix anglicus
Crossognathus sabaudians
Goulmimichthys arambourgi
Notelops brama
Rhacolepis buccalis
Oncorhynchus mykiss
Thymallus thymallus
Humbertia operta
Erichalcis arcta
Orthogonikleithrus hoelli
Leptolepides haerteisi
Leptolepides sprattiformis
Diplomystus dentatus
Santanaclupea silvasantosi
Denticeps clupeoides
Engraulis ringens
Esox lucius
Umbra krameri
Brycon meeki
Opsariichthys bidens
Chanos chanos
Gordichthys conquensis
Tharrhias araripis

MPT 12

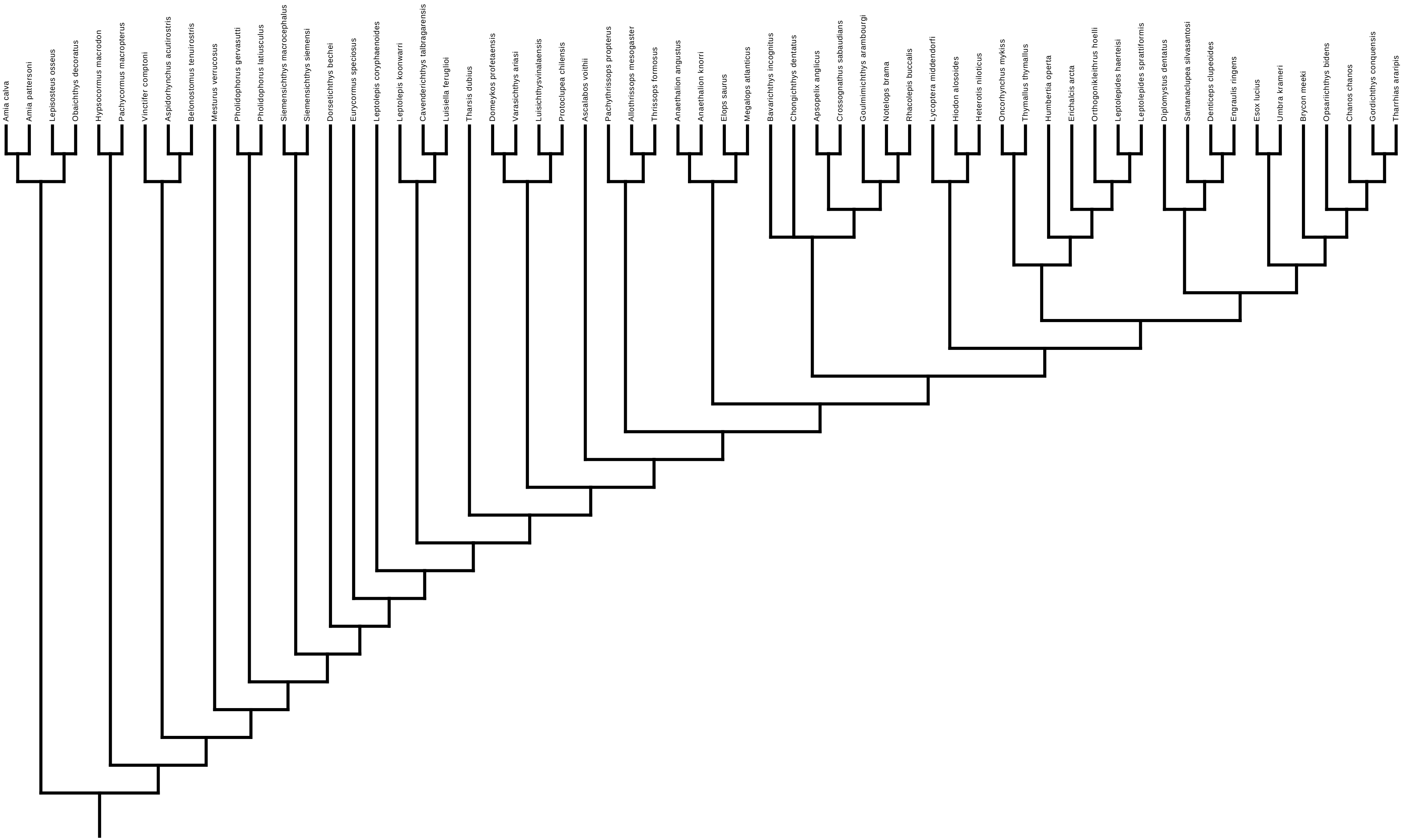
Amia calva
Amia pattersoni
Lepisosteus osseus
Obaichthys decoratus
Hypsocormus macrodon
Pachycormus macropterus
Vinctifer comptoni
Aspidorhynchus acutirostris
Belonostomus tenuirostris
Mesturus verrucosus
Pholidophorus gervasutti
Pholidophorus latiusculus
Siemensichthys macrocephalus
Siemensichthys siemensi
Dorsetichthys bechei
Eurycormus speciosus
Leptolepis coryphaenoides
Leptolepis koonwarri
Cavenderichthys talbragarensis
Luisiella feruglioi
Tharsis dubius
Domeykos profetaensis
Varasichthys ariasi
Luisichthysvinalaensis
Protoclupea chilensis
Ascalabos voithii
Pachythrissops propterus
Allothrissops mesogaster
Thrissops formosus
Anaethalion angustus
Anaethalion knorri
Elops saurus
Megalops atlanticus
Bavarichthys incognitus
Chongichthys dentatus
Apsopelix anglicus
Crossognathus sabaudians
Goulmimichthys arambourgi
Notelops brama
Rhacolepis buccalis
Lycoptera middendorfi
Hiodon alosoides
Heterotis niloticus
Oncorhynchus mykiss
Thymallus thymallus
Humbertia operta
Erichalcis arcta
Orthogonikleithrus hoelli
Leptolepides haerteisi
Leptolepides sprattiformis
Diplomystus dentatus
Santanaclupea silvasantosi
Denticeps clupeoides
Engraulis ringens
Esox lucius
Umbra krameri
Brycon meeki
Opsariichthys bidens
Chanos chanos
Gordichthys conquensis
Tharrhias araripis

MPT 13

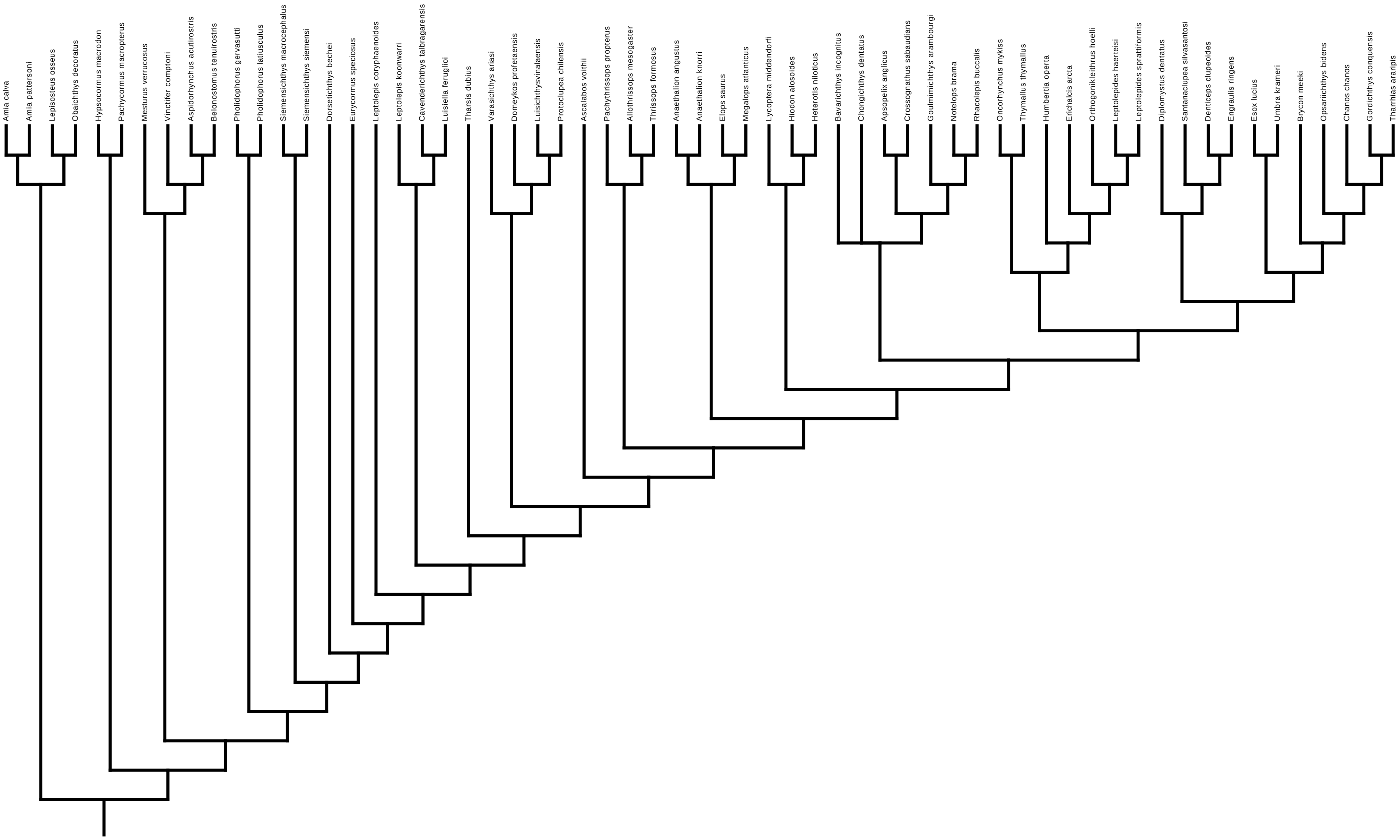

Amia calva
Amia pattersoni
Lepisosteus osseus
Obaichthys decoratus
Hypsocormus macrodon
Pachycormus macropterus
Mesturus verrucosus
Vinctifer comptoni
Aspidorhynchus acutirostris
Belonostomus tenuirostris
Pholidophorus gervasutti
Pholidophorus latiusculus
Siemensichthys macrocephalus
Siemensichthys siemensi
Dorsetichthys bechei
Eurycormus speciosus
Leptolepis coryphaenoides
Leptolepis koonwarri
Cavenderichthys talbragarensis
Luisiella feruglioi
Tharsis dubius
Varasichthys ariasi
Domeykos profetaensis
Luisichthysvinalaensis
Protoclupea chilensis
Ascalabos voithii
Pachythrissops propterus
Allothrissops mesogaster
Thrissops formosus
Anaethalion angustus
Anaethalion knorri
Elops saurus
Megalops atlanticus
Lycoptera middendorfi
Hiodon alosoides
Heterotis niloticus
Bavarichthys incognitus
Chongichthys dentatus
Apsopelix anglicus
Crossognathus sabaudians
Goulmimichthys arambourgi
Notelops brama
Rhacolepis buccalis
Oncorhynchus mykiss
Thymallus thymallus
Humbertia operta
Erichalcis arcta
Orthogonikleithrus hoelli
Leptolepides haerteisi
Leptolepides sprattiformis
Diplomystus dentatus
Santanaclupea silvasantosi
Denticeps clupeoides
Engraulis ringens
Esox lucius
Umbra krameri
Brycon meeki
Opsariichthys bidens
Chanos chanos
Gordichthys conquensis
Tharrhias araripis

MPT 14

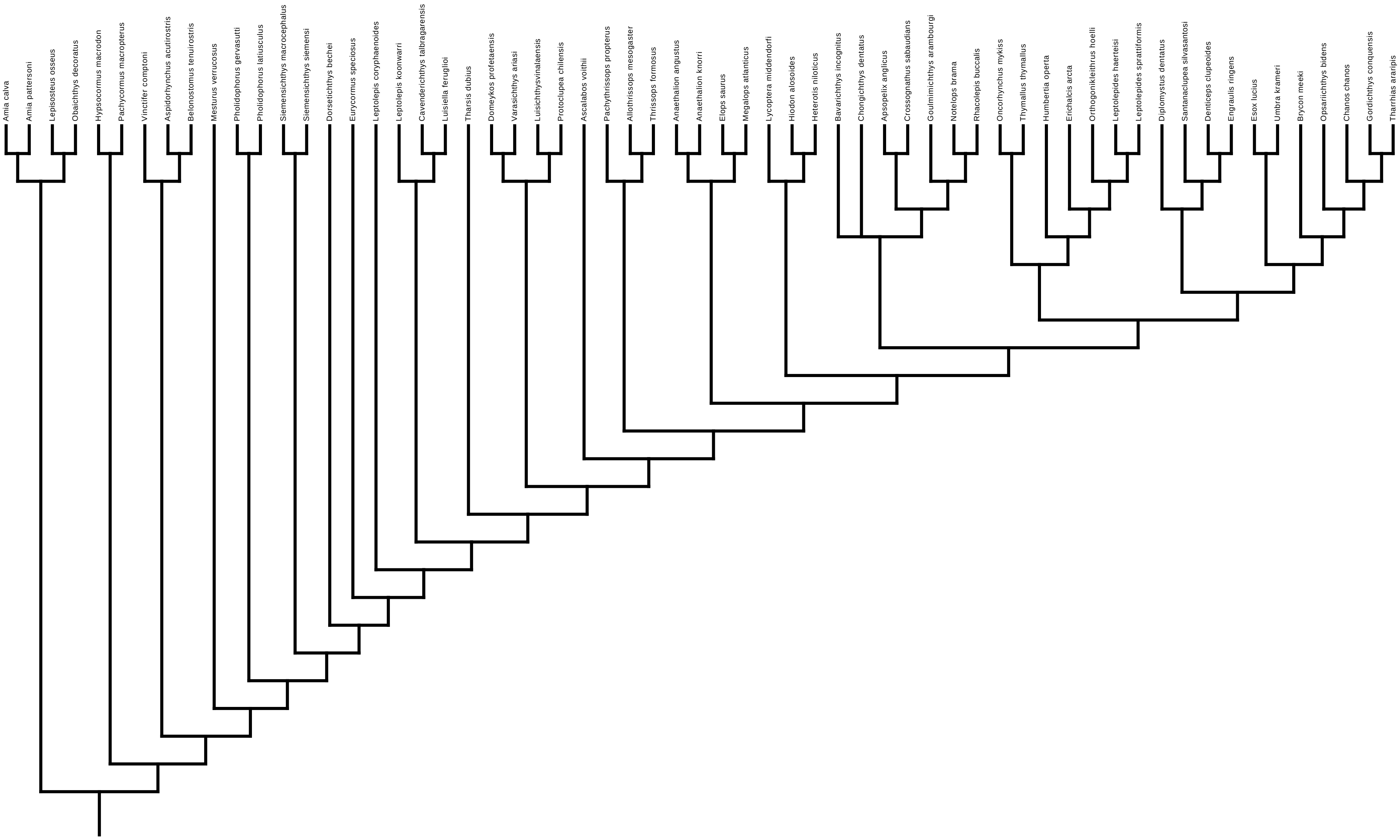
Amia calva
Amia pattersoni
Lepisosteus osseus
Obaichthys decoratus
Hypsocormus macrodon
Pachycormus macropterus
Vinctifer comptoni
Aspidorhynchus acutirostris
Belonostomus tenuirostris
Mesturus verrucosus
Pholidophorus gervasutti
Pholidophorus latiusculus
Siemensichthys macrocephalus
Siemensichthys siemensi
Dorsetichthys bechei
Eurycormus speciosus
Leptolepis coryphaenoides
Leptolepis koonwarri
Cavenderichthys talbragarensis
Luisiella feruglioi
Tharsis dubius
Domeykos profetaensis
Varasichthys ariasi
Luisichthysvinalaensis
Protoclupea chilensis
Ascalabos voithii
Pachythrissops propterus
Allothrissops mesogaster
Thrissops formosus
Anaethalion angustus
Anaethalion knorri
Elops saurus
Megalops atlanticus
Lycoptera middendorfi
Hiodon alosoides
Heterotis niloticus
Bavarichthys incognitus
Chongichthys dentatus
Apsopelix anglicus
Crossognathus sabaudians
Goulmimichthys arambourgi
Notelops brama
Rhacolepis buccalis
Oncorhynchus mykiss
Thymallus thymallus
Humbertia operta
Erichalcis arcta
Orthogonikleithrus hoelli
Leptolepides haerteisi
Leptolepides sprattiformis
Diplomystus dentatus
Santanaclupea silvasantosi
Denticeps clupeoides
Engraulis ringens
Esox lucius
Umbra krameri
Brycon meeki
Opsariichthys bidens
Chanos chanos
Gordichthys conquensis
Tharrhias araripis

MPT 15

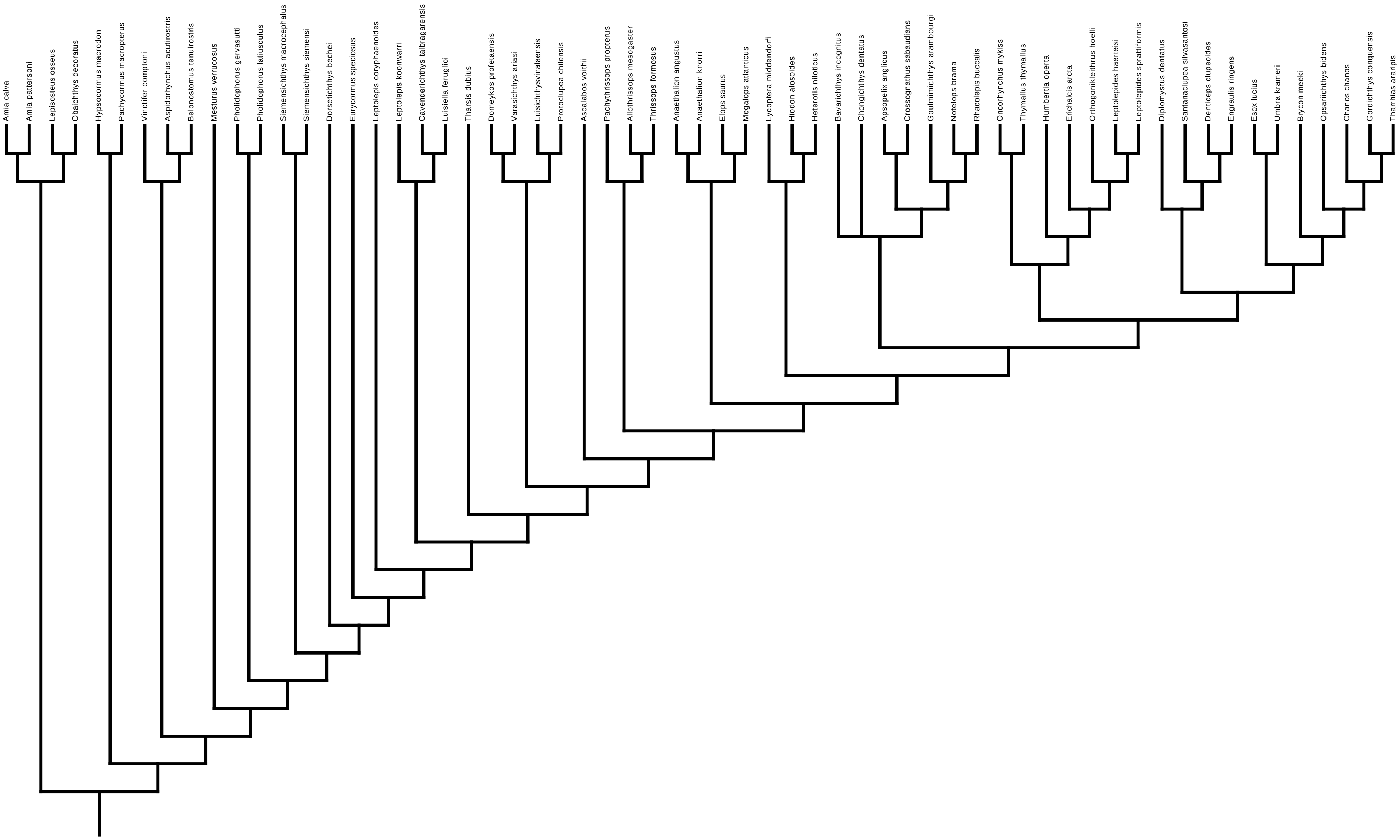
Amia calva
Amia pattersoni
Lepisosteus osseus
Obaichthys decoratus
Hypsocormus macrodon
Pachycormus macropterus
Vinctifer comptoni
Aspidorhynchus acutirostris
Belonostomus tenuirostris
Mesturus verrucosus
Pholidophorus gervasutti
Pholidophorus latiusculus
Siemensichthys macrocephalus
Siemensichthys siemensi
Dorsetichthys bechei
Eurycormus speciosus
Leptolepis coryphaenoides
Leptolepis koonwarri
Cavenderichthys talbragarensis
Luisiella feruglioi
Tharsis dubius
Domeykos profetaensis
Varasichthys ariasi
Luisichthysvinalaensis
Protoclupea chilensis
Ascalabos voithii
Pachythrissops propterus
Allothrissops mesogaster
Thrissops formosus
Anaethalion angustus
Anaethalion knorri
Elops saurus
Megalops atlanticus
Lycoptera middendorfi
Hiodon alosoides
Heterotis niloticus
Bavarichthys incognitus
Chongichthys dentatus
Apsopelix anglicus
Crossognathus sabaudians
Goulmimichthys arambourgi
Notelops brama
Rhacolepis buccalis
Oncorhynchus mykiss
Thymallus thymallus
Humbertia operta
Erichalcis arcta
Orthogonikleithrus hoelli
Leptolepides haerteisi
Leptolepides sprattiformis
Diplomystus dentatus
Santanaclupea silvasantosi
Denticeps clupeoides
Engraulis ringens
Esox lucius
Umbra krameri
Brycon meeki
Opsariichthys bidens
Chanos chanos
Gordichthys conquensis
Tharrhias araripis

MPT 16
